# Gender-different effect of Src family kinases antagonism on photophobia and trigeminal ganglion activity

**DOI:** 10.1186/s10194-024-01875-3

**Published:** 2024-10-11

**Authors:** Zhuoan Huang, Junyu Yao, Lingdi Nie, Xinchen Nie, Xuechunhui Xiong, Sulev Kõks, John P. Quinn, Aditi Kanhere, Minyan Wang

**Affiliations:** 1 Department of Biological Sciences, School of Science, Centre for Neuroscience, Xi’an Jiaotong-Liverpool University, Suzhou, China; 2 Institute of Systems, Molecular and Integrative Biology, University of Liverpool, Liverpool, UK; 3 Centre for Molecular Medicine and Innovative Therapeutics, Perron Institute for Neurological and Translational Science, Murdoch University, Perth, WA Australia; 4 Wisdom Lake Academy of Pharmacy, Xi’an Jiaotong-Liverpool University, Suzhou, China

**Keywords:** Src family kinases, Transient receptor potential ankyrin 1, Light aversion, Migraine, Trigeminal ganglion, Gender

## Abstract

**Background:**

Src family kinases (SFKs) contribute to migraine pathogenesis, yet its role in regulating photophobia behaviour, one of the most common forms of migraine, remains unknown. Here, we addressed whether SFKs antagonism alleviates photophobia behavior and explored the underlying mechanism involving hypothalamus and trigeminal ganglion activity, as measured by the alteration of neuropeptide levels and transcriptome respectively.

**Methods:**

A rapid-onset and injury-free mouse model of photophobia was developed following intranasal injection of the TRPA1 activator, umbellulone. The role of SFKs antagonism on light aversion was assessed by the total time the mouse stays in the light and transition times between the dark and light compartments. To gain insight to the preventive mechanism of SFKs antagonism, hypothalamic neuropeptides levels were assessed using enzyme linked immunofluorescent assay and trigeminal ganglion activity were assessed using RNA-sequencing and qPCR analysis.

**Results:**

SFKs antagonism by a clinically relevant SFKs inhibitor saracatinib reduced the total time in light and transition times in male mice, but not in females, suggesting SFKs play a crucial role in photophobia progressing and exhibit a male-only effect. SFKs antagonism had no effect on hypothalamic calcitonin gene-related peptide and pituitary adenylate cyclase-activating polypeptide levels of all mice investigated, suggesting the gender-different effect of saracatinib on light aversion appears to be independent of these hypothalamic neuropeptide levels. In trigeminal ganglion of male mice, photophobia is associated with profound alteration of differentially expressed genes, part of which were reversed by SFKs antagonism. Subsequent qPCR analysis showed SFKs antagonism displayed gender-different modulation of expression in some candidate genes, particularly noteworthy those encoding ion channels (*trpm3*,* Scn8a)*, ATPase signaling (*crebbp*,* Atp5α1)* and kinase receptors (*Zmynd8*,* Akt1*).

**Conclusions:**

In conclusion, our data revealed that SFKs antagonism reduced photophobia processing in male mice and exhibited gender-different modulation of trigeminal ganglion activity, primarily manifesting as alterations in the transcriptome profile. These findings underscore the potential of SFKs antagonism for allieving photophobia in males, highlighting its value in the emerging field of precision medicine.

**Supplementary Information:**

The online version contains supplementary material available at 10.1186/s10194-024-01875-3.

## Background

Photophobia, a prevalent manifestation of migraine, can induce eye pain and headaches, particularly affecting women post-puberty [1, 2] and impacts up to 90% of patients, while 75% of migraine sufferers experience persistent light sensitivity between attacks. The co-occurrence of photophobia and migraine headache involves intricate interactions within various brain regions, including retina, trigeminal ganglion (TG), midbrain, hypothalamus, thalamus and visual cortex [3, 4]. Photic signals from retinal ganglion cells, activated by migraine triggers, converge with trigeminal nociceptive signals in the thalamus, projecting to the somatosensory and visual cortex [5, 6]. Communication between these pathways in photophobia and migraine may depend on calcitonin gene-related peptide (CGRP) or pituitary cyclase-activating polypeptide (PACAP) transmission [3, 7–9]. Current therapies for photophobia rely primarily on optical means of modulating the pathway, botulinum toxin, and CGRP pathway inhibition [6]. However, the exact molecular mechanism underlying migraine photophobia has been incompletely elucidated and many patients have persistent photophobia despite the use of these therapies.

Src family kinase (SFKs) are composed of four domains - a unique domain varying between family members, Src Homology 3, Src Homology 2, kinase domain and a C-terminal regulatory segment. Sufficient research findings have held notable significance on the pivotal role of this family kinases in migraine pathogenesis. Activation of SFKs via phosphorylation of the tyrosine 416 site within the kinase domain has been found to facilitate the cortical spreading depression propagation [10, 11] and neuroinflammation [12, 13]. Also, SFKs promote trigeminal ganglion sensitization via facilitating cross talk between cytokine and CGRP [13] and alter changes in cerebral blood flow [14] in rodent migraine models [12–15]. Furthermore, this family kinases serve as a regulatory hub for various signals that promote the transmission of migraine pain signals. SFKs activation enhances central sensitization through NR2A-containing receptors and purinergic P2 × 7 receptor signaling via coupling to pannexin-1 [10, 12]. In the periphery, SFKs activity is essential for TG sensitization in mice via CGRP signaling and the stress-sensing cation channel, transient receptor potential ankyrin 1 (TRPA1) [15]. These pieces of evidence imply therapeutic potential of SFKs-targeted drugs for migraine. Yet it is not known whether and how SFKs play a role in photophobia processing.

In this study, we explored whether SFKs antagonism reduces photophobia behavior in mice and explored its mechanism involving hypothalamus and trigeminal ganglion activation. Conventional photophobia models are usually more invasive, often necessitating single or repeated injection of chemicals, such as CGRP [16, 17] or nitroglycerin [18]. Most of them involve invasive injections such as intracerebral ventricle injection [9, 17] or intraperitoneal injections [7, 19]. Umbellulone (UMB), is a volatile activator of TRPA1 that is known to play a crucial role in migraine progression [15, 20, 21]. In our study, we applied UMB and successfully induced rapid-onset and injury-free mouse photophobia behavior. Using this model, we found that SFKs antagonism by a clinical relevant SFKs inhibitor, saracatinib (SRCT) [22] exhibited gender-different alleviation of photophobia behavior in mice and distinct expression modulation of candidate genes in the trigeminal ganglion. Given the known role of CGRP and PACAP in migraine and that they display independent mechanisms in inducing light aversion in CD-1 mice [7], we also investigated their potential involvement in the hypothalamus, a brain region important for the induction of photophobia [4, 23].

## Methods

### Animals

A total of 41 adult male C57BL/6J mice (21.0 ± 0.20 g, mean ± SEM) and 40 adult female C57BL/6J mice (18.3 ± 0.13 g), age at 6–7 weeks were purchased from Shanghai SLAC Laboratory Animal Corporation Ltd. China. Mice were sourced from four cohorts and housed in groups of 3–5 unless otherwise indicated, on a 12 h light cycle with food and water *ad libitum.* All behavior experiments were housed in the Experimental Animal Centre of Soochow University for at least one week and acclimated to the housing room before use. For all experiments, investigators were blinded. Animal procedures were approved by the Ethical Review Panels of Xi’an Jiaotong–Liverpool University under the agreement with Soochow University and performed in accordance with relevant China national and provincial guidelines. Ethical approval code: ER-UEC-1,088,616,620,221,018,145,157.

### Light aversion assay in mice and experimental design

Photophobia behavior test was performed between 7 am and noon in a quiet and dark room with constant temperature at 25 ℃. Mice were individually monitored in a light aversion chamber that was divided into two equally-sized compartments: one painted white without a top; the other one painted black with a black ceiling. A small opening between the two compartments allowed the mouse to freely move in between. A circular light source (J&K Photoelectronic) and a near-infrared camera (Hikvision) were both placed on the top of the white zone. A video tracking system (Hikvision) composed of the camera, a video recorder and a monitoring software were used to observe and analyze mice behavior.

In order to investigate the effects of SRCT (S1006, Selleckchem) on light aversion, we developed an acute injury-free photophobia model by intranasal injection (*i.n.*) of a selective TRPA1 agonist UMB (SML0782 Sigma-Aldrich) that was previously reported to trigger headache pain [21, 24, 25] and induce cutaneous allodynia [26]. The volatile property enabled the drug intranasally administered for inducing an acute and injury-free light aversion response in C57BL/6J mice under bright light.

**Fig. 1:**
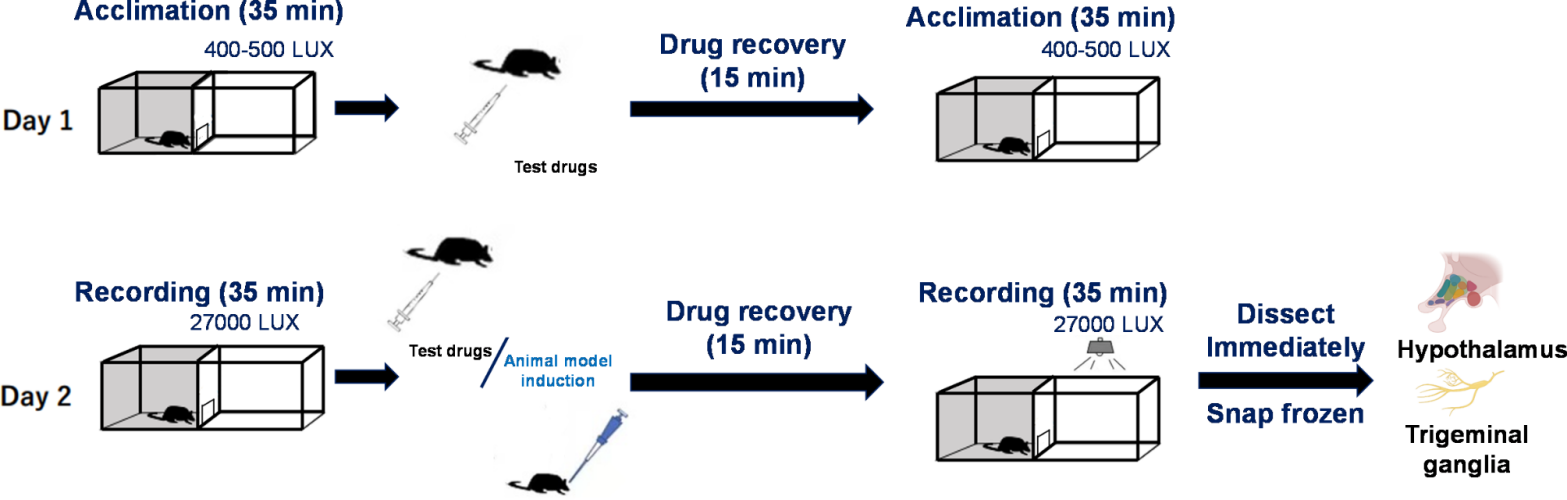
Experimental flowchart showing light aversion behavior induction paradigm. On day 1, each mouse was acclimated in the testing chamber with the light (400–500 lx) on for 35 min twice and *i.p*. administration of the test drugs was conducted in between the acclimation. On day 2, each mouse was acclimated with the same timeline as on day 1: Mouse behavior was recorded in the testing chamber for 35 min under light intensity of 27,000 lx [17] as the self-control. The mouse was then *i.p.* administered with the test drugs, immediately followed by *i.n.* administration of either 1 µmol/kg UMB or its vehicle 0.2% dimethyl sulfoxide (DMSO). Subsequently, mouse light aversion behavior was recorded for another 35 min. After light aversion recording, mice were immediately sacrificed by rapid cervical dislocation, dissected for the hypothalamus and TG. Due to the small amounts of tissue, the left and right hypothalamus and TG of each mouse were merged respectively, rapidly homogenated within 15 s in liquid nitrogen, aliquoted and stored in -80 degree for subsequent analysis

The experimental procedures were conducted in consecutive two days (Fig. 1) to ensure both the well-being of the animals and the efficacy of pretreatment of SRCT within the acute model framework. Kaiser EA and colleagues reported that bright light intensity of 27,000 lx, but not dim light (400–500 lx), inside the white zone is required to successfully induce light aversion behavior after a single intraperitoneal injection (*i.p*). of CGRP [17]. In our experiment, the dim light was applied on the acclimation day (day 1) to let mice get accustomed to the light/dark box environment and to reduce any stress, whilst bright light intensity was applied on day 2 to trigger light aversion responses before and after drug injections. On day 1, each mouse was acclimated in the testing chamber with the dim light for 35 min twice. Intraperitoneal administration of the test drugs, either SRCT at 20 mg/kg or its vehicle, 0.25% DMSO (D2650, Sigma-Aldrich), was carried out in between the acclimation. On day 2, each mouse was acclimated with the same timeline as on day 1. Mouse behavior was recorded in the testing chamber for 35 min under bright light [17] as the self-control. The mouse was intraperitoneally administered with 20 mg/kg SRCT or the vehicle, immediately followed by *i.n.* administration of either 1 µmol/kg UMB or its vehicle 0.2% DMSO. Subsequently, mouse light aversion behavior was recorded for another 35 min. After light aversion recording, mice were immediately sacrificed by rapid cervical dislocation, dissected for the hypothalamus and TG. Due to the small amounts of tissue, it was necessary to combine the left and right hypothalamus and TG of each mouse respectively. All the merged tissue samples were rapidly homogenated within 15 s in liquid nitrogen, aliquoted and stored in -80 degree (906GP-ULTS, Thermo Scientific, Finland) for subsequent analysis.

**Table 1:** Overview of the photophobia mice test groups, models and test drugs

Group	Animal model	Test drugs	Animal numbers
**Male**			
Group 1	Control (*i.n.* 0.2% DMSO, vehicle 1)	*i.p.* 20 mg/kg DMSO (vehicle 2)	*n* = 10
Group 2	Photophobia (*i.n*. 150 µg/kg UMB)	*i.p.* 20 mg/kg DMSO (vehicle 2)	*n* = 11
Group 3	Photophobia (*i.n*. 150 µg/kg UMB)	*i.p.* 20 mg/kg SRCT	*n* = 10
**Female**			
Group 4	Control (*i.n*. 0.2% DMSO, vehicle 1)	*i.p.* 20 mg/kg DMSO (vehicle 2)	*n* = 12
Group 5	Photophobia (*i.n.* 150 µg/kg UMB)	*i.p*. 20 mg/kg DMSO (vehicle 2)	*n* = 13
Group 6	Photophobia (*i.n.* 150 µg/kg UMB)	*i.p.* 20 mg/kg SRCT	*n* = 12

**Abbreviations**: UMB, Umbellulone, TRPA1 agonist; SRCT, Saracatinib, SFKs inhibitor. *i.n*. intranasal; *i.p.*, intraperitoneal

There were 3 experimental groups (Table 1) for each gender: (i) control (vehicle 1, *i.n.*, vehicle 2, *i.p.*) (ii) photophobia (1 µmol/kg UMB, *i.n.*, vehicle 2, *i.p.*) (iii) photophobia (1 µmol/kg UMB, *i.n.*) + SRCT (20 mg/kg, *i.p.*). Individual numbers of animals used for each experiment are shown in Table 1 below. Animals from each cohort were randomly allocated to different experimental groups with the same experimental procedure in order to reduce bias and minimize potential confounders, particularly considering mice were sourced from different cohorts and the potential influence of mice remembering the light/dark configuration on light aversion behavior under bright light [17]. For these studies, total 81 mice were tested, 13 of which were excluded for preliminary experiments. Hence, data are from a total of 68 mice (31 male, 37 female). Expected effect sizes were based on previous publications with light aversion [16] and the drug SRCT action in migraine models [12, 13]. Two behavioral parameters were assessed to reflect the degree of light avoidance: total time in light (the total time that the mouse spent in the light compartment) and transitions (the times that the mouse moved between the light and dark compartments). Data were presented as absolute values or fold change relative to respective self-control of each experimental group.

### Enzyme-linked immunosorbent assay

In order to determine whether the effect of SFKs antagonism on photophobia is associated with hypothalamic CGRP and PACAP, the merged hypothalamus homogenate of each male and female mouse was prepared. Protein extraction and detection was conducted following the manufacture’s instructions using the CGRP enzyme-linked immunosorbent assay kit (CSB-Eq. 027706MO, Cusabio) or PACAP kit (LS-F5688, LifeSpan BioScience). Briefly, 1 ml/100 mg tissue homogenates were reconstituted with enzyme immunoassay buffer, centrifuged for 5 min at 5000 *g* at 2–8 °C. The supernatant was aliquoted and stored at -80 °C. Immediately before assay, samples were reconstituted with enzyme immunoassay buffer, and analyzed using a microplate reader. CGRP and PACAP levels are presented as mean ± SEM.

### cDNA library construction and RNA sequencing

In order to identify if TG activation is involved in the UMB-induced acute light aversion and if such activation is sensitive to SFKs antagonism, we conducted transcriptomic analysis. To minimize the cost, we randomly selected TG of 7 male mice from each experimental group. The total RNA of the mouse TG was extracted using TRIZOL reagent (T9424 Sigma-Aldrich, St. Louis, MO, USA). The RNA samples were shipped to Beijing Genomics Institute (BGI, Shenzhen, China) for cDNA library construction using MGIEasy RNA Library Prep Kit (1000005276, MGI, Shenzhen, China). The library was amplified using Phi29 DNA polymerase (A39392, Thermo Fisher Scientific, Waltham, MA, USA) to generate DNA nanoballs (DNBs) containing more than 300 copies. Finally, the DNBs were sequenced using combinational probe anchor synthesis-based DNBSEQ-500 sequencer (BGI, Shenzhen, China).

Mouse reference genome version GRCm39 was used for whole genome transcriptome analysis. Salmon was used to quantify the transcripts of individual samples [27]. For differential analysis of the transcript expressions, DEseq2 package and its functions were used. Comparisons between photophobia vs. control and photophobia_SRCT vs. photophobia were performed and false discovery rate correction was applied for nominal *p*-values. In order to make comparisons among the three groups, genes with multiple Ensembl Gene IDs were consolidated. Differentially expressed genes (DEGs) were determined as those with an adjusted *P*-value < 0.05. The Venn diagrams were generated using VennDiagram R package [28]. Gene Ontology (GO) enrichment analysis was applied to DEGs using the clusterProfiler R package. Pathway analysis was performed to determine the functions and signalling pathways of the biological system related to DEGs according to the annotation of the Kyoto Encyclopedia of Genes and Genomes (KEGG) database. The clusterProfiler R package [29] was used to test the statistical enrichment of DEGs in KEGG pathways. GO terms and KEGG pathways with adjusted *P*-value < 0.05 were considered statistically significant, and the “BH” method of Benjamini and Hochberg was used to control the false discovery rate.

### Quantitative polymerase chain reaction (qPCR)

In order to validate RNA-sequencing data in male mice and compare gene expression of selected genes of TG in both sexes, we conducted qPCR analysis. The total RNA extracted from TG homogenate of each mouse in the three experimental groups were reversed to cDNA using GoScript Reverse Tran-scription System (A5001, Promega). DEGs with |log2FoldChange|>20 and migraine-related genes were selected for further real-time quantitative PCR (qPCR) analysis. Peptidylprolyl isomerase A and β-actin were used as reference genes. Primers were designed using Primer Premier 5 and validated prior to qPCR analysis, which have been shown in Table 2. The qPCR reaction was performed using GoTaq qPCR Master Mix (A6002, Pro-mega) in QuantStudio 5 Real-Time PCR System (Applied Biosystems). The level of individual gene mRNA was normalized to the geometric mean of β-actin and PPIA using the 2 ^−(ΔCt)^ method.

**Table 2:** The forward and reverse primer sequences of selected genes for qPCR analysis

Gene	Gene ID	Primer sequences (5’ − 3’)		
*Crebbp*	XM_006521754.5	Forward	GGAAAGCCTGCCAAGCCAT	
		Reverse	TGGAACTGGGGTCTATGGGA	
*Trpm3*	XM_036161558.1	Forward	TATGTACCATAGGTATCGCCC	
		Reverse	GGTATGGTCGGACTACATCTC	
*Zmynd8*	NM_001363018.1	Forward	TGCCCGTTTTAGCTTGGCT	
		Reverse	TAGGTTCTTTGGAGCGAGTAG	
*Akt1*	NM_009652.4	Forward	CTTCTATGGTGCGGAGATTGT	
		Reverse	TCCTTGTCCAGCATGAGGTT	
*Atp5a1*	NM_007505.2	Forward	GCCCTCGGTAATGCTATTGA	
		Reverse	GCAATCGATGTTTTCCCAGT	
*Scn8a*	NM_011323.3	Forward	TGGTCCAAGAATGTGGAGTACA	
		Reverse	CTGAGACATTGCCCAGGTCC	
*ACTB*	NM_007393.5	Forward	CTGTCCACCTTCCAGCAGAT	
		Reverse	CGCAGCTCAGTAACAGTCCG	
*PPIA*	NM_017101.1	Forward	TTGCTGCAGACATGGTCAAC	
		Reverse	TGTCTGCAAACAGCTCGAAG	

### Data presentation and statistical analysis

Raw experimental data were analysed using GraphPad Prism 9.4.1. Normality test was performed for all quantitative data by Shapiro-Wilk test. For comparisons between two independent groups, if the data passed the normality test, the data were presented as mean ± SEM and analyzed by one-way ANOVA was used for comparison of independent groups, followed by two-tailed unpaired t-test; if not, the data were analyzed by Kruskal–Wallis followed by Mann-Whitney test. The criteria used for data exclusion during the analysis is that, for a normally distributed data set, the outlier points were removed by eliminating any points that were beyond (mean ± 3* standard deviation); For an abnormally distributed data set, the outlier points were removed by eliminating any points that were beyond (Q3 + 1.5*IQR) (Q1: first quartile, Q3: third quartile, IQR: interquartile range). For data between two dependent experimental groups (before and after pre-treatment of test drugs), paired t-test was used for comparison. Data presentation of each quantitative data set was described in respective figure legend. Significant differences were shown by *P <* 0.05, *P <* 0.01, *P <* 0.001, or *P <* 0.0001.

## Results

### Saracatinib reduced light aversion in male mice

Expanding on established light aversion models induced by chemicals, such as nitroglycerin or CGRP injections, which typically require 5–11 days for induction [18, 30]; we have developed a novel photophobia model induced by *i.n.* administration of UMB (150 µg/kg), under bright light in C57BL/6J mice (Fig. 1). This preparation allowed the induction of a rapid-onset and injury-free light aversion behavior of mice after direct application of UMB or its vehicle to the upper intranasal region, thereby favoring subsequent behavior observation.

**Fig. 2:**
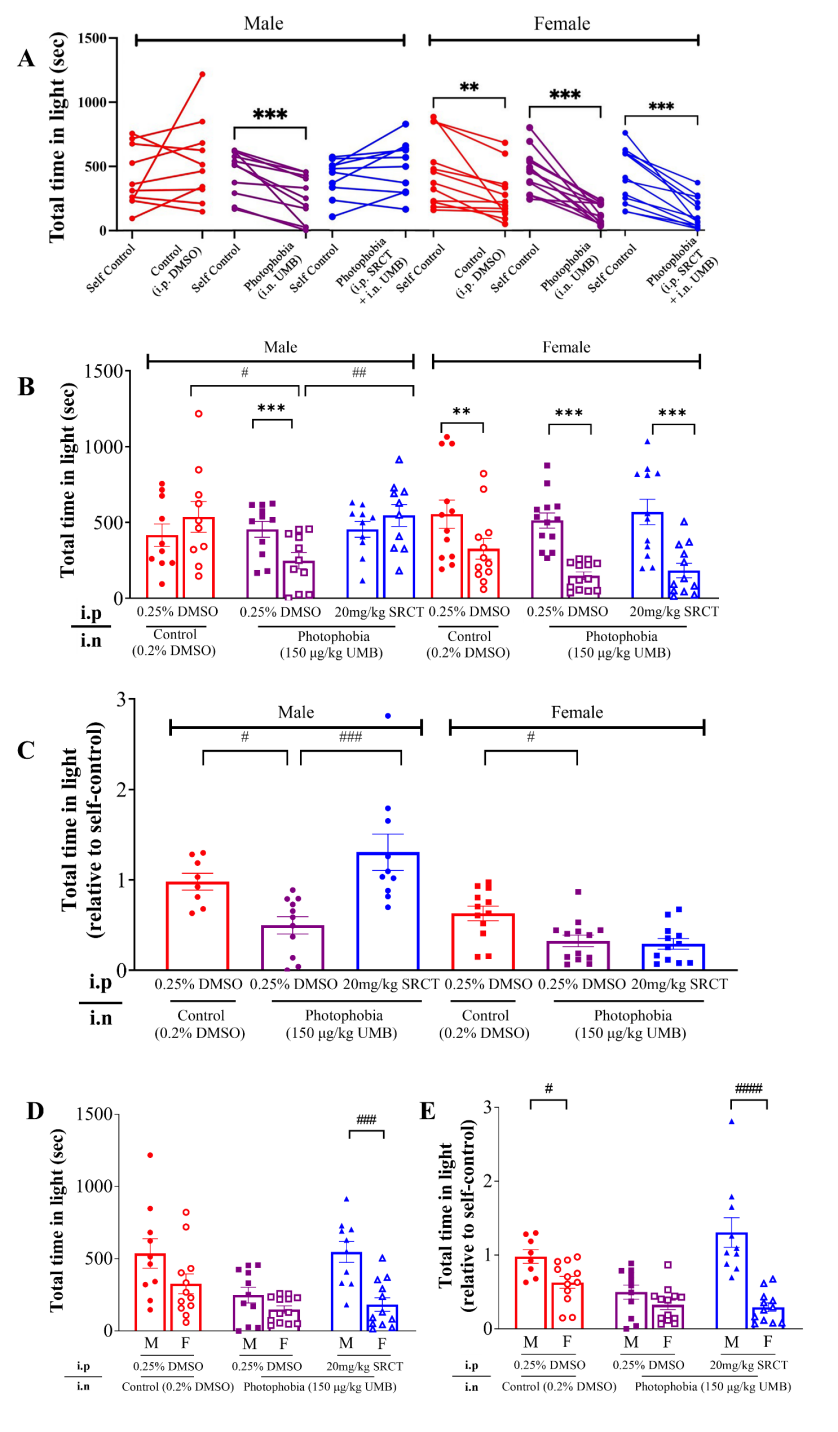
SFKs antagonism by SRCT displayed gender different effects on the total time in light in C57BL/6J photophobia mice induced by intranasal injection of UMB. Light aversion was induced by intranasal injection (*i.n.*) of UMB (150 µg/kg). 0.2% DMSO was injected as the control. There was total three experimental groups in each sex: (1) Control (0.25% DMSO, vehicle, *i.p.*), photophobia (0.25% DMSO, vehicle, *i.p.*) and photophobia with the SFKs inhibitor, SRCT (20 mg/kg, *i.p.*) groups. SRCT or its vehicle was intraperitoneally injected for 2 consecutive days prior to photophobia induction. Light aversion behavior of each mouse was recorded before and after light aversion induction for self-comparison in both male (M) and female (F) mice. (**A**, **B**) Total time in light (sec) before and after light aversion induction. (**C**) Total time in light recorded after light aversion induction relative to self-control. (**D**) Comparison of total time in light (sec) recorded after light aversion induction between male and female mice. (**E**) Comparison of total time in light relative to self-control recorded after light aversion induction between male and female mice. Data are shown as mean ± SEM. Significant difference was shown by * *P <* 0.05, ** *P <* 0.01, *** *P <* 0.001, or **** *P <* 0.0001; # *P <* 0.05, ## *P <* 0.01, ### *P <* 0.001, #### *P <* 0.0001. * Indicates comparison between dependent groups and ^#^ indicates comparison between independent groups

Behavior of each mouse was recorded before and after light aversion induction as indicated by the total time in light (Fig. 1). In male mice that were administered with the vehicle (0.2% DMSO, *i.n.)*, the total time in light was unaltered before (416.3 ± 74.08 sec) and after (536.8 ± 102.0 sec) administration of the vehicle (0.2% DMSO *i.n.*) (*P =* 0.2849, *n =* 10, Fig. 2A and B), suggesting mice’ memory effects in the bright light does not influence the second behavior recording. Differently, UMB (150 µg/kg, *i.p.*) led to a significant reduction of the total time in light (248.2 ± 53.85 s) compared to its self-control (455.1 ± 52.34 s, *P =* 0.0010, *n =* 11, Fig. 2A and B). This data was consistent with the observation that the relative total time in light was significantly lower in the UMB group (0.50) compared to that in the control group (0.98) (*P =* 0.0185, Fig. 2C) when the confounding factor due to mice’s memory effects in the bright light was eliminated by normalizing the data to the self-control.

Female mice in the vehicle group were more sensitive than male to the intranasal injection of the vehicle with a marked reduction of the total time in light (326.4 ± 68.25 sec) compared to the self-control (554.5 ± 93.12 sec, *P =* 0.0070, *n =* 12, Fig. 2A and B). The reason to account for this reduction is unknown, but it may not link to mice’ memory effects in the bright light as this observation was not observed in male mice. The total time in light (149.0 ± 24.82 s) was also significantly reduced by UMB when compared to its self-control (512.9 ± 49.80 s, *P =* 0.0002, *n =* 13, Fig. 2A and B). We further compared relative values (normalized to self-control) to eliminate confounding factor due to mice’s memory effects in the bright light. We observed that female mice showed a significant reduction (*P =* 0.0155, Fig. 2C) of total time spent in light when compared to the vehicle control (0.33 ± 0.064, UMB group *vs* 0.63 ± 0.080 in vehicle control). These data suggest the photophobia model’s validity in females, analogous to the effects observed in males, although the observed effect in female mice was not as pronounced as in males.

**Fig. 3:**
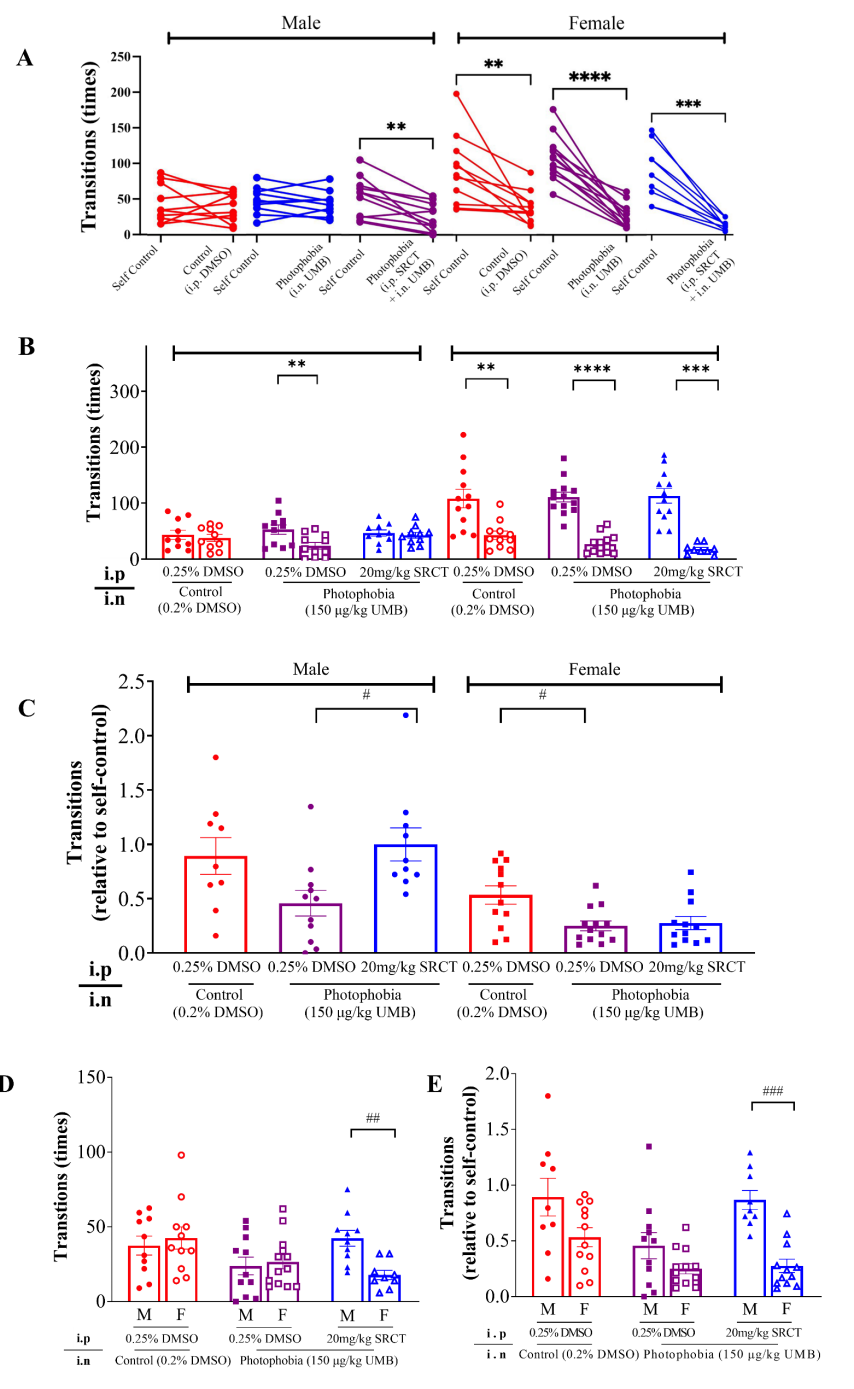
SFKs antagonism by saracatinib reduced transition times between the light and dark compartments in light in male C57BL/6J photophobia mice induced by intranasal injection of UMB. Transition times data was collected as the same as total time in light from the same mice shown in Fig. 2. (**A**, **B**) Transition (times) between the light and dark compartments before and after light aversion induction. (**C**) Transition times recorded after light aversion induction relative to self-control. (**D**) Comparison of transition times recorded after light aversion induction between male (M) and female (F) mice. (**E**) Comparison of transition times recorded after light aversion induction relative to self-control between male and female mice. Data are shown as mean ± SEM. Significant difference was shown by * *P <* 0.05, ** *P <* 0.01, *** *P <* 0.001, or **** *P <* 0.0001; ^#^*P <* 0.05, ^##^*P <* 0.01. *Indicates comparison between dependent groups. ^#^ Indicates comparison between independent groups

In order to better understand the UMB-induced photophobia-like behavior, we also analysed transition times of mice between the light and dark compartments of the chamber. In male mice, there was no alteration of transition times in the vehicle group (37.50 ± 6.330) compared to the self-control (43.40 ± 8.394, *P =* 0.5177, *n =* 10, Fig. 3A and B). Differently, the transition times was significantly lower (*P =* 0.0022, Fig. 3A and B) in the UMB group (23.86 ± 5.932, *n =* 11) compared to its self-control (52.50 ± 8.422). In compatible, a clear trend of reduction in transition times normalised to the self-control (*P =* 0.0708, Fig. 3C) was observed in the UMB group (0.4574 ± 0.1173, *n* = 11) compared to that in the control group (0.8931 ± 0.1690, *n* = 9). In female mice, significant reduction of transition times normalised to self-control (*P =* 0.018, Fig. 3C) was observed in the UMB group when compared to that in the vehicle group. It was noted that there was also a significant reduction of transition times by the vehicle (*P =* 0.0064) compared to that of self-control (Fig. 3A and B).

Females spent transition times than males even after the vehicle administration. It is possible that females are typically more susceptible to external stimuli compared to their male counterparts. Whether mice’ memory effect affecting the second behavior recording only in females requires further investigation as similar results were not observed in male mice. When the male and female photophobia mice were compared, there was no gender-different difference in the total time in light (M: 248.2 ± 53.85 s, *n* = 11; F: 149.0 ± 24.8 s, *n* = 13; *P =* 0.3031, Fig. 2D) or those normalised to the self-control (M: 0.4982 ± 0.09587; F: 0.3256 ± 0.06432; *P =* 0.1522, Fig. 2E). Nor there was significant difference in transitions times of male and female photophobia mice (M: 23.86 ± 5.932; F: 26.62 ± 4.720; *P =* 0.7204, Fig. 3D), or those normalised to the self-control (M: 0.4574 ± 0.1173; F: 0.2500 ± 0.04524; *P =* 0.1231, Fig. 3E). Taken all these together, the reduced total time in light and decreased transition times in male mice, to a lesser extent, in female mice by intranasal injection of UMB demonstrate successful provocation of light aversion behavior, which provides a rapid onset and injury-free mouse model of photophobia for subsequent investigation of drug effect.

Using this novel photophobia model, we investigated the effects of the SFKs inhibitor, SRCT on light aversion in mice that were intraperitoneally administered with SRCT (20 mg/kg) or its vehicle (0.25% DMSO) for consecutive 2 days prior to photophobia induction. In male photophobia mice, the total time in light was significantly higher (*P* = 0.0075, Fig. 2B) in the SRCT group (546.7 ± 72.11 s) than that in the vehicle group (248.2 ± 53.85 s). Higher total time in light normalised to self-control (*P =* 0.0005, Fig. 2C) was similarly observed in SRCT group (1.31 ± 0.20) than that in the vehicle group (0.50 ± 0.10), suggesting male photophobia mice was sensitive to SFKs antagonism. Differently, there was no alternation of the total time in light (relative to self-control) in female photophobia mice that were treated with SRCT compared to that in the vehicle group (Fig. 2C). There were significant differences in total time in light presented in both absolute and relative values between the two sexes, suggesting male photophobia mice, but not female, was sensitive to SFKs antagonism (Fig. 2D and E). Likewise, the transition times was reversed by SRCT only in male photophobia mice, but not in female (Fig. 3B and C) and this parameter was significantly higher (*P =* 0.0001, Fig. 3F) in male (M: 0.8680 ± 0.08493) than that in female (0.2765 ± 0.06048). These data support that the effects of SRCT on reducing light aversion only in male mice.

### The effect of saracatinib on light aversion is not associated with hypothalamic CGRP and PACAP levels

**Fig. 4:**
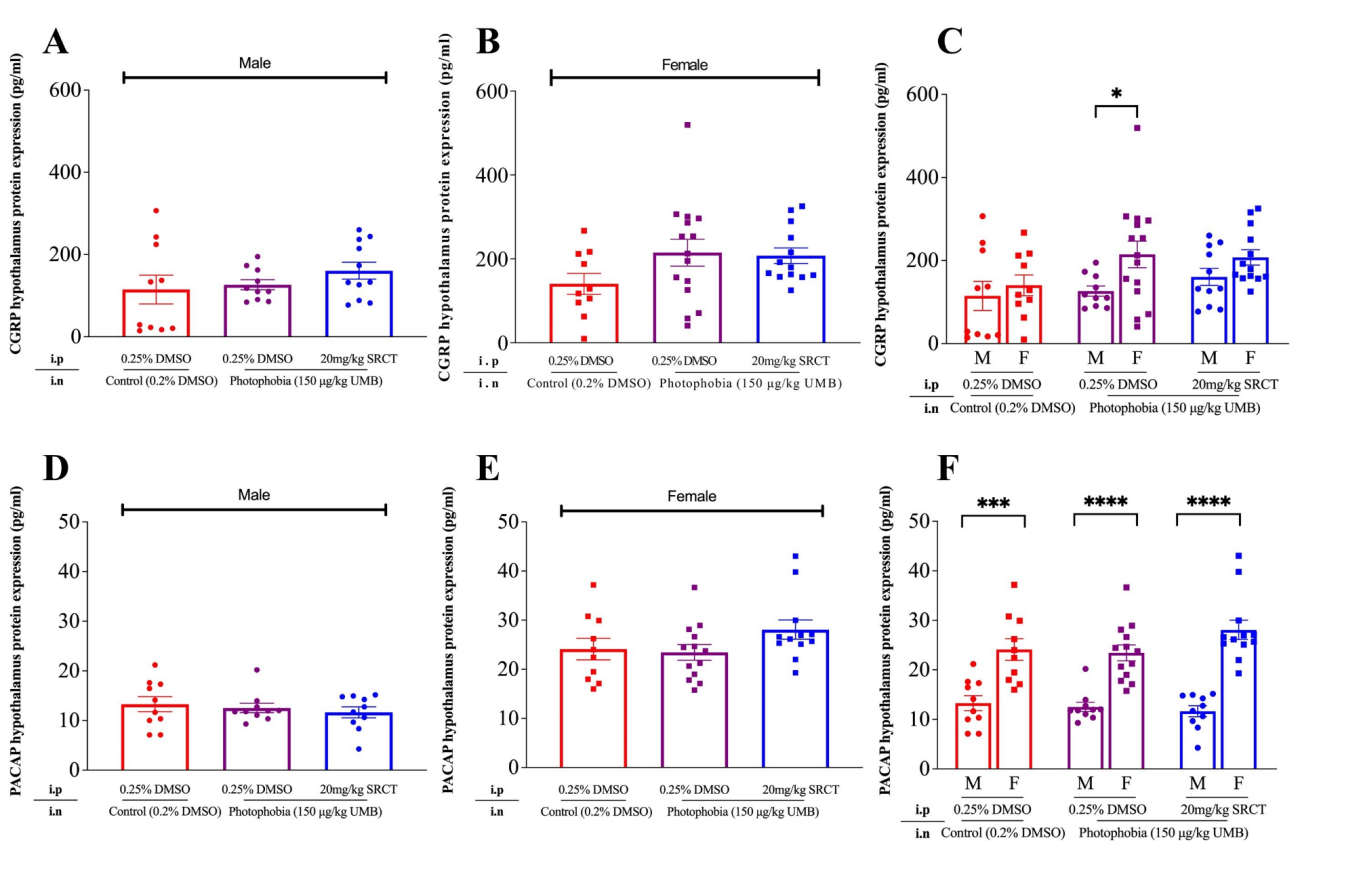
SFKs antagonism did not alter CGRP and PACAP levels in the hypothalamus of C57BL/6J photophobia mice. The hypothalamus of the mouse in the control, photophobia, and photophobia with SRCT groups in both male and female mice was collected from the same mouse cohort as the light aversion behavior shown in Fig. 2. (**A**, **B**) Levels of hypothalamus CGRP protein expression (pg/ml) in male and female mice respectively. (**C**) Comparison of hypothalamus CGRP protein expression (pg/ml) between male and female mice. (**D**, **E**) Levels of hypothalamus PACAP protein expression (pg/ml) in male and female mice respectively. (**F**) Comparison of hypothalamus PACAP protein expression (pg/ml) between male and female mice. Data were presented as mean ± SEM. One-way ANOVA was used for comparison among the three groups and two-tailed unpaired t-test was used for comparison between male and female group. Significance differences were indicated as * *P <* 0.05, *** *P <* 0.001, or **** *P <* 0.0001

CGRP and PACAP are widely known for inducing light aversion via independent convergent pathways in CD-1 mice [7]. We explored the impact of SFKs antagonism by SRCT on hypothalamic CGRP and/or PACAP levels using the hypothalamus tissue dissected immediately after the behavior observation. Both CGRP and PACAP were detected in the hypothalamus of all mice investigated (Fig. 4). In the control group, the basal level of CGRP and PACAP in male mice was 114.9 ± 35.05 pg/ml (Fig. 4A) and 13.29 ± 1.514 pg/ml respectively (Fig. 4D). Female mice had similar basal CGRP levels as in male (Fig. 4B and C); Whilst PACAP level was 1.81-fold higher (*P =* 0.0009, Fig. 4E and F) in female (24.11 ± 2.186 pg/ml) than that in male (13.29 ± 1.514 pg/ml). Neither CGRP nor PACAP level was altered after intranasal injection of UMB in the absence or presence of SRCT in both female and male mice (Fig. 4A, B, D and E). Notably, when comparing the CGRP or PACAP levels between the two sexes, female photophobia mice exhibited overall higher levels of these neuropeptides, particularly with PACAP (Fig. 4C and F), which is SFKs-independent.

### Photophobia significantly altered transcriptome of the trigeminal ganglion in male mice with some changes being SRCT-sensitive

**Fig. 5:**
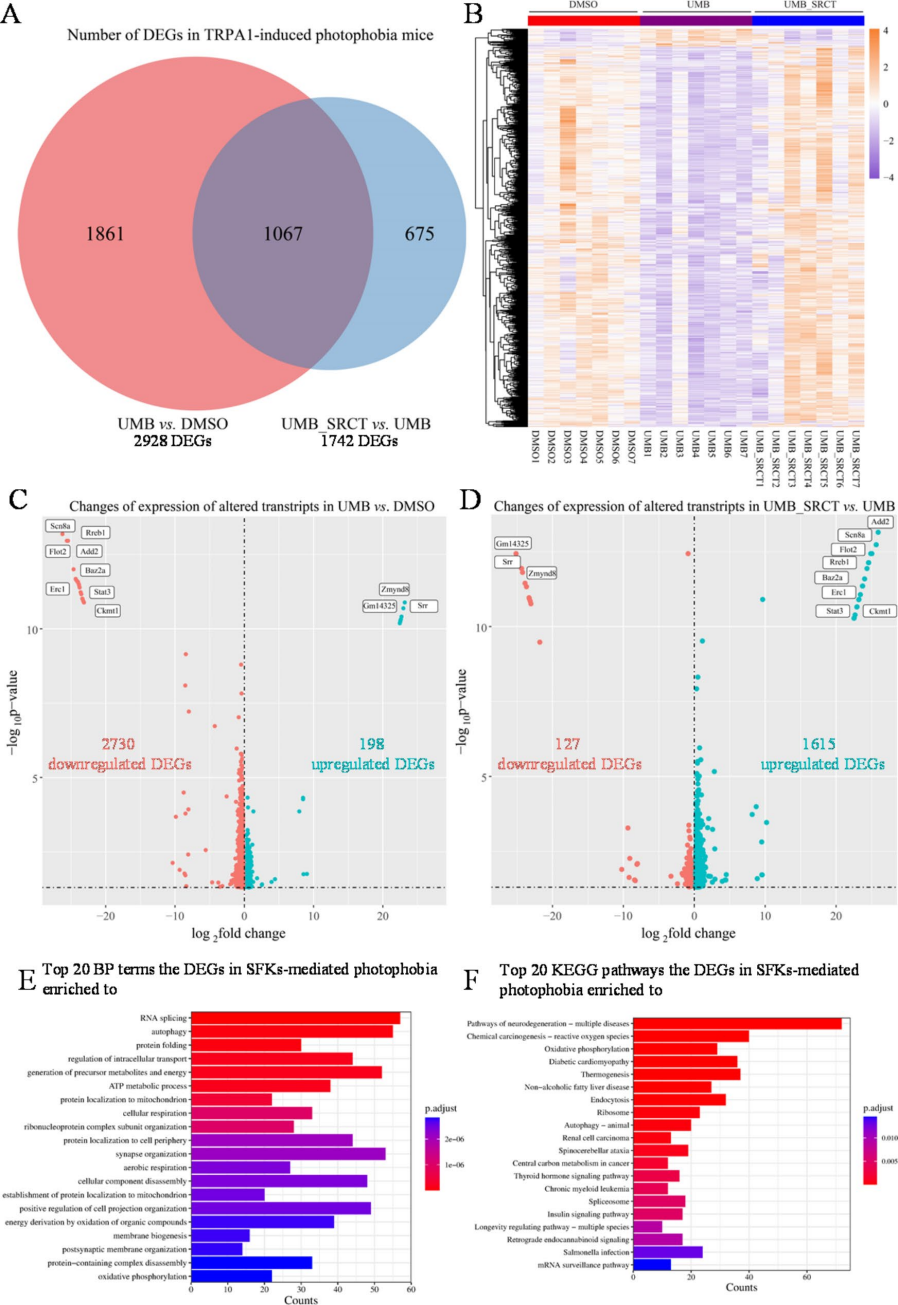
RNA-sequencing and enrichment analysis revealed profound alternation of transcriptome of TG in male photophobia mice sensitive to pre-treatment of SRCT. The merged TG of each mouse was collected from the same male mouse cohort as the light aversion behavior shown in Fig. 2. (**A**) Venn diagram visualized numbers of differentially expression genes (DEGs, adjusted *P*-value < 0.05) identified from UMB vs. DMSO and UMB_SRCT vs. UMB groups. The overlapped region showed the number of DEGs that are sensitive to SRCT. (**B**) Heatmap showed the expression level of 1067 DEGs in each sample of the three experimental groups. 95.5% of these DEGs were downregulated by UMB, of which 98.0% were restored by pre-treatment of SRCT. (**C**,** D**) The volcano plot depicted the numbers of DEGs (|log2FoldChange|≥0, adjusted *P-*value ≤ 0.05) between UMB vs. DMSO and UMB_SRCT vs. UMB. Each dot represents a DEG, and red or blue dots indicate down- or up- regulation, respectively; The transcripts with |log2FoldChange|>20 in both two comparisons were labelled by the names of genes they transcript from. (**E**) Gene ontology enrichment analysis and the top 20 terms in the categories of biological process (BP) for the SFKs-sensitive DEGs in male photophobia mice (adjusted *P*-value < 0.05) and (**F**) KEGG pathway analysis and the top 20 pathways for the SFKs-sensitive DEGs in male photophobia mice. The count represents numbers of DEGs enriched to respective top 20 functions or pathways

We next tested the hypothesis that the light aversion and the reduction of this behaviour due to SFKs antagonism in male mice are associated with TG activation. Following RNA sequencing analysis of the TG immediately dissected post-behaviour observation, a general examination of gene expression profile revealed 2928 DEGs in the photophobia mice compared to the control (Fig. 5A and B; S1 Table). Among these, 2730 genes were downregulated and 198 genes upregulated (Fig. 5C). In photophobia mice pretreated with SRCT for consecutive two days, a total of 1742 genes in the photophobia mouse TG were differentially expressed compared to the photophobia only group (Fig. 5A; S2 Table). Among these, 127 genes were downregulated and 1615 genes were upregulated (Fig. 5D). Notably, of the 1742 genes identified, 1067 genes in the TG were sensitive to SFKs antagonism in these photophobia mice (Fig. 5A). It is noteworthy that 95.5% of the 1067 DEGs were downregulated in the photophobia group, of which 98.0% were restored by pre-treatment of SRCT as illustrated in the heat map analysis (Fig. 5B), suggesting profound transcriptomic alternation.

We further conducted pathway enrichment analysis using clusterProfiler R package. The results showed that 1067 SRCT-sensitive DEGs were enriched in 481 Gene Ontology (GO) terms in biological process and 37 in Kyoto Encyclopedia of Genes and Genomes (KEGG) pathways. The top 3 most significant biological process (BP) of GO terms were enriched in RNA splicing, autophagy, intracellular transport, ATP metabolism and mitochondrial (Fig. 5E) pathways, and those in the KEGG database pathways enriched to neurodegeneration diseases, reactive oxygen species and oxidative phosphorylation (Fig. 5F).

**Fig. 6:**
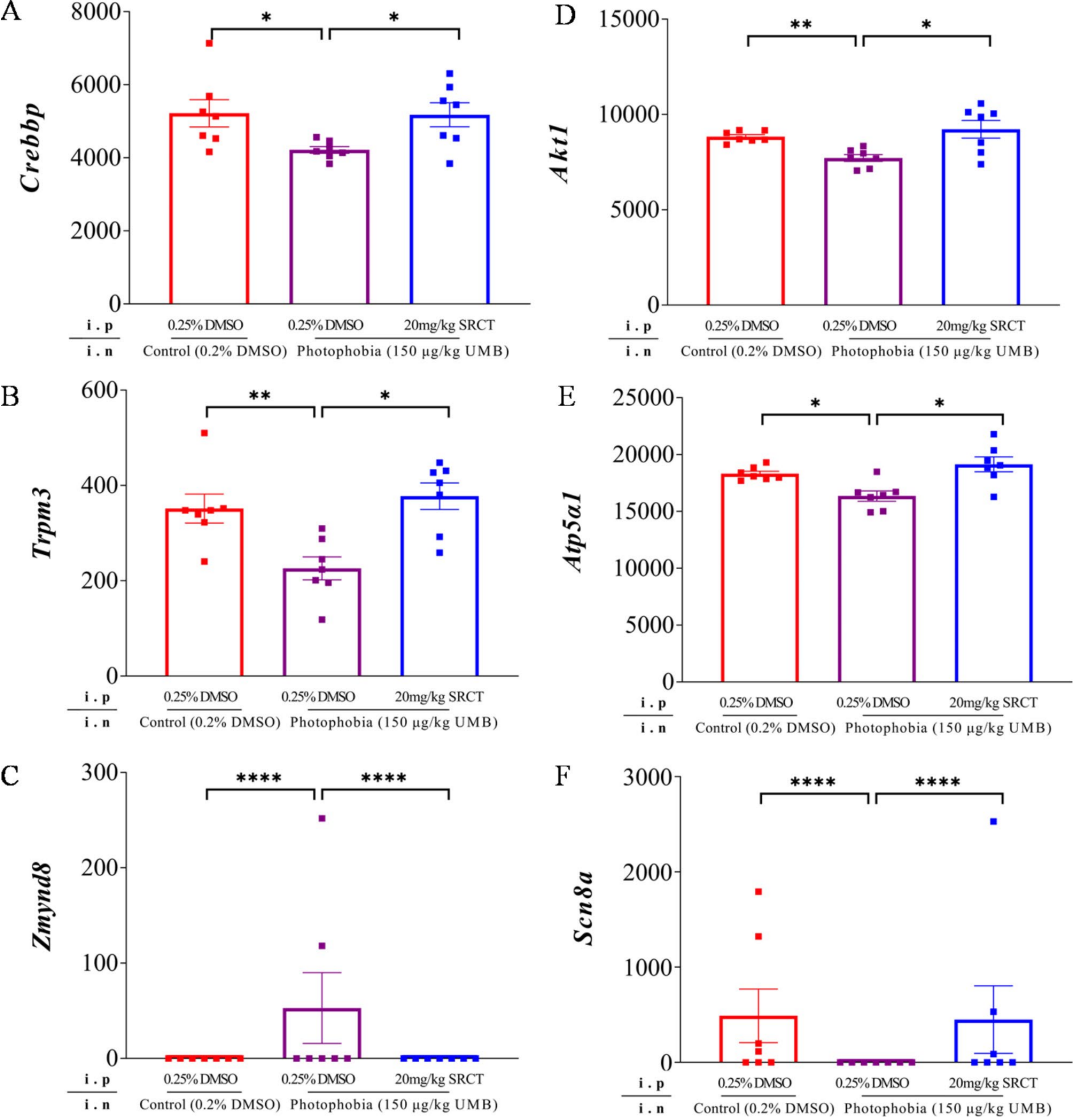
Differential gene expression of selected candidate genes of TG in male mice in control, photophobia, and photophobia with SRCT groups. The merged TG of each mouse was collected from the same male mouse cohort as the light aversion behavior shown in Fig. 2. Gene level counts were calculated by DEseq2 package and its functions. Normalized counts were compared between control (*n =* 7, red) vs. photophobia (*n =* 7, purple) groups and photophobia vs. photophoibia + SRCT (*n =* 7, blue) groups using the Wald test for significance. Changes in gene expression of (**A**) *Crebbp*, (**B**) *Trpm3*, (**C**) *Zmynd8*, (**D**) *Akt1*, (**E**) *Atp5a1* and (**F**) *Scn8a* were presented in respective order. Mouse number and sex are indicated. Group data were presented as mean ± SEM. Significant differences were indicated by * *P <* 0.05, ** *P <* 0.01, *** *P <* 0.001, or **** *P <* 0.0001

In order to identify hub genes that are pivotal for photophobia, we filtered 1067 SRCT-sensitive DEGs with an absolute value of log2FoldChange that are greater than 20 for further analysis. Total 33 DEGs induced by photophobia were identified (S3 Table), of which expression of 32 of these transcripts were reversed by SRCT (S4 Table). We then compared these genes with those known migraine-related genes reported earlier from RNA-sequencing on the TG of PACAP-induced photophobia mice, RNA-seq of migraineurs post-mortem TG and a genome-wide association study of 102,084 migraine cases [7, 31, 32]. We found expression induction of several migraine-related genes (*Crebbp*, *Trpm3*,* Atp5α1* and *Akt1)* were downregulated by photophobia, notably this reduction was restored by SRCT in our study (Fig. 6A, B, D and E). Notably, *Scn8a* that encodes a sodium voltage-gated channel, was also downregulated by photophobia with the highest fold change; Conversely, gene expression of *Zmynd8*, a risk locus in migraineurs [31], was markedly upregulated by photophobia, both of *Scn8a and Zmynd8* DEGs were restored by the SFKs inhibitor (Fig. 6C and F). These data support that male photophobia mice are associates with profound activation of TG, some of which are reversed by SFKs antagonism.

### SFKs antagonism displayed gender-different expression modulation of the candidate genes of TG in the photophobia mice

**Fig. 7:**
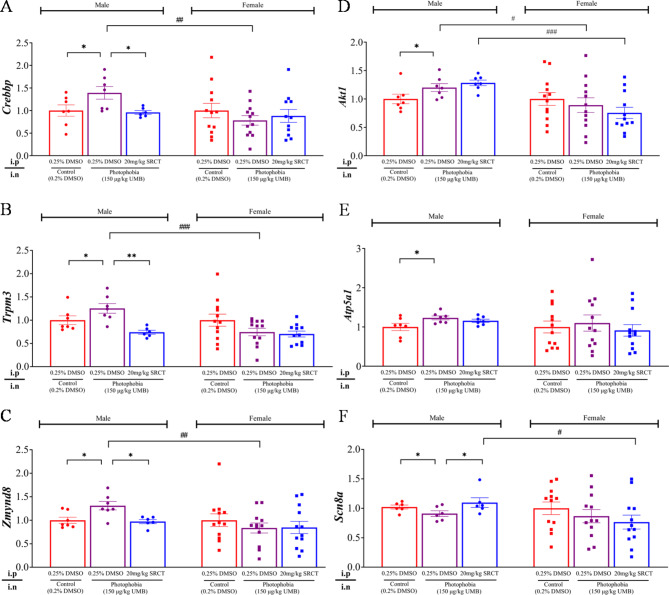
The photophobia-induced expression of the selected candidate genes of TG showed gender-different modulation by SFKs antagonism assessed by qPCR. The merged TG of each mouse was collected from the same male and female mouse cohort as the light aversion behavior shown in Fig. 2. The relative gene expression levels in control (red, male *n =* 6 or 7, female *n =* 12), photophobia (purple, male *n =* 6 or 7, female *n =* 12) and photophobia + SRCT (blue, male *n =* 6 or 7, female *n =* 11 or 12) groups were quantified using qPCR and presented by the fold changes normalized to the geometric mean of peptidylprolyl isomerase A (PPIA) and β-actin *(ACTB)*, and expressions were determined using 2^−ΔCT^ method. Changes in gene expression level of (**A**) *Crebbp*, (**B**) *Trpm3*, (**C**) *Zmynd8*, (**D**) *Akt1*, (**E**) *Atp5a1* and (**F**) *Scn8a* between control vs. photophobia groups, photophobia vs. photophoibia + SRCT groups, and male vs. female were presented in respective order. Either Mann-Whitney test (abnormal distributed data) or unpaired t-test (normally distributed data), one-tailed test was used for significance. Group data were presented as mean ± SEM. Significant differences were indicated by * *P* < 0.05, ** *P* < 0.01, *** *P* < 0.001, or **** *P* < 0.0001. # *P* < 0.05, ## *P* < 0.01, ### *P* < 0.001, #### *P* < 0.0001. * Comparisons within the same gender and # comparisons between male and female

In order to validate RNA-sequencing data of these 6 selected genes (*Crebbp*, *Trpm3*,* Zmynd8*,* Akt1*,* Atp5α1* and *Scn8a*) in male mice and to identify the candidate genes in the TG that may link with the SRCT-exhibited gender different action on photophobia, we compared mRNA levels of the above 6 DEGs of TG between the male and female mice using qPCR. We noted that there were inconsistent results between RNA-sequencing and qPCR analysis in male mice for 4 (*Crebbp*,* Trpm3*,* Akt1*,* Atp5α1)* of the 6 genes (Figs. 6 and 7). This difference is likely due to different amplification efficiency with individual gene features (e.g., transcript length, number of exons and read quality) between the two methods and that the transcript used for qPCR assays was only one of many transcriptomes for above non-concordant genes thus reads mapping to shared exons from transcripts cannot be detected by qPCR assay. It was reported that the non-concordant genes were mostly borderline, and over 66% of all genes in the non-concordant fraction have a Δfold change (FC) < 1 and 93% have a ∆FC < 2 in an independent benchmarking study [33]. In our current study, *Crebbp*,* Trpm3*,* Akt1*, and *Atp5a1* are non-concordant genes, all of which has a ΔFC < 1. In contrast, this is not the case for genes (*Zmynd8* and *Scn8a*) with ΔFC > 20 and consistent data were seen between the two methods (Figs. 6 and 7).

Our qPCR data showed that, in the male mouse TG, gene expression of *Crebbp*, *Trpm3*,* Zmynd8*,* Akt1 and Atp5α1* (Fig. 7A-E) were upregulated, whilst *Scn8a* (Fig. 7F) was downregulated in the photophobia group. Such changes in gene expression of *Crebbp*, *Trpm3*,* Zmynd8* and *Scn8a* were all reversed by the SFKs inhibitor, except that of *Akt1 and Atp5α1* (Fig. 7D and E). In contrast to male mice, none of the mRNA levels of these 6 genes was altered by photophobia in the absence or presence of SRCT pretreatment in the TG of female mice (Fig. 7). These data suggest female photophobia mice showed different gene expression modulation by SRCT from that of the male mice. When mRNA levels of *Crebbp*, *Trpm3*,* Zmynd8* and *Akt1* were compared between the two sexes, female photophobia mice showed significant lower levels than that of males (Fig. 7A-D). In contrast, there was no difference in the induction of gene expression of *Atp5α1 and Scn8a* (Fig. 7E and F).

## Discussion

In this study, we report that SFKs antagonism by SRCT reduced photophobia behavior in male mice and exhibited distinct gender-different modulation of candidate gene expression in trigeminal ganglia, underscoring SFKs antagonism potential for precision medicine in photophobia therapy.

We initially established an injury-free, rapid-onset photophobia model in C57BL/6J mice by intranasal injection of UMB for studying the impact of SFKs antagonism on light aversion and the underlying mechanism. The volatile feature of the TRPA1 activator enabled the drug being administered through the vascular-rich upper nasal space, where it can be rapidly absorbed to trigger mouse light avoidance as early as 15 min after the administration. This injury-free model is an important addition to the existing photophobia surrogates such as minimally invasive light aversion models induced by a single *i.p.* CGRP injection [16]. Also, it minimizes any stress-induced variability in experimental outcomes comparing with those existing models requiring intracerebroventricular [19] or repeated *i.p.* injections [9] of chemical triggers that introduce unavoidable element of physical discomfort and stress. This aspect is particularly important in studies examining gender different effect of antimigraine drugs, given the varying sensitivities to stress across genders. Furthermore, the UMB-induced light aversion is not significantly linked to sex, albeit female mice had light aversive behavior, to a lesser extent, than males. The data align with that CGRP-induced light aversion without showing gender differences, although females tend to spend less time in the light [17] and PACAP-38-induced light aversion behavior, where female mice have a higher percentage of nonresponders to PACAP-38 [7]. The fact that UMB is capable of inducing light aversion offers new insight into the critical role of TRPA1 in migraine pathogenesis. These data are compatible with the ability of UMB to induce cutaneous allodynia in mice [26] and promote CGRP and interleukin 1β mRNA level in TG of mice by transmitting signals to SFKs [15]. We suspect that the UMB-triggered light aversion may be attributed to its ability to induce rapid signal transduction, neurogenic inflammation, and vasodilatation in the central and trigeminovascular system [15, 21, 34], but this awaits further investigation.

Using the novel photophobia surrogate, we found SRCT reduced light aversion behavior only in male mice. This data is in line with our earlier findings using male rodents that SFKs antagonism reduces cortical spreading depression propagation [12, 15] and TG sensitization by reducing SFKs activity at the Y416 phosphorylation site in various migraine models [13, 15]. The male-, but not female- decline, under SFKs antagonism may imply that female migraine sufferers is generally less responsive to drug treatment. This, in fact, corresponds with the higher prevalence of the condition in females compared to males. Additionally, SFKs serve as a convergent hub by transmitting signaling of NR2A-containing receptor [10] and purinergic P2X receptor 7 in cortical spreading depression migraine model [12] and that of TRPA1 and CGRP signaling in ex vivo models of migraine [15]. SRCT has undergone clinical trails for treating various cancer [35, 36] and Alzheimer’s disease [37, 38], exhibiting satisfactory tolerability of safety in patients. Therefore, SFKs antagonism could potentially serve as an effective therapy for male individuals suffering from photophobia and migraine.

To reveal mechanisms underlying the SFKs antagonism-displayed gender different reduction of light aversion in mice, we examined hypothalamus levels of CGRP and PACAP, the known neuropeptides for inducing photophobia and migraine [7, 39]. In our study, the lack of association between light aversion in both male and females and these two neuropeptides do not support the involvement of hypothalamic CGRP or PACAP in the acute photophobia, regardless of the presence or absence of SFKs antagonism. Notwithstanding, The higher levels of hypothalamic neuropeptides in female mice compared to males across all experimental groups suggest that these neuropeptides may be linked to the predominance of migraine in females, which warrants further research. It is possible that the initiation of the acute light aversion may primarily involve trigeminovascular pathways rather than central pathways.

In order to explore the involvement of TG activation and to comprehend the genetic basis driving the male-only effect of SFKs antagonism on light aversion, we conducted RNA-sequencing analysis of TG in male mice. As expected, a profound regulation of the transcriptome of TG is linked with the mouse photophobia behavior, of which 1067 SRCT-sensitive differentially expressed genes were identified. These DEGs are largely enriched in signal transduction, energy metabolism and post-transcriptional modification, including those genes encoding ion channels (*Trpm3*,* Scn8a)*, ATPase signaling *(Crebbp* and *Atp5a1)* and kinase receptors *(Zmynd8* and *Akt1)*. Based on our own RNA-sequencing data, genes previously known to link with migraine through genome-wide analysis [31] or various migraine models [40–47], *Crebbp*, *Trpm3*,* Zmynd8* and *Akt1* are among the potential candidate genes that have especially caught our interest. Notably, *Zmynd8*, encoding the receptor of protein kinase C [44], is a risk locus in migraineurs [31]; However, knowledge on *Zmynd8* linking with SFKs in migraine is sparse, albeit previous findings have shown that Src phosphorylation is critical for protein kinase C-mediated sensitization of transient receptor potential vanilloid-1 in vitro [48] and reciprocal interaction between protein kinase C and SFKs can upregulate the action potential firing activity in hypothalamic arcuate nucleus neurons [49]. Future studies on understanding the role of *Zmynd8* in photophobia and the mechanism underlying its relation with SFKs may prove to be important. We also identified two novel genes, *Scn8a* and *Atp5α1* that have not been reported previously in models of migraine. It worth noting that, *Scn8a* exhibit a pronounced gene expression change following photophobia and SFKs antagonism (> 10). *Scn8a* is associated with treatable epilepsy [50] and is a variant of *Scn1a*, which intensifies trigeminal nociception in Familial Hemiplegic Migraine type 3 [51]. *Scn1a* encodes Nav1.1, a neuronal voltage-gated Na^+^ channel and the activity of Nav1.1 channel can be reduced by SFKs inhibitors in cultured rat spiral ganglion neurons [52]. Therefore, we postulate that *Scn8a* may have similar function to play a significant role in the SFKs-mediated light aversion. Future research probing the role of *Scn8a* in mediating neuronal excitability in response to migraine triggers could provide significant insights into SFKs-mediated mechanism of photophobia.

When gene expression was statistically compared between the two sexes using qPCR, all the 6 genes exhibited gender-different modulation and are prominent in male mice, but not in females. Both the induction of gene expression of *Crebbp*,* Trpm3*,* Zmynd8* and the reduction of gene expression of *Scn8a* by photophobia was reversed by SFKs antagonism in males, but such alteration was not observed in females. This gender-different modulation pattern in mice in fact aligns with reduction of photophobia behavior by SFKs antagonism only in male mice. Among these genes, *Crebbp*,* Trpm3*,* Zmynd8*,* Scn8a* have 22, 76, 13 and 36 ECRs in respective order with a conservation criterion of ≥ 350 bp ≥ 77% ID [53] based on the human/rodent conservation profiles using the evolutionary conserved element (ECR) browser [54].Therefore,*Trpm3* is more conserved and likely to determine the observed SFKs-mediated light aversion behavior. *Trpm3-*encoding protein transient receptor potential melastatin-3 (TRPM3) has been reported to play an important role in neuroinflammation [55] and promote CGRP release from sensory nerves [56]. TRPM3 induces an intracellular signaling cascade involving a rise in intracellular *Zmynd8*, the risk locus in migraineurs [31]. It would be interesting to explore the role of *Trpm3* and *Zmynd8* in photophobia and the mechanism underlying their relation with SFKs.

Our study has limitations. Bright light was applied twice in day 2 for inducing the UMB-induced light aversion. Mice’s memory of the light/dark areas could mask their light aversion behavior under study as light aversion behavior induced by CGRP varies depending on strong light exposure time [17]. Although this confounding factor was minimized through thorough analysis using relative values and that male mice does not show photophobia behavior in the vehicle control, future investigation is necessary to clarify this following single light exposure in a day. Also, future investigation on the effects of anti-migraine drugs like sumatriptan or CGRP antagonists in our models would be intriguing.

## Conclusions

In conclusion, we developed a novel rapid-onset and injury-free light aversion model. Our data revealed that SFKs antagonism alleviates photophobia in male mice, underscoring its potential for precision medicine in photophobia therapy. The observed gender-different responses are mirrored in the selective modulation of gene expression within the trigeminal ganglion, including several intriguing candidate genes *Trpm3*,* Zmynd8*, and *Scn8a*, pointing to the significant role of trigeminal ganglion activation in photophobia processing. These findings pave the way for future research into gender-different mechanisms and treatments for photophobia. Future work is necessary to investigate how respective genes and their encoding proteins function in mediating light aversion behavior.

## Electronic supplementary material

Below is the link to the electronic supplementary material.

Supplementary Material 1: S1 Table. RNA-sequencing analysis shows gene expression profile of TG in male mice in photophobia (UMB) vs. control (DMSO) groups.

Supplementary Material 2: S2 Table. RNA-sequencing analysis shows gene expression profile of TG in male photophobia mice in the absence or presence of SRCT.

Supplementary Material 3: S3 Table. Gene expression of 33 candidate genes of TG (criteria: absolute value of log2FoldChange greater than 20) in male photophobia (UMB) vs. control (DMSO) groups.

Supplementary Material 4: S4 table. Gene expression of 32 genes of TG (criteria: absolute value of log2FoldChange greater than 20) in male photophobia mice in the presence or absence of pretreatment of SRCT groups.

Supplementary Material 5: S5 Table. Statistic data for Figs. 2, 3, 4 and 6–7

## Data Availability

No datasets were generated or analysed during the current study.

## Abbreviations

CGRP: Calcitonin gene-related proteins
DEGs: Differentially expressed genes
DNBs: DNA nanoballs
DMSO: Dimethyl sulfoxide
GO: Gene Ontology
i.n: intranasal
i.p.: Intraperitoneal
KEGG: Kyoto Encyclopedia of Genes and Genomes
PACAP: Pituitary adenylate cyclase-activating polypeptide
PPIA: Peptidylprolyl isomerase A
qPCR: Quantitative Polymerase Chain Reaction
SFKs: Src family kinases
SRCT: Saracatinib
TG: Trigeminal ganglion
TRPA1: Transient receptor potential ankyrin type 1
TRPM3: The transient receptor potential melastatin-3
UMB: Umbellulone
