# Supplementary material for: Gender-different effect of Src family kinases antagonism on photophobia and trigeminal ganglion activity
Source: J Headache Pain. 2024 Oct 11;25(1):175. doi: 10.1186/s10194-024-01875-3 (PMC11468534; doi:10.1186/s10194-024-01875-3)
Supplement: Supplementary file 1 — Supplementary Material 1: S1 Table. RNA-sequencing analysis shows gene expression profile of TG in male mice in photophobia (UMB) vs. control (DMSO) groups. [file 10194_2024_1875_MOESM1_ESM.pdf]

Supporting Table 1. RNA-sequencing analysis shows gene expression profile of TG in male mice in photophobia (UMB) vs. control (DMSO) groups.

GeneID	baseMean	log2FoldChange	lfcSE	stat	pvalue	padj	length
Add2	290.9506269	25.93317119	2.95494577	8.776191922	1.69E-18	7.16E-14	2289
Scn8a	233.6444727	25.65525574	2.986734267	8.589734958	8.72E-18	1.85E-13	7120
Ube2i	109.686074	-0.880480515	0.104035259	-8.463289488	2.60E-17	3.67E-13	1144
Rpl22	145.3683636	25.04916804	2.986798645	8.386627628	5.00E-17	3.67E-13	2051
Repin1	95.45922301	-25.13464925	2.986882666	-8.415010586	3.93E-17	3.67E-13	3145
Baz2a	130.0568146	24.89273898	2.986818705	8.334198169	7.80E-17	3.67E-13	8385
Flot2	139.9584275	24.99666758	2.986805232	8.369031672	5.81E-17	3.67E-13	2699
Flot1	138.2100407	24.9722443	2.986807471	8.360848346	6.23E-17	3.67E-13	1383
Kif1a	194.638917	24.57217222	2.986755569	8.227044914	1.92E-16	7.39E-13	5391
Ckmt1	105.6162621	24.60003704	2.986862771	8.236078764	1.78E-16	7.39E-13	851
Synm	88.83101444	24.3615825	2.986907081	8.156123319	3.46E-16	1.16E-12	2697
Nfat5	54.04328904	-24.35256291	2.987076245	-8.152641884	3.56E-16	1.16E-12	5899
Snx14	48.46336325	-24.21678225	2.987127618	-8.107046417	5.19E-16	1.57E-12	3112
Spats2l	69.252571	24.00999092	2.986985901	8.038200285	9.12E-16	2.57E-12	2315
Kat7	37.47141806	-23.86662314	2.987273546	-7.989433433	1.36E-15	3.59E-12	3324
Erc1	56.4164708	23.72314507	2.98706727	7.941952064	1.99E-15	4.43E-12	8356
Stat3	139.7767755	23.73691542	2.986805524	7.947258443	1.91E-15	4.43E-12	2506
Rreb1	95.29432381	23.74977049	2.986888217	7.951342256	1.85E-15	4.43E-12	8395
Zfp869	32.5601884	-23.67036576	2.987370616	-7.923478138	2.31E-15	4.70E-12	3199
Fam13c	32.52910613	-23.66699811	2.987371316	-7.922348983	2.33E-15	4.70E-12	3262
Gtf2ird1	45.44948756	23.42319216	2.987173187	7.841256831	4.46E-15	8.58E-12	3487
Pja2	27.151529	-23.30319568	2.987518101	-7.800185601	6.18E-15	1.09E-11	4531
Gm14325	23.824918	-23.25304983	2.987642108	-7.78307742	7.08E-15	1.20E-11	1494
Fubp1	71.54007471	9.649119741	1.241933282	7.769434862	7.88E-15	1.24E-11	2552
Pgap6	39.09506463	23.21692507	2.987261744	7.771975494	7.73E-15	1.24E-11	2235
Katna1	38.24868356	23.18627252	2.987275766	7.761677973	8.38E-15	1.27E-11	1774
Nes	24.65187567	-23.1329672	2.987608056	-7.742972562	9.71E-15	1.42E-11	5958
Zmynd8	25.57768887	-23.04088212	2.987572873	-7.712241038	1.24E-14	1.74E-11	5093
Myo7a	32.11561202	22.94005779	2.987399377	7.678939071	1.60E-14	2.19E-11	6849
Kmt2d	31.69309066	22.92159489	2.98740968	7.672732347	1.68E-14	2.23E-11	19805
Slc37a1	26.30357241	22.66443381	2.987569816	7.586244075	3.29E-14	4.10E-11	2972
Stk3	24.25759133	22.54775073	2.987649151	7.546987477	4.45E-14	5.24E-11	2730
Arrb2	25.50321129	22.53296517	2.987599279	7.542164481	4.62E-14	5.29E-11	1443
Prpf8	2919.558856	1.14798128	0.157082282	7.308152569	2.71E-13	3.02E-10	7249
Srr	24.45045639	-21.78352955	2.987616493	-7.291273696	3.07E-13	3.33E-10	3239
Atp5g3	768.2286773	0.497561512	0.071971682	6.913295561	4.74E-12	4.89E-09	723
Ndufb11	1019.007638	0.32561125	0.048012812	6.781757514	1.19E-11	1.20E-08	908
Usf1	544.0899851	0.760779399	0.124981872	6.087117969	1.15E-09	1.11E-06	2167
Mib1	508.0887277	0.919196225	0.154908829	5.933788492	2.96E-09	2.79E-06	3216
Rps20	956.127479	0.467558823	0.079147091	5.907466924	3.47E-09	3.20E-06	3706
Ddrgk1	738.1360502	0.437019392	0.074283907	5.883096455	4.03E-09	3.63E-06	1238
Pabpn1	266.2067403	2.837641432	0.491577872	5.772516614	7.81E-09	6.89E-06	1030
Srgap3	8230.395231	0.365415597	0.063617021	5.743990972	9.25E-09	7.99E-06	8887
Smyd2	937.9875828	0.325050549	0.056745506	5.72821656	1.01E-08	8.59E-06	1680
Prdm15	305.9609777	0.702175083	0.123123471	5.703015663	1.18E-08	9.77E-06	6194
Bex2	1617.654885	0.499599955	0.088244964	5.661512341	1.50E-08	1.22E-05	903
Vti1b	860.5137377	0.495778706	0.088635612	5.593448215	2.23E-08	1.78E-05	1490
Hdac9	223.9225261	0.62344002	0.113264458	5.504286443	3.71E-08	2.80E-05	1683
Snx16	209.1669763	0.579359325	0.105537407	5.489611126	4.03E-08	2.99E-05	2512
Mrfap1	6316.905675	0.460903367	0.084130281	5.478447978	4.29E-08	3.13E-05	1648
Syne1	844.1707317	0.574209478	0.105151012	5.4608079	4.74E-08	3.40E-05	3894
Dhx9	284.5685406	1.009983373	0.186258705	5.422476074	5.88E-08	4.15E-05	2346
Gtf2f1	1007.802117	0.374544412	0.069269964	5.407024785	6.41E-08	4.45E-05	1717
Rps25	807.695925	0.328021416	0.060755125	5.39907396	6.70E-08	4.50E-05	577
Engase	254.1978712	0.442633207	0.081963293	5.400383416	6.65E-08	4.50E-05	4064
Chchd3	273.381797	0.537898418	0.101038091	5.323719129	1.02E-07	6.52E-05	734
Brd3	1336.467496	0.268616009	0.050695792	5.298585901	1.17E-07	7.37E-05	5272
Smc1a	2092.931396	0.300437207	0.056904518	5.279672278	1.29E-07	8.06E-05	4691
Aip	1529.912567	0.446476636	0.084710527	5.270615713	1.36E-07	8.34E-05	1261
Cdh2	3009.895904	0.269366724	0.05117905	5.263222407	1.42E-07	8.56E-05	4843
Rps27a	828.6523971	0.352126545	0.066951895	5.259396272	1.45E-07	8.60E-05	637
Hnrnpul2	3202.46566	0.21270703	0.040460553	5.257145948	1.46E-07	8.60E-05	5118
Nsun2	1951.089038	0.319020962	0.060920274	5.236696067	1.63E-07	9.48E-05	2834
Lasp1	37.72770289	8.725767985	1.671413329	5.220592556	1.78E-07	0.000102044	686
Mt3	1504.877906	0.488251422	0.094473454	5.16813349	2.36E-07	0.000128343	536
2310061I0	1075.59698	0.514619062	0.099749561	5.159111046	2.48E-07	0.000132979	1293
Efcab14	1293.962839	0.310349498	0.060529372	5.127254504	2.94E-07	0.000153674	2792
Micu2	706.0931343	0.34152631	0.066920213	5.103485097	3.33E-07	0.000172172	2329
Pde4dip	5694.575794	0.488074379	0.095860345	5.091514934	3.55E-07	0.000175835	8264
Fkbp2	564.3488869	0.387837033	0.076100757	5.096362368	3.46E-07	0.000175835	642
Septin3	599.0431042	0.740436365	0.145632823	5.08426845	3.69E-07	0.000177557	2397

Antkmt	314.9107814	0.432145534	0.085166643	5.074117272	3.89E-07	0.000183139	1321
Cox17	166.7884005	0.60166778	0.118753059	5.066545511	4.05E-07	0.000186755	444
Slc29a1	25.66271779	8.16994936	1.612634301	5.06621331	4.06E-07	0.000186755	2216
Rtcb	1900.717739	0.235521447	0.046723378	5.040762409	4.64E-07	0.000208849	2009
Sirt2	723.0014801	0.538056836	0.107374537	5.011028208	5.41E-07	0.000238774	1843
Nucb1	358.5328766	2.042299742	0.408798155	4.995863401	5.86E-07	0.000255663	755
Uqcrh	931.3399575	0.241411918	0.048642357	4.962997987	6.94E-07	0.000296858	547
Golga2	98.01812582	1.013787837	0.204652607	4.953701064	7.28E-07	0.000305239	1010
Chmp5	1146.697299	0.277972103	0.056095129	4.955369716	7.22E-07	0.000305239	1519
Akap8	104.111168	10.19033624	2.069847821	4.923229688	8.51E-07	0.000346559	3731
Phactr1	183.3850024	0.625065557	0.127087041	4.918405156	8.73E-07	0.000351826	707
Med28	152.25562	0.634851614	0.129201129	4.913669243	8.94E-07	0.000356628	734
Ndufa13	1485.345335	0.424096104	0.086337857	4.912052693	9.01E-07	0.000356628	1251
Dnajb11	842.8047958	0.382106754	0.07807619	4.894024073	9.88E-07	0.000380261	1927
Nisch	4665.543445	0.810995935	0.165869741	4.889354329	1.01E-06	0.000381255	5559
Dapk3	772.7107541	0.737645464	0.151162284	4.8798248	1.06E-06	0.000394347	1730
Ppp1r2	596.9100066	0.777124963	0.159456836	4.873575714	1.10E-06	0.000403495	958
Tmod2	547.1143977	1.20344969	0.247060042	4.871081874	1.11E-06	0.0004051	1528
Prelid3b	742.2785162	0.316688891	0.065106251	4.864185608	1.15E-06	0.000415896	1553
Rps23	1164.89466	0.4640188	0.095439408	4.861920328	1.16E-06	0.00041712	937
Lgals4	83.54250216	-0.761699725	0.15700451	-4.85145124	1.23E-06	0.000425338	1384
Unc13a	2386.75021	0.474842053	0.097861583	4.852180354	1.22E-06	0.000425338	5654
Polr2l	539.05502	0.478855028	0.098761395	4.848605369	1.24E-06	0.000427363	1650
Hspa1a	1256.145828	0.876681295	0.18215049	4.812950524	1.49E-06	0.000503726	2967
Rps9	2243.70725	0.382932797	0.079654999	4.807391897	1.53E-06	0.000509374	751
Thy1	803.9270385	1.878308061	0.391159195	4.801901844	1.57E-06	0.000513313	446
Zfp961	60.03604557	-9.358015322	1.952593372	-4.792608362	1.65E-06	0.00052804	2689
D6Wsu163	495.6409339	0.274810474	0.057549157	4.775230194	1.80E-06	0.000567159	2501
Ddx23	1576.482015	0.283470757	0.059465368	4.766989036	1.87E-06	0.00057791	3187
Gnal	550.078409	2.574913908	0.540755358	4.761698371	1.92E-06	0.000588974	2022
Tshz2	3283.725246	0.381693438	0.080260001	4.755711832	1.98E-06	0.000594018	4314
Cdr2	1999.759333	0.325092012	0.068359329	4.755634946	1.98E-06	0.000594018	2525
Syf2	701.1442677	0.291641672	0.061529694	4.739852466	2.14E-06	0.000637691	1369
Ncam1	208.6793791	-0.756174194	0.159769395	-4.732910171	2.21E-06	0.000655288	2525
Hagh	1161.811372	0.390071893	0.08248596	4.728948928	2.26E-06	0.000658985	1191
Sart3	1068.059831	0.294355327	0.062550966	4.705847849	2.53E-06	0.000719805	4447
Anp32b	960.42548	0.246114832	0.052434207	4.693783826	2.68E-06	0.000752001	1716
Ankrd17	534.1992195	0.55529648	0.11838284	4.690683899	2.72E-06	0.000758462	9311
St13	4396.014597	0.266412416	0.056892272	4.68275227	2.83E-06	0.000778181	3346
Thrap3	3266.868564	0.254083122	0.054341712	4.675655466	2.93E-06	0.000795109	4378
U2af2	2473.584043	0.3746217	0.080143572	4.67438237	2.95E-06	0.000795109	2183
Atp5e	659.181268	0.443387976	0.094974828	4.668478848	3.03E-06	0.000813118	421
Cct4	597.950596	0.543053745	0.116411269	4.66495856	3.09E-06	0.00082196	1634
Bex3	814.3914965	0.521222616	0.111869197	4.659214765	3.17E-06	0.000829578	930
Csrnp3	141.0698919	0.647122856	0.138986537	4.656011086	3.22E-06	0.000831735	2958
Nudc	1398.393843	0.339911563	0.073000962	4.656261392	3.22E-06	0.000831735	1316
Spag7	564.962387	0.380740867	0.081793552	4.654900732	3.24E-06	0.000831735	1159
Aarsd1	330.8777641	0.368510908	0.079200267	4.652899811	3.27E-06	0.000834791	1516
Cfdp1	781.5755693	0.340997777	0.073353642	4.648682308	3.34E-06	0.000846938	1178
Sdf4	717.161246	0.692232089	0.149039449	4.644623234	3.41E-06	0.000858619	1654
Sart1	1036.432153	0.400934719	0.08645648	4.637416664	3.53E-06	0.000873498	5091
Crk	2171.457982	0.272078693	0.058717057	4.633724966	3.59E-06	0.000884058	3642
Glrx5	614.177027	0.284133885	0.061435904	4.624883237	3.75E-06	0.000901651	2925
Ankrd9	138.2438176	0.813706108	0.175921476	4.625393818	3.74E-06	0.000901651	1706
Trio	7311.381977	0.287851725	0.062235703	4.62518633	3.74E-06	0.000901651	11495
Gripap1	1675.79985	0.373530373	0.080736104	4.626559313	3.72E-06	0.000901651	3015
Noa1	914.8663457	0.408901197	0.088501078	4.620296235	3.83E-06	0.00091661	2288
Rnf145	2767.981051	0.278136035	0.060362368	4.607772139	4.07E-06	0.000952058	3570
Ankle2	494.439531	0.319336451	0.069413818	4.600473808	4.22E-06	0.000975257	4077
Mrps7	680.7803972	0.319270149	0.069396143	4.60069014	4.21E-06	0.000975257	1962
Cux1	163.8303957	0.723311471	0.157449042	4.59394013	4.35E-06	0.000979549	805
Atp5j2	422.3477153	0.428258219	0.093220894	4.594015345	4.35E-06	0.000979549	540
Hk1	1952.852352	0.843874004	0.183651213	4.594981935	4.33E-06	0.000979549	2916
Snrpd2	561.055223	0.384175826	0.08399366	4.573866954	4.79E-06	0.001061365	1420
Sorbs1	139.9693547	-0.632278847	0.138328563	-4.570848086	4.86E-06	0.001071164	1057
Timm13	961.4271263	0.483651616	0.106131913	4.557079996	5.19E-06	0.00112621	1225
Spop	2143.789073	0.905654089	0.199428247	4.541252816	5.59E-06	0.001207978	3063
Mfsd6	369.1578896	-0.573103352	0.126303597	-4.537506186	5.69E-06	0.00122339	3493
Kmt5a	1081.646951	0.639938634	0.141202195	4.532072848	5.84E-06	0.001248951	1425
Cox5b	2024.088372	0.407364643	0.090047647	4.523878844	6.07E-06	0.001281553	865
Znhit1	346.3386236	0.734784052	0.162438944	4.523447603	6.08E-06	0.001281553	1208
Mrto4	327.6220177	0.272709372	0.060481372	4.508981281	6.51E-06	0.001326542	1221
Cpe	18207.44562	0.305413283	0.067714129	4.510333182	6.47E-06	0.001326542	2117
Ahsa1	2717.331057	0.343864429	0.076223892	4.511242085	6.44E-06	0.001326542	1377

Ssu72	485.5625414	0.497496726	0.110529122	4.501046593	6.76E-06	0.001344108	874
Chd9	674.7742108	0.393302659	0.087376688	4.501231022	6.76E-06	0.001344108	12008
Bcas3	137.903509	0.747157008	0.165906761	4.503475349	6.69E-06	0.001344108	412
Gcsh	831.4892278	0.31592618	0.070223284	4.49888076	6.83E-06	0.001351527	1443
Arl2	784.8876305	0.405831064	0.090249672	4.49675941	6.90E-06	0.001352437	1286
Ip6k2	621.9203736	0.4680551	0.104409069	4.482896995	7.36E-06	0.001423605	1708
Usp47	753.2185876	0.376513376	0.08402648	4.480889546	7.43E-06	0.001424057	4697
Comtd1	112.5084513	0.698042824	0.155761962	4.481471684	7.41E-06	0.001424057	714
Ndufa10	3495.264807	0.371135309	0.082887814	4.477561816	7.55E-06	0.001427058	2020
Rnf216	581.7653654	0.353490219	0.078936149	4.478179176	7.53E-06	0.001427058	3704
Ndufb5	186.0427763	1.014602085	0.226783926	4.473871239	7.68E-06	0.001445472	625
Hspa4l	1147.724742	0.437172063	0.097931804	4.464045857	8.04E-06	0.001478302	2744
Kcnab2	2886.295606	0.681644552	0.152591438	4.46712187	7.93E-06	0.001478302	1325
Pafah1b2	187.4114394	0.867218141	0.194293836	4.463436213	8.07E-06	0.001478302	794
Prdx5	1874.710271	0.462129001	0.103443094	4.467470784	7.91E-06	0.001478302	1262
Hectd4	65.17519643	9.514563255	2.136683998	4.452957603	8.47E-06	0.001539022	3103
Elp3	1997.238864	0.308462441	0.069486288	4.43918434	9.03E-06	0.001620013	2851
Chmp4b	4547.945176	0.408542123	0.092067628	4.437413374	9.10E-06	0.001626503	1616
Nab2	145.9041454	0.906325238	0.204451127	4.432967677	9.29E-06	0.001653441	2311
Serbp1	485.4547753	0.601570268	0.135848865	4.428231838	9.50E-06	0.001669118	1617
Hnrnpab	995.7833349	0.562803602	0.127049458	4.429799316	9.43E-06	0.001669118	1437
Auh	1125.437901	0.33618305	0.07590865	4.428784485	9.48E-06	0.001669118	1320
Tox2	564.4802335	0.588119291	0.132851414	4.426895221	9.56E-06	0.001672302	2947
Pgp	2485.835347	0.407322408	0.092028688	4.426037348	9.60E-06	0.001672302	2627
Rrp1	5745.135901	0.363433044	0.082229898	4.419719044	9.88E-06	0.001694062	1984
Nudt18	216.170952	0.375121869	0.084867834	4.420071204	9.87E-06	0.001694062	3926
Hspa1b	1693.917091	0.966729212	0.218693648	4.4204723	9.85E-06	0.001694062	2803
Camsap3	310.2225493	0.612134775	0.138996067	4.403971898	1.06E-05	0.001775208	4450
Timm44	620.5354704	0.455183541	0.103367622	4.403540808	1.06E-05	0.001775208	1790
Rpl41	1218.424896	0.833279367	0.189416677	4.399186908	1.09E-05	0.001804093	437
Haghl	1188.317639	0.326793948	0.074305238	4.397993415	1.09E-05	0.001806954	1405
Stambpl1	211.6757419	0.642575349	0.14645244	4.387604269	1.15E-05	0.001880739	1988
Pdcd5	360.5147371	0.520649676	0.118712419	4.385806297	1.16E-05	0.001889021	684
Sult1a1	97.66605709	-0.944239686	0.215854824	-4.374420117	1.22E-05	0.001960082	1370
Spg7	887.3983175	0.403142095	0.092151785	4.374761645	1.22E-05	0.001960082	2481
Farsa	507.7907143	0.486315006	0.111234211	4.371991319	1.23E-05	0.001963871	1826
Agpat3	489.4008149	0.835650653	0.191303488	4.368193492	1.25E-05	0.001971809	1316
Sars	791.0941819	0.267139044	0.061195748	4.365320331	1.27E-05	0.001990507	3570
Pno1	458.496808	0.316342846	0.072485042	4.364250036	1.28E-05	0.001992895	1544
Dnajb1	2247.511491	0.413229598	0.094734896	4.361957583	1.29E-05	0.002006493	2996
Ndufs5	795.2540574	0.392684947	0.090063352	4.360096967	1.30E-05	0.002016218	525
Mpc2	861.5755008	0.412666934	0.094718896	4.356754051	1.32E-05	0.002039773	1025
Adgrl1	11963.07589	0.346997171	0.079663909	4.355763777	1.33E-05	0.002041567	8181
Camk1	825.4466705	0.374376284	0.085977379	4.354357955	1.33E-05	0.002047267	1496
Rpl29	2208.60782	0.432352815	0.099345283	4.352021577	1.35E-05	0.002061741	733
Nrxn1	198.4194879	0.685469827	0.158165363	4.33388078	1.47E-05	0.002213847	1439
Stim2	1657.882564	0.370582264	0.085553996	4.331559964	1.48E-05	0.002217923	4934
Uqcr11	1268.366995	0.532497501	0.122942469	4.331273864	1.48E-05	0.002217923	445
Atg3	1058.254121	0.310674694	0.071746224	4.330188804	1.49E-05	0.002221032	2057
Sin3b	2517.664801	0.252423373	0.058324546	4.327909782	1.51E-05	0.002236258	4115
Ndufa2	459.6775021	0.4220486	0.097629082	4.322980313	1.54E-05	0.002239682	572
Ndufaf7	339.5703901	0.444969508	0.103091215	4.316269895	1.59E-05	0.002293068	1749
Rnf149	538.9889671	0.294592463	0.068284335	4.314202709	1.60E-05	0.002293293	2492
Cic	117.5283711	0.806194015	0.18687863	4.313997881	1.60E-05	0.002293293	596
Klc1	8796.42793	0.637765234	0.147826868	4.314271441	1.60E-05	0.002293293	2504
Golga4	2517.75183	0.410598824	0.095322513	4.307469554	1.65E-05	0.00235406	7583
Nudt3	7733.833601	0.383787848	0.089207397	4.302197575	1.69E-05	0.002402701	2223
Map1lc3b	419.4739535	0.60703216	0.141343419	4.294732395	1.75E-05	0.002460222	766
Map7d2	1046.468564	0.636786717	0.148538248	4.287021873	1.81E-05	0.002530384	1312
Erp29	1008.199386	0.452905911	0.105782265	4.281491915	1.86E-05	0.002554546	1123
Ccny	2138.697653	0.17215775	0.040211883	4.281265589	1.86E-05	0.002554546	5757
Lsm4	541.1411729	0.556251528	0.130134366	4.274439903	1.92E-05	0.002608659	925
Ddx17	1892.712525	-0.459239508	0.107422313	-4.275084906	1.91E-05	0.002608659	2821
Agap3	1335.778498	0.915458703	0.214208449	4.273681591	1.92E-05	0.002609162	1191
Crip2	187.8108	2.895546149	0.677763739	4.272205762	1.94E-05	0.002618104	772
Rbm42	875.9247124	0.469364105	0.109903347	4.270698901	1.95E-05	0.002627463	1526
Zswim9	331.0178517	0.571214259	0.134188078	4.256818248	2.07E-05	0.002759396	3346
Hexim1	2390.832815	0.478577159	0.112441614	4.256228128	2.08E-05	0.002759396	3495
Dnajc2	359.5564144	0.470070349	0.11062478	4.249231953	2.15E-05	0.002811738	2133
Eif3a	2869.450956	0.253544103	0.05965182	4.250400119	2.13E-05	0.002811738	5125
Vdac2	572.734234	0.591901904	0.13940909	4.245791314	2.18E-05	0.002828963	874
Hypk	63.19661496	0.677682036	0.159671734	4.244220437	2.19E-05	0.002829146	961
Klf13	125.8534199	1.210971657	0.285377654	4.243400416	2.20E-05	0.002829146	487
Polr3d	509.0078233	0.30249383	0.071291737	4.243041953	2.21E-05	0.002829146	2016

Rpl36	1338.795378	0.480567357	0.113213768	4.244778407	2.19E-05	0.002829146	383
Tnpo3	1410.598614	0.316843587	0.074705383	4.241241725	2.22E-05	0.002843321	4251
Mtres1	221.3928072	0.403956311	0.095350806	4.236527498	2.27E-05	0.002868981	860
1110065P2	277.1144327	0.56227293	0.133039437	4.226362808	2.37E-05	0.002957491	762
Rpl35	1798.370504	0.352725071	0.083501511	4.2241759	2.40E-05	0.002977595	946
Alyref	553.4680307	0.264103064	0.062539002	4.22301372	2.41E-05	0.002984245	3527
Mark3	1003.363842	0.535599411	0.127041903	4.215927159	2.49E-05	0.003061627	3365
Alkbh3	79.16307557	0.762698607	0.181140802	4.210529043	2.55E-05	0.003103567	1395
Kif3a	703.7291717	0.564296743	0.134050068	4.209596839	2.56E-05	0.003103567	2290
Ranbp1	851.8725253	0.363335557	0.086271789	4.211522247	2.54E-05	0.003103567	1085
Epn1	2964.162769	0.404655321	0.096212945	4.205830322	2.60E-05	0.003143647	2127
Cadm1	240.4415597	0.575414075	0.136839175	4.205039043	2.61E-05	0.003143647	2126
Csde1	882.0988911	0.716596806	0.170502782	4.202845249	2.64E-05	0.003148441	2671
Micu3	966.6368834	0.387519395	0.092200593	4.203003304	2.63E-05	0.003148441	2219
Pspc1	630.8568174	0.269408667	0.064106817	4.202496369	2.64E-05	0.003148441	4114
Rps11	2502.749699	0.318311528	0.075821044	4.198194994	2.69E-05	0.003199817	661
Coprs	363.6219733	0.48701142	0.116048009	4.196637459	2.71E-05	0.003212867	793
Rpl13a	1411.057559	0.394214709	0.093975717	4.194857146	2.73E-05	0.00322916	552
Eif4b	7767.129275	0.241581392	0.057644768	4.19086418	2.78E-05	0.003277369	3854
Grk6	118.2827556	-0.73026321	0.174289923	-4.189933642	2.79E-05	0.003281107	2169
Nol12	37.99053835	0.918805472	0.219319604	4.189344929	2.80E-05	0.003281107	742
Dtymk	155.0049927	0.48489537	0.11589328	4.183981762	2.86E-05	0.003350244	1003
Pfn2	632.0273479	0.704484868	0.168587877	4.178739782	2.93E-05	0.00339381	905
Arf5	2929.258911	0.447762114	0.10715739	4.178546283	2.93E-05	0.00339381	1116
Naca	2087.433552	0.261768576	0.062637459	4.17910591	2.93E-05	0.00339381	885
Far1	111.465872	0.637232496	0.152567594	4.176722451	2.96E-05	0.003411803	733
Srcap	7505.003855	0.220449884	0.05288863	4.168190466	3.07E-05	0.00349793	10654
Rpl37	941.4327073	0.340329995	0.081622395	4.169566384	3.05E-05	0.00349793	835
Map2k1	2954.96674	0.32846943	0.078863206	4.165052965	3.11E-05	0.003533323	2436
Atxn7l3b	4391.885036	0.230036745	0.055244218	4.163996768	3.13E-05	0.00353557	3566
Elf2	134.949026	-0.622530938	0.149634221	-4.160351388	3.18E-05	0.003578077	5919
Lrp8	225.7860248	0.823106339	0.197906436	4.159068062	3.20E-05	0.003588693	2531
Olfm2	135.6473697	1.268787861	0.305235621	4.15674899	3.23E-05	0.003604506	1836
Ttll12	1025.00994	0.335776619	0.080874918	4.151801644	3.30E-05	0.003656096	3774
Rpl3	7222.969192	0.245436265	0.059126502	4.151036429	3.31E-05	0.003658765	1351
Snf8	511.9829128	0.418234211	0.100773146	4.150254573	3.32E-05	0.003660579	1004
Nsmce4a	673.0035609	0.328468753	0.079181504	4.148301543	3.35E-05	0.003673962	1435
Sirpa	161.2358165	0.830955577	0.200369299	4.147120244	3.37E-05	0.003683419	3342
Zeb2	254.7947738	-0.705841953	0.17027652	-4.145268832	3.39E-05	0.003694217	2070
Zfp622	984.7764342	0.684276574	0.165162181	4.143058467	3.43E-05	0.003720453	2802
Aftph	1249.703805	0.23305893	0.056291428	4.14022059	3.47E-05	0.003757148	3998
Cdh3	132.9727431	0.563507595	0.136264115	4.135407147	3.54E-05	0.003824968	4384
Ilrun	109.6458689	0.557696423	0.134889653	4.134464064	3.56E-05	0.003824968	1348
Ppm1g	1550.114501	0.242477366	0.058684053	4.131912421	3.60E-05	0.003844629	2307
Cndp2	1087.288783	0.442982919	0.107222425	4.131439104	3.60E-05	0.003844629	2124
Pfkl	2706.404996	0.335187219	0.081154346	4.130243609	3.62E-05	0.00384562	3730
Taf11	261.7460651	0.47491417	0.115135576	4.124825601	3.71E-05	0.003927109	1220
Prkcsh	833.663286	0.683538519	0.165758425	4.123703039	3.73E-05	0.003936466	1983
Ptma	6330.093749	0.290431267	0.070448975	4.122576216	3.75E-05	0.003945938	1216
Dars2	268.5851238	0.801419933	0.19466992	4.116814421	3.84E-05	0.004015882	3619
Itsn1	2171.143125	0.312577659	0.075924158	4.116972349	3.84E-05	0.004015882	5364
Ube2r2	3003.560209	0.426782345	0.103723056	4.114633341	3.88E-05	0.004040721	3600
Nus1	1913.509169	0.282588177	0.06868512	4.11425613	3.88E-05	0.004040721	4605
Dnajc8	1101.974948	0.257688067	0.062672678	4.111649236	3.93E-05	0.004076611	1401
Fez2	559.161667	0.591225866	0.143828626	4.110627238	3.95E-05	0.004084686	2001
Keap1	50.03354383	1.161935548	0.283060049	4.104908308	4.04E-05	0.004146508	2498
Tmed9	1447.463901	0.308274858	0.075227491	4.097901621	4.17E-05	0.004253411	1420
Ftsj3	565.3925806	0.258138859	0.06307961	4.092271035	4.27E-05	0.004337148	3144
Bod1	1199.060589	0.383469187	0.093853061	4.085846343	4.39E-05	0.00442709	1394
Nkd1	294.2307782	0.43505792	0.106546048	4.083285364	4.44E-05	0.00445497	1474
Eif3g	1525.377604	0.379636026	0.093019838	4.081237223	4.48E-05	0.004473216	1101
Rpl19	3181.406834	0.354779451	0.086923395	4.081518565	4.47E-05	0.004473216	751
Sf3b2	6120.125526	0.28798556	0.070639837	4.076815182	4.57E-05	0.004533284	3222
Plaat3	577.4326425	0.542439663	0.133065165	4.076496361	4.57E-05	0.004533284	1901
Coq7	390.7762935	0.341165222	0.083710482	4.07553766	4.59E-05	0.004541367	914
Gle1	1910.450502	0.324152399	0.079638744	4.070285191	4.70E-05	0.004582642	4030
Srrt	784.5040256	0.725651302	0.178306655	4.069681547	4.71E-05	0.004582642	3024
Dnajc7	915.1864568	0.334296308	0.082125369	4.070560804	4.69E-05	0.004582642	1828
Ahsa2	469.5576854	0.349331264	0.085868116	4.068230224	4.74E-05	0.004594311	3023
Rps12	1364.253288	0.329082618	0.080963032	4.064603436	4.81E-05	0.004629789	584
Zmynd19	605.0706169	0.42385739	0.104328859	4.062705107	4.85E-05	0.00462991	3903
1810037I1	593.2732577	0.361677554	0.089028629	4.062485956	4.86E-05	0.00462991	1160
Zfand2a	783.3122565	0.475185962	0.116954275	4.063006335	4.84E-05	0.00462991	3169
Snrnp25	194.9113829	0.43047987	0.105960911	4.062629013	4.85E-05	0.00462991	821

Zfp91	4340.384199	0.191361029	0.047165669	4.057210062	4.97E-05	0.004667731	5531
Rpusd4	218.8454551	0.339450936	0.08372164	4.054518461	5.02E-05	0.004695394	1821
Cyrib	349.8658338	0.437857425	0.108020494	4.053466248	5.05E-05	0.004706183	1896
Phf23	376.4944226	0.702673759	0.173474688	4.05058378	5.11E-05	0.004754066	2006
Coro2b	87.46521204	1.553318326	0.383654714	4.04874036	5.15E-05	0.004770691	987
Mrpl34	492.0204612	0.321704572	0.079471447	4.048052267	5.16E-05	0.004774288	607
Cd46	102.5748144	-0.614815613	0.151907225	-4.04730989	5.18E-05	0.00477813	6288
Rgs12	746.6267909	0.312031595	0.077104929	4.046843666	5.19E-05	0.00477813	5472
Btbd1	1893.311036	0.214123605	0.052929959	4.04541416	5.22E-05	0.004796954	3001
Grpel1	850.6403245	0.257612876	0.063700815	4.044106466	5.25E-05	0.004813362	3528
Utp3	484.2128291	0.299705593	0.074141826	4.042328162	5.29E-05	0.004839548	1629
Picalm	261.9079432	-0.673458037	0.166692859	-4.040113297	5.34E-05	0.004856525	8209
Dyrk1a	2686.734851	0.210647289	0.052140512	4.039992745	5.35E-05	0.004856525	5759
Pdf	204.4010461	0.4288869	0.106219916	4.037725859	5.40E-05	0.00489318	1306
Fam174a	934.1080715	0.590162948	0.146269909	4.034752957	5.47E-05	0.004919659	2112
Hras	1661.297611	0.493511111	0.122324049	4.03445696	5.47E-05	0.004919659	1199
Gtpbp6	298.8733532	0.547425899	0.135744343	4.03277137	5.51E-05	0.004923731	1617
Rps3	3012.21771	0.246315876	0.061173549	4.026509496	5.66E-05	0.005046003	1908
Atp5a1	17447.42572	0.266328943	0.066253123	4.019870037	5.82E-05	0.005168576	2471
Macf1	2510.762908	0.703775112	0.175302565	4.014630996	5.95E-05	0.005242275	14463
Csnk1d	1958.795407	0.317402397	0.079062735	4.014563829	5.96E-05	0.005242275	3675
Lamtor4	409.3487771	0.417344137	0.103984278	4.013531149	5.98E-05	0.005254349	877
Klf15	74.91262606	-1.042838026	0.259903967	-4.012397497	6.01E-05	0.005268723	1545
Gatd3a	1568.217427	0.36852005	0.091904499	4.009815117	6.08E-05	0.005304711	1378
Clk3	951.9578913	0.455590894	0.113694188	4.007160811	6.15E-05	0.005331679	2496
Rsrp1	3190.098019	0.77937931	0.194539678	4.00627428	6.17E-05	0.005340776	1749
Gins4	361.5885605	0.258999597	0.064663054	4.005372191	6.19E-05	0.005350259	1349
Cmas	2408.005757	0.412577204	0.103020058	4.004824021	6.21E-05	0.005351759	1760
Zfp334	1354.835604	0.219289437	0.054803004	4.001412679	6.30E-05	0.005413072	8205
Arl6ip4	628.641535	0.486198056	0.121514011	4.00116868	6.30E-05	0.005413072	1192
Mfn2	171.3114266	0.544642419	0.136247327	3.997453968	6.40E-05	0.00543258	708
Ube2h	4700.712275	0.214515019	0.053646007	3.998713593	6.37E-05	0.00543258	4657
Leo1	538.7379513	0.417537085	0.104404798	3.999213575	6.36E-05	0.00543258	2180
Cope	2246.156308	0.33879133	0.084808684	3.994771664	6.48E-05	0.005472517	1300
Tm2d1	431.6104499	0.304696994	0.076290971	3.993880131	6.50E-05	0.005475744	1027
Myh1	49.93818748	-9.092329123	2.276674918	-3.993687922	6.51E-05	0.005475744	6012
Pomp	65.69365399	-0.565813122	0.141862968	-3.988448372	6.65E-05	0.005586973	3850
Scp2	1067.199711	0.308899235	0.077528131	3.98435034	6.77E-05	0.005673006	772
Rpl7a	4823.025679	0.348618246	0.087591488	3.98004707	6.89E-05	0.005753897	914
B9d2	186.16686	0.435396116	0.109583832	3.973178436	7.09E-05	0.00587953	995
Noc2l	1409.053169	0.225672943	0.056811074	3.972340752	7.12E-05	0.005879952	2785
D8Ertd738	881.6216886	0.389266887	0.098000486	3.972091397	7.12E-05	0.005879952	616
Jade1	670.4135524	0.305330542	0.076888989	3.971056782	7.16E-05	0.00589406	5584
Rps19bp1	244.6931499	0.498788491	0.125674919	3.968878562	7.22E-05	0.005915916	838
Scyl1	561.4435778	0.519660074	0.130909947	3.969599592	7.20E-05	0.005915916	2586
Rpl7	3938.850739	0.238020462	0.060050844	3.963648931	7.38E-05	0.005920258	1163
Arpc5l	819.8772517	0.371843985	0.093832171	3.962862418	7.41E-05	0.005920258	1339
Ddx27	777.8708972	0.282508631	0.07122716	3.966304871	7.30E-05	0.005920258	2714
Zbtb17	675.2387901	0.469811437	0.11840435	3.967856223	7.25E-05	0.005920258	2722
Rbm19	680.1882059	0.253321523	0.063892554	3.964805089	7.35E-05	0.005920258	4010
Leng8	5599.567426	-0.449010563	0.113309523	-3.962690433	7.41E-05	0.005920258	5105
Psd3	273.3577525	0.688596678	0.173639221	3.965674774	7.32E-05	0.005920258	1739
Ngdn	308.2084906	0.34653888	0.087391958	3.965340622	7.33E-05	0.005920258	1200
Coro1b	2246.950253	0.338523479	0.085469583	3.960747968	7.47E-05	0.005935035	1888
Cog8	1231.538264	0.408335473	0.103127026	3.959538917	7.51E-05	0.00595399	2954
Luc7l2	857.8163063	0.306281466	0.077378936	3.958202087	7.55E-05	0.005965064	4253
Olfm1	501.8452975	1.448193675	0.366011244	3.956691767	7.60E-05	0.005991708	1072
Syt7	1351.147148	0.635679396	0.160714065	3.955343907	7.64E-05	0.006014391	1605
Phpt1	196.1567598	0.456108935	0.11547797	3.949748478	7.82E-05	0.006100041	937
5730409E0	3069.039689	0.350146567	0.088669094	3.948913341	7.85E-05	0.006110106	2855
Snrpf	121.0126814	0.465525251	0.117931643	3.947415987	7.90E-05	0.006137153	861
E2f6	289.668142	0.324648479	0.082272094	3.946033995	7.95E-05	0.006161359	2418
Pdcd6	585.9852438	0.282008001	0.071484013	3.945049946	7.98E-05	0.006175406	1131
Cox7c	1465.670451	0.236694137	0.060017736	3.943736536	8.02E-05	0.006198012	540
Zfp511	191.9532505	0.433697812	0.109988021	3.943136774	8.04E-05	0.006202221	1076
Larp4b	995.1746812	0.36870848	0.093568569	3.940516395	8.13E-05	0.006236289	5720
Uqcc2	1079.512321	0.426471806	0.108250287	3.939682899	8.16E-05	0.006246678	500
Ube2q2	774.1871001	0.575758652	0.146174998	3.93883126	8.19E-05	0.006257573	1680
Diras1	10700.70022	0.443070614	0.112798903	3.927969174	8.57E-05	0.006488409	2953
Atpif1	2390.371445	0.378425964	0.096367274	3.92691365	8.60E-05	0.006497133	539
Nfe2l1	1762.787365	0.406709986	0.103701134	3.921943494	8.78E-05	0.006582223	4654
Polrmt	1396.764826	0.342645297	0.087501637	3.91587298	9.01E-05	0.006690874	3755
Arfgap1	252.7648395	0.509223548	0.130072169	3.914930849	9.04E-05	0.006705291	1209
Bola2	391.4573487	0.580880504	0.148487681	3.911977751	9.15E-05	0.006764143	1266

Strap	2713.809932	0.312980743	0.080033406	3.910626287	9.21E-05	0.006767944	2650
Ctsf	1487.795649	0.263616256	0.067411019	3.910581077	9.21E-05	0.006767944	1980
Clip3	315.0105298	0.782475742	0.200121748	3.90999853	9.23E-05	0.006772529	561
Apmap	881.2128858	0.24049126	0.061531791	3.908406629	9.29E-05	0.006787194	2235
Slc35c2	768.4574026	0.354066532	0.090595273	3.908223022	9.30E-05	0.006787194	2008
Zfp512	988.7661065	0.183374909	0.046913264	3.908807293	9.28E-05	0.006787194	3299
Sfr1	3103.579818	0.402926197	0.103180779	3.90505095	9.42E-05	0.00682974	1657
Erich3	660.0517189	0.315586781	0.080838119	3.903935245	9.46E-05	0.00684959	8280
Rpl28	1670.937421	0.301441033	0.077258475	3.901721255	9.55E-05	0.006885066	1106
Myt1l	1189.071706	0.344826123	0.08837674	3.901774621	9.55E-05	0.006885066	5027
Rgs3	117.8071125	-0.655323242	0.168079411	-3.89889063	9.66E-05	0.006934583	2136
Get3	1666.248694	0.286990836	0.073661293	3.896087392	9.78E-05	0.006991594	1256
Arf1	5899.094951	0.227648663	0.058449764	3.894774742	9.83E-05	0.007017709	1800
Rere	1091.983499	0.703559509	0.18071555	3.893187429	9.89E-05	0.007051914	6503
Hmga1	263.4805158	1.220694122	0.313753759	3.890611939	1.00E-04	0.007103272	1717
Agpat5	942.1232322	0.304795925	0.078474043	3.884034933	0.00010274	0.007261749	2841
Psap	89.88642873	1.186685745	0.305808257	3.880489546	0.00010425	0.007319551	2654
Cox6a1	2675.32666	0.506512837	0.13059404	3.878529492	0.00010509	0.007366551	571
Adrb1	210.4865238	0.536975981	0.138571814	3.875073612	0.00010659	0.007452186	2952
Rab7	2732.358211	0.678768915	0.175324637	3.87149763	0.00010817	0.007520123	1275
Zfp316	473.8945966	0.790579065	0.204290511	3.869876588	0.00010889	0.007548583	3054
Rp9	441.2703129	0.317927136	0.082191174	3.86814203	0.00010967	0.007574591	1134
Strn3	1371.401576	0.278142824	0.071901063	3.868410471	0.00010955	0.007574591	2791
Fam131b	476.9465111	0.496695419	0.128469319	3.866257106	0.00011052	0.00760852	4065
Snx17	1662.611658	0.228691647	0.05917424	3.864716247	0.00011122	0.00764428	2018
Ciapin1	138.7852764	0.991272148	0.256598684	3.86312249	0.00011195	0.007681876	346
Jakmip2	1769.051967	0.269667073	0.069838797	3.861278908	0.0001128	0.007727556	3512
Tsen2	319.0459923	0.401084459	0.103971143	3.85765171	0.00011448	0.007780156	2106
Traf3	3496.54071	0.273944819	0.071000738	3.858337628	0.00011416	0.007780156	7060
Sdha	6015.432442	0.223846543	0.058023099	3.857886695	0.00011437	0.007780156	2900
Zfp397	44.75069779	0.779037248	0.20200941	3.856440399	0.00011505	0.007793772	557
Syt12	918.6218489	0.387867532	0.100575515	3.856480697	0.00011503	0.007793772	3581
Ints1	1767.514319	0.682483352	0.176995083	3.855945256	0.00011528	0.007797091	7070
Trp53bp1	851.2920288	0.238864792	0.061968299	3.854628809	0.00011591	0.007826661	3098
Nlgn2	949.2892978	1.159880345	0.301197295	3.850898942	0.00011769	0.007934184	5003
Fbxo11	2395.712364	0.238692506	0.062019023	3.848698254	0.00011875	0.007980375	4019
Ckap5	62.72322969	1.069853443	0.278102711	3.846972369	0.00011959	0.008013451	6511
Mrps18b	426.5486404	0.322756566	0.083900257	3.846907978	0.00011962	0.008013451	1079
Slc12a6	3198.150174	0.254553327	0.066205727	3.844883824	0.00012061	0.008067122	4556
Pcsk6	23.6395874	-8.013360254	2.085951861	-3.841584461	0.00012224	0.008125011	3734
Hnrnpa0	2063.382442	0.382942822	0.099759007	3.838679187	0.0001237	0.008170431	2678
Platr25	26.43375633	-1.024626245	0.266909453	-3.838853332	0.00012361	0.008170431	2509
Lonp1	1596.195763	0.325500783	0.084773341	3.839659717	0.00012321	0.008170431	2951
Rpp25l	271.6400842	0.433082524	0.113053689	3.830768621	0.00012774	0.008326064	856
Thap1	244.0706376	0.338845461	0.08846596	3.830235505	0.00012802	0.008326064	2295
Exoc8	1958.581622	0.315325991	0.082286486	3.832050753	0.00012708	0.008326064	4598
Msl1	2899.027059	0.416209699	0.108661912	3.8303182	0.00012798	0.008326064	4593
Emc9	760.6888905	0.497716273	0.129897904	3.831595868	0.00012732	0.008326064	855
Cyfip2	219.0452925	0.690216682	0.180243904	3.82934828	0.00012848	0.00834332	6604
Arfip2	1369.099747	0.369781125	0.096606684	3.827697101	0.00012935	0.00838661	3275
Tenm3	562.9930453	0.453353182	0.118454122	3.827246985	0.00012958	0.008389109	2267
Tmem11	311.0701434	0.456243352	0.119270478	3.825283159	0.00013062	0.008427615	1457
Afg3l2	2638.32299	0.309071426	0.080826258	3.823898729	0.00013136	0.008441486	3094
Ikbkb	599.5313884	0.403176845	0.105491327	3.821895671	0.00013243	0.008483836	3580
Ndufs2	3585.098197	0.266058068	0.069651721	3.819834812	0.00013354	0.008540779	1623
Lamtor2	479.3847287	0.320486893	0.083954693	3.817379135	0.00013488	0.008574399	668
Carns1	841.4858136	-0.584113545	0.152999348	-3.817751853	0.00013467	0.008574399	2371
Senp6	231.7704331	0.588861518	0.154313111	3.816017406	0.00013562	0.008608905	1150
Myh4	24.86060912	-8.086024729	2.119338398	-3.81535329	0.00013599	0.00861917	6016
Rnf220	1872.2502	0.410737186	0.107681216	3.814381021	0.00013653	0.008640238	1949
Rps13	531.4498051	0.318193018	0.083469719	3.812077291	0.0001378	0.008690265	589
Sugp2	2028.490556	0.339112523	0.088963321	3.811823998	0.00013795	0.008690265	3874
Trpm3	298.1320741	0.781884911	0.205297828	3.808539607	0.00013979	0.008755277	5803
Snx8	677.3472117	0.373553646	0.09818469	3.804601793	0.00014203	0.008843403	2627
Sumo3	1429.097042	0.345886889	0.090961985	3.802543347	0.00014322	0.00887806	2630
Tomm40	2084.339362	0.394518952	0.103781971	3.801420863	0.00014387	0.008895204	1577
Flywch1	2425.795428	0.604452166	0.159211672	3.796531738	0.00014674	0.009029935	2443
Ppip5k1	1733.568595	0.438345751	0.115485234	3.795686557	0.00014724	0.009047606	5226
Rps28	347.7249927	0.500062781	0.131941151	3.790044104	0.00015062	0.009215496	382
Ndufv3	654.9153047	0.397002409	0.104865516	3.785824213	0.0001532	0.009332853	493
Tcf25	1988.252989	-0.304586544	0.080483949	-3.784438341	0.00015406	0.009362631	5002
Tspyl1	5401.112502	0.302248996	0.079887959	3.78341119	0.00015469	0.009383338	3086
Fbxl14	1478.588561	0.303399893	0.080213546	3.78240221	0.00015532	0.009407987	2963
Supt5	3629.553395	0.334906469	0.088567642	3.781363737	0.00015597	0.009433741	3513

Hnrnpl	2539.64924	0.305835162	0.080887144	3.781010748	0.00015619	0.009433741	1898
Txndc16	156.1853537	-0.789938122	0.209102127	-3.777762247	0.00015824	0.009504039	4069
Fbxl12	236.3800063	-0.768793316	0.203599849	-3.776001414	0.00015937	0.009557225	4182
Med4	274.6533175	0.322065265	0.085304146	3.775493716	0.00015969	0.009563167	1328
Otub1	5603.570667	0.288122759	0.076380378	3.772209123	0.00016181	0.009676288	1713
Mrpl12	1680.816174	0.434891824	0.115399111	3.768589041	0.00016417	0.009762524	1316
Rmnd5a	816.2535573	0.309363004	0.082138804	3.76634414	0.00016566	0.009809349	3768
Tbcc	624.2019042	0.470175582	0.124848393	3.765972245	0.0001659	0.009810243	1164
Smim1	229.5932218	0.747592152	0.198541739	3.765415553	0.00016627	0.009818415	3373
Ccdc12	224.2941608	0.473014268	0.125654038	3.76441758	0.00016694	0.009843978	834
Vezt	318.5565615	-0.470692312	0.125049066	-3.764061009	0.00016718	0.009844322	3705
Ndufb7	1526.065592	0.37624026	0.099993996	3.762628511	0.00016814	0.009887148	623
Anapc13	85.2658925	0.715334371	0.19022685	3.760427996	0.00016962	0.009946903	589
Stip1	6592.698476	0.324756968	0.08635548	3.760699015	0.00016944	0.009946903	2108
Pycr2	580.6143705	0.384206423	0.102226549	3.758382013	0.00017102	0.010006198	1580
Gatc	523.4116204	0.209120501	0.05564307	3.758248774	0.00017111	0.010006198	1797
Hs6st3	1065.353371	0.408295158	0.108663481	3.757427563	0.00017167	0.010025235	1708
Ldhb	9902.215216	0.311394676	0.08291805	3.755451036	0.00017303	0.010080007	1287
Celf6	133.3455109	0.942338328	0.250954017	3.755023885	0.00017333	0.010080007	2973
Pkm	22609.52073	0.375291332	0.099971074	3.753999208	0.00017404	0.010080007	2041
Pld2	103.9501808	-0.697865627	0.185889198	-3.754202147	0.00017389	0.010080007	3391
Rbm39	1007.855202	0.421862938	0.11242033	3.752550265	0.00017505	0.010124615	2711
Nob1	376.740323	0.382007163	0.101822012	3.751714925	0.00017563	0.010141914	1658
Ube2l3	94.98709764	1.348429017	0.359475893	3.751097201	0.00017606	0.010141914	650
Trp53rka	300.1508278	0.364429403	0.097170721	3.750403408	0.00017655	0.0101562	1880
Pithd1	1370.027572	0.273837264	0.073083482	3.746910467	0.00017903	0.01025575	1700
Ddx54	2238.168691	0.467824968	0.124850627	3.747077445	0.00017891	0.01025575	4322
Marcks	6873.245968	0.731276584	0.195184214	3.746596956	0.00017925	0.01025575	4048
Phlda1	670.8436763	0.559618635	0.149385222	3.746144546	0.00017957	0.010260388	1949
Ntpcr	288.0032421	0.308891697	0.08247136	3.745442025	0.00018008	0.010275278	1155
Map2k7	1679.656453	0.414235831	0.110649053	3.743690691	0.00018134	0.010305532	3497
Mrps34	740.3007696	0.451476122	0.120592424	3.743818285	0.00018125	0.010305532	918
Pgam1	10356.03427	0.299562275	0.080074345	3.741051812	0.00018325	0.010372587	1775
Gnl1	2883.153665	0.316911722	0.084742879	3.739685574	0.00018425	0.010415201	2848
Psmc5	2008.729054	0.299203741	0.080015039	3.73934381	0.0001845	0.010415464	1316
Nol10	725.2637823	0.217389492	0.058141771	3.738955437	0.00018479	0.010417676	3021
Stard10	2079.049103	0.633907358	0.169612051	3.737395742	0.00018594	0.010453025	1400
Nrip3	26.06559219	1.538833721	0.411772045	3.737101001	0.00018615	0.010453025	3927
Rfxap	431.8400449	0.437574505	0.117202237	3.733499594	0.00018884	0.01051081	2162
Pakap	1901.072608	0.414417698	0.110993885	3.733698456	0.00018869	0.01051081	6830
Myl6b	440.3819692	0.32096563	0.0859321	3.735107465	0.00018764	0.01051081	1026
Chmp7	1373.384514	0.269330405	0.072141011	3.733388294	0.00018892	0.01051081	2649
Cuedc2	1490.216374	0.424090472	0.11356089	3.734476486	0.00018811	0.01051081	999
H2aj	134.1556619	0.857821753	0.230005015	3.729578475	0.0001918	0.010588876	577
Znhit2	780.0072877	0.443796251	0.118991987	3.729631412	0.00019176	0.010588876	1281
Clec16a	81.67024507	-0.825274959	0.221316886	-3.728929022	0.0001923	0.01060103	3730
Golga3	1692.614228	0.371907744	0.099784263	3.727118215	0.00019368	0.010632568	4805
Zfp609	2844.723845	0.156792676	0.042068831	3.727050912	0.00019373	0.010632568	7777
Btbd2	213.5467255	0.906406594	0.243206322	3.726903914	0.00019385	0.010632568	3026
Sod1	2872.585499	0.349652632	0.093827582	3.726544204	0.00019412	0.010632568	641
Clstn1	9794.614844	0.45849676	0.123070372	3.725484478	0.00019494	0.010649785	3319
Tmed7	977.1292845	0.354191317	0.095108834	3.724063287	0.00019604	0.010696149	1348
Stk11	1805.1213	0.361474673	0.097108699	3.722371684	0.00019736	0.010754227	2566
Cdc34	1002.369702	0.544733975	0.146356579	3.72196438	0.00019768	0.010757747	1266
Dcaf8	2150.127944	0.312424693	0.083972821	3.720545402	0.00019879	0.010790637	2859
Htra1	4473.591485	0.402522009	0.108209981	3.719823306	0.00019936	0.010795217	2041
Upf3a	271.9558419	0.585535306	0.157414132	3.719712436	0.00019945	0.010795217	2057
Nlrx1	45.24971738	0.75760672	0.203686812	3.719468682	0.00019964	0.010795217	775
Rpl4	7479.05927	0.245913957	0.066153723	3.717310943	0.00020136	0.010873917	1538
Ndrg4	8450.784719	0.803123538	0.216162678	3.715366349	0.00020291	0.010930998	916
Dhx38	1303.885808	0.28667247	0.077159078	3.715343409	0.00020293	0.010930998	4463
Serf2	417.9476934	0.28581485	0.077000554	3.711854456	0.00020575	0.011068732	525
Trappc3	843.0619076	0.231532427	0.06242035	3.709245869	0.00020788	0.011155038	1336
Rpl27a	1400.745972	0.342821761	0.092417852	3.709475525	0.00020769	0.011155038	1158
Psmc3	3668.489283	0.391096772	0.105463934	3.708346147	0.00020862	0.01118057	1636
Ralgds	143.098899	1.618300607	0.436494174	3.707496465	0.00020932	0.011189809	3102
Sh3yl1	247.7186597	0.408184065	0.110134258	3.706240669	0.00021036	0.011196479	1750
Brdt	181.0973841	0.352014957	0.095023263	3.70451347	0.0002118	0.011251276	4745
Ccdc124	3110.909601	0.572831135	0.154696357	3.70293876	0.00021312	0.011307184	1356
Suds3	1804.555576	0.374054759	0.101046681	3.701801526	0.00021407	0.011343781	2425
Eif4enif1	153.7495011	0.446428937	0.120681389	3.699235986	0.00021625	0.011444732	869
Rex1bd	971.6656887	0.543834914	0.147096441	3.6971317	0.00021805	0.011521747	671
Foxred1	270.4729271	0.51689976	0.139819813	3.696899239	0.00021825	0.011521747	2289
Sulf2	3325.24145	0.265457416	0.071817122	3.696297032	0.00021877	0.011534715	3927

Ube2v1	4697.208842	0.357277759	0.096719955	3.693940507	0.00022081	0.011568746	2041
Alkbh5	4806.588894	0.28380379	0.076861537	3.692403261	0.00022215	0.011568746	5730
Rpl32	1374.106412	0.201700139	0.054663987	3.689817531	0.00022442	0.011644001	517
Eef2	52720.01158	0.237979894	0.06449368	3.689972334	0.00022428	0.011644001	3089
Swi5	1576.91145	0.363667055	0.098575627	3.68921879	0.00022494	0.011646828	768
Polr2e	590.9963039	0.370061749	0.100311307	3.689132961	0.00022502	0.011646828	1148
Sdhb	2290.198641	0.270812574	0.073437102	3.687680586	0.00022631	0.011692611	1142
Tshz3	1729.22149	0.271526066	0.073640761	3.687170843	0.00022676	0.011692611	5098
Chchd2	7140.218606	0.524050539	0.142177158	3.685898269	0.0002279	0.011724116	915
Rtn4	910.3655625	0.726876242	0.197242107	3.685198113	0.00022853	0.011742126	1272
Mtarc2	897.5618396	0.418732421	0.113667491	3.683836228	0.00022975	0.011790764	1917
mt-Nd6	808.892625	0.815888864	0.22157199	3.682274383	0.00023116	0.011827397	519
B4galt7	417.5739747	0.313296242	0.08512378	3.680478491	0.0002328	0.011875156	2130
Emc8	2106.268381	0.243487566	0.06621323	3.677324993	0.00023569	0.012008414	4942
Ptn	3625.266442	0.450254494	0.122474055	3.676325505	0.00023662	0.012041047	1614
Ncf2	124.2461036	0.873911486	0.237839921	3.674368386	0.00023844	0.012090118	2596
Nedd4l	28.79290138	-1.352919434	0.368175967	-3.674654397	0.00023817	0.012090118	2018
Txndc15	981.9863771	0.239375454	0.065160946	3.673603091	0.00023915	0.012097427	1527
Smarca2	2757.487602	0.650750292	0.177136564	3.673720874	0.00023904	0.012097427	5862
Arhgef12	4859.861449	0.244433302	0.066568336	3.671915464	0.00024074	0.012123375	10059
Fam20b	2445.223604	0.218376308	0.059508866	3.669643234	0.00024289	0.012170043	4532
Nfic	2286.605798	0.669365369	0.1823926	3.669915171	0.00024263	0.012170043	1655
Ubfd1	3763.153354	0.261074323	0.071153484	3.669171323	0.00024334	0.012178116	4849
Mrpl40	430.1503377	0.375141515	0.102295762	3.667224406	0.0002452	0.012242242	1280
Letm1	3186.897087	0.345619271	0.094264449	3.666485899	0.00024591	0.012256397	5272
Syt1	5787.367305	0.269383425	0.073518869	3.664139939	0.00024817	0.012318072	4745
Gne	1247.418313	0.373846048	0.102085041	3.662104087	0.00025015	0.012401865	2920
Ptpra	2478.231682	0.331962421	0.090672674	3.661107648	0.00025113	0.012435643	3066
Aggf1	1040.414035	0.31743863	0.086740105	3.659652369	0.00025256	0.012491866	3249
Coa8	109.4624554	-0.557899301	0.152555031	-3.657036409	0.00025515	0.012575923	3107
Hnrnpf	398.1338624	0.425412393	0.116400174	3.654740178	0.00025744	0.01263184	2401
Atox1	712.4997549	0.51671094	0.141349063	3.655566782	0.00025661	0.01263184	541
Smdt1	1057.635584	0.372035297	0.101789716	3.654939939	0.00025724	0.01263184	705
Cdk5r2	9646.3508	0.593403587	0.162600407	3.649459424	0.00026279	0.012669322	2799
Romo1	250.5492758	0.380882507	0.10426389	3.653062489	0.00025913	0.012669322	569
Slc4a1ap	300.9434589	0.695618509	0.190526348	3.651035761	0.00026119	0.012669322	2499
Uba5	888.6705167	0.245620487	0.067297901	3.649749615	0.0002625	0.012669322	2404
R3hdm2	2367.485744	0.238780383	0.065400289	3.651060065	0.00026116	0.012669322	4281
Rpl38	659.2380031	0.348968578	0.095589995	3.650681011	0.00026155	0.012669322	371
Donson	267.3463158	0.332188282	0.090948161	3.652501368	0.0002597	0.012669322	2365
Evi5	664.4516164	0.330424234	0.090568742	3.648325311	0.00026396	0.012699549	2441
Actn4	679.1882612	0.528807647	0.144993333	3.64711699	0.0002652	0.012737597	1594
Zbtb37	1391.618796	-0.263682509	0.072318891	-3.646108298	0.00026624	0.012751597	18814
Ndufb2	397.9195932	0.370999316	0.101761123	3.645786392	0.00026658	0.012751755	455
Rab40c	108.8483013	-10.21638054	2.802444981	-3.645524037	0.00026685	0.012751755	2456
Agfg1	448.3569476	0.954811084	0.261976145	3.644648957	0.00026776	0.012753089	3142
Klhl17	1240.427655	0.325537746	0.089356568	3.643131685	0.00026934	0.012753089	2723
Timm50	569.3008361	0.266187602	0.073041061	3.644355601	0.00026806	0.012753089	1515
Ndn	5669.791152	0.397655326	0.109159079	3.642897405	0.00026959	0.012753089	3694
Zfp668	24.43051885	1.281319486	0.351729213	3.642914599	0.00026957	0.012753089	626
Tsr1	481.336912	0.355342067	0.097523451	3.64365764	0.00026879	0.012753089	3389
Htatsf1	239.517832	0.466773424	0.128109439	3.643552165	0.0002689	0.012753089	1128
Brd9	429.7077527	0.460557223	0.126437399	3.642571149	0.00026993	0.012755018	2407
Hbp1	143.8698212	-0.624858889	0.171557837	-3.642263733	0.00027025	0.012756027	1319
Arid1b	1852.20018	0.425541671	0.116967855	3.63810786	0.00027465	0.012906037	9888
Bloc1s1	662.0439609	0.415896618	0.114384906	3.635939668	0.00027697	0.013000662	550
Tpr	3496.655999	0.242980973	0.066836627	3.635446378	0.0002775	0.013011141	7430
Samd1	980.8436137	0.414033603	0.113913156	3.634642543	0.00027837	0.013037336	2265
Bpnt1	1229.935245	0.229202492	0.06307787	3.633643518	0.00027945	0.013073483	2200
Sox13	314.8187854	-0.454748516	0.125200133	-3.632172772	0.00028105	0.013119248	3179
Lrrfip2	627.150053	0.363075597	0.100082937	3.627747231	0.00028591	0.013229432	1839
Lrig2	258.1082918	0.332038505	0.091553633	3.62671033	0.00028706	0.013268146	3946
Dlg5	2755.944974	0.33065374	0.091185201	3.626177669	0.00028765	0.013280521	7851
Ivns1abp	649.9267345	-0.359212333	0.099080951	-3.625442918	0.00028847	0.013289867	2783
Mrpl9	533.9054379	0.238186843	0.065732587	3.62357323	0.00029056	0.013346412	1612
Arf3	14095.14332	0.295483638	0.08154389	3.623614688	0.00029051	0.013346412	4566
Arl3	637.772917	0.310173128	0.085600347	3.623503176	0.00029064	0.013346412	843
Tle5	16207.18542	0.31007204	0.085585289	3.622959562	0.00029125	0.013354164	1411
Cyhr1	1152.905159	0.542451963	0.149960516	3.617298578	0.00029769	0.013563924	1854
Rapgef2	2686.138959	0.251355206	0.069530187	3.615051504	0.00030029	0.013626892	6964
Lemd2	1486.119531	0.485383135	0.134347526	3.612892261	0.0003028	0.013726201	2627
Rdh5	40.1883346	-0.998379388	0.276364723	-3.612542794	0.00030321	0.013730019	668
Dynll2	16139.93076	0.33095615	0.091627586	3.611970625	0.00030388	0.013745655	2476
Chpf	3967.685705	0.468919169	0.12990686	3.609656695	0.0003066	0.01383431	2892

Fh1	1292.221896	0.241623888	0.066941589	3.609473444	0.00030682	0.01383431	2636
Ctdp1	998.959275	0.191447752	0.053035892	3.609777175	0.00030646	0.01383431	3713
Dnajb6	1549.570474	0.175080673	0.048538209	3.607069096	0.00030968	0.01393341	1605
Clta	245.3036519	0.38551284	0.107020637	3.602228992	0.0003155	0.014136654	1031
Gkap1	344.6145828	0.373703228	0.103727878	3.602727011	0.0003149	0.014136654	1523
Fam117b	3245.004859	0.287212215	0.079750079	3.601403522	0.0003165	0.014150982	5532
Rbm15b	2959.792285	0.394402476	0.109530535	3.600844968	0.00031719	0.014150982	6511
Trir	1867.708276	0.390301006	0.108442068	3.599166004	0.00031924	0.014219374	902
Borcs6	1762.362615	0.513305374	0.142622706	3.599043858	0.00031939	0.014219374	1862
Vps72	824.1716865	0.346356423	0.09625316	3.598390156	0.00032019	0.014240182	1456
Pcyox1	37.61458054	0.841322474	0.233824422	3.59809496	0.00032056	0.014241391	703
Rpl6	4777.211203	0.2246005	0.062444948	3.596776133	0.00032219	0.014298756	1270
Mrps15	469.8531558	0.379217329	0.105445889	3.596321602	0.00032275	0.014308753	937
Ncor2	78.82756807	-0.610812611	0.169897043	-3.595192714	0.00032415	0.014340911	832
Scfd2	375.9964495	0.450664551	0.125382459	3.594318968	0.00032524	0.014374099	3672
Banp	108.4587795	0.801142411	0.222938741	3.593554034	0.0003262	0.014390093	1289
Hnrnpk	239.3127407	-0.774895265	0.215730664	-3.591956987	0.0003282	0.01442973	2585
Mpped2	600.2570657	0.330500634	0.092022973	3.591501361	0.00032878	0.014434394	2848
Peli2	151.804146	-0.897037718	0.249797313	-3.59106232	0.00032933	0.014434394	1320
Gdap1l1	309.2962192	1.088373345	0.303135359	3.590387309	0.00033019	0.014456862	1206
Arhgef4	609.4085104	0.64094715	0.178593206	3.588866368	0.00033212	0.014526433	1206
Sdad1	577.5912437	0.232611399	0.064858213	3.5864602	0.0003352	0.014630839	5020
Psip1	450.4061897	0.366001965	0.102064966	3.585970559	0.00033583	0.014643227	1898
Aars	6609.704117	0.316274273	0.088210375	3.585454373	0.00033649	0.014657127	5750
Clasp1	368.9463399	0.799445403	0.223184954	3.581986094	0.00034099	0.01476837	5373
Pik3cd	625.2304064	0.987053856	0.275569132	3.581873808	0.00034114	0.01476837	4832
Homer2	561.887082	0.547346782	0.152792507	3.582288123	0.0003406	0.01476837	1736
Fam53b	291.3871589	-0.700879272	0.195647123	-3.582364322	0.0003405	0.01476837	1232
Ankrd42	247.4586079	0.421455967	0.117701993	3.580703751	0.00034267	0.014819211	2782
Mrpl53	261.3090979	0.45084655	0.125945909	3.579683964	0.00034401	0.014847135	695
Eef1b2	965.8184154	0.317001769	0.088570942	3.579071904	0.00034482	0.014851184	1939
Pxn	179.9230221	-0.591915563	0.165405507	-3.578572272	0.00034548	0.014851184	3189
Zbtb7a	641.3125153	0.754319874	0.210841406	3.577664789	0.00034668	0.014886395	1841
Fam171a1	2612.078228	0.295155775	0.082530344	3.576330386	0.00034845	0.014941164	4149
Nrd1	174.9153625	-0.504001271	0.140950026	-3.575744432	0.00034923	0.014941164	2190
Ahdc1	295.3870326	-1.212291175	0.339091436	-3.575115873	0.00035007	0.01495634	6602
Srsf4	1476.768603	0.43103614	0.120632601	3.573131446	0.00035274	0.015024705	2257
Myl6	859.7929648	0.479319621	0.134190235	3.571941131	0.00035435	0.015063222	665
Nudt11	770.0578522	0.560879659	0.15706364	3.571034389	0.00035557	0.015099956	3036
Pja1	2608.582661	0.260782393	0.073057168	3.569566155	0.00035757	0.015169619	2666
Ctbp1	3134.398082	0.436289343	0.122268024	3.568302882	0.0003593	0.015210794	2436
Mapk10	1307.466038	0.469693004	0.131667064	3.56727787	0.00036071	0.015210794	7198
Actl6b	648.4433958	0.330464875	0.092648193	3.566878794	0.00036126	0.015210794	1592
Lmo1	299.3982524	0.511308917	0.143332357	3.567295826	0.00036068	0.015210794	912
Mdga1	1202.46452	0.54877843	0.15385894	3.566763354	0.00036142	0.015210794	7566
Rab10	4475.894084	0.246285448	0.069065681	3.565959901	0.00036253	0.015227205	3513
Septin6	1258.754325	0.321730814	0.09021807	3.56614603	0.00036227	0.015227205	3457
Wrnip1	1277.935641	0.507006184	0.142250661	3.564174538	0.000365	0.015316019	2633
Eif4h	5938.851541	0.292426953	0.082055083	3.563788404	0.00036554	0.015316675	2367
Trim8	4729.709635	0.361098496	0.101328448	3.563643795	0.00036574	0.015316675	3580
Edf1	2177.211574	0.526273841	0.147706542	3.562969065	0.00036668	0.015340939	722
Bsg	9375.062225	0.308264732	0.086576146	3.560619701	0.00036998	0.015417922	1261
Ak2	225.5134886	0.381567984	0.107184445	3.559919388	0.00037097	0.015443896	986
Ccdc117	578.4413896	0.263610097	0.074098681	3.557554516	0.00037432	0.015568251	3372
Dcun1d2	1199.570517	0.262124718	0.073687888	3.557229332	0.00037479	0.015572232	2748
Gmcl1	767.7499418	0.270007301	0.075941666	3.555456654	0.00037732	0.015657276	2949
Ttc7b	123.11174	1.255965184	0.353374467	3.554204677	0.00037912	0.015657276	3307
Rpl8	3257.787812	0.285866434	0.080435175	3.553997782	0.00037942	0.015657276	851
Wdr82	2174.69504	0.190058768	0.05351955	3.551202652	0.00038348	0.015793725	3747
Wdr13	2477.864058	0.325456546	0.091682695	3.549814361	0.0003855	0.015861814	4183
Lrfn1	1996.909863	0.410014151	0.115568771	3.547793646	0.00038847	0.015952992	3060
Rpl23a	2492.816872	0.358302372	0.101049006	3.545827764	0.00039138	0.016041355	560
Rps6ka1	24.55692219	-1.32513775	0.374245862	-3.540821381	0.00039888	0.016285756	2637
Coasy	587.362589	0.352095146	0.099437743	3.54086018	0.00039883	0.016285756	2138
Rab5if	804.1808754	0.370693852	0.104724648	3.539700137	0.00040058	0.016317085	1045
Hspa4	6712.771129	0.268271705	0.075792562	3.539551858	0.00040081	0.016317085	4618
Thap11	988.9254187	0.37327767	0.105492209	3.538438274	0.0004025	0.016338932	1819
Copg1	10549.15066	0.249959886	0.070656217	3.537691334	0.00040364	0.016366924	4140
Tomm20	33.24913338	1.002074181	0.283273419	3.537480455	0.00040396	0.016366924	458
Nudt10	292.3596295	0.473151892	0.133773333	3.536967201	0.00040475	0.016383085	1775
Cox10	771.1642547	0.263526161	0.074525306	3.536062772	0.00040614	0.016407905	2915
Nr2f1	1083.311849	0.401133524	0.113438151	3.536142987	0.00040602	0.016407905	3207
Ptms	12192.61566	0.492454436	0.139279134	3.535737358	0.00040664	0.016412467	1149
Spr	1129.025943	0.416663763	0.117970414	3.531934404	0.00041253	0.01659049	1230

Septin10	63.06698415	0.524180806	0.148524207	3.529261775	0.00041672	0.016707896	1051
Sptbn2	16480.75182	0.399355481	0.113149021	3.529464744	0.0004164	0.016707896	8299
Rpl9	592.4393282	0.651166426	0.184562268	3.528166585	0.00041845	0.016739434	680
Wnk1	32.80234497	0.729593494	0.206781348	3.528333201	0.00041819	0.016739434	181
Pcsk1n	45830.01462	0.605884013	0.171735255	3.528011847	0.00041869	0.016739434	2193
Txnrd1	1048.973937	0.512863506	0.145383573	3.527657879	0.00041925	0.016746019	2297
Comt	436.5272198	0.374863353	0.106279608	3.527142783	0.00042007	0.016762819	1978
Rbm17	777.3177672	0.29640605	0.084094444	3.524680535	0.00042399	0.016763138	1599
Sptan1	57114.36113	0.35537408	0.100814368	3.525034039	0.00042343	0.016763138	7892
Hipk3	5845.862021	0.227025563	0.06437281	3.526730691	0.00042072	0.016763138	7454
Atp8b2	1074.283999	0.544337559	0.154419418	3.525058996	0.00042339	0.016763138	4265
Zfp428	288.2723591	0.486257893	0.137894276	3.526309494	0.00042139	0.016763138	1182
Rab6a	456.0196078	1.395230134	0.395813726	3.524966527	0.00042354	0.016763138	3110
Gprin1	758.1573002	0.38989453	0.110619259	3.524653259	0.00042404	0.016763138	4141
Plec	1449.953512	0.814153983	0.230915203	3.525770384	0.00042225	0.016763138	4937
Spock1	1294.712703	0.62836701	0.178539779	3.519479044	0.0004324	0.017045792	2005
Marchf9	1143.303117	0.590011302	0.167714061	3.517959671	0.00043488	0.017127738	3037
Evi5l	3826.30728	0.38831322	0.110495372	3.514293965	0.00044093	0.017221684	3938
Rai1	747.6106658	-0.499994027	0.142245402	-3.515010109	0.00043974	0.017221684	7217
Gcc1	1007.260884	0.321824635	0.091627651	3.51230913	0.00044423	0.017318872	4650
Oprm1	33.87965167	-1.711407534	0.487356004	-3.511616807	0.00044539	0.017335638	1500
Mea1	758.2614907	0.386254241	0.110031606	3.510393555	0.00044744	0.017396077	945
Mrps10	121.7373612	0.58127526	0.165613087	3.509838938	0.00044838	0.017415536	1018
Ppp1r14b	857.8395066	0.58999139	0.168161513	3.508480508	0.00045067	0.017457546	999
Rapgef4	3281.341261	0.289998578	0.08267193	3.507823991	0.00045179	0.017484685	4181
Wipi2	651.682491	0.478233195	0.136417067	3.505669817	0.00045546	0.017591808	1959
Jmy	4095.280885	0.162252286	0.046279381	3.505930346	0.00045501	0.017591808	8776
Ppp2r5c	247.9069689	0.460195777	0.131375691	3.502898999	0.00046022	0.01769793	1971
Zfp280c	337.4817392	-0.552851172	0.157841779	-3.502565528	0.0004608	0.017704019	4045
Ecsit	420.2597381	0.46257547	0.132137906	3.500702291	0.00046403	0.017779848	1676
Safb	480.7046781	0.852323884	0.243471012	3.50072017	0.000464	0.017779848	3106
Fam214b	421.4892923	0.446658648	0.127686	3.498101957	0.00046858	0.017921673	3280
Grip1	225.2638753	0.540334545	0.154487145	3.497601983	0.00046946	0.017939095	4857
Ccne1	306.6803275	0.35815673	0.102484016	3.49475699	0.00047449	0.018066172	2000
Pdgfa	1663.54853	0.371767532	0.106392771	3.494293166	0.00047532	0.01808133	2060
Cep170b	9323.126489	0.338652479	0.096961361	3.492653919	0.00047825	0.018160047	6641
Nelfe	340.2838686	0.633713888	0.181555099	3.490476947	0.00048216	0.018275863	1381
Ndufb6	534.5041539	0.266471835	0.076364903	3.489454247	0.00048401	0.018293644	659
H2ax	1101.76573	0.427389112	0.1224754	3.489591464	0.00048376	0.018293644	1384
Nosip	1845.346016	0.366399913	0.105033167	3.488421071	0.00048588	0.01833491	1814
Dync1i1	153.271004	-0.665471244	0.190788992	-3.487996013	0.00048666	0.01834773	2518
Ube2j2	282.387409	0.427879234	0.122716975	3.48671596	0.00048899	0.018402748	3483
Atp2a2	11212.18136	0.368683492	0.105746569	3.486481848	0.00048942	0.018402748	4486
Svip	1828.460994	0.228802102	0.06564877	3.485245842	0.00049169	0.018439858	3053
Hddc2	141.2525047	0.416128403	0.119450738	3.483682132	0.00049457	0.018465124	858
Bola3	129.3974927	0.581948242	0.167074905	3.483157703	0.00049554	0.018485034	554
Dlg4	2115.052091	0.364703171	0.104753833	3.481525784	0.00049857	0.018549013	3488
Rnps1	1093.522774	0.287532418	0.082654017	3.478747071	0.00050376	0.018709523	1920
Egln2	2925.345383	0.407381295	0.117125011	3.478175081	0.00050484	0.018733062	2107
Cir1	502.3407886	0.321346267	0.092504814	3.473832888	0.00051308	0.018892824	2821
Rcl1	360.9686836	0.235717913	0.067862568	3.473459938	0.00051379	0.018899676	1803
Pura	6735.787429	0.298661965	0.085997855	3.472900184	0.00051487	0.018922696	5957
Ccdc59	122.7812962	0.53969805	0.155447049	3.471909268	0.00051677	0.018976206	1813
Fau	645.6891453	0.817835258	0.235617848	3.471024223	0.00051848	0.019005894	472
Polg	749.3712684	0.227462711	0.065540005	3.470593407	0.00051931	0.019009245	7837
Smurf2	59.02254168	4.518024933	1.301832286	3.470512278	0.00051947	0.019009245	2358
Mrpl28	843.4125175	0.278326871	0.080211531	3.469910967	0.00052063	0.019035399	1068
Dnajb12	349.2854256	0.699559798	0.201638571	3.46937491	0.00052167	0.019048485	2238
Ddit3	238.2231853	0.380508242	0.109702283	3.46855353	0.00052327	0.019065968	890
Sgsm3	1397.412174	0.273818711	0.078957645	3.467918911	0.00052451	0.019094031	2997
Phf3	152.660723	0.499286997	0.144047837	3.466119364	0.00052803	0.019156994	941
Cdc42bpb	7294.298755	0.273740343	0.0789735	3.466230348	0.00052781	0.019156994	6714
BC003965	523.5328095	0.291774846	0.084191383	3.465614118	0.00052902	0.019176593	3030
Stard7	1473.765499	0.242469456	0.069998528	3.463922237	0.00053236	0.019198983	3116
Eif3c	5098.272748	0.240831507	0.069523109	3.464049705	0.00053211	0.019198983	3383
Tedc1	77.43675464	0.558758558	0.161291359	3.464280791	0.00053165	0.019198983	2441
Slc45a4	1523.324548	0.500436314	0.144444683	3.464553373	0.00053111	0.019198983	4240
Drap1	1373.088697	0.344474163	0.099481244	3.462704625	0.00053478	0.019253268	992
Tmem150c	1713.71846	0.360961331	0.104342099	3.459402635	0.00054138	0.019337542	3084
Mthfd2	551.1818292	0.224680387	0.064967652	3.458342414	0.00054351	0.019337542	2085
Prr12	5161.802027	0.279067785	0.080690319	3.458503903	0.00054318	0.019337542	7023
Gm2296	683.2349088	0.620541133	0.179409158	3.458804103	0.00054258	0.019337542	988
Ncor1	890.5692848	0.221145339	0.063925217	3.459438231	0.0005413	0.019337542	2979
Ubtf	661.0373941	0.567994114	0.164130256	3.460630148	0.00053891	0.019337542	3258

Homer1	290.161295	0.487926818	0.141051359	3.459213884	0.00054176	0.019337542	2468
Pip4p1	68.89520941	9.594641	2.774004682	3.458768855	0.00054265	0.019337542	1445
Tnk2	65.44873146	9.520605383	2.752007497	3.45951288	0.00054115	0.019337542	939
Prxl2b	508.7509713	0.365418737	0.105691846	3.457397629	0.00054542	0.019350983	883
Sap18b	1056.923189	0.337801921	0.09770824	3.457251101	0.00054572	0.019350983	789
Chd3	3790.729988	0.288296442	0.083382465	3.457518842	0.00054517	0.019350983	7261
Abcc4	177.0484882	1.288576062	0.372814569	3.456345783	0.00054755	0.019399851	5504
Arf6	2541.272971	0.414962376	0.120096688	3.455235792	0.00054981	0.019447352	1534
Apc	1671.922854	0.378405122	0.109510377	3.455427086	0.00054942	0.019447352	3588
Zmiz2	1854.980071	0.387212177	0.112100696	3.454146068	0.00055204	0.019509807	3806
Arpc4	125.7161239	0.808673498	0.234215329	3.452692453	0.00055502	0.019538196	794
Fbxl17	2237.722939	0.366546489	0.106164413	3.452630484	0.00055515	0.019538196	4925
Bad	315.3453595	0.342717429	0.09924565	3.453223673	0.00055393	0.019538196	1452
Impdh2	684.0185977	0.219194893	0.06350824	3.451440224	0.0005576	0.019566575	1858
Cct5	190.2424155	0.614353477	0.17800417	3.45134317	0.0005578	0.019566575	845
Ube2e3	1461.260223	0.317151867	0.091904972	3.450867325	0.00055879	0.019568679	2249
Brd2	1840.455021	0.335050243	0.097090145	3.450919159	0.00055868	0.019568679	4657
Maco1	1559.608654	0.213205804	0.061796151	3.450146982	0.00056028	0.019596098	3826
Snapc2	1048.473928	0.315265508	0.091396221	3.449437014	0.00056176	0.019607766	1536
Cnnm2	604.5969121	0.299799357	0.086933966	3.44858712	0.00056353	0.019637199	4955
Svil	76.28384552	-1.610702553	0.467241046	-3.447262537	0.0005663	0.019701307	7423
Myo10	1143.984587	0.514116251	0.1491961	3.445909451	0.00056914	0.0197192	5880
Mrpl18	476.6596703	0.319258519	0.09263766	3.446314601	0.00056829	0.0197192	1219
Ppp1r3d	277.6091097	0.39778764	0.115552358	3.442488305	0.00057639	0.019921408	3267
Prkaca	12410.34321	0.311287385	0.090441773	3.441854062	0.00057774	0.019935625	2276
Rab24	1002.684324	0.44812932	0.130195952	3.441960476	0.00057752	0.019935625	1168
Hsdl2	1913.353043	0.306936279	0.089189861	3.441380823	0.00057875	0.019954266	2611
Fsd1	1988.125903	0.409832519	0.119108385	3.440836831	0.00057992	0.019978156	1748
Zbtb18	821.8730866	0.375516409	0.109161989	3.439992364	0.00058173	0.020024302	3734
Cracd	2826.371587	0.406571053	0.118207782	3.439460972	0.00058287	0.020047356	6921
Jph4	8695.580965	0.482343678	0.140308531	3.437735916	0.0005866	0.020126447	4489
Scg3	6339.711148	0.349939118	0.101826388	3.436625051	0.00058901	0.020169224	2152
Dus3l	778.9754411	0.403233519	0.117355436	3.436002058	0.00059037	0.020190248	2025
Pcp4	1076.36614	0.35544708	0.10346462	3.435445675	0.00059158	0.020215422	641
Mid2	59.76255758	-0.652675917	0.190019276	-3.434787937	0.00059302	0.020231891	757
Iqce	172.1188338	0.58003499	0.168933581	3.433509105	0.00059582	0.020306698	2494
Coq2	1012.149424	0.260046311	0.075773135	3.431906467	0.00059935	0.020391862	1743
Cacna2d2	562.4124264	0.388684349	0.113260266	3.431780283	0.00059963	0.020391862	5332
Lurap1l	678.2116499	0.340536133	0.099241757	3.43137952	0.00060052	0.020405625	3066
Rbm45	571.5770235	0.194953284	0.056823033	3.430884898	0.00060162	0.020410106	1898
Thyn1	237.1106218	0.476966019	0.139156335	3.427555199	0.00060904	0.020546812	918
H2bc4	1049.733667	0.513841571	0.14989623	3.427981945	0.00060809	0.020546812	1156
Rap1gap2	282.9404143	0.461152717	0.134560381	3.427106212	0.00061005	0.02056443	764
Phax	513.2191065	0.264755699	0.077267645	3.426475586	0.00061147	0.020589966	1942
F8a	607.7667166	0.400686476	0.116950464	3.426121299	0.00061227	0.020589966	2505
Snrnp27	477.0362056	0.320916576	0.09369634	3.425070576	0.00061464	0.020595949	885
Etfb	1135.844901	0.445210759	0.129981949	3.425173735	0.00061441	0.020595949	893
Stx4a	484.0181044	0.24025316	0.070133643	3.425647779	0.00061334	0.020595949	1456
Rad23b	5659.897635	0.323161015	0.094379801	3.424048504	0.00061696	0.020635402	3810
Csnk1g2	3042.839063	0.438731454	0.128148537	3.423616578	0.00061794	0.020635402	2348
Yeats4	394.7292829	0.33323242	0.097345503	3.423192747	0.0006189	0.020649079	1327
Camk2g	924.8194979	0.432410922	0.126339096	3.422621619	0.0006202	0.020659948	673
Zfp787	497.7356017	0.331238518	0.096789021	3.422273656	0.000621	0.020670145	1897
Zmat3	1804.415903	0.439969308	0.128625071	3.420556396	0.00062493	0.020752146	7797
Med30	262.3786446	0.37421145	0.109453898	3.418895591	0.00062876	0.020862857	1007
Nap1l3	1235.524073	0.296555611	0.086766769	3.417847814	0.00063118	0.020910559	2837
Camta1	679.2717207	0.413491397	0.121036471	3.416254563	0.00063489	0.020935009	4961
Mrpl51	632.2069915	0.281293868	0.08233626	3.41640326	0.00063454	0.020935009	3427
Mtch1	5482.542058	0.443060363	0.12968681	3.416387239	0.00063458	0.020935009	1952
Mocs3	490.9087574	0.482736599	0.141315029	3.416031565	0.00063541	0.020935855	1973
Slc22a17	1593.791634	0.559310951	0.163777114	3.415073922	0.00063765	0.020993264	2294
Abcg1	5180.850261	0.205002059	0.060074364	3.41247158	0.00064377	0.021142144	5832
Sbds	898.125923	0.249879508	0.073247568	3.411437588	0.00064621	0.021143688	1548
Plpbp	466.9358627	0.302578199	0.088703889	3.411104086	0.000647	0.021143688	3603
Nr2f6	1565.010013	0.486900874	0.142752398	3.410806971	0.00064771	0.021143688	2218
Tceal1	380.238838	0.26218889	0.07686711	3.410937286	0.0006474	0.021143688	2803
Noct	591.4764595	0.595121551	0.174502457	3.410390669	0.0006487	0.021158422	2992
Rell2	2211.13697	0.434048904	0.127279722	3.410196835	0.00064916	0.021158422	1762
Zxdc	256.2379599	1.273528166	0.373646688	3.408375359	0.00065351	0.021169802	1612
Dnajc12	104.8848439	0.678483934	0.199046786	3.408665601	0.00065281	0.021169802	592
Khnyn	436.0777099	0.303584209	0.089056731	3.408885628	0.00065229	0.021169802	3898
Ccdc134	128.391433	0.416021282	0.122056765	3.408424621	0.00065339	0.021169802	2046
Tfcp2	180.7350755	0.648555963	0.190247804	3.409006301	0.000652	0.021169802	1903
Ak6	125.2963895	0.371946751	0.109150068	3.407663934	0.00065522	0.021208841	1298

Nme2	380.6935146	0.414024389	0.121537068	3.406568848	0.00065785	0.02127784	734
Ptcd1	867.2517376	0.209054081	0.061384453	3.405651949	0.00066006	0.021301719	3087
Timm10	320.7062097	0.308671865	0.090672582	3.404246987	0.00066347	0.021306027	690
Hey1	1180.115214	0.279774354	0.08216375	3.405082589	0.00066144	0.021306027	2423
Mapt	899.8856033	0.836522937	0.245723896	3.404320661	0.00066329	0.021306027	1362
Map3k12	589.7888305	0.881122686	0.258838366	3.404142516	0.00066372	0.021306027	5185
Calm2	9420.670398	0.28699646	0.084308331	3.404129292	0.00066375	0.021306027	1205
Fgf13	856.1077206	0.425494324	0.124981791	3.404450538	0.00066297	0.021306027	2499
Gps1	578.7854146	0.400702779	0.117754719	3.402859621	0.00066685	0.021372872	2000
Cacul1	389.6180442	0.485679615	0.142722334	3.402968556	0.00066658	0.021372872	1305
Zfyve19	687.4912411	0.385871771	0.113481953	3.400291959	0.00067314	0.021459211	2008
Atp2a1	53.46109996	-3.295674417	0.96940262	-3.399696213	0.00067461	0.021459211	3477
Eif5a	338.6276541	0.618735849	0.181993786	3.399763592	0.00067444	0.021459211	1259
Mafg	143.9119976	0.764032511	0.224936392	3.396660286	0.00068214	0.021602965	840
Scand1	1180.698777	0.63901426	0.188185333	3.395664522	0.00068462	0.021631505	803
Sem1	439.218464	0.356287857	0.104912745	3.396039783	0.00068368	0.021631505	1472
Ctr9	1822.943562	0.232229704	0.068387389	3.395797222	0.00068429	0.021631505	4300
Yjefn3	192.9917934	0.56597203	0.166732408	3.394493231	0.00068756	0.021675721	785
Tmub2	977.5156259	0.457154706	0.134665189	3.394750415	0.00068691	0.021675721	1876
Myl12b	2196.541175	0.383318645	0.112922588	3.394525853	0.00068748	0.021675721	1001
Dhtkd1	133.9993157	-1.094109751	0.322415835	-3.393473989	0.00069012	0.021740356	5480
Atp13a2	5020.044387	0.463788447	0.136695629	3.392854982	0.00069168	0.021757165	3882
Ttbk2	230.3206322	2.282515903	0.672809347	3.392515148	0.00069254	0.021767999	1684
Rxrb	901.6010125	0.289722679	0.085409648	3.392153982	0.00069346	0.021780544	2627
Zcchc3	551.5256194	0.325150822	0.095878998	3.391262202	0.00069572	0.021821896	3073
Slc38a6	1788.771015	-0.452063057	0.133311625	-3.391025038	0.00069632	0.021821896	3927
Atg9a	2596.176493	0.498248113	0.147039307	3.388536858	0.00070267	0.021859052	4041
Brd3os	345.9149759	0.321587368	0.094891402	3.389004293	0.00070147	0.021859052	1898
Taok2	5671.073483	0.364210434	0.107437822	3.389964775	0.00069902	0.021859052	4720
Tsen54	262.8579829	0.313333082	0.092421283	3.39026979	0.00069824	0.021859052	1979
Foxk2	3196.35776	0.215805455	0.063686866	3.38853942	0.00070266	0.021859052	5029
Dbn1	884.183157	0.55517103	0.163829745	3.388707152	0.00070223	0.021859052	3078
Ddi2	1375.832212	0.339826775	0.100304576	3.387948871	0.00070417	0.021889882	3539
Tufm	1847.226301	0.323816476	0.09563841	3.38584129	0.0007096	0.021994091	1679
Lrp12	650.7596859	0.456867811	0.1349269	3.386039479	0.00070909	0.021994091	4244
Tmem65	4337.717836	0.315174087	0.093091658	3.385631897	0.00071015	0.021994777	3747
Galnt16	53.57736514	-0.760526178	0.224731214	-3.384159079	0.00071397	0.02204859	771
Cdv3	1939.503521	0.231245147	0.068352443	3.383129215	0.00071665	0.022083118	3787
Tnik	1985.582932	0.458357181	0.135599812	3.380219891	0.00072428	0.022252667	4404
Pam16l	279.4240405	0.33328208	0.09859214	3.380412275	0.00072377	0.022252667	563
Fam104a	1463.789252	0.364667248	0.107888761	3.380029982	0.00072478	0.022252667	2619
Rnf19b	1282.378563	0.677878185	0.200608868	3.379103791	0.00072723	0.022295438	2532
Cmtr1	195.058558	0.55533799	0.164341543	3.379169876	0.00072705	0.022295438	1271
Timmdc1	802.302133	0.2655932	0.078644618	3.377131277	0.00073246	0.02242347	1573
Plekha3	1118.804869	0.214644206	0.063600364	3.374889592	0.00073845	0.022460751	2534
Smarca4	7388.931433	0.359284054	0.10643377	3.375658443	0.00073639	0.022460751	5405
Bag6	2146.193871	0.29679597	0.087928266	3.375432985	0.000737	0.022460751	1722
B4galt6	6573.071091	0.292568814	0.08670613	3.374257548	0.00074015	0.022496229	5808
Stmn1	6816.154044	0.3155807	0.093547115	3.373494742	0.00074221	0.022515847	1060
Rhpn1	142.8812425	0.979334432	0.290308611	3.373425365	0.00074239	0.022515847	1932
Lrrc47	2403.772913	0.430098978	0.127515602	3.372912581	0.00074378	0.022541672	3395
Gabrb3	217.1403461	1.185959451	0.35201602	3.369049658	0.00075428	0.022746006	4348
Cbr1	1094.802128	0.367873816	0.109181863	3.369367461	0.00075341	0.022746006	1237
Egln1	3040.22797	0.261110284	0.077529951	3.367863379	0.00075753	0.022795391	3594
Tmem242	689.557363	0.271803082	0.080702713	3.3679547	0.00075728	0.022795391	948
Kif5b	14179.96943	0.231002258	0.068597054	3.367524459	0.00075846	0.022807219	6032
Nudt4	4946.399385	0.299021439	0.088801484	3.367302289	0.00075907	0.022809403	3210
Car10	1564.438369	0.272195118	0.080867246	3.365950145	0.00076281	0.022856616	3334
Eif6	1155.899097	0.339282657	0.100812957	3.36546676	0.00076414	0.022864339	1500
Atad2	515.4009052	0.285089055	0.084715526	3.365251563	0.00076474	0.02286602	5684
Rpl11	800.3183343	0.466826103	0.138805728	3.363161662	0.00077055	0.023007343	833
Mrpl48	505.8048446	0.308582051	0.091775418	3.362360593	0.00077279	0.023057937	1625
Fkbp15	144.6670347	-0.567819025	0.168963103	-3.3606096	0.00077771	0.023171937	3329
Phf20	3259.257413	0.215031657	0.064010062	3.359341499	0.00078129	0.023179809	5737
Dhrs3	206.8829147	0.699632919	0.20836665	3.357701044	0.00078594	0.023179809	2337
Rac1	7286.866089	0.235463066	0.070118069	3.358093992	0.00078482	0.023179809	2325
Gadd45a	187.6469444	0.516909103	0.153924019	3.358209504	0.00078449	0.023179809	1223
Itfg2	729.2622872	0.251631145	0.074943288	3.357620852	0.00078616	0.023179809	2320
Nap1l4	2320.422684	0.205311469	0.061134052	3.358381496	0.000784	0.023179809	2259
Itm2b	15418.69012	0.311501484	0.092746564	3.358630977	0.0007833	0.023179809	1796
Zc3h7b	6480.936281	0.213161299	0.063485945	3.357614026	0.00078618	0.023179809	5740
Gramd4	1085.095757	0.323756617	0.096384966	3.358994986	0.00078227	0.023179809	4292
Hsd17b4	1573.88913	0.234093203	0.069712285	3.357990684	0.00078511	0.023179809	2684
Clstn2	1274.807169	0.294386301	0.087685131	3.357311518	0.00078704	0.023189039	4174

Dbnl	1989.448322	0.414630086	0.123511522	3.357015427	0.00078789	0.023189053	2001
Tango2	86.57692448	0.532044119	0.158497972	3.356788187	0.00078854	0.023189053	632
Rrs1	797.8259364	0.542312882	0.161768023	3.352410885	0.00080111	0.023280445	2048
Dedd2	303.0206666	0.672315571	0.200414223	3.354630023	0.00079471	0.023280445	1828
Kars	1838.083723	0.245647316	0.073265139	3.352854005	0.00079983	0.023280445	2008
Rpl13	4959.120015	0.364788542	0.108808917	3.352561103	0.00080068	0.023280445	780
Ccdc85a	1342.731536	0.32412881	0.096666575	3.353059847	0.00079923	0.023280445	5332
Pitrm1	711.7271955	0.330579473	0.098549788	3.354441225	0.00079525	0.023280445	3318
Khsrp	4746.627878	0.244516047	0.072908402	3.353743043	0.00079726	0.023280445	3978
D030056L2	412.6084264	0.493727152	0.147192993	3.354284345	0.00079571	0.023280445	1677
Tars	712.3771028	0.233685312	0.069713905	3.352061691	0.00080212	0.02329288	4005
Cactin	1015.39628	0.41777619	0.124645374	3.351718372	0.00080312	0.023305798	2715
Map1lc3a	7657.712005	0.536351396	0.160067293	3.350786941	0.00080582	0.023307154	1112
Gadd45gip	823.3659489	0.484940494	0.14473391	3.35056583	0.00080647	0.023307154	2494
Taco1	369.5205461	0.318445357	0.095031597	3.350941878	0.00080537	0.023307154	1383
Hnrnpa1	1749.967724	0.165283899	0.049317211	3.351444534	0.00080391	0.023307154	1737
Arxes1	447.7368754	0.481808535	0.143792881	3.350712027	0.00080604	0.023307154	1580
Ehmt2	247.8074325	0.374580228	0.111844185	3.349125636	0.00081067	0.023364853	1325
Mark4	3947.66478	0.37002379	0.110491802	3.348880047	0.00081139	0.023369664	3966
Ddah1	1954.090119	0.242585421	0.072450209	3.348305322	0.00081307	0.02337049	3764
Gspt1	686.1308622	0.409428713	0.122275056	3.348423855	0.00081273	0.02337049	2898
Msra	228.7889473	0.398630707	0.119063425	3.34805343	0.00081381	0.023375873	1180
Pigc	138.754621	0.572722893	0.171137179	3.346572001	0.00081817	0.023413353	3290
H2az1	1049.895427	0.252955102	0.075573118	3.347157161	0.00081645	0.023413353	1069
Wrap73	442.7031203	0.348992523	0.104315499	3.345548146	0.0008212	0.023413353	1598
Emc10	1665.965341	0.444352329	0.132798284	3.346069813	0.00081966	0.023413353	1662
Hdac2	1636.620759	0.315579588	0.09429859	3.346599218	0.00081809	0.023413353	2005
Lrrc3b	259.7380515	0.65661129	0.196225926	3.346200481	0.00081927	0.023413353	1655
Numa1	5380.191821	0.295356831	0.088300767	3.344895412	0.00082314	0.023436958	7180
0610010K1	34.49956547	0.991861435	0.296633916	3.343722288	0.00082662	0.023488893	545
Ppp1r12c	2606.984257	0.369582413	0.110542485	3.343351786	0.00082773	0.023504505	2983
Mbp	4485.983787	-0.603385095	0.180539348	-3.342125155	0.0008314	0.023576999	4797
Slc25a42	53.24276517	-9.184735141	2.748852527	-3.341297887	0.00083388	0.023609126	3081
Dnaja2	2283.704809	0.226591896	0.067817724	3.341189923	0.0008342	0.023609126	2919
Tceal3	687.4005282	0.379607924	0.113630946	3.340709	0.00083565	0.023634257	1116
Fam131a	207.1512277	0.379064031	0.113508938	3.339508213	0.00083927	0.023705001	962
Gpatch11	654.5615185	0.271115047	0.081180767	3.339646272	0.00083885	0.023705001	4134
Mtln	273.4319216	0.349579526	0.104745638	3.337413695	0.00084562	0.023804984	800
Zc3h11a	1158.162797	0.47347305	0.142014446	3.333978073	0.00085613	0.024029848	4395
Mprip	9737.267119	0.320604522	0.096155756	3.334220797	0.00085539	0.024029848	7765
Cops6	663.0567294	0.29702552	0.089116858	3.332989125	0.00085918	0.024074751	1787
Eri3	115.0775069	-0.603805156	0.181256548	-3.33121845	0.00086467	0.024164452	830
Pacsin2	1702.348938	0.446395554	0.134000171	3.331305863	0.0008644	0.024164452	3186
Jakmip1	827.0102147	1.113352822	0.334286895	3.330530869	0.00086681	0.024208231	2496
Mapre2	189.4330353	-0.531729418	0.159722311	-3.329086682	0.00087131	0.024302051	3052
Serp2	323.1246556	0.359233447	0.107921782	3.328646345	0.00087269	0.024324478	748
Sec22b	1593.418104	0.319668604	0.096061086	3.327763792	0.00087546	0.024353566	2810
Hsd17b11	84.18090775	-0.775880276	0.233321088	-3.325375702	0.000883	0.024498761	2234
0610012G0	858.3688252	0.417942147	0.12567907	3.325471354	0.00088269	0.024498761	1445
Rps7	2454.788585	0.253358387	0.076199551	3.324932794	0.0008844	0.024521643	954
Pitpna	3741.587042	0.337304204	0.101470503	3.324160176	0.00088685	0.024542462	1665
Cltb	3409.667606	0.323821473	0.097412386	3.324233056	0.00088662	0.024542462	2186
Mid1ip1	5042.332095	0.383541815	0.115391627	3.323827084	0.00088791	0.024542462	2019
Nlgn3	3240.010138	0.242672804	0.073008823	3.323883248	0.00088773	0.024542462	8465
Cbfa2t2	51.91466274	-0.843070642	0.25371141	-3.322951235	0.00089071	0.024599838	3247
Hspd1	187.7958258	0.474277798	0.142776894	3.321810587	0.00089435	0.024652375	863
Ino80b	609.1465923	0.39305544	0.118332483	3.321619148	0.00089497	0.024653243	1503
Rundc1	1004.810893	0.257803863	0.077629491	3.320952644	0.00089711	0.024679923	3223
Cgrrf1	29.53680747	-0.70806077	0.213221667	-3.320773078	0.00089769	0.024679923	419
Rexo1	2262.709987	0.300618006	0.090550777	3.319883244	0.00090055	0.024742654	5250
Lysmd2	1351.785323	0.373312761	0.112484066	3.318805707	0.00090403	0.024815501	1189
Gemin6	42.7779878	0.597047677	0.17990407	3.318700218	0.00090438	0.024815501	1134
Cep120	584.1471198	0.420931764	0.126843792	3.318505036	0.00090501	0.024816766	3261
Vps45	588.9767457	0.179854355	0.054228191	3.316620955	0.00091113	0.024904062	2637
Pus1	383.064245	0.500844832	0.150990604	3.317059599	0.0009097	0.024904062	1814
Tfdp2	95.73071654	0.811715238	0.244774986	3.316169064	0.00091261	0.024928275	2341
Mrps26	485.1232476	0.585657681	0.176646031	3.315430743	0.00091502	0.024978102	4278
3830406C1	214.2493234	0.570540979	0.172096169	3.315245093	0.00091563	0.024978593	2292
Prx	879.2901246	-1.131306897	0.341362439	-3.314093083	0.00091941	0.025017259	4497
Fbxl3	1080.653974	0.313124717	0.094482228	3.314112336	0.00091935	0.025017259	4309
Ncoa4	863.1267341	0.559704457	0.168914144	3.313544047	0.00092122	0.025030583	4065
Ncaph2	1323.856134	0.444160613	0.134117912	3.31171732	0.00092725	0.025149851	3299
Chchd6	546.2845819	0.42151061	0.127346207	3.309958114	0.0009331	0.025253479	1121
Mgat2	1398.97723	0.357807148	0.108142764	3.308655484	0.00093745	0.025287688	2614

Slmap	379.8049888	0.43768973	0.132289363	3.30857842	0.00093771	0.025287688	3962
Mark2	113.504961	-0.93102049	0.281380287	-3.308762314	0.00093709	0.025287688	3018
Eid2	904.5644411	0.498114527	0.150582438	3.307919127	0.00093992	0.025331159	1392
Lonrf2	4174.152429	0.242094531	0.073201317	3.307242835	0.00094219	0.025376245	6932
Eral1	620.1784688	0.308418441	0.093274807	3.30655674	0.0009445	0.025409882	2014
Ndufb3	703.7245034	0.362379919	0.109628845	3.305516149	0.00094802	0.025419928	763
Pigk	2738.552522	0.293457821	0.088775686	3.305610302	0.0009477	0.025419928	4737
Bsdc1	2113.726262	0.265021008	0.080191264	3.304861339	0.00095023	0.025431073	2823
Ndufab1	889.9462748	0.408158857	0.1234909	3.305173567	0.00094918	0.025431073	1416
Mrpl54	530.3990934	0.444570497	0.134594795	3.303028891	0.00095647	0.025517193	604
Entpd7	1444.932432	0.198030829	0.059953357	3.303081592	0.00095629	0.025517193	5911
Fez1	618.1756294	1.306344793	0.395753171	3.300907958	0.00096373	0.025662358	1285
Hint1	1683.119262	0.319970246	0.096958675	3.300068279	0.00096661	0.025706929	636
Ankrd44	42.33431094	8.892061825	2.696656179	3.297439954	0.00097571	0.025748113	6192
Traf3ip1	35.53030113	0.754964348	0.228930128	3.297793757	0.00097448	0.025748113	2257
Stk40	1324.624331	0.260360207	0.078919019	3.299080646	0.00097002	0.025748113	3492
Rilpl2	241.1508821	0.437406716	0.132653663	3.297358732	0.00097599	0.025748113	1391
Chd4	7271.870687	0.273667584	0.082980474	3.29797568	0.00097385	0.025748113	6537
Nutf2	152.3472987	0.68550744	0.207810966	3.298706766	0.00097131	0.025748113	948
Ip6k1	184.9428693	0.522020523	0.158312861	3.297398081	0.00097585	0.025748113	1244
Ripor1	2210.976747	0.201638696	0.061169004	3.296419474	0.00097926	0.025768027	4108
Cmip	9012.774484	0.390078173	0.118330109	3.296525098	0.00097889	0.025768027	2408
Rbfox3	1788.720479	0.420330308	0.12750313	3.296627364	0.00097853	0.025768027	3084
Eef1a2	50943.62106	0.407635235	0.123680878	3.29586304	0.0009812	0.025773113	2084
2510002D2	163.564425	0.644949878	0.195685997	3.295840721	0.00098128	0.025773113	1281
Rassf4	406.1623953	0.879882854	0.26707954	3.29445997	0.00098611	0.025867972	1243
Macir	393.9691343	0.383703843	0.116543742	3.29235904	0.00099351	0.02601603	3495
Dohh	1496.621423	0.351462309	0.106800276	3.290837084	0.0009989	0.02613864	1444
Macroh2a2	547.9019691	0.362993284	0.110322218	3.290300827	0.0010008	0.026169569	2175
Aplp2	1622.962611	0.527882003	0.160520216	3.28857023	0.00100698	0.026257648	1961
Dipk1b	1659.816163	0.370519954	0.11271031	3.287365227	0.0010113	0.026306968	1622
Zbtb40	759.1230218	0.185211773	0.056341674	3.287296251	0.00101154	0.026306968	7192
Ruvbl1	648.1955079	0.283591806	0.086262824	3.287532121	0.0010107	0.026306968	1675
Fam149a	704.7593181	0.333046025	0.101344134	3.286288127	0.00101517	0.026343062	2661
Snx18	2686.01083	0.384139584	0.116941886	3.284875904	0.00102027	0.026452748	4447
Pisd	90.49267323	0.584476211	0.177949223	3.284511168	0.00102159	0.026470801	897
Gstp1	3020.081065	0.226764677	0.069061132	3.283535468	0.00102514	0.026546357	821
Insyn1	5335.400331	0.468745513	0.142775051	3.283105212	0.0010267	0.026554426	2139
H3f3a	978.6995405	0.317289662	0.096663283	3.282421728	0.0010292	0.026597587	888
Khdrbs1	3565.119862	0.258749929	0.078873078	3.280586192	0.00103592	0.026663149	3738
Nmnat1	118.6333336	0.447202513	0.136346672	3.279893137	0.00103846	0.026663149	1238
Fam216a	662.533695	0.273766905	0.083456172	3.280367388	0.00103672	0.026663149	1388
Actr8	910.0662161	0.205007897	0.06248605	3.280858645	0.00103492	0.026663149	2119
Ndufb8	3133.181476	0.319043758	0.097267187	3.280075926	0.00103779	0.026663149	680
Otud3	529.6646389	0.438374447	0.133740715	3.277793495	0.00104622	0.026802748	1668
Ddn	76.65032816	1.006441945	0.307043072	3.277852642	0.001046	0.026802748	368
Cst6	27.39800105	0.966175644	0.294769238	3.277735668	0.00104643	0.026802748	432
Bicdl1	1494.498783	0.48781425	0.148874108	3.276689651	0.00105032	0.026804938	3026
Myo5a	6492.070233	0.387557802	0.118252786	3.277367196	0.0010478	0.026804938	6480
Chst2	5861.386124	0.369335462	0.112701633	3.277108344	0.00104876	0.026804938	7328
Neu1	1272.282315	0.263928559	0.08054628	3.276731834	0.00105016	0.026804938	2474
Rab6b	2356.628389	0.754725376	0.230374872	3.276075079	0.00105261	0.026847153	716
Egfl7	134.6924209	0.555688149	0.169692773	3.274671866	0.00105785	0.026899828	1163
Irf2bp1	1929.409409	0.349170715	0.106624423	3.274772364	0.00105747	0.026899828	2734
Nap1l5	4109.591278	0.390789016	0.119369103	3.273786988	0.00106117	0.026932195	1844
Ankrd46	173.2687765	0.738542533	0.225601887	3.273654062	0.00106167	0.026932195	532
Ccdc149	702.6765957	0.402120288	0.122856286	3.273094927	0.00106377	0.026969371	3295
Ptpa	286.9631247	0.666797075	0.203743696	3.27272494	0.00106516	0.026988535	1838
Inpp4a	5539.578433	0.186797291	0.05709208	3.271859974	0.00106843	0.027022981	5691
Atp5l	908.3856644	0.283595177	0.086677343	3.271848975	0.00106847	0.027022981	544
Pcbp4	67.59686048	2.927736548	0.894819563	3.271873647	0.00106837	0.027022981	651
Aamp	2124.690049	0.273929488	0.083789702	3.269250062	0.00107833	0.027192413	1817
Stxbp5	2415.886995	0.275262874	0.084197665	3.269245933	0.00107835	0.027192413	4702
Timm17b	479.6696188	0.348268714	0.106564014	3.268164389	0.00108248	0.027280296	2381
Srp9	646.0578001	0.273110667	0.083589029	3.2673028	0.00108578	0.027290385	1367
Hmox1	557.9672258	0.383793334	0.117452341	3.267651649	0.00108444	0.027290385	1569
Mrps2	721.6651188	0.280092308	0.085819666	3.263731049	0.00109956	0.027380295	2080
Dab2ip	1511.141619	0.672496181	0.206029192	3.264082019	0.00109819	0.027380295	3972
Tpd52l2	582.1564103	0.314650228	0.096383885	3.264552237	0.00109637	0.027380295	985
Msantd3	243.2055463	0.350718236	0.107468751	3.263443859	0.00110067	0.027380295	1597
Atp6v0a2	2588.967662	0.204217488	0.062545486	3.265103546	0.00109424	0.027380295	5345
Ccdc91	1440.734385	0.256339286	0.078526863	3.26435154	0.00109715	0.027380295	2459
Babam1	1961.684632	0.358946797	0.109944439	3.264801765	0.00109541	0.027380295	1433
Dnaaf2	80.54019331	0.515626472	0.15792643	3.264978958	0.00109472	0.027380295	3390

Basp1	8679.389415	0.690487864	0.211458482	3.26535904	0.00109325	0.027380295	1855
Hdx	137.66763	0.932428016	0.285535019	3.265546967	0.00109253	0.027380295	9534
Setd7	12353.63521	0.214418539	0.065716342	3.262788728	0.00110322	0.027411454	7411
Sema4c	1288.74542	-0.665174607	0.20391041	-3.262092445	0.00110593	0.027462757	3892
Tmem108	371.9480286	0.556863459	0.1707214	3.261825749	0.00110697	0.027472493	2145
Exoc7	869.471979	0.45216611	0.138663692	3.260883244	0.00111066	0.027486185	3205
Usp20	5424.168329	0.389960075	0.119657128	3.258979073	0.00111814	0.027636249	4970
Sbf1	4899.133054	0.337059832	0.103434356	3.258683521	0.00111931	0.027648912	6190
Hgh1	165.755066	0.412041535	0.126512741	3.256917295	0.00112629	0.0277891	1993
Nedd8	905.2956519	0.452037128	0.138896644	3.254485604	0.00113598	0.027946672	824
Cul3	2596.355707	0.210888907	0.064822985	3.253304488	0.00114071	0.027999414	4709
Dlgap3	5440.6929	0.438966143	0.134955977	3.252661745	0.0011433	0.027999414	3884
Slc4a4	31.45292519	-0.872315065	0.268187421	-3.252632289	0.00114341	0.027999414	2518
Atn1	6198.659881	0.264763428	0.081397918	3.252705145	0.00114312	0.027999414	4433
Palm	193.2538327	-0.599312106	0.184244126	-3.252815273	0.00114268	0.027999414	2725
Mthfd1	1162.377422	0.349532472	0.107425816	3.253710178	0.00113908	0.027999414	3241
Pmm1	3073.281392	0.381638662	0.11730506	3.253386179	0.00114038	0.027999414	1300
Tceanc2	878.307417	0.226292862	0.069584297	3.252067973	0.00114569	0.028027585	6431
Cdkn2d	44.8857427	1.095710776	0.336977226	3.251587028	0.00114763	0.028053899	691
Ptov1	3682.845986	0.346590608	0.106624049	3.250585675	0.00115168	0.028125792	1883
Epas1	10241.02708	0.242952125	0.074742282	3.250531274	0.0011519	0.028125792	5516
Bdnf	368.390761	0.395062242	0.121567735	3.249729386	0.00115515	0.028156501	3973
Map3k9	3020.167883	0.424219771	0.130533573	3.24989014	0.0011545	0.028156501	3303
S100a6	1155.158085	0.528421346	0.162701484	3.247796726	0.00116302	0.028219641	464
Papss1	1635.631492	0.314061947	0.096682341	3.248389968	0.0011606	0.028219641	2600
Upf1	4010.487952	0.296869005	0.091398063	3.248088581	0.00116183	0.028219641	4518
Son	2597.064172	-0.225861899	0.069528845	-3.248463275	0.0011603	0.028219641	7529
Rab11b	5317.897881	0.248067811	0.076372711	3.248121048	0.0011617	0.028219641	1544
Pdzd4	1657.141807	0.37819107	0.11642447	3.248381293	0.00116064	0.028219641	3970
Gtf3c1	8638.7841	0.239722148	0.073830464	3.246927283	0.00116658	0.028288609	6961
Mlip	135.9199123	-0.734845075	0.226357625	-3.246389757	0.00116879	0.028325859	2668
Ccnl1	254.9545938	0.432283877	0.133201083	3.245348053	0.00117307	0.028383319	2169
Dennd2a	1261.217775	0.251349505	0.07747232	3.2443782	0.00117707	0.028383319	4468
Nsd3	178.2530157	0.81052893	0.249850008	3.244062055	0.00117838	0.028383319	5261
Tmem254	854.8469043	0.630952103	0.194419402	3.2453145	0.00117321	0.028383319	2147
Prr13	1128.139523	0.418071282	0.128860248	3.244377442	0.00117708	0.028383319	1224
Slc39a7	1004.259777	0.445548119	0.137321759	3.24455587	0.00117634	0.028383319	1550
Spindoc	611.6657615	0.293373234	0.09043441	3.24404432	0.00117845	0.028383319	3152
Kifbp	3595.640808	0.285664591	0.088068875	3.243649815	0.00118009	0.028388476	2472
Rps15	4859.305837	0.315207705	0.097196858	3.242982452	0.00118286	0.028422756	500
Blvrb	382.317768	0.534633139	0.164895702	3.242250304	0.0011859	0.028463573	1028
Kctd15	646.3400855	0.430339671	0.132746625	3.241812523	0.00118772	0.028470851	2407
Dnajc28	115.2938684	0.589259401	0.181781393	3.241582606	0.00118868	0.028470851	3368
Rbfa	493.7180487	0.290357722	0.089567652	3.241769961	0.0011879	0.028470851	1377
Frmpd3	2819.011101	0.308038704	0.095028743	3.24153192	0.00118889	0.028470851	7314
Tfip11	2415.69638	0.276980024	0.085452096	3.241348518	0.00118966	0.028473071	3560
Ubxn1	1662.642047	0.35742416	0.110308843	3.24021311	0.0011944	0.028522205	1033
Pik3r2	3669.557104	0.373101616	0.11516818	3.239624144	0.00119687	0.028561824	3165
Brd4	603.353291	0.510969127	0.157731066	3.239495814	0.00119741	0.028561824	507
Hspe1	305.2870812	0.33374678	0.103054621	3.238542596	0.00120142	0.028625186	774
Dynll1	3628.736471	0.417871862	0.129042425	3.238251762	0.00120265	0.028638269	2030
Psd4	58.26601046	0.54282674	0.167674078	3.237392121	0.00120628	0.028655253	11265
Tmem234	857.699331	0.270704627	0.083604499	3.237919389	0.00120405	0.028655253	1437
Magi2	31.22678093	-8.414935292	2.599333931	-3.237342917	0.00120648	0.028655253	3264
Tmf1	3102.891916	0.240123641	0.074181532	3.236973333	0.00120805	0.028655253	5732
Arhgef40	823.9704422	0.757762859	0.234050881	3.237598831	0.0012054	0.028655253	4756
Gnaz	4321.015576	0.371527814	0.1147975	3.236375471	0.00121058	0.028682015	3519
Sh3gl2	319.8783344	0.510002385	0.157621142	3.235621686	0.00121378	0.028713379	2286
Borcs5	866.5506717	0.249643713	0.077176258	3.234721678	0.00121761	0.028713379	1764
Rps5	3039.389411	0.264835523	0.081863776	3.235075842	0.0012161	0.028713379	761
Cdyl2	416.0971903	0.331624005	0.102543174	3.233993946	0.00122072	0.028713379	2411
Cox4i1	4679.167435	0.283262215	0.08756082	3.235033845	0.00121628	0.028713379	745
Sdf2	780.482712	0.323572943	0.100035161	3.234592123	0.00121817	0.028713379	1268
Ppp1r9b	15314.38501	0.439820679	0.13599212	3.23416298	0.00122	0.028713379	4508
Dip2c	5490.449388	0.289329633	0.089460427	3.234163334	0.00122	0.028713379	8023
Mrpl52	357.3606742	0.57602508	0.17806802	3.234859797	0.00121703	0.028713379	418
Mapk14	108.585452	0.898879886	0.277943285	3.234040667	0.00122052	0.028713379	1478
Luc7l	23.92941827	4.358769907	1.348075112	3.233328669	0.00122357	0.028722849	1700
Nono	3554.033924	0.242422034	0.07497121	3.233535015	0.00122268	0.028722849	2562
Gm14308	30.9620503	-8.402656002	2.59944913	-3.232475645	0.00122723	0.028754577	1458
Rplp1	4312.562638	0.32928267	0.101895818	3.231562158	0.00123116	0.028814754	499
Psma4	29.09605556	-0.872074322	0.269876911	-3.23137803	0.00123195	0.028817395	768
Rabl6	3620.774755	0.195043026	0.060414766	3.228399923	0.00124485	0.029054919	3230
Dusp11	1862.260124	-0.283359173	0.087769966	-3.228429791	0.00124472	0.029054919	6435

Pex11b	167.3065321	0.333542341	0.103404154	3.225618392	0.00125701	0.029258096	1122
Hdac4	3117.913625	0.175316999	0.054386567	3.223534958	0.00126619	0.029306633	8100
Srrm1	1368.389024	-0.268382042	0.083250358	-3.22379445	0.00126504	0.029306633	3797
Usf2	3106.284642	0.320406827	0.099375081	3.224217027	0.00126318	0.029306633	2419
Ei24	382.1046539	0.688163404	0.213403581	3.22470411	0.00126103	0.029306633	2104
Tafa2	338.2843118	0.396754861	0.123061655	3.224033201	0.00126399	0.029306633	4364
Rps14	3888.907706	0.319541112	0.099140255	3.22312175	0.00126802	0.02931327	597
Nudt22	114.3759319	0.651568431	0.202169403	3.222883489	0.00126907	0.02931327	1073
Hars	1846.923649	0.280935998	0.087175804	3.22263731	0.00127016	0.029321315	1989
Inka2	268.3854192	0.61288345	0.190209518	3.222149226	0.00127233	0.029340477	1868
Cops7a	897.0408337	0.387757921	0.120381435	3.221077411	0.0012771	0.029418383	1328
Fam8a1	2858.098428	0.367274779	0.114073309	3.219638165	0.00128353	0.029534327	3664
Brf1	1302.047578	0.222420675	0.069090719	3.219255442	0.00128524	0.029541667	2615
Oplah	892.5405036	0.523272248	0.162557346	3.219000933	0.00128638	0.029551856	3970
Ehbp1l1	252.316464	0.638587643	0.198395446	3.218761599	0.00128746	0.029560492	2852
Slu7	861.6253043	0.241081683	0.074908175	3.218362769	0.00128925	0.029569559	3625
Unc119	182.1635985	0.576005736	0.179004854	3.217821882	0.00129168	0.029609335	933
Ipo9	2061.731896	0.161084612	0.0500663	3.217425962	0.00129346	0.029634186	6247
Tysnd1	1179.469143	0.415997816	0.129354202	3.215959036	0.00130009	0.029693025	2286
Bcr	7530.788701	0.328578268	0.102152824	3.216536312	0.00129748	0.029693025	6839
Sdr39u1	1093.848497	0.261034555	0.081163382	3.216161638	0.00129918	0.029693025	1255
Cdc37	717.5484926	0.458004498	0.142461252	3.214940837	0.00130471	0.029763087	1144
Pbx2	522.803052	0.637876667	0.198401559	3.215078907	0.00130409	0.029763087	1990
Epb41l2	189.0217515	-0.582370359	0.181168429	-3.214524527	0.00130661	0.029790229	4345
Bcl9	1808.619384	0.18812763	0.05852858	3.214286591	0.00130769	0.029798881	6102
Pus7	183.3618806	0.431314437	0.134208059	3.213774484	0.00131003	0.029836018	3266
Ecd	1335.201986	0.230203193	0.071657914	3.212529933	0.00131571	0.02988521	3132
Nrip1	652.2644259	0.538886285	0.167807427	3.211337514	0.00132119	0.029993416	4529
Sirt6	654.3479944	0.356094879	0.110895768	3.211077261	0.00132238	0.030004505	1891
Zfp503	1085.958487	0.437183187	0.136163136	3.2107309	0.00132398	0.0300246	4216
Plrg1	822.4873899	0.240510365	0.074931991	3.209715396	0.00132867	0.030082529	1851
Hectd1	6949.636578	0.17293991	0.053879345	3.20976266	0.00132845	0.030082529	8988
Rtl8b	5344.366632	0.317116237	0.09882316	3.208926302	0.00133232	0.030132982	1232
Cdc42bpa	4337.660842	0.3275109	0.102119411	3.207136586	0.00134063	0.030231708	6704
Tfeb	73.0078085	-0.640034647	0.199570197	-3.207065261	0.00134097	0.030231708	1974
Rnf40	1211.574102	0.404232595	0.126066247	3.206509312	0.00134356	0.030241876	5279
Susd4	298.1615687	0.44383432	0.138427119	3.206267113	0.00134469	0.030246095	1898
Ccdc32	515.8540503	0.278861529	0.08698085	3.206010637	0.00134589	0.030246095	1870
Nudt9	1279.092629	0.337641212	0.105328866	3.205590488	0.00134786	0.030258166	1488
Sobp	1600.555946	0.340539599	0.106229298	3.205703203	0.00134733	0.030258166	4980
G0s2	278.3525239	0.409776728	0.127844664	3.205270495	0.00134936	0.030259739	871
Fkbp5	90.02882682	-0.722781284	0.225516909	-3.204998193	0.00135063	0.030264791	2431
Apoe	67798.34856	0.554701656	0.173112676	3.204280977	0.001354	0.030283739	1104
Capn10	291.6696074	0.30228408	0.094358251	3.203578688	0.00135731	0.030338229	1268
Dclk1	1543.978302	0.35482747	0.110791432	3.202661662	0.00136164	0.03036504	7847
Mettl14	77.90831178	-0.769006624	0.240111067	-3.202712123	0.0013614	0.03036504	7197
Ppp5c	2841.691904	0.242437517	0.075707438	3.202294544	0.00136338	0.03036504	2072
Bcap31	2493.33614	0.250818616	0.078321927	3.202406069	0.00136285	0.03036504	1219
Plekhg3	256.8960851	0.888168004	0.27737454	3.202053095	0.00136452	0.030373531	5138
Usp7	2235.062676	0.252674044	0.078931225	3.201192486	0.0013686	0.030417421	4003
Slc25a19	25.4776132	-1.03172126	0.32242788	-3.199851262	0.00137499	0.030511269	826
Ubac1	1421.284807	0.360413169	0.112695633	3.198111214	0.00138331	0.030615749	1852
Tbcb	1152.529033	0.324897269	0.101588314	3.198175617	0.001383	0.030615749	1416
H2ac13	70.02732832	0.985092642	0.30800395	3.198311714	0.00138235	0.030615749	393
Smad1	156.2612763	-0.444549919	0.139033725	-3.197425075	0.0013866	0.030656626	3775
Mzt2	287.9114496	0.30668021	0.095931811	3.196856261	0.00138934	0.030701109	1035
Nudt2	204.7385007	0.323222688	0.101111249	3.196703526	0.00139008	0.030701345	845
Setd5	411.3304274	1.459034446	0.456552046	3.195768057	0.00139459	0.030784994	6527
Ranbp3	1228.693451	0.264779736	0.082858597	3.195561413	0.00139559	0.030790994	2373
Otulin	449.1680393	0.284803924	0.089167495	3.194033014	0.001403	0.030906164	1491
C1qc	782.3454289	0.327208339	0.102472222	3.193141841	0.00140734	0.030962124	1203
Lrp11	239.0789276	1.363407361	0.426951669	3.193352922	0.00140631	0.030962124	1492
H1f10	1874.64418	0.617990172	0.193625822	3.1916723	0.00141452	0.031062885	1217
Mrpl20	333.3905686	0.377153868	0.118184369	3.191233089	0.00141667	0.03109402	764
Atp5j	646.8126814	0.409808391	0.128459203	3.190183203	0.00142183	0.031158749	809
Atp6v1g1	1869.853626	0.33501051	0.105026569	3.189769134	0.00142387	0.03116272	1135
Tagln3	3347.269012	0.29135389	0.091342156	3.189697966	0.00142422	0.03116272	1240
Enah	2230.042621	0.394754087	0.123775713	3.189269353	0.00142633	0.031176741	4231
Cox11	210.6569598	0.421804328	0.132256214	3.189296869	0.00142619	0.031176741	1224
Arhgef18	2548.527912	0.282110149	0.088462748	3.189027625	0.00142752	0.031186723	5271
Hes6	380.0001108	0.438719879	0.137615182	3.188019472	0.00143251	0.031230774	1335
Gm45902	189.3228113	-0.367210623	0.115195886	-3.187706042	0.00143406	0.031230774	4314
Aldh2	360.9824128	0.563108763	0.176662443	3.187484293	0.00143516	0.031230774	802
Git1	5764.486965	0.409456013	0.128428634	3.188198763	0.00143162	0.031230774	3512

Cox7a2l	2850.529227	0.201383918	0.063194497	3.186731897	0.0014389	0.031277127	1076
Pfkm	341.9424327	0.802756141	0.251943239	3.186257917	0.00144126	0.031309147	2816
Nudt5	221.0524914	0.325062837	0.102053217	3.1852287	0.0014464	0.031383746	1590
Tmem59l	5166.224539	0.282093406	0.088559349	3.185359991	0.00144574	0.031383746	1491
Ubqln1	4517.409588	0.397751652	0.124890662	3.184798984	0.00144855	0.031398155	3561
Fbxl16	7292.837603	0.320790184	0.100728695	3.18469514	0.00144907	0.031398155	3489
Asphd2	127.7236727	0.552505403	0.173618639	3.182293131	0.00146114	0.031611213	717
Mydgf	543.776472	0.346225225	0.108809185	3.181948532	0.00146288	0.031632685	1710
Sox3	69.39681097	0.717134912	0.225399278	3.181620271	0.00146454	0.03165239	2065
Strip1	1710.163265	0.209699236	0.065913272	3.181441751	0.00146544	0.031655745	3215
Ache	1475.951942	0.248583942	0.07814276	3.181151307	0.00146691	0.031671341	2176
Chmp2a	93.47079056	3.942217871	1.239826539	3.179652754	0.00147452	0.031818447	606
Rgl1	568.3951721	0.409313803	0.128803303	3.177820698	0.00148387	0.031955928	3037
Isyna1	844.3799811	0.312799288	0.098459093	3.176946672	0.00148834	0.032003554	1836
Vgf	6521.166361	0.599721301	0.188818423	3.176180007	0.00149228	0.032055697	2553
Fam32a	1828.308057	0.298771059	0.094083221	3.175604066	0.00149525	0.032103113	2564
Dnajc4	107.8680508	0.756833852	0.238383386	3.174859895	0.00149909	0.032152945	840
Lin37	353.7963004	0.375068854	0.118185897	3.173549997	0.00150587	0.032233071	998
Orai2	1894.559434	0.359605845	0.113334738	3.172953431	0.00150897	0.03226675	3908
Terf2	135.7150803	-0.428572114	0.135093494	-3.172411204	0.00151179	0.032310744	2471
Parl	412.6081544	0.245862076	0.077504212	3.17224148	0.00151267	0.032313323	1348
Dnajb5	182.2455456	0.615855918	0.194322702	3.169243276	0.00152836	0.03251729	3093
Sorl1	17755.8374	0.192097618	0.06061782	3.168995832	0.00152967	0.032528641	10715
Nipa2	43.53261088	-0.725329186	0.228917336	-3.168520118	0.00153217	0.032561343	3925
Hp1bp3	737.7191355	0.519473467	0.164051781	3.166521343	0.00154274	0.032667366	3113
Phf1	1327.79667	0.307705885	0.097170763	3.166650898	0.00154205	0.032667366	2522
Cabp1	7855.457629	0.572364062	0.180792972	3.165853488	0.00154629	0.032701412	1621
Prr7	362.7318008	0.474073312	0.149769058	3.165362172	0.0015489	0.032740333	1359
Lrrc59	4752.886006	0.31752468	0.100359359	3.163877119	0.00155683	0.032834944	2837
Adcy9	1650.692824	0.427141162	0.135036378	3.163156239	0.00156069	0.032882727	4974
Ttc3	85.25069956	0.649717771	0.205406954	3.163075822	0.00156112	0.032882727	599
Fkbp8	8977.945073	0.391386508	0.123759122	3.162486131	0.00156428	0.032884871	1698
Cpsf6	231.2993944	0.507744952	0.160550472	3.162525433	0.00156407	0.032884871	2200
Bysl	845.4916774	0.284013166	0.08981432	3.162225887	0.00156568	0.032897941	3854
Fam184b	372.4489594	0.4120492	0.130313855	3.161975372	0.00156703	0.032909923	4236
Lmo4	1076.898158	0.258485693	0.081752943	3.161790675	0.00156802	0.032914476	1669
Zfp251	908.7778492	0.345260899	0.109231717	3.160811805	0.0015733	0.032992569	4036
Psmd7	1401.904887	0.20206811	0.063943095	3.160123998	0.00157702	0.033029894	1581
Mcrs1	1496.736403	0.354489448	0.112172153	3.16022683	0.00157646	0.033029894	1916
Fbxo44	61.76170079	0.656739831	0.207845884	3.159744221	0.00157908	0.033048216	793
Itm2c	11433.76522	0.292859024	0.09272095	3.158498954	0.00158584	0.033102812	2230
Lin7b	361.4051618	0.441193836	0.13968699	3.158446152	0.00158613	0.033102812	735
Cib2	434.4240691	0.315467103	0.099881932	3.158400089	0.00158638	0.033102812	1433
Socs5	414.5054828	0.366494351	0.116011802	3.159112642	0.0015825	0.033102812	978
Pitpnm1	3741.935385	0.324625119	0.102771103	3.158719817	0.00158464	0.033102812	4206
Raly	688.8518603	0.675088538	0.213861014	3.156669495	0.00159582	0.033214968	1100
Atp6v0e	150.3570923	-0.662974955	0.210010969	-3.156858708	0.00159479	0.033214968	2812
Cotl1	1978.784922	0.31448447	0.099655476	3.155716897	0.00160104	0.03322868	1596
Asphd1	226.807698	0.345431607	0.109507875	3.154399688	0.00160829	0.033242104	1647
Rnf122	96.10705583	-0.482855747	0.153071836	-3.154438847	0.00160807	0.033242104	3582
Gatad2a	2695.388841	0.247219823	0.078371855	3.154446486	0.00160803	0.033242104	3363
Rfng	1354.301708	0.35999898	0.114129061	3.154314739	0.00160876	0.033242104	2118
Pde12	930.9781266	0.23986774	0.076032626	3.154800167	0.00160608	0.033242104	7725
Nefl	176359.5483	0.489712835	0.155210945	3.155143705	0.00160419	0.033242104	3380
Tsc22d1	4077.776586	0.259763835	0.082351743	3.154321013	0.00160872	0.033242104	1762
Add1	550.8757071	0.533614575	0.169211817	3.153530203	0.00161309	0.033315327	745
Smyd4	409.3220764	0.225499018	0.071535469	3.152268669	0.00162007	0.0333782	3517
Prrc2b	23624.31751	0.266896375	0.084677286	3.151924077	0.00162198	0.033382609	10671
Rab3ip	664.6045014	0.345834161	0.109724008	3.151854983	0.00162237	0.033382609	1844
Rtca	1180.70334	0.372056284	0.118086172	3.150718475	0.00162869	0.033441937	1594
Rph3a	2175.411729	1.156317169	0.366961196	3.151061147	0.00162678	0.033441937	2621
Sun1	483.4673798	0.354915277	0.112640347	3.15087165	0.00162784	0.033441937	2608
Nme1	1290.502259	0.272844751	0.086595379	3.15080037	0.00162824	0.033441937	3181
Map2k2	953.7961627	0.332303141	0.105475614	3.15052103	0.0016298	0.033448328	2405
Tonsl	352.2623636	0.253523169	0.080477046	3.150254405	0.00163128	0.03346265	4235
Hsf1	985.2236556	0.318821719	0.101222696	3.149705857	0.00163435	0.033493084	2084
Dhrs7b	368.40016	0.339462188	0.107786272	3.149400955	0.00163606	0.033511832	1553
Lrfn3	1101.142037	0.287728511	0.091393388	3.148242085	0.00164256	0.033570409	2886
Chga	8991.6886	0.442050385	0.140414384	3.148184476	0.00164288	0.033570409	1883
Smim10l2a	395.416045	0.357400712	0.113546265	3.147621898	0.00164605	0.033618853	2393
Ubc	21918.3198	0.235479783	0.074828153	3.146941026	0.00164988	0.033680988	693
Specc1l	1274.016416	0.322384647	0.102453367	3.146647667	0.00165154	0.033684537	4725
Mtdh	145.6787456	0.518629636	0.164840096	3.146258998	0.00165374	0.03369465	1593
Rpn1	3263.821247	0.301072647	0.095716112	3.145475095	0.00165817	0.033749792	3633

Diablo	163.1295532	-0.464309717	0.147633899	-3.145007474	0.00166083	0.033774115	2588
Syk	149.6432236	0.539705626	0.171622861	3.144718725	0.00166247	0.03377502	5363
Sarm1	2489.994294	0.441982793	0.140583747	3.143911038	0.00166706	0.033803496	4083
Cab39	6268.368195	0.259619048	0.082608936	3.142747733	0.0016737	0.033876931	3805
Dnm1	136.7063389	2.548256698	0.810989747	3.142156492	0.00167708	0.033876931	641
Drosha	3398.904011	0.198520095	0.063175403	3.142363736	0.0016759	0.033876931	4519
Pstpip2	747.9173496	0.301631222	0.095993836	3.14219367	0.00167687	0.033876931	2267
Larp1b	819.7522836	0.506097756	0.161147399	3.140589056	0.00168608	0.034042502	2497
Psmb6	2006.008815	0.350638686	0.111655239	3.140369324	0.00168735	0.03405181	798
Trmt112	595.3923954	0.422935621	0.134683654	3.140214931	0.00168824	0.034053531	985
Mycbp2	10236.00604	0.200218195	0.063775444	3.139424544	0.0016928	0.034113023	14620
Osbpl1a	875.7668617	0.512887399	0.163402317	3.138801264	0.00169641	0.034169397	2674
Nomo1	5460.244107	0.248014337	0.079030361	3.138215928	0.0016998	0.034192393	4257
Zcchc18	611.8253855	0.388803408	0.123926213	3.137378267	0.00170466	0.034270474	2045
Rbbp6	2097.387417	0.141992894	0.045264656	3.136948451	0.00170716	0.034300224	6184
Recql5	476.4220621	0.258781275	0.082517084	3.136093318	0.00171215	0.034355734	3971
Taok3	921.2310281	0.301325543	0.096108269	3.13527178	0.00171695	0.034384892	2172
Rpl31	1105.831512	0.389680924	0.124322998	3.134423477	0.00172192	0.034411669	1049
Osbp	3462.409881	0.167972291	0.053590489	3.13436759	0.00172225	0.034411669	4488
Psmb5	1989.441077	0.338307046	0.107973779	3.133233368	0.00172892	0.034479845	899
Ccdc13	66.13768382	-0.752936802	0.240452264	-3.131335882	0.00174013	0.034638162	969
Itga6	176.0635851	0.929962344	0.297150371	3.129601831	0.00175043	0.034721624	3393
Dnttip2	505.9914692	0.214599121	0.068567435	3.129752793	0.00174954	0.034721624	2429
Uqcrc1	7826.515836	0.290553255	0.092828588	3.12999757	0.00174808	0.034721624	1653
Apba3	990.0393714	0.347811375	0.111120367	3.130041622	0.00174782	0.034721624	2106
Txnrd2	26.51720743	-0.89941722	0.287387611	-3.129631158	0.00175026	0.034721624	2874
Adrm1	3645.738766	0.397623095	0.127086492	3.12875971	0.00175546	0.034745223	1535
Mrpl13	240.3362829	0.282037111	0.09015373	3.128402005	0.0017576	0.034757058	1223
Mrm3	227.7750403	0.268347857	0.085783177	3.128210741	0.00175874	0.034763448	1510
Tns1	1593.645907	0.686164349	0.219386596	3.127649369	0.0017621	0.034787348	2813
Ppp1r37	4847.753463	0.351764124	0.11246766	3.127691322	0.00176185	0.034787348	3816
Sorcs2	1578.325332	0.224163347	0.071713319	3.125825871	0.00177307	0.034883737	5707
Kcmf1	2765.417399	0.363733295	0.116359622	3.125940828	0.00177237	0.034883737	3251
Ankrd13c	314.4496036	1.10048519	0.352226263	3.124370056	0.00178186	0.034917158	1735
Mkrn1	334.3839131	0.315189679	0.100884036	3.124277067	0.00178243	0.034917158	1485
Sap30l	3586.491691	0.593588876	0.189984276	3.124410554	0.00178162	0.034917158	1151
Ube2o	14110.76283	0.345198298	0.110476261	3.124637759	0.00178024	0.034917158	5205
Akt1	8339.976815	0.298656868	0.095583479	3.12456577	0.00178068	0.034917158	2690
Plac9	293.8615211	0.849338599	0.271852178	3.124266297	0.00178249	0.034917158	1555
Safb2	206.3974128	-0.602772619	0.192946242	-3.124044369	0.00178384	0.034917158	2636
Polr2i	98.95368263	0.400277505	0.128187271	3.122599467	0.00179262	0.035072798	819
Smox	132.0624761	-0.588338217	0.188439774	-3.122155176	0.00179532	0.035109549	2299
Sympk	6028.695717	0.323078397	0.103487741	3.121900168	0.00179688	0.03512375	4116
Lzts3	2049.597339	0.209039431	0.066975215	3.121146107	0.00180149	0.035165112	4201
Ddx24	5171.371765	0.240704456	0.077171887	3.11906919	0.00181423	0.035342203	2850
Nefm	106101.9312	0.533944952	0.171214962	3.118564794	0.00181734	0.035376751	3334
Maz	1619.618313	0.429506008	0.137765549	3.117659017	0.00182294	0.035469337	2347
Tmsb10	1813.484924	0.516106462	0.165552368	3.117481605	0.00182403	0.035474389	570
Med8	74.96204339	-0.54134829	0.173733231	-3.115974347	0.00183338	0.035628704	1743
Acox3	140.9765376	0.585089938	0.18780505	3.115411098	0.00183689	0.035658855	2956
BC004004	32.75796159	-0.621389087	0.199555669	-3.113863367	0.00184655	0.035797194	1396
Smn1	253.4106096	0.267645536	0.086024551	3.111269189	0.00186285	0.035997819	1222
Msrb1	70.19996501	-0.559688279	0.17989014	-3.111278247	0.00186279	0.035997819	1211
Rwdd4a	526.6538647	0.260097691	0.083617341	3.110571171	0.00186726	0.036022027	2921
Snx1	1771.554585	0.182207964	0.058573819	3.110740728	0.00186619	0.036022027	2073
Rnf187	13893.06979	0.387797452	0.124668866	3.11061986	0.00186695	0.036022027	1949
Mrpl2	412.900878	0.330441226	0.106233024	3.110532046	0.00186751	0.036022027	1031
Atp5h	1884.801077	0.233455731	0.075057035	3.110377751	0.00186848	0.036024439	625
Col24a1	755.0506889	-0.468164968	0.150531793	-3.110073682	0.00187041	0.036045131	7143
Rpl37a	1047.241039	0.284089023	0.091389384	3.108556042	0.00188004	0.036132096	558
Itga3	1497.121008	0.334740158	0.107675813	3.108777636	0.00187863	0.036132096	4697
Atg101	563.4653693	0.332617245	0.107012397	3.108212258	0.00188223	0.036145195	1270
Tomm34	3070.686694	0.363471759	0.117011765	3.106283883	0.00189455	0.036312015	1923
Cbx7	107.6500199	1.004287235	0.323305109	3.106314155	0.00189435	0.036312015	710
Tma7	874.1187541	0.336483535	0.108341375	3.105771329	0.00189783	0.036358553	944
Tcf20	5580.570921	0.251087985	0.08089659	3.103814223	0.00191043	0.036550294	7323
Qsox1	1764.885539	0.279670315	0.090116723	3.103423058	0.00191296	0.036565598	3348
Fbxo41	1339.035824	0.368248557	0.118670133	3.103127521	0.00191487	0.036569115	6357
Atxn7l2	233.7289599	0.525484284	0.169396465	3.102097105	0.00192155	0.036572135	2387
Rps17	682.9789041	0.304627447	0.098209015	3.101827751	0.0019233	0.036572135	494
Kbtbd11	2824.851249	0.36749355	0.118479549	3.101746702	0.00192383	0.036572135	6973
Ddx28	354.7793043	0.371800515	0.119839837	3.102478485	0.00191908	0.036572135	2262
Mfsd12	1929.365376	0.321818956	0.103734252	3.102340354	0.00191997	0.036572135	3971
Satb1	785.9470321	0.349766011	0.112739655	3.102422216	0.00191944	0.036572135	5993

Ndfip1	1976.270948	0.951537457	0.306724752	3.102251937	0.00192054	0.036572135	1392
Atxn7l3	4589.817731	0.296105962	0.09548025	3.101227339	0.0019272	0.036606476	3688
Rps21	756.6492115	0.293902433	0.0948079	3.099978308	0.00193535	0.036744711	394
Guk1	1159.234755	0.31454044	0.101481768	3.099477332	0.00193862	0.036772652	1078
Kat2a	1852.11561	0.424463849	0.136958182	3.099222279	0.00194029	0.036772652	3043
Chkb	599.5006838	0.458385963	0.147900366	3.099288899	0.00193986	0.036772652	1707
Tbx3	952.4708129	0.597537437	0.192889738	3.097818692	0.00194951	0.036847802	4856
Large1	952.5216743	0.446972854	0.144260221	3.098379114	0.00194582	0.036847802	3669
Naa38	302.7281318	0.379904151	0.122649745	3.097471994	0.00195179	0.036847802	671
Mien1	1045.339203	0.32550418	0.105057595	3.098340303	0.00194608	0.036847802	747
Hid1	3622.392951	0.230420967	0.074388449	3.097536916	0.00195136	0.036847802	3236
Cant1	922.7963388	0.467023551	0.150759598	3.097803106	0.00194961	0.036847802	2873
Vps26b	3430.701597	0.228208486	0.073707044	3.096155716	0.00196047	0.036973051	3575
Tpgs1	1222.83083	0.532444362	0.171987762	3.095827028	0.00196265	0.036997578	1101
Aifm1	148.4446788	0.535375806	0.172946136	3.095621673	0.00196401	0.037006731	2221
Pcbp1	7421.545597	0.389019754	0.125692516	3.095011278	0.00196805	0.037066479	1830
Gtf2ird2	590.9051463	0.193488732	0.062519031	3.094877356	0.00196894	0.03706673	3482
Tspan13	2793.421784	0.30765861	0.099448618	3.093643896	0.00197715	0.037176536	1932
Psmd4	1926.491318	0.300378066	0.097104884	3.093336336	0.0019792	0.037193616	1332
Gid4	2973.869469	0.208515755	0.067436267	3.092041769	0.00198785	0.03729179	4283
Pfkp	2806.656829	0.435996215	0.141000249	3.092166274	0.00198702	0.03729179	2679
Camk2n2	3626.044581	0.43294058	0.140018347	3.092027512	0.00198794	0.03729179	1291
Mgll	808.6048182	0.576657613	0.18651714	3.091713792	0.00199005	0.037314689	3867
Txn2	1875.249273	0.22660229	0.073313774	3.090855605	0.00199581	0.037358245	1298
Rab18	463.5020331	0.301113242	0.097421107	3.090841922	0.0019959	0.037358245	1184
Ppp1r35	382.3477025	0.524286542	0.169653691	3.090333833	0.00199932	0.037405682	956
Tsnax	1962.433716	0.220464708	0.071363496	3.089320448	0.00200615	0.037483828	2391
Zfr2	2262.667336	0.475346477	0.15388799	3.088912111	0.00200891	0.037510776	3349
Mrpl27	483.3333645	0.331229737	0.107234178	3.088844843	0.00200936	0.037510776	756
Gmpr	1193.116082	0.317713577	0.102867317	3.088576493	0.00201118	0.03752536	1580
Pex6	1738.998298	0.342593672	0.110926755	3.088467455	0.00201192	0.03752536	3200
Cdk5r1	2769.354509	0.279717004	0.090587531	3.087809108	0.00201638	0.037542432	4162
Flna	2845.735748	0.450264082	0.145814087	3.087932664	0.00201554	0.037542432	8312
Echs1	870.0416176	0.208005261	0.06737533	3.087261462	0.0020201	0.037546631	1557
Rps10	713.8652392	0.357317036	0.115732841	3.087429928	0.00201895	0.037546631	498
Fbxl19	2237.440983	0.429408769	0.139109508	3.086839812	0.00202297	0.037561262	3410
Ift20	327.3702393	0.39027959	0.126429155	3.086942963	0.00202226	0.037561262	1141
Ppp1r3f	843.5712146	0.254243914	0.082366308	3.0867465	0.0020236	0.037561262	3066
Axin1	735.3681074	0.28083127	0.090989303	3.08642072	0.00202582	0.037585967	3777
2210016L2	2760.862236	0.385712313	0.124977908	3.086243966	0.00202702	0.037591845	2607
Sh2d3c	1191.131077	0.588643935	0.190767659	3.085658951	0.00203102	0.037632911	3107
Pds5a	1538.344179	0.200014632	0.064844984	3.084504324	0.00203892	0.037663128	4146
Dhps	710.6336207	0.407747609	0.132185896	3.084652902	0.0020379	0.037663128	1333
Slc1a4	1999.92105	0.393078584	0.127452772	3.08411169	0.00204161	0.037663128	3872
Jag2	110.9861708	0.667091798	0.216267495	3.084568019	0.00203848	0.037663128	742
Wbp4	1217.032151	0.326671624	0.105924933	3.08399179	0.00204243	0.037663128	3704
Taf2	933.7668843	0.124899811	0.04049039	3.084677918	0.00203773	0.037663128	5031
Arxes2	944.0074295	0.405043184	0.131326734	3.084240138	0.00204073	0.037663128	1532
Zrsr1	2383.594776	0.251718281	0.08163672	3.083395335	0.00204653	0.037686774	4492
Lrrc8a	8268.205846	0.279477194	0.090672211	3.082280552	0.00205421	0.037780815	4301
Svop	2493.018639	0.298885469	0.096985002	3.081769985	0.00205774	0.037780815	3379
Taf10	401.0317495	0.21555955	0.06994543	3.081824666	0.00205736	0.037780815	4281
R3hdm4	2069.358786	0.239736004	0.077823244	3.080519304	0.0020664	0.037890555	1898
Nt5c	553.5385984	0.374014182	0.121421903	3.080285946	0.00206802	0.037903842	858
Rpl35a	90.97200877	0.525506684	0.170614139	3.080088716	0.00206939	0.03791254	510
Yars	2601.093621	0.209779254	0.068118233	3.079634405	0.00207255	0.03793758	2909
Dmwd	1088.34882	0.490795433	0.159365583	3.079682726	0.00207221	0.03793758	2494
Rhbdl1	214.220463	0.458143485	0.148785598	3.079219308	0.00207544	0.037941272	1711
Atp1b1	43455.98995	0.314507795	0.102148143	3.078937977	0.0020774	0.037960728	2580
Stmn3	17125.52572	0.356409295	0.115765453	3.078718963	0.00207893	0.037972255	1136
Nub1	2342.027667	0.278434465	0.090470915	3.077613009	0.00208666	0.038097012	3269
Terf2ip	1264.424555	0.314707709	0.102265887	3.077347872	0.00208851	0.038100418	3560
Lrrc58	10128.81554	0.144238931	0.046871463	3.077329433	0.00208864	0.038100418	8604
Ipo4	3637.338585	0.269795333	0.08769301	3.076588804	0.00209384	0.038162315	3846
Virma	230.9703205	-0.499967508	0.162524159	-3.076265781	0.00209611	0.038170823	3954
Kcng2	401.2650634	0.50624783	0.16460146	3.075597441	0.00210081	0.038223597	2813
Ncbp1	1424.127722	0.181801122	0.059116246	3.075315744	0.0021028	0.038243286	3010
Msh6	695.0534496	0.226254787	0.073578139	3.075027306	0.00210483	0.038263854	4251
Pebp1	6865.198231	0.305576166	0.099393553	3.074406309	0.00210922	0.038327145	1240
Zfp82	48.94319154	0.74323047	0.241771377	3.074104469	0.00211136	0.038349486	2181
Pold1	435.2627048	0.314840712	0.102431902	3.073658749	0.00211451	0.038371818	3454
Ppp2r2d	546.493295	0.332471164	0.108196367	3.072849603	0.00212025	0.038445139	1938
Bphl	250.9534478	0.363193236	0.118216483	3.072272385	0.00212436	0.0384866	1253
Cbln4	24.98239377	-2.371376215	0.772259723	-3.070697778	0.00213559	0.038525278	2902

Rap1gap	299.9798293	0.483509876	0.15744362	3.071003285	0.00213341	0.038525278	4140
Il16	213.3720343	-0.861752122	0.280558146	-3.071563365	0.00212941	0.038525278	3183
Fgfr1	7436.251755	0.382972012	0.124716442	3.07074196	0.00213528	0.038525278	4046
Sptb	19101.11567	0.248667719	0.080979242	3.070758803	0.00213516	0.038525278	10394
D17H6S53	322.7817821	0.314391217	0.102370466	3.071112491	0.00213263	0.038525278	2142
Set	3045.379694	0.272594424	0.088793242	3.069990668	0.00214066	0.038573372	2834
Hdgf	4631.374666	0.2326717	0.075804658	3.069358865	0.00214519	0.038599697	2245
Rgs19	32.99373041	0.696894464	0.227106015	3.068586549	0.00215074	0.038633937	779
Tpi1	9203.695861	0.279580826	0.091123384	3.06815676	0.00215384	0.038642576	1601
Kif3c	2199.914228	0.438099977	0.142801884	3.067886529	0.00215579	0.038642576	4144
Zmat2	1261.165047	0.258196315	0.084159684	3.067933505	0.00215545	0.038642576	2310
Pank1	909.7447427	0.406149777	0.132376732	3.068135697	0.00215399	0.038642576	7414
Aco1	1195.641407	0.202820441	0.06612417	3.06726634	0.00216026	0.038661422	3832
Tomm7	292.6763566	0.283819769	0.092532951	3.0672292	0.00216053	0.038661422	1125
Gfod2	633.621162	0.207541624	0.067659125	3.067459491	0.00215887	0.038661422	4352
Rtn4r	676.4318184	0.360703521	0.117598194	3.067253899	0.00216035	0.038661422	1938
Sh3kbp1	749.9952236	0.416738994	0.135882478	3.066907524	0.00216286	0.038661422	7317
Mthfr	71.16328616	0.919633432	0.299994529	3.065500674	0.00217306	0.038804361	2652
Mif	2389.007344	0.336497824	0.109802467	3.064574346	0.0021798	0.038875547	544
Tspan5	354.0129415	0.461039105	0.150455336	3.064292142	0.00218186	0.038895856	6653
Fdx1	139.681965	0.387585138	0.126531632	3.063148177	0.00219022	0.038979212	875
Gde1	3214.919745	0.264963628	0.086537716	3.061828299	0.0021999	0.039069378	1582
Rnf14	293.7664523	0.508709711	0.166144281	3.061855074	0.0021997	0.039069378	2410
Epb41l3	1888.814737	0.533121247	0.174129726	3.061632597	0.00220134	0.039078536	666
Atp9a	5312.891021	0.646637529	0.211248449	3.061028533	0.00220578	0.039098773	3456
Zyx	77.63769574	0.825540888	0.269691743	3.06105362	0.0022056	0.039098773	525
Snph	553.9680257	0.347612329	0.113630124	3.059156476	0.00221961	0.039271268	845
Emsy	271.6402502	0.342518539	0.111994344	3.05835568	0.00222555	0.039327073	5079
Tut1	644.8251825	0.183895816	0.060128896	3.058360091	0.00222552	0.039327073	2753
Pcid2	1040.105541	0.293885283	0.096107564	3.057878822	0.0022291	0.039333453	2860
Thop1	2041.026519	0.428712957	0.140190944	3.058064546	0.00222772	0.039333453	2832
Kremen1	91.2440208	-0.990497097	0.323928028	-3.057769042	0.00222991	0.039333453	3183
Snrnp70	168.5581692	-0.422002664	0.138038855	-3.057129568	0.00223468	0.039356893	620
Commd3	652.6018985	0.20208088	0.066113661	3.056567705	0.00223887	0.039414337	935
Supt16	2172.351501	0.169366234	0.055414258	3.056365604	0.00224038	0.039419274	4673
Tbc1d9	2883.428694	0.366422995	0.119912127	3.055762621	0.00224489	0.039438323	4631
Ppp1r12a	1377.376406	0.259272004	0.084845545	3.055811628	0.00224452	0.039438323	3355
Os9	1552.67191	0.328109801	0.107373772	3.055772317	0.00224482	0.039438323	2627
Caprin1	3790.042389	0.388927576	0.127301608	3.055166251	0.00224936	0.039461186	6169
Slc37a4	67.19409575	0.538276668	0.176185801	3.055164862	0.00224937	0.039461186	891
Scaf4	1064.623352	0.325024409	0.106387782	3.05509151	0.00224992	0.039461186	4266
Syn2	3846.141463	0.284431736	0.093113731	3.054670146	0.00225308	0.039500301	3806
Cul4a	2015.868809	0.243464059	0.079706854	3.054493385	0.00225441	0.039507226	3728
Gramd1b	722.438297	0.389832298	0.127650771	3.053896944	0.0022589	0.039526665	2781
Csnk1e	274.0488171	-0.328088257	0.107434322	-3.053849568	0.00225925	0.039526665	3174
Jtb	349.2089902	0.261868738	0.085793727	3.052306357	0.0022709	0.03958125	1101
Pfn1	4516.807178	0.426961	0.139838415	3.053245417	0.00226381	0.03958125	861
Hdac7	29.41965264	-1.276614614	0.41826881	-3.05213916	0.00227217	0.03958125	3767
Crkl	2737.516696	0.203741614	0.066744134	3.052577082	0.00226886	0.03958125	5042
Ccdc85b	2053.979316	0.431153719	0.141265791	3.0520745	0.00227266	0.03958125	4715
Rnaseh2c	361.4100993	0.378586967	0.124001504	3.053083672	0.00226503	0.03958125	1126
Map3k10	5987.414697	0.356709394	0.116928492	3.050662742	0.00228337	0.039735125	3682
Atp5b	23273.20487	0.263834253	0.086500173	3.05010086	0.00228765	0.039776843	1916
Tmem248	2305.731471	0.214175118	0.070258535	3.048385782	0.00230074	0.039928529	3570
Acsf3	429.5755287	0.350210059	0.114897777	3.048014233	0.00230359	0.039939273	2206
Cyb561a3	162.0389669	0.538365612	0.176624125	3.048086501	0.00230304	0.039939273	2634
Adprh	2848.23046	0.324293873	0.106426834	3.047106265	0.00231056	0.040027334	2966
Dpp10	452.1893436	0.371240989	0.121843878	3.046857964	0.00231247	0.040044027	15297
Prnp	10836.09793	0.219780117	0.07216058	3.045708837	0.00232133	0.040143507	2184
Selenom	3639.600826	0.423470864	0.139047874	3.045504059	0.00232291	0.040143507	707
Gfod1	4624.421059	0.297868997	0.097808369	3.045434651	0.00232344	0.040143507	6928
Zscan25	478.637577	0.312906529	0.10277249	3.044652608	0.00232949	0.040172024	1796
Pth1r	121.5037022	0.954828058	0.313604112	3.0446924	0.00232918	0.040172024	2219
Ccar1	620.1637003	0.337909707	0.110988444	3.044548559	0.0023303	0.040172024	3862
Tbca	678.0045814	0.306512228	0.100700931	3.043787421	0.0023362	0.040201056	559
Acox1	1762.369863	0.3360497	0.110425482	3.043226004	0.00234057	0.040234337	3538
Tbc1d10b	4873.399386	0.323712222	0.106420709	3.04181607	0.00235156	0.040308706	3614
Lage3	222.5930738	0.344560878	0.113264595	3.042088119	0.00234943	0.040308706	761
Acot7	9365.608132	0.371498628	0.12216051	3.041069723	0.00235739	0.040376066	1457
Agfg2	805.3168868	0.400225401	0.131620355	3.040756132	0.00235985	0.040401795	2893
Cartpt	157.5200213	0.476260065	0.156678663	3.039725106	0.00236794	0.040491229	1238
Pdxdc1	2676.559298	0.229338396	0.075445509	3.039788556	0.00236744	0.040491229	4228
Nenf	624.8998238	0.350075405	0.11519655	3.038940026	0.00237412	0.040529955	742
Rnf227	65.84851788	-1.134444355	0.373316232	-3.038829438	0.00237499	0.040529955	2081

Nelfb	1801.893249	0.320072683	0.105372162	3.037544997	0.00238514	0.040674637	2637
Fam118a	278.4153564	0.312815382	0.102997946	3.037103103	0.00238864	0.04069719	2518
Dap3	625.6938815	0.258454589	0.085105899	3.036858684	0.00239058	0.04071382	4760
Cdk13	2142.438264	0.540543858	0.178004907	3.036679528	0.002392	0.040721643	12548
Deaf1	1282.245496	0.389997722	0.128470858	3.035690183	0.00239986	0.040789882	2135
Lamp1	12739.12117	0.212342562	0.069945707	3.035819798	0.00239883	0.040789882	2229
Gas8	295.0587272	0.358200991	0.117993494	3.035768982	0.00239923	0.040789882	1681
Kcnc2	190.6694278	0.359543224	0.118495972	3.034223175	0.00241156	0.040939464	2944
Nucks1	319.8839919	0.551502172	0.181790396	3.033725572	0.00241554	0.040974187	2375
Eif3m	904.7311364	0.222761287	0.073451797	3.032754745	0.00242333	0.040974897	1283
Prmt2	484.7000706	0.461315749	0.152087094	3.033234023	0.00241948	0.040974897	2052
Glud1	7179.775271	0.299474826	0.098740356	3.032952678	0.00242174	0.040974897	3390
Tra2b	984.7406828	0.239154865	0.078841094	3.033378315	0.00241832	0.040974897	3403
Plin4	1151.292029	-1.140564738	0.376072221	-3.032834318	0.00242269	0.040974897	6350
Kmt5b	117.200018	-0.72621175	0.239482628	-3.032419331	0.00242602	0.040987722	3216
Fus	392.7342779	-0.465527661	0.15352377	-3.032283939	0.00242711	0.040989756	5536
Mrps24	566.9444958	0.41402754	0.136546242	3.032141589	0.00242825	0.04099274	995
D5Ertd579	175.6926533	-0.395236742	0.130361294	-3.031856548	0.00243055	0.041003005	2014
Mpnd	2361.472251	0.362603494	0.119610972	3.031523676	0.00243323	0.041027661	1701
Mrpl46	329.0814445	0.287889122	0.094982525	3.03096933	0.0024377	0.04104935	1134
Gsg1l	790.6722747	0.420421738	0.138714808	3.030835316	0.00243878	0.04104935	3923
Anxa6	3937.724609	0.392186587	0.129434139	3.030008852	0.00244547	0.041070437	2471
Map2	816.7493635	0.401380684	0.13251238	3.029005157	0.00245361	0.041158154	1695
Cc2d1a	1651.698486	0.296373267	0.097865462	3.028374473	0.00245873	0.041226618	3471
Naxd	594.2082893	0.317291022	0.104819476	3.027023559	0.00246975	0.04128173	1359
Prrt3	1110.185112	0.462374442	0.152775891	3.026488279	0.00247412	0.041338572	3304
Ralyl	688.2271497	0.358511251	0.118468971	3.02620381	0.00247645	0.041344854	2933
Sephs2	738.341771	0.199224745	0.065830782	3.026315963	0.00247554	0.041344854	2177
Cdk2ap1	1485.032847	0.276328202	0.091321183	3.025893806	0.00247899	0.041370952	1315
Sec13	1102.69151	0.240196727	0.07940942	3.024788855	0.00248807	0.041463026	1353
Clpb	667.5966585	0.284893056	0.094191167	3.02462604	0.00248941	0.041463026	4159
Klhl11	1716.787248	0.224676516	0.074300472	3.023890852	0.00249547	0.041541582	2393
Pelo	572.1976786	0.282593402	0.0934559	3.023815518	0.00249609	0.041541582	1600
Abhd8	5562.523415	0.373287309	0.123472908	3.023232501	0.0025009	0.041575425	1978
Ola1	1114.889381	0.277668881	0.091864673	3.022586058	0.00250625	0.041628889	2175
Smap2	4241.459424	0.248923549	0.082361914	3.022313787	0.0025085	0.041633688	2905
Myh14	4207.099167	0.408228153	0.135092699	3.021837272	0.00251246	0.041650299	6499
Septin5	60.94478451	1.2589008	0.416593564	3.0218921	0.002512	0.041650299	1314
Trim44	7299.000223	0.151661235	0.050228438	3.019429663	0.00253251	0.04185167	5612
Tomm5	608.6968565	0.339232986	0.112345451	3.019552503	0.00253148	0.04185167	639
Eif2b5	1779.338399	0.224713914	0.074422367	3.019440569	0.00253242	0.04185167	2554
Mecp2	509.6751546	0.59177827	0.195975249	3.019658219	0.0025306	0.04185167	1739
Relch	1220.940322	0.397206027	0.131558304	3.0192395	0.0025341	0.041861611	4328
Eif4a1	2289.256081	0.244002855	0.080820874	3.019057364	0.00253563	0.04187045	1774
Klc2	11206.41804	0.35949053	0.119081043	3.018872867	0.00253717	0.041875832	3169
Cct7	1340.928817	0.375871878	0.124530018	3.018323479	0.00254177	0.041890507	1544
Garem1	90.45407931	-0.931036873	0.3084797	-3.018146323	0.00254326	0.041898489	4932
Ppp2r2c	13913.41072	0.301954153	0.100066551	3.017533346	0.00254841	0.041950673	4088
Mark1	3518.376264	0.303988949	0.100763957	3.016842112	0.00255423	0.041980181	4221
Ube2d3	1740.370005	0.284700876	0.09438131	3.016496323	0.00255714	0.041980181	2504
Rnf41	2253.848087	0.360630808	0.119550805	3.016548567	0.0025567	0.041980181	3144
Ik	1247.333077	0.26763826	0.088715256	3.016823389	0.00255439	0.041980181	1996
Nrxn2	10357.28967	0.433543632	0.143714289	3.016705115	0.00255538	0.041980181	3503
Lemd3	1309.836082	0.246160169	0.081619654	3.015942316	0.00256182	0.042034276	4743
Fbxo2	5066.900186	0.445516156	0.147755014	3.015235447	0.0025678	0.042073536	1288
Ppm1f	2539.084892	0.213409776	0.070780701	3.015084233	0.00256908	0.042078234	4922
Snx3	1957.100446	0.278613948	0.092413213	3.014871349	0.00257088	0.042091495	1401
Timm10b	79.75177433	-0.420449213	0.13950297	-3.01390869	0.00257905	0.042176351	1083
Rilpl1	965.0105967	0.304807097	0.101138419	3.013761735	0.0025803	0.042180492	2249
Ppp1r3e	934.28924	0.688914493	0.228599621	3.013629206	0.00258143	0.042182634	1852
Vps11	2318.888283	0.250916768	0.083297367	3.012301318	0.00259275	0.042334934	3536
Soga3	9705.621598	0.414259479	0.137568968	3.011285794	0.00260144	0.042424648	4105
Nemf	395.6058989	0.266063632	0.08835828	3.01119072	0.00260225	0.042424648	4732
Ipmk	108.2821228	0.599289388	0.199036947	3.010945441	0.00260436	0.042442589	1492
Kcnb1	1977.131244	0.577999244	0.19198214	3.010692787	0.00260652	0.042461574	4015
Stk16	139.3789467	0.619550549	0.205799968	3.010450173	0.00260861	0.042479166	691
Cdr2l	11053.30984	0.455749107	0.151401295	3.01020613	0.0026107	0.042496969	3700
Eapp	332.6215187	0.293819482	0.09762773	3.009590441	0.002616	0.042566834	2658
Usp39	665.0691589	0.276354219	0.091852417	3.008676612	0.00262388	0.042608936	2244
Hspa8	38922.7774	0.289677785	0.096280355	3.008690463	0.00262376	0.042608936	2394
Pard3	878.2668773	0.34575057	0.114931161	3.008327482	0.0026269	0.042629466	5659
Nat14	308.7555091	0.385605021	0.128199311	3.007855631	0.00263098	0.042632229	1415
Urgcp	2171.601394	0.2589183	0.086080786	3.00785242	0.00263101	0.042632229	3972
Twnk	33.48147374	1.1649814	0.387399314	3.007184987	0.00263679	0.042691839	2195

Ankrd6	657.1932876	0.278541263	0.092646822	3.006484819	0.00264287	0.042738096	4444
Mrpl43	1256.520011	0.314012953	0.104446789	3.006439519	0.00264327	0.042738096	1398
Rangap1	229.5022178	0.593477242	0.197429071	3.006027635	0.00264685	0.042772885	990
Heatr5b	3058.948194	0.155046361	0.051588353	3.005452815	0.00265186	0.0428048	6388
Man2a2	7210.149883	0.209463287	0.069716426	3.004504075	0.00266014	0.04290581	6554
Cavin3	1762.173396	0.456309403	0.151911317	3.003788071	0.00266641	0.042941471	1662
Xpot	2462.16839	0.157894091	0.052564469	3.003817838	0.00266615	0.042941471	6005
Tnks2	3840.933479	0.13533117	0.045059472	3.003390075	0.0026699	0.042981326	6481
Gas6	7025.835101	0.302902017	0.100888577	3.002342052	0.00267911	0.043113197	2548
Mospd3	483.6210727	0.355247685	0.118370896	3.001140459	0.00268971	0.043204209	1318
C2cd2l	954.6841243	0.433357623	0.144388651	3.001327453	0.00268805	0.043204209	2808
Eif4a3	1026.054523	0.2370056	0.07897233	3.00112204	0.00268987	0.043204209	1509
Rraga	2529.370278	0.248399436	0.082776994	3.000826979	0.00269248	0.043215665	1618
Use1	655.8208649	0.441178846	0.147025557	3.000694943	0.00269364	0.043215665	854
Eif4g3	3672.431983	0.204672963	0.068214505	3.000431692	0.00269597	0.043220282	5289
Abcg4	1234.160185	0.344736306	0.114891595	3.000535464	0.00269505	0.043220282	3999
Ogfod2	30.40433442	0.937984131	0.312634521	3.000257709	0.00269751	0.043228611	532
Caml	983.9784884	0.277350086	0.092447431	3.000084306	0.00269905	0.043236864	1382
Armt1	283.9959397	0.285699524	0.095265526	2.998981238	0.00270884	0.043332936	2429
Kif21a	9759.984265	0.436236636	0.145463247	2.998947476	0.00270914	0.043332936	6333
Marchf5	1390.675789	0.234896435	0.078322169	2.999105327	0.00270774	0.043332936	1809
Eif3f	3725.867038	0.280264398	0.09350606	2.997285928	0.00272395	0.043520513	2514
Pclo	14284.54214	0.279741871	0.0933906	2.995396443	0.00274088	0.043741526	20084
Lsm14b	1487.061865	0.371799785	0.124130478	2.995233668	0.00274235	0.043748386	2534
Tbp	76.65712002	-0.84302187	0.281557144	-2.994141288	0.00275219	0.043798632	2067
Pcdhgc3	3582.633701	0.292498629	0.097676163	2.994575329	0.00274827	0.043798632	4687
Vim	4859.011062	0.625296448	0.208877811	2.993599196	0.00275708	0.043834756	1777
Sema6a	623.8684109	0.528096357	0.176430982	2.993217815	0.00276053	0.043873098	4123
Wsb2	1095.255562	0.718101525	0.239965961	2.992514115	0.0027669	0.043891991	1526
Arpc1b	2763.602156	0.614131478	0.205192456	2.992953496	0.00276292	0.043891991	3105
Ubqln2	7101.509175	0.291675448	0.097467686	2.992534878	0.00276671	0.043891991	3344
Tmem120l	360.7588439	0.200263946	0.066945242	2.991458974	0.00277648	0.043928744	2164
Znhit3	138.7674976	0.365789968	0.122259671	2.991910299	0.00277238	0.043928744	998
Crebbp	4629.072481	0.339869204	0.113596194	2.991906615	0.00277241	0.043928744	7749
Dcaf6	62.72335012	-0.681928715	0.227991789	-2.991023129	0.00278044	0.043934514	849
Cyp2u1	911.1838327	0.184863885	0.061820015	2.990356513	0.00278652	0.043934514	4378
2410004B1	301.30973	0.360326719	0.12050714	2.990086063	0.00278899	0.043934514	1547
Ift22	845.3205919	0.212539455	0.071080346	2.990129719	0.00278859	0.043934514	3071
Pgbd5	1227.414144	0.353329925	0.118136035	2.990873389	0.00278181	0.043934514	2824
Klhdc2	3578.856256	0.246200496	0.082331672	2.990349764	0.00278658	0.043934514	1905
Hsp90aa1	16353.04989	0.246406584	0.082408783	2.990052467	0.0027893	0.043934514	2852
Metap1	907.4105994	0.184888915	0.061854529	2.989092586	0.00279807	0.044002314	2686
Dctn2	12067.32787	0.388059533	0.129810485	2.98943135	0.00279497	0.044002314	1784
Psmc1	2138.110199	0.219331113	0.073381409	2.988919348	0.00279966	0.044002314	1591
Vars2	890.0881664	0.263591331	0.088190431	2.988888114	0.00279995	0.044002314	4425
Dpy30	315.4058806	0.209616711	0.070155752	2.987876326	0.00280923	0.044084535	734
Emc1	2599.18879	0.310924483	0.104108697	2.986537068	0.00282157	0.044196188	6232
L3mbtl2	816.2114511	0.273778156	0.091668492	2.986611326	0.00282088	0.044196188	3487
Ctif	10944.4083	0.323200753	0.108218409	2.986559832	0.00282136	0.044196188	6031
Rps6	3606.496463	0.299146923	0.100178519	2.986138416	0.00282525	0.04423748	878
Ano8	3543.973	0.449467072	0.150529589	2.985905121	0.0028274	0.044242032	3653
Cul9	4444.69106	0.270749316	0.09067653	2.985880849	0.00282763	0.044242032	7848
Ank2	1440.890347	0.41223522	0.138098599	2.985078938	0.00283505	0.044278623	3536
Pmpcb	711.3160179	0.24641594	0.082548558	2.985102901	0.00283483	0.044278623	1615
Bag1	1851.645113	0.60614489	0.20308437	2.98469494	0.00283861	0.044282985	1336
Eprs	4388.775042	0.246682095	0.082653257	2.984541723	0.00284004	0.04428885	4906
Nav3	466.7022435	0.506213501	0.169624893	2.984311399	0.00284217	0.04429014	9962
Ndufaf6	79.81014183	0.436779754	0.146373981	2.983998608	0.00284508	0.044309712	1082
Saraf	5043.030571	0.283742997	0.095104155	2.983497373	0.00284975	0.044309712	1860
Peli1	679.0257596	0.294478289	0.098700834	2.983544096	0.00284931	0.044309712	3514
Ctnnd2	3348.738055	0.367547211	0.123185057	2.983699649	0.00284786	0.044309712	5944
Cnpy3	1538.190777	0.26094186	0.087449904	2.983901059	0.00284599	0.044309712	1908
Clint1	1261.656841	0.33811624	0.113363457	2.982585823	0.00285825	0.044425563	3398
Cyb5b	5661.98158	0.245501662	0.08232385	2.98214504	0.00286236	0.044466354	4316
P4htm	1631.508839	0.298359994	0.10005682	2.981905611	0.0028646	0.044466354	1841
Iscu	4652.573044	0.374774765	0.125711481	2.981229406	0.00287094	0.04448462	957
Ufsp1	323.0650525	0.356725851	0.119659712	2.981169226	0.0028715	0.04448462	1037
Dctpp1	161.0119408	0.3628341	0.121695664	2.9814875	0.00286852	0.04448462	676
Park7	782.9255317	0.364502144	0.122285018	2.980758805	0.00287535	0.044518023	908
Jund	12982.83554	0.52152367	0.175032942	2.979574378	0.00288649	0.044651502	1667
Mxd4	376.6842811	0.496589387	0.166735567	2.978305082	0.00289847	0.04480411	1160
Bcl7b	1268.97662	0.266123654	0.089383255	2.977332318	0.00290769	0.044859571	1758
Gramd1a	1293.840802	0.34844105	0.117024316	2.97750982	0.002906	0.044859571	2713
Rock2	2647.728331	0.124126087	0.041688927	2.977435409	0.00290671	0.044859571	7644

Slc24a2	6059.139776	0.319147534	0.107212476	2.976776071	0.00291297	0.04488727	10499
Trpc4ap	6478.837152	0.201779133	0.067790917	2.976492165	0.00291567	0.044893449	3204
Cacng7	4981.625705	0.279594093	0.093944022	2.976177579	0.00291866	0.044919325	2212
Prdm2	4267.377641	0.132585449	0.0445565	2.975670165	0.00292349	0.044977358	7304
Cbfb	278.9638806	1.237974238	0.416067667	2.975415625	0.00292592	0.044982009	2883
Sorbs2	144.2189938	-0.556681	0.187116155	-2.97505579	0.00292935	0.045018462	3798
Cenpb	7571.491895	0.240898943	0.080987209	2.974530738	0.00293437	0.045062864	4886
Itprid2	4088.627048	0.229626106	0.077221369	2.973608333	0.00294321	0.045163726	5168
Tesk1	2047.916567	0.498928477	0.167784867	2.973620237	0.00294309	0.045163726	3947
Lrpap1	4557.034363	0.298538929	0.10039947	2.973510996	0.00294414	0.045163726	2981
Miga2	2283.229623	0.256763494	0.086378002	2.972556532	0.00295331	0.045245691	3381
Hnrnpll	596.696053	0.275728458	0.092754747	2.972661417	0.0029523	0.045245691	3009
Narf	1528.777267	0.189201974	0.063670285	2.971589856	0.00296262	0.045356064	4395
Dlat	3705.201853	0.225960241	0.076045451	2.971384072	0.00296461	0.045362677	4035
Ubl3	3833.611805	0.259769122	0.087430982	2.971133529	0.00296703	0.045383301	2537
Maged1	20236.19603	0.290289336	0.09771855	2.970667643	0.00297153	0.04541938	2821
Mthfd2l	212.130741	0.240853992	0.081085964	2.970353664	0.00297457	0.045449424	2159
Igsf8	3106.955086	0.327691214	0.110347838	2.969620603	0.00298168	0.045498014	2278
Cend1	5003.778533	0.378905237	0.127589244	2.969727109	0.00298064	0.045498014	1733
C030006K1	457.2726447	0.3086522	0.103941942	2.969467323	0.00298317	0.045498645	1754
Sowahc	1348.220571	0.28061957	0.094516138	2.969012232	0.00298759	0.045516894	4482
Ctps	845.4249527	0.189972985	0.064001956	2.968237188	0.00299513	0.045549876	2705
Usp10	1658.108084	0.190452767	0.064174389	2.967737919	0.003	0.045607541	3274
Golim4	1107.031863	0.366712088	0.123618066	2.966492694	0.00301218	0.045748336	4551
Ppp1r8	651.5993606	0.16622887	0.056036957	2.966414985	0.00301294	0.045748336	2037
Paip1	1081.593889	0.299674627	0.101024738	2.966348956	0.00301358	0.045748336	1731
Kctd7	925.0839664	0.217378522	0.073306025	2.965356826	0.00302332	0.045863261	4291
B3galt6	249.6275578	0.338623971	0.114235849	2.964253111	0.00303419	0.045945781	3184
Syt5	95.05117483	0.564558013	0.190472088	2.963993404	0.00303675	0.045968134	397
Mrps28	159.9631785	0.374582958	0.126392146	2.963656924	0.00304007	0.045985541	783
Rps18	1469.429075	0.329562586	0.111216752	2.963245901	0.00304413	0.046030549	560
Ptges2	685.4471381	0.333766007	0.112647945	2.962912517	0.00304743	0.04604098	4978
Flywch2	202.995516	0.356104711	0.120182304	2.963037798	0.00304619	0.04604098	694
Dmd	206.5544921	0.531179044	0.179279972	2.962846542	0.00304809	0.04604098	4179
Trim28	2212.717791	0.209481169	0.070723853	2.961959239	0.00305688	0.0461245	3245
Rita1	30.97242279	0.982190122	0.331624955	2.961749737	0.00305896	0.046127144	733
Qrich1	3077.69479	0.272383309	0.091996659	2.960795675	0.00306845	0.04613469	3040
Calcoco1	2225.109703	0.27870863	0.094130779	2.960866087	0.00306775	0.04613469	2824
Pip5k1b	503.2270049	0.300497857	0.101488336	2.960910282	0.00306731	0.04613469	2573
Tmsb15l	35.0327278	0.652066768	0.220214822	2.961048498	0.00306594	0.04613469	816
Araf	1312.278799	0.321036286	0.108475325	2.95953282	0.00308106	0.046270615	1520
Ccar2	2574.683779	0.217699531	0.073575557	2.958856717	0.00308783	0.046310832	3725
Coa3	810.484401	0.280878437	0.095007332	2.956386963	0.00311266	0.046633772	886
Apbb1	8734.759659	0.303678348	0.102757674	2.955286332	0.00312379	0.046655526	2690
Irf2bp2	5147.526326	0.298502228	0.100999099	2.955493981	0.00312169	0.046655526	5080
Mpst	563.9771486	0.307655596	0.104101096	2.955354037	0.0031231	0.046655526	1279
Qk	3720.697834	-0.372407696	0.126015105	-2.955262361	0.00312403	0.046655526	8547
Lrpprc	2718.564444	0.176470666	0.059723886	2.954775321	0.00312897	0.046663399	4393
A830018L1	111.5609127	0.725101369	0.245468247	2.9539518	0.00313733	0.046673162	1832
Cog4	2003.429021	0.28558556	0.096681595	2.953877203	0.00313809	0.046673162	2737
Cnot10	663.5501034	0.179568137	0.060791373	2.953842445	0.00313844	0.046673162	2853
Abcf1	2229.986397	0.405868505	0.137393043	2.954068835	0.00313614	0.046673162	3161
Asl	374.6581518	0.439351133	0.148754238	2.953536903	0.00314155	0.046686593	1824
Hspa9	4339.763753	0.167323385	0.056670104	2.952586488	0.00315124	0.046748513	3049
Cct3	2995.075901	0.297353987	0.100721895	2.952227893	0.0031549	0.046786448	1977
Thsd7a	658.5547048	0.572205019	0.193891118	2.951166748	0.00316576	0.046858622	5678
Aopep	60.78426509	0.914934056	0.310042498	2.95099563	0.00316751	0.046858622	1055
Map6	3568.565654	0.646270345	0.219082298	2.949897596	0.00317879	0.046992634	2906
Ubqln4	3279.675366	0.248129306	0.08415507	2.948477228	0.00319344	0.047159719	3330
Pdap1	941.741212	0.244650908	0.082990082	2.947953572	0.00319885	0.047190298	2353
Tenm2	800.4486663	0.452674043	0.153644983	2.946233806	0.00321669	0.047362469	8298
Gldc	77.9290109	0.386897564	0.131374076	2.945006937	0.00322947	0.047493126	3767
Usp48	402.2186179	-0.526974911	0.178968605	-2.944510353	0.00323466	0.047513857	3036
Celf4	1965.872456	0.33084967	0.112381962	2.943974843	0.00324026	0.047551559	3995
Nyap1	4570.768715	0.419667784	0.14266832	2.941562526	0.00326561	0.047841738	2499
Lancl1	2642.108745	0.280806933	0.095483583	2.94089228	0.00327268	0.047879107	4461
Sync	834.2142216	0.516987398	0.175790166	2.94093469	0.00327224	0.047879107	2042
Abcb9	2709.728625	0.305010193	0.103732644	2.940349167	0.00327843	0.047899137	3661
Bin1	4930.167097	0.315700784	0.107400942	2.939460106	0.00328785	0.047951812	2406
Ap1s1	127.3000995	0.745715179	0.25376146	2.938646314	0.00329649	0.04801173	483
Hsp90ab1	52181.55185	0.272575405	0.092753931	2.93869382	0.00329599	0.04801173	2520
Igfbpl1	35.89832215	0.687392612	0.233945125	2.938264318	0.00330056	0.04802764	2864
Mvb12a	678.5762395	0.285132418	0.097043908	2.938179473	0.00330146	0.04802764	1062
Riox1	323.09618	0.316856238	0.107848641	2.93797154	0.00330367	0.04802764	2344

Tollip	4794.441225	0.211968388	0.072170383	2.937055062	0.00331345	0.048104437	3788
Smug1	32.16858545	0.710496861	0.241952623	2.936512327	0.00331926	0.048128079	491
Ap3d1	8548.186597	0.255865825	0.087175505	2.935065605	0.00333477	0.048309077	4805
Fzd8	1072.371161	0.49408546	0.168340897	2.935029278	0.00333516	0.048309077	5974
Vapb	7419.995188	0.233382347	0.079524343	2.93472837	0.0033384	0.048322882	7032
Sf3b5	554.9901989	0.474551748	0.161696914	2.93482254	0.00333739	0.048322882	3841
Sf3a2	837.4838606	0.397210351	0.135394619	2.933723319	0.00334923	0.048452862	2213
Atmin	4451.305445	0.242740905	0.082751488	2.9333721	0.00335302	0.04846822	4877
Acad9	687.1353733	0.27686666	0.094394023	2.933095242	0.00335601	0.048494906	3930
Ndufb9	1310.197399	0.223357992	0.076171627	2.932299076	0.00336463	0.048602826	704
Mtss1	2374.200029	0.147267567	0.050229431	2.931898009	0.00336897	0.048649037	4938
Gak	3529.737656	0.254605649	0.086862666	2.931128635	0.00337733	0.048659743	4453
Nop2	830.74957	0.271510499	0.09263029	2.931120051	0.00337742	0.048659743	2597
Hspbp1	1897.641368	0.315893042	0.107773269	2.93108899	0.00337776	0.048659743	1593
Ndrg1	38.67395667	-0.72584663	0.247583822	-2.931720758	0.0033709	0.048659743	738
Taf1	798.3256165	0.317706025	0.108377624	2.931472493	0.00337359	0.048659743	5954
Cisd1	1243.829383	0.291543951	0.099470398	2.930961935	0.00337914	0.048663091	1084
Bag4	1272.66688	0.179798362	0.06135012	2.930692899	0.00338207	0.04868869	4876
Trap1	2332.116886	0.210605237	0.071864806	2.930575456	0.00338335	0.048690543	2320
Mbnl1	517.8420847	0.501599889	0.171195343	2.92998559	0.00338978	0.048766489	4552
Sh3glb2	729.5635974	0.653035342	0.222927862	2.929357213	0.00339664	0.048848597	1749
Cox6c	1422.20716	0.216111677	0.073787075	2.928855464	0.00340213	0.048910901	503
Pars2	49.05604458	-0.685152591	0.233976949	-2.928290994	0.00340831	0.04896654	3046
Galnt2	2110.701293	0.228419941	0.078016121	2.927855663	0.00341308	0.049018516	4113
Stk25	1053.168926	0.362721383	0.123924001	2.926966356	0.00342286	0.049021071	4249
Naa20	44.32047703	-0.729524757	0.249257979	-2.926785977	0.00342485	0.049021071	1883
Rab43	620.0063882	0.361303786	0.123443891	2.926866472	0.00342396	0.049021071	4605
Tada3	298.0945264	0.557980135	0.190659703	2.926576119	0.00342716	0.049021071	1282
Nol6	4346.902555	0.213637972	0.07303535	2.925131095	0.00344311	0.049149661	4599
Mtmr3	2788.063413	0.198790491	0.067973998	2.924507865	0.00345001	0.049215002	5674
Mcoln1	1994.469864	0.251963561	0.08616375	2.924240903	0.00345297	0.049226234	2065
Txndc5	2926.546971	0.353081431	0.120747829	2.924122392	0.00345429	0.049226234	2843
Akr7a5	811.8831041	0.361511471	0.123637582	2.923961016	0.00345608	0.049235189	1288
Bbip1	118.7377305	0.407886115	0.139506813	2.923772015	0.00345818	0.049248521	662
Fbln7	293.8916926	0.393499898	0.134632795	2.922764089	0.00346939	0.0493087	2905
Scn1b	12992.43987	0.397626857	0.136069708	2.922229074	0.00347536	0.049376911	1573
Cdh18	780.591771	0.270761824	0.092668907	2.921819548	0.00347993	0.049408718	2744
Creb3	978.0648242	0.24889353	0.085249351	2.919594435	0.00350487	0.04972948	1884
Ndufa6	579.1188379	0.351535597	0.120422791	2.919178276	0.00350956	0.049762568	590
Clcn4	314.3035815	0.628023025	0.215154131	2.918944773	0.00351218	0.049766947	2445
Josd1	1806.98978	0.249176934	0.085394564	2.917948425	0.00352343	0.049875742	3440
Fbxo9	2060.277289	0.337860005	0.115829383	2.916876496	0.00353556	0.049957869	2155
Psd	354.7542122	0.483762356	0.16583578	2.917116903	0.00353283	0.049957869	2034
