# Supplementary material for: Gender-different effect of Src family kinases antagonism on photophobia and trigeminal ganglion activity
Source: J Headache Pain. 2024 Oct 11;25(1):175. doi: 10.1186/s10194-024-01875-3 (PMC11468534; doi:10.1186/s10194-024-01875-3)
Supplement: Supplementary file 2 — Supplementary Material 2: S2 Table. RNA-sequencing analysis shows gene expression profile of TG in male photophobia mice in the absence or presence of SRCT. [file 10194_2024_1875_MOESM2_ESM.pdf]

Supporting Table 2. RNA-sequencing analysis shows gene expression profile of TG in male photophobia mice in the absence or presence of SRCT.

GeneID	baseMean	log2FoldChange	lfcSE	stat	pvalue	padj	length
Scn8a	257.2014901	-26.25555456	2.986726589	-8.790745916	1.49E-18	6.53E-14	7120
Flot2	145.1322853	-25.622442	2.986801122	-8.578556442	9.61E-18	1.10E-13	2699
Add2	127.1955564	-25.55770401	2.986825246	-8.556812636	1.16E-17	1.10E-13	2289
Rreb1	123.9258763	-25.52342244	2.986830397	-8.545320305	1.28E-17	1.10E-13	8395
Mast2	119.1556595	-25.46828832	2.986838418	-8.526838335	1.50E-17	1.10E-13	5445
Baz2a	70.53504558	-24.64203859	2.986982041	-8.249811431	1.59E-16	9.96E-13	8385
Magi2	51.84183823	-24.32901978	2.987108951	-8.144671045	3.80E-16	2.09E-12	4478
Erc1	58.22990974	-24.24608183	2.987056446	-8.11704843	4.78E-16	2.33E-12	8356
Tdp1	42.9359032	-24.07854831	2.987208269	-8.060552244	7.60E-16	2.62E-12	2019
Tnk2	49.71361202	-24.07534091	2.987129472	-8.059691128	7.65E-16	2.62E-12	939
Clvs1	42.29274194	-24.05420447	2.987217067	-8.052379164	8.12E-16	2.62E-12	3526
Birc2	41.17666479	-24.02145917	2.987233019	-8.041374414	8.88E-16	2.62E-12	3130
Asap1	69.39943913	-24.01737149	2.986987848	-8.040665953	8.94E-16	2.62E-12	3228
Dync1i2	37.9572083	-23.90854738	2.987284166	-8.003439262	1.21E-15	3.13E-12	2466
Lasp1	39.89513944	-23.85516029	2.987252442	-7.985652621	1.40E-15	3.41E-12	686
Ambra1	34.43407912	-23.76881103	2.987351038	-7.956484096	1.77E-15	4.10E-12	4690
Gtf2i	30.59348367	-23.61742404	2.987441594	-7.905568461	2.67E-15	5.86E-12	4381
Stat3	28.92380479	-23.54004105	2.987488408	-7.879542222	3.29E-15	6.88E-12	2506
Gm28635	25.92558239	-23.38733426	2.987587652	-7.828166731	4.95E-15	9.72E-12	4734
Tyk2	25.77005346	-23.37728823	2.987593368	-7.824789171	5.09E-15	9.72E-12	4828
Ckmt1	24.80768624	-23.3315088	2.98763081	-7.80936812	5.75E-15	1.01E-11	851
Fyn	23.34015111	-23.24864831	2.987693757	-7.781469655	7.17E-15	1.17E-11	675
Pabpc4	21.70240843	-23.15054333	2.987774039	-7.748425092	9.30E-15	1.30E-11	3050
Snx25	21.65576292	-23.14514014	2.987776351	-7.746610664	9.44E-15	1.30E-11	3067
Fubp1	33.81722288	-23.14389691	2.98736401	-7.747263753	9.39E-15	1.30E-11	2552
Pip4p1	116.2506648	-10.33808877	2.823756521	-3.661111958	0.000251123	0.007444789	1445
Akap8	84.38288813	-9.875910554	2.019634349	-4.889949787	1.01E-06	0.000209808	3731
Pgap6	58.0294842	-9.335718935	2.728215949	-3.421913481	0.000621821	0.012727976	2235
Dnajb6	39.00687923	-8.762599766	1.622913283	-5.399302512	6.69E-08	3.20E-05	505
Pde1a	35.5462166	-8.628692994	2.624167936	-3.288163412	0.001008433	0.017078487	4122
Phc1	32.9330097	-8.51826415	1.245849864	-6.837311941	8.07E-12	8.06E-09	3127
Trp53	32.69717164	-8.508120479	2.636322354	-3.227268648	0.001249781	0.019470802	1772
Dtnbp1	32.32607547	-8.491468673	1.712075737	-4.959750606	7.06E-07	0.000163327	1305
Tfr2	31.23673687	-8.442238719	1.174752091	-7.186400246	6.65E-13	7.13E-10	2926
Gm14434	30.10106392	-8.388758947	2.987454768	-2.807995299	0.004985095	0.044958192	1458
Gabpb2	29.71488571	-8.370127037	2.987465487	-2.80174853	0.005082648	0.045527719	8661
Smarca1	24.76742158	-8.107421725	2.071219996	-3.914321868	9.07E-05	0.003884802	4024
Slc37a1	24.18470153	-8.073119357	1.598521345	-5.050366942	4.41E-07	0.000118172	2972
Slc29a1	23.06194272	-8.004194826	1.224681061	-6.535738227	6.33E-11	6.05E-08	2216
Avp	57.89327642	-5.576189971	1.379227534	-4.042980461	5.28E-05	0.002769512	577
Pld3	143.7665121	-4.641962628	1.567840656	-2.960736226	0.003069047	0.033659894	2297
Luc7l	22.78613314	-4.27422091	0.673530775	-6.345991998	2.21E-10	1.87E-07	1700
9430015G1	21.7853867	-3.75002818	1.271577247	-2.94911551	0.003186848	0.034382771	2528
Cdyl	30.87090718	-3.518753099	1.26445588	-2.782819989	0.00538887	0.047261451	3470
Rhoq	28.33669604	-3.168316251	1.12570423	-2.814519273	0.004885025	0.044442658	544
Pabpn1	227.5835054	-2.563935077	0.481182235	-5.328407593	9.91E-08	4.31E-05	1030
Ttc3	33.57101432	-2.040245328	0.70607518	-2.889558203	0.003857836	0.038706014	671
Dnm3	56.50698893	-1.873695194	0.619904098	-3.022556552	0.002506492	0.029735008	1305
Zfp668	28.42239676	-1.833273894	0.62901754	-2.914503615	0.003562547	0.036679233	3132
Pccb	30.63073825	-1.69814188	0.522929768	-3.247361276	0.001164804	0.018621132	2188
Rplp2	225.0016187	-1.596976098	0.521170441	-3.064210806	0.002182451	0.027438216	449
Ints8	64.13725799	-1.326741442	0.413535039	-3.20829269	0.001335255	0.020249914	3433
Ginm1	40.49104516	-1.322890796	0.446240125	-2.964526767	0.003031489	0.033369905	1557
Csf1r	43.28240225	-1.289657738	0.339819163	-3.795129528	0.000147566	0.005317833	416
Setd5	380.5952902	-1.287997607	0.420231879	-3.064968824	0.002176927	0.027396603	6527
Zdhhc4	27.38397121	-1.275413062	0.383262508	-3.327779357	0.000875412	0.015767094	848
Carm1	104.9333852	-1.221061224	0.432268125	-2.824777388	0.004731348	0.043758985	3152
Prpf8	3016.972129	-1.199766247	0.229545768	-5.226697304	1.73E-07	6.54E-05	7249
Srsf1	125.2412253	-1.199458239	0.397597134	-3.016767822	0.002554854	0.030082961	1209
Zxdc	247.7643457	-1.188056365	0.334336321	-3.553476818	0.000380175	0.009466978	1612
Gabrb3	217.4127474	-1.17178321	0.340050452	-3.445909877	0.00056914	0.012011098	4348
Lrch3	27.52495907	-1.168066665	0.404787215	-2.885631315	0.003906295	0.038945728	793
Ttc7b	118.7532519	-1.165192775	0.406160849	-2.868796383	0.00412037	0.040318729	3307
Rnh1	87.68381999	-1.162380091	0.402496777	-2.887923972	0.003877936	0.038801423	445
Atf5	249.9065994	-1.154723009	0.387359793	-2.981008947	0.002873004	0.032238506	1735
Popdc3	40.96720524	-1.150325509	0.346812144	-3.316854756	0.000910369	0.016114572	1369
Ctnnb1	1431.564992	-1.142401218	0.35062745	-3.258162527	0.001121362	0.018185417	2702
Ltbp4	33.94354681	-1.125348643	0.384221424	-2.928906546	0.003401567	0.035624079	5386

Ap2a1	2082.518311	-1.107208795	0.182393061	-6.070454588	1.28E-09	1.06E-06	3449
Ube2l3	85.03377763	-1.104368931	0.335058034	-3.296052676	0.000980536	0.016813291	650
Gnal	247.4538822	-1.073626459	0.29873677	-3.593887887	0.00032578	0.008643887	2022
Cfap54	40.71854075	-1.069149996	0.357992092	-2.986518471	0.002821738	0.031834156	9516
Nqo2	53.03994443	-1.059651258	0.362527779	-2.922951896	0.003467301	0.036063373	957
Dnajc13	23.24032074	-1.036726734	0.374764254	-2.766343706	0.005668875	0.048649638	668
Ism2	53.08728503	-1.035848465	0.365933209	-2.830703635	0.004644574	0.043240743	2526
Abcc4	157.8016073	-1.03195598	0.358986222	-2.874639513	0.00404489	0.039873004	5504
Ciapin1	140.9934063	-1.008385952	0.330109057	-3.054705503	0.002252816	0.027937492	346
Cst6	27.97984955	-0.992487963	0.280631039	-3.536629327	0.000405268	0.009865786	432
Zfp874a	28.10588077	-0.989361254	0.354682196	-2.78943027	0.005280087	0.046604899	1092
Wdr59	71.0178035	-0.979569914	0.226256691	-4.329462736	1.49E-05	0.001258928	3618
Smug1	69.16941459	-0.976603648	0.243485525	-4.010931031	6.05E-05	0.002967814	3882
Asf1b	26.93204582	-0.975127824	0.341526584	-2.855203285	0.004300929	0.041277089	1788
Pik3cd	626.5028302	-0.973525874	0.240818944	-4.042563501	5.29E-05	0.002769512	4832
Cpsf4	97.54753194	-0.967632228	0.329156542	-2.939732631	0.003284956	0.034917669	1713
Celf6	135.8913166	-0.965306074	0.298720953	-3.231464232	0.001231577	0.019262291	2973
Dhx9	280.1031272	-0.958163759	0.191285367	-5.009080271	5.47E-07	0.000135846	2346
Gse1	238.4790657	-0.955940045	0.32737682	-2.91999918	0.003500323	0.03624868	4492
Rpl41	1296.811969	-0.95435039	0.315356271	-3.026261017	0.002475985	0.029515642	437
Bcl2l13	47.95513232	-0.954100714	0.322766187	-2.956011978	0.00311645	0.033939737	349
Kdm1a	232.9046417	-0.936856728	0.284511746	-3.292857817	0.000991746	0.016926868	2051
Plec	1539.161687	-0.929772787	0.243561802	-3.817399852	0.000134866	0.005015991	4937
Sirpa	169.6914013	-0.927210718	0.219429991	-4.225542333	2.38E-05	0.001673241	3342
Klf13	110.9254158	-0.924195106	0.203120386	-4.549986961	5.36E-06	0.000646216	487
Ptgds	7953.59629	-0.92228935	0.326850322	-2.821748332	0.004776264	0.043949029	830
Nab2	147.8629245	-0.918389823	0.290628511	-3.160012834	0.001577622	0.02241763	2311
1700030J2	26.73226039	-0.912440068	0.263916597	-3.457304605	0.000545608	0.011741383	1482
Bscl2	686.8147481	-0.908819081	0.265336689	-3.425154222	0.000614451	0.012619524	1684
Agap3	1340.704398	-0.906385993	0.183989859	-4.926282333	8.38E-07	0.000182042	1191
Fau	672.8551739	-0.893021381	0.28634081	-3.118735966	0.001816286	0.024404961	472
Leng8	614.449839	-0.890435796	0.205242587	-4.338455337	1.43E-05	0.001241818	3620
Pth1r	118.6037761	-0.884326948	0.205298115	-4.30752591	1.65E-05	0.001341629	2219
Rere	1189.085961	-0.879418635	0.231347895	-3.801282208	0.000143949	0.005217418	6503
Gpn2	36.27624391	-0.85581069	0.298831097	-2.863860887	0.004185118	0.040640555	996
Dhrs3	223.1725056	-0.85464387	0.253239456	-3.374844837	0.000738573	0.0141067	2337
Opa1	168.468877	-0.85189139	0.266359449	-3.198277337	0.001382513	0.020674205	3230
Tent4a	135.1424072	-0.850829499	0.237474566	-3.582823682	0.0003399	0.008863408	2118
Cwc15	159.3208242	-0.841312741	0.184243083	-4.566319273	4.96E-06	0.000612811	831
Mtus1	1310.103627	-0.836773681	0.209691557	-3.990497715	6.59E-05	0.003154301	6426
Kcnj13	54.69722019	-0.831743816	0.240610735	-3.456802611	0.000546625	0.011756824	1270
Ndfip1	1883.366386	-0.826420629	0.232426346	-3.555623719	0.000377083	0.009466978	1392
Nlrx1	46.87035845	-0.817907125	0.202508927	-4.038869481	5.37E-05	0.002787881	775
Maz	401.4379059	-0.817454041	0.214470713	-3.811494961	0.000138129	0.005086127	796
Nat9	70.96598461	-0.815468268	0.288813942	-2.823507278	0.004750135	0.043821711	1143
Bhlhe41	28.34191048	-0.814282124	0.266228752	-3.058580707	0.002223882	0.027758446	744
Alkbh3	81.49808451	-0.812220424	0.160858917	-5.04927199	4.43E-07	0.000118172	1395
Cyld	330.0641228	-0.810413712	0.254043637	-3.190057115	0.001422447	0.021028202	4476
Acbd4	35.36833881	-0.809336977	0.203264858	-3.981686685	6.84E-05	0.003227934	434
Nipal3	150.1847249	-0.807235545	0.168740001	-4.78390149	1.72E-06	0.000301143	814
Nrxn1	210.3818372	-0.802083641	0.124227078	-6.456592673	1.07E-10	9.42E-08	1439
Diaph1	256.5915052	-0.791207343	0.239236626	-3.307216612	0.00094228	0.016504796	5666
Robo2	93.30747572	-0.785024282	0.272668255	-2.879045388	0.003988809	0.039479513	886
Agfg1	418.3764543	-0.782882941	0.137237911	-5.704567586	1.17E-08	7.22E-06	3142
Eif5a	365.7350623	-0.780274081	0.188436977	-4.140769465	3.46E-05	0.002119521	1259
Ccdc50	48.78388911	-0.776570161	0.222471081	-3.490656667	0.000481835	0.010880265	1096
Pcdhga7	509.2194324	-0.775212036	0.204757693	-3.785997114	0.000153093	0.005420643	4730
Hhex	27.93289592	-0.770152147	0.266576093	-2.889051828	0.003864054	0.038741876	1802
Igfbp6	755.7655466	-0.768989155	0.174154432	-4.415558925	1.01E-05	0.000995381	1112
Zyx	76.30646405	-0.768562083	0.273719087	-2.807849802	0.004987348	0.044960578	525
Cdca2	29.26111233	-0.765373981	0.23254482	-3.291296627	0.000997267	0.016994125	3568
Apba2	50.71379299	-0.762084239	0.16447569	-4.633415677	3.60E-06	0.000492629	714
Tfdp2	94.38231438	-0.761308614	0.265918504	-2.862939591	0.004197306	0.040679287	2341
Tmod2	448.5439196	-0.754227393	0.211010593	-3.57435796	0.000351088	0.009047831	1528
Usf1	545.6804632	-0.749196549	0.133960892	-5.59265123	2.24E-08	1.26E-05	2167
Nrp2	258.2775039	-0.747209844	0.237054449	-3.152059983	0.00162123	0.02277232	4216
Pcdhga1	174.7189939	-0.744155654	0.183374952	-4.058109607	4.95E-05	0.002662199	4692
Bean1	540.0758687	-0.744092506	0.148739103	-5.002669052	5.65E-07	0.000137926	1373
Mdfic	91.44029771	-0.741606646	0.242253431	-3.061284388	0.002203897	0.027597355	1871
Irf2	41.91981518	-0.739266321	0.234927876	-3.146779913	0.001650792	0.022996532	383
Enah	2259.543951	-0.737870976	0.175997349	-4.192511875	2.76E-05	0.001834046	2832

Tns1	1640.907436	-0.735390803	0.189158832	-3.887689492	0.000101203	0.004169999	2813
Cacna1h	2598.337426	-0.731990229	0.207627911	-3.525490503	0.000422699	0.010094501	8230
Banp	105.990823	-0.730116422	0.254524223	-2.868553776	0.004123531	0.04033162	1289
Shfl	302.2302649	-0.727297317	0.257854511	-2.820572402	0.004793805	0.044010511	1683
Wnk1	33.00439843	-0.726970097	0.234709193	-3.097322644	0.001952772	0.025576882	181
Wipf2	105.9203436	-0.726788645	0.222952532	-3.259835806	0.001114767	0.018131981	660
Pbx2	547.1992413	-0.725873587	0.183238979	-3.961349216	7.45E-05	0.003438194	1990
Fen1	249.6045315	-0.723349998	0.236185294	-3.062637759	0.002193955	0.027527744	2341
Dapk3	772.9162376	-0.719930597	0.125028657	-5.758124698	8.51E-09	5.58E-06	1730
Edc4	273.8873995	-0.719674288	0.251003991	-2.867182643	0.004141439	0.040371915	4645
Ddn	67.90498082	-0.717194226	0.244571771	-2.93244892	0.003363002	0.035367162	368
Stx18	48.99379089	-0.716255589	0.190196086	-3.765879749	0.000165964	0.005722818	900
Pfn2	639.9087644	-0.714831985	0.150074954	-4.763166431	1.91E-06	0.00031659	905
Gpr137c	88.86329692	-0.713619474	0.215322559	-3.314188154	0.000919096	0.016234661	1395
Ints3	205.8989908	-0.713475598	0.257800171	-2.767552846	0.005647889	0.048519853	4038
Mtch1	2784.357517	-0.710107569	0.163924067	-4.331929924	1.48E-05	0.001252319	1897
Mrpl24	132.2371354	-0.709798896	0.250008075	-2.839103879	0.004524043	0.042554458	1115
Proser2	99.06102687	-0.704186595	0.255449287	-2.756659075	0.00583952	0.049553079	2061
Foxc1	802.3075022	-0.700079277	0.214180221	-3.268645797	0.001080635	0.017779587	5844
Jag2	113.448324	-0.69995912	0.189219485	-3.699191553	0.000216287	0.006797049	742
Bmpr1a	28.81722217	-0.692317514	0.229244712	-3.01999338	0.002527802	0.029842862	748
Nudt3	303.0115585	-0.691666245	0.155198357	-4.456659569	8.32E-06	0.000877683	465
Cdc34	66.74799986	-0.691459492	0.188319446	-3.671737069	0.000240907	0.007249484	799
Trafd1	29.54454779	-0.690808113	0.210502063	-3.281716593	0.001031772	0.017340167	627
Sh3gl2	345.7051017	-0.676787961	0.202360416	-3.344468135	0.000824405	0.015177959	2286
Brox	285.54642	-0.676040806	0.237686761	-2.844250988	0.004451597	0.042165696	3413
Gfus	201.7797913	-0.675463428	0.203412462	-3.320659026	0.000898052	0.015965567	1335
Sphk1	46.33647739	-0.67451773	0.196415224	-3.434141793	0.000594433	0.012327478	2527
Gm2296	704.4827535	-0.674066413	0.233109584	-2.891628915	0.003832503	0.038583926	988
Mrps10	127.3115145	-0.66813528	0.166283776	-4.018042519	5.87E-05	0.002915282	1018
Tox2	586.9536267	-0.661979719	0.182569732	-3.625900699	0.000287956	0.00809462	2947
Asphd2	134.4124726	-0.656693989	0.127161027	-5.164270877	2.41E-07	7.98E-05	717
Rpl22	128.3926053	-0.656619651	0.222345553	-2.953149469	0.003145496	0.034108036	474
Tmem164	692.1104877	-0.654027144	0.229300853	-2.852266515	0.004340869	0.041507583	5224
Gps1	340.1308676	-0.653597996	0.194096432	-3.367387994	0.000758838	0.014386517	1843
Sdf4	408.7443224	-0.653170255	0.173045225	-3.774563871	0.000160288	0.005577991	973
Ranbp3	454.8928761	-0.651839553	0.177089661	-3.680844774	0.000232462	0.007113166	2567
Etnk1	78.47487217	-0.649499417	0.220396653	-2.946956808	0.00320918	0.034530497	1791
Foxd2	200.2069827	-0.647895863	0.17576672	-3.686112262	0.000227706	0.007018954	2592
Pawr	121.7687275	-0.647755032	0.233680389	-2.771970022	0.005571817	0.048174026	1745
Lrrc3b	260.5158049	-0.645219961	0.135530108	-4.7607131	1.93E-06	0.000318846	1655
Rab7	2715.368042	-0.645068342	0.124380838	-5.186235697	2.15E-07	7.55E-05	1275
Gm49342	82.07004602	-0.644057345	0.229643322	-2.804598625	0.005037927	0.045276466	750
Gng2	468.2826724	-0.641974652	0.192674474	-3.331913342	0.000862511	0.015643319	393
Fam102b	1887.252507	-0.641408931	0.117565005	-5.455781073	4.88E-08	2.49E-05	5702
Meis3	90.89327679	-0.641348448	0.22668102	-2.829299287	0.004665005	0.04334413	659
Dlg2	442.0872693	-0.639914478	0.226713353	-2.822570746	0.004764031	0.043919191	7801
Rsrp1	3026.894637	-0.638727824	0.201800314	-3.165147818	0.001550042	0.022161818	1749
Tmx2	223.6608402	-0.637829732	0.219236339	-2.90932487	0.003622103	0.037129012	1926
Ccdc28b	112.6949664	-0.637695064	0.215875069	-2.954000511	0.003136835	0.034068909	879
Ubl7	687.3912251	-0.637184173	0.136379945	-4.672125151	2.98E-06	0.000436864	1364
Ints1	1746.376545	-0.635619191	0.127450969	-4.987166418	6.13E-07	0.000144828	7070
Med28	153.365852	-0.632743002	0.21581658	-2.93185539	0.003369436	0.035422586	734
Kcnk1	581.6892722	-0.632271758	0.218261357	-2.896856171	0.003769225	0.038182946	1309
Rpl9	592.3050739	-0.631718151	0.223283891	-2.82921507	0.004666233	0.04334413	680
Rnf44	151.6313462	-0.631471418	0.20822748	-3.032603667	0.002424538	0.029172089	792
Lipa	154.0421434	-0.6306085	0.216502485	-2.912707902	0.003583096	0.036814866	2965
Tpgs1	1283.191436	-0.629457043	0.150564375	-4.180650599	2.91E-05	0.001894177	1101
Pqbp1	195.028587	-0.629005528	0.211047833	-2.980393203	0.002878786	0.032278966	1148
Pdlim1	60.80149739	-0.628273197	0.198067595	-3.172014064	0.001513857	0.021864886	692
Hp1bp3	776.8036728	-0.624702144	0.20456128	-3.053863093	0.002259152	0.02797752	3113
Mrpl48	55.70997076	-0.624582892	0.188770716	-3.308685299	0.000937351	0.016438234	865
Ndufaf3	59.42564671	-0.624101242	0.184173401	-3.388661118	0.000702348	0.013713572	478
Ahsa2	94.12609903	-0.62244694	0.221998515	-2.803833808	0.005049893	0.045339561	638
H2-D1	1258.575038	-0.62243432	0.214887978	-2.896552549	0.003772874	0.038190638	1744
Tango2	90.65343293	-0.621493703	0.152072381	-4.086828255	4.37E-05	0.002468082	632
3830406C1	220.336222	-0.619599302	0.157864926	-3.924869943	8.68E-05	0.003760421	2292
Bola3	132.4884911	-0.618918963	0.167792083	-3.688606464	0.000225486	0.006981322	554
Ak8	45.42089957	-0.61884571	0.168340724	-3.676149742	0.000236781	0.007173997	1567
Med25	1292.999906	-0.618809604	0.163689733	-3.780381292	0.000156588	0.005501347	2619
Raly	677.0792581	-0.615471866	0.192900874	-3.190612116	0.001419717	0.021007959	1100

Mpp1	238.4162939	-0.613785552	0.182192625	-3.368882526	0.000754736	0.014339656	1558
Rnf170	75.29607274	-0.609593624	0.173351691	-3.516513855	0.000437254	0.010262997	741
Mapt	102.0790732	-0.609506234	0.141064139	-4.3207738	1.55E-05	0.001292914	418
Ptpn3	165.2293483	-0.609107226	0.12278343	-4.960825957	7.02E-07	0.000163285	800
Kif3a	722.359374	-0.608369231	0.111274841	-5.467266683	4.57E-08	2.39E-05	2290
Tent5b	240.6324415	-0.60784544	0.141503625	-4.295617453	1.74E-05	0.001393845	2287
Add3	646.9970856	-0.602865114	0.165867216	-3.634624895	0.000278385	0.007905353	4426
Tmem219	121.9693213	-0.601219351	0.198505843	-3.028723701	0.002455892	0.029364504	941
Noct	597.510977	-0.600391383	0.198712767	-3.021403163	0.002516061	0.029817416	2992
Tnip1	611.1145833	-0.599186564	0.14113125	-4.245598083	2.18E-05	0.001604563	2787
Nudt22	112.7025927	-0.597605562	0.17269526	-3.460463018	0.000539247	0.011694529	1073
Nedd4l	252.449942	-0.597398517	0.146153967	-4.087460157	4.36E-05	0.002468082	770
Mgat1	430.9981184	-0.596637475	0.152752943	-3.905898394	9.39E-05	0.003979996	2729
Tgfbrap1	923.2951612	-0.596168077	0.172963189	-3.446791659	0.000567286	0.012002273	5607
Islr	1546.54167	-0.595972633	0.200352275	-2.974623729	0.002933481	0.032593001	2076
Lrp3	1271.167347	-0.595125585	0.196646615	-3.02637086	0.002475085	0.029515642	3877
Wbp1	164.9612458	-0.591057957	0.137834478	-4.288172051	1.80E-05	0.001416865	981
Ppp1r3f	197.1820267	-0.591015902	0.145170835	-4.07117518	4.68E-05	0.002586832	4760
Frmpd4	339.7839732	-0.590616399	0.196134124	-3.011288337	0.002601417	0.030409811	5337
Ntrk3	741.6180538	-0.588221702	0.153415731	-3.834168089	0.00012599	0.004825202	2678
Fam110b	300.5662653	-0.587770788	0.151997925	-3.86696588	0.000110198	0.004397252	3119
Mdga1	1231.15303	-0.58653397	0.172006788	-3.409946646	0.000649756	0.013079907	7566
Csde1	3532.519595	-0.585971616	0.128498929	-4.560128391	5.11E-06	0.000620882	3849
Iffo1	786.171187	-0.583322988	0.143546877	-4.063641101	4.83E-05	0.002628818	2833
Mtmr14	119.6096542	-0.582040366	0.187436118	-3.105273264	0.001901033	0.025144079	1205
Serbp1	485.3698292	-0.581821973	0.210833837	-2.759623323	0.005786804	0.049267398	1617
Phpt1	208.1803426	-0.581254867	0.140763102	-4.129312716	3.64E-05	0.002194325	937
Ube2q2	782.0297833	-0.580987641	0.109509481	-5.305363838	1.12E-07	4.75E-05	1680
Rffl	101.9297809	-0.580824774	0.199291756	-2.914444568	0.003563221	0.036679233	1719
Hs3st6	95.09762231	-0.578992989	0.171562336	-3.374825742	0.000738624	0.0141067	1220
Slc4a1ap	288.699066	-0.578219753	0.159787959	-3.618669122	0.000296122	0.008239875	2499
Spop	1875.014775	-0.577842391	0.200241961	-2.885720794	0.003905184	0.038945728	3063
Lrp11	369.6143341	-0.576932757	0.106890024	-5.397442503	6.76E-08	3.20E-05	652
Dars2	245.6486393	-0.574633916	0.155526948	-3.694754673	0.000220099	0.006875681	3619
Dxo	72.03307748	-0.573939109	0.161473756	-3.554380135	0.000378871	0.009466978	584
Scube1	217.8351354	-0.573244711	0.206522188	-2.775705198	0.005508213	0.047878327	3991
Trmt9b	64.93725698	-0.572243877	0.205049637	-2.79075782	0.00525848	0.046470167	2573
Pomp	169.9333861	-0.572183883	0.192134363	-2.978040338	0.002900978	0.032395606	383
Sf3b5	582.0842146	-0.571442546	0.195165064	-2.927996096	0.003411543	0.03569455	3841
Fbxo25	85.30344867	-0.571391643	0.185174908	-3.08568611	0.002030831	0.02620648	737
Arpc2	1705.121884	-0.570520472	0.108212138	-5.27224101	1.35E-07	5.39E-05	1404
Mgat4a	1254.288323	-0.56742479	0.180123059	-3.150206268	0.001631552	0.022873468	7251
Tpk1	71.84549876	-0.567058094	0.205544599	-2.758808047	0.00580126	0.049342696	2671
Atxn1	381.6213458	-0.566720174	0.185177857	-3.060410041	0.002210342	0.027638699	3751
Asb8	63.8953237	-0.566528288	0.159077268	-3.561340319	0.000368966	0.009365826	1049
Cdk5r1	139.532909	-0.56531549	0.191484161	-2.952283301	0.003154334	0.034132485	659
Ybx1	945.4581028	-0.565144453	0.172398714	-3.278124533	0.001044993	0.017455589	1054
Sstr4	72.41727081	-0.564930431	0.191972291	-2.942770684	0.003252893	0.034771074	1424
Abcc5	154.239595	-0.564693185	0.197038461	-2.86590335	0.004158212	0.040461416	1728
Pcsk1n	45393.22252	-0.563519766	0.186529115	-3.021082076	0.002518731	0.029817416	2193
1810010H2	86.78859749	-0.563457007	0.181959772	-3.09660207	0.001957525	0.025606239	1677
Tmem11	327.4076877	-0.562800746	0.13544237	-4.155278329	3.25E-05	0.002023356	1457
Myoc	540.5765394	-0.562077294	0.196086798	-2.866471892	0.00415075	0.040427056	2074
Gm1673	214.0772746	-0.561920036	0.179367852	-3.132780092	0.001731591	0.023627998	511
Gas2l1	2091.981445	-0.561060199	0.108357452	-5.177864435	2.24E-07	7.62E-05	2847
Rnf24	971.2356045	-0.560514367	0.203070988	-2.760189296	0.005776788	0.049220247	5978
Wipi2	679.3254339	-0.560382253	0.126826247	-4.418503791	9.94E-06	0.000988581	1959
Epb41l3	288.2046088	-0.560357268	0.155065809	-3.61367391	0.000301889	0.008342261	559
Osbp2	92.1573675	-0.560204036	0.155160694	-3.61047648	0.000305635	0.008393032	478
Map1lc3b	414.598826	-0.560187965	0.119065946	-4.704854615	2.54E-06	0.000394012	766
Wnk2	620.3816889	-0.559200935	0.19556847	-2.859361409	0.004244949	0.040993941	6534
Eno1	8788.40437	-0.558060349	0.189785855	-2.940473868	0.003277106	0.034872626	1902
Tmem233	911.1384302	-0.557809216	0.138611982	-4.024249635	5.72E-05	0.002870131	702
Tbx3	943.7655312	-0.557005546	0.111516174	-4.994840897	5.89E-07	0.000141467	4856
Trappc6a	201.9201586	-0.556190167	0.178791813	-3.110825705	0.00186565	0.024851453	785
Rbfox2	291.1982242	-0.556054183	0.160015028	-3.47501226	0.00051083	0.01128417	1694
Ndrg4	835.0787726	-0.554771783	0.110060021	-5.040629472	4.64E-07	0.000121428	1029
Myo10	71.45845233	-0.554771464	0.168712457	-3.288266168	0.001008065	0.017078487	1030
Otud1	891.1199419	-0.554150821	0.154081946	-3.596468219	0.000322567	0.008607375	2832
Faim	74.86532581	-0.553944122	0.19379062	-2.858467151	0.004256932	0.041088037	803
Ppp1r2	546.8246442	-0.552543964	0.131960551	-4.187190503	2.82E-05	0.001856051	958

Cstf1	29.60998276	-0.552403908	0.17602373	-3.138235443	0.001699683	0.023358487	674
Syvn1	848.9902829	-0.551368541	0.135531849	-4.068184311	4.74E-05	0.00259186	3464
Sdcbp	121.2288124	-0.551226766	0.134125555	-4.109781815	3.96E-05	0.002318455	975
Atxn2	465.6890576	-0.550835993	0.157829094	-3.490078916	0.000482878	0.01088601	1434
Use1	690.9292205	-0.550056505	0.193655188	-2.840391276	0.004505823	0.042483059	854
Zfp622	937.4384663	-0.548022953	0.172277713	-3.181043815	0.001467454	0.021462618	2802
Septin10	64.24673233	-0.547518778	0.173622454	-3.153502136	0.00161324	0.022689097	1051
Cars2	145.9863091	-0.547373142	0.166807912	-3.281457909	0.001032719	0.017342822	1902
Tmem158	2973.516323	-0.545316454	0.139567871	-3.907177549	9.34E-05	0.003966643	1712
Marchf9	1131.254523	-0.545144341	0.170607851	-3.195306308	0.001396825	0.020824388	3037
Man2c1	331.9556411	-0.545104264	0.185447168	-2.939404621	0.003288435	0.034938625	3820
Wdr25	154.9540758	-0.544765126	0.186787654	-2.916494292	0.003539892	0.036524607	2657
Slc37a4	67.92806688	-0.544507805	0.171805476	-3.169327422	0.001527922	0.021974182	891
Ankrd46	160.930344	-0.544458102	0.178539395	-3.049512413	0.002292132	0.028180527	532
Scamp5	32.56603864	-0.544078589	0.174947883	-3.109946685	0.001871211	0.024880885	418
Fzd2	634.7604472	-0.543267772	0.136251163	-3.987252377	6.68E-05	0.003170171	3663
Senp6	229.2732655	-0.542870783	0.166684179	-3.256882474	0.001126431	0.018213873	1150
C1ql4	285.4115802	-0.542508375	0.116841322	-4.643120816	3.43E-06	0.000472983	1474
Mlst8	480.5697222	-0.542015416	0.187476409	-2.89111264	0.003838805	0.038599466	3358
Rab6b	2170.025166	-0.541003152	0.153764737	-3.518382454	0.000434186	0.010213279	716
Lsg1	257.1608534	-0.54082277	0.157923112	-3.424595448	0.000615716	0.01263024	3180
Ppp1r1a	826.2242485	-0.540583462	0.126730726	-4.265606922	1.99E-05	0.001498843	1306
Lmo1	305.4738425	-0.540491407	0.151101632	-3.577005761	0.000347553	0.008988321	912
Klhdc3	435.8441449	-0.540194726	0.156951151	-3.441801621	0.000577854	0.012109316	1954
Fam219b	269.6074484	-0.539681588	0.187694786	-2.875314753	0.004036249	0.039796747	3001
Esd	485.7912705	-0.538898923	0.1381774	-3.900051117	9.62E-05	0.004027264	1988
Nenf	678.7718585	-0.537129976	0.181195063	-2.964374235	0.003032992	0.033378095	742
Foxred1	274.7457788	-0.535384787	0.120774098	-4.432943788	9.30E-06	0.000943827	2289
Tfpt	99.11711335	-0.534928832	0.143613763	-3.724774144	0.00019549	0.006338405	1166
Tmpo	149.2417506	-0.534210602	0.188912681	-2.827817584	0.00468665	0.043460996	1854
Rnf19b	1216.805999	-0.533261599	0.148696686	-3.586237272	0.000335484	0.008810957	2532
Eya2	460.9253792	-0.53285999	0.149937511	-3.553880455	0.000379592	0.009466978	2447
Camta1	718.114343	-0.531857158	0.159566654	-3.333134743	0.000858733	0.015600915	4961
Hoxd1	1399.554504	-0.531643679	0.123826372	-4.293460822	1.76E-05	0.001396003	1862
Sh2d3c	1172.431614	-0.530847305	0.15976024	-3.322774829	0.000891268	0.015915763	3107
Sstr2	73.15388676	-0.530613771	0.145525448	-3.646192329	0.000266155	0.007684149	2143
Ncoa7	524.7081977	-0.530493295	0.152658741	-3.475027317	0.000510801	0.01128417	1445
Vim	4705.697599	-0.528827184	0.13051298	-4.05191256	5.08E-05	0.002709587	1777
Rbms1	519.0438552	-0.528391968	0.14428246	-3.662205152	0.000250053	0.007424876	1806
Rbfox1	120.7223642	-0.527296149	0.188032849	-2.804276771	0.005042959	0.04530317	1295
Hk1	4591.68791	-0.527283906	0.092262642	-5.71503152	1.10E-08	6.89E-06	3639
Vdac2	561.894164	-0.526260442	0.149491458	-3.520337885	0.000430997	0.010176584	874
Agpat3	552.2972587	-0.525049868	0.120961996	-4.340618437	1.42E-05	0.00123696	642
Srrm1	957.4119676	-0.524739753	0.163539558	-3.208641131	0.001333638	0.020237057	3152
Rpl19	169.2543486	-0.524525077	0.172040773	-3.048841667	0.002297255	0.028227732	737
Prr13	1186.43838	-0.523620093	0.107443754	-4.87343445	1.10E-06	0.000224272	1224
Msh3	259.9604832	-0.523395919	0.134728899	-3.884808104	0.000102411	0.004211869	3946
Syt7	1299.794735	-0.523033289	0.131345248	-3.982125724	6.83E-05	0.003225436	1605
Fam174a	915.6686469	-0.522471812	0.132852271	-3.932727747	8.40E-05	0.003688363	2112
Fcho1	418.3793683	-0.5204957	0.184474945	-2.821498061	0.004779993	0.043964932	3188
Shisal1	177.4181779	-0.519937316	0.163279738	-3.184334572	0.001450872	0.021290919	754
Syt3	397.1008347	-0.519315436	0.174554903	-2.97508363	0.002929087	0.032552402	2627
Znhit1	318.6499454	-0.517577304	0.160108117	-3.232673737	0.001226375	0.0192122	1208
Romo1	266.4237448	-0.514162546	0.123273191	-4.170919418	3.03E-05	0.001939388	569
Polr2i	104.5156983	-0.513911009	0.171767608	-2.991897105	0.002772497	0.031480586	508
Zfp516	147.2987145	-0.513021591	0.154459494	-3.321398884	0.000895674	0.015942686	765
Ssu72	492.5353587	-0.51282376	0.096850292	-5.295015092	1.19E-07	4.89E-05	874
Actn4	276.5915078	-0.511260219	0.162346942	-3.149182923	0.001637277	0.022883158	774
H2-T22	129.3955107	-0.510631769	0.178383975	-2.862542838	0.004202564	0.040700651	1534
Mafa	3052.458102	-0.509659538	0.098444217	-5.177140429	2.25E-07	7.62E-05	2739
Wdsub1	64.91924565	-0.508931893	0.181526191	-2.803627904	0.005053119	0.045356889	816
Isyna1	919.7136444	-0.508727439	0.152858203	-3.32810035	0.000874404	0.015767094	1836
Ccnt1	405.2035933	-0.508590589	0.153865545	-3.305422197	0.000948334	0.016545046	2175
Mapk10	1338.176063	-0.507846771	0.102217678	-4.968287108	6.75E-07	0.000157963	7198
Mms19	394.3926135	-0.50754095	0.144170923	-3.520411332	0.000430878	0.010176584	3162
Tbccd1	241.3004015	-0.505870779	0.1463704	-3.456100266	0.000548051	0.011763883	2645
Prdm4	435.4436252	-0.503967001	0.179034162	-2.814920887	0.004878924	0.044422605	3962
Map7d2	998.2291645	-0.503913547	0.148453646	-3.394416775	0.000687749	0.013555642	1312
Znrd2	362.0980735	-0.503785047	0.131484183	-3.831525848	0.000127351	0.004854312	699
Stard10	1984.682757	-0.502679406	0.100168358	-5.018345294	5.21E-07	0.000131689	1400
Pih1d1	164.1777047	-0.50254615	0.14276237	-3.520158357	0.000431289	0.010178009	1185

Map1lc3a	7611.82554	-0.50217541	0.099275819	-5.058385969	4.23E-07	0.000115461	1112
Kcnab2	2697.606786	-0.500012428	0.104366817	-4.790913862	1.66E-06	0.000295515	1325
Mtfr1l	939.4990227	-0.499711017	0.108959632	-4.586203231	4.51E-06	0.000573552	2025
Nfe2l1	4640.007915	-0.498555913	0.147595693	-3.377848648	0.000730553	0.014044055	4219
Ciz1	490.9272168	-0.498346633	0.163694297	-3.044373826	0.002331652	0.028483449	2973
Kcnn1	359.3572616	-0.497781016	0.133726972	-3.722368124	0.000197363	0.006368414	4047
Ccdc74a	222.7220727	-0.497577455	0.130813796	-3.803707797	0.000142546	0.005183663	1273
Irs3	79.48237837	-0.496987725	0.172228999	-2.885621632	0.003906415	0.038945728	2337
Stard13	849.5599421	-0.495705265	0.087268775	-5.680213413	1.35E-08	8.21E-06	3373
Zfand5	3787.695516	-0.495379561	0.084104126	-5.890074395	3.86E-09	2.93E-06	7385
Timm13	973.4477387	-0.494690123	0.103552097	-4.777210117	1.78E-06	0.000307081	1225
Csrnp3	133.4023201	-0.494481448	0.115641236	-4.275995871	1.90E-05	0.001464608	2958
Tmc6	236.0129044	-0.494315707	0.095755141	-5.162288931	2.44E-07	8.00E-05	940
Atg9a	2612.2353	-0.49397322	0.11128204	-4.438930298	9.04E-06	0.000928679	4041
Ebf3	374.8189325	-0.493796486	0.154678796	-3.192399327	0.001410961	0.020935845	1863
Cct4	590.8318423	-0.493737015	0.137806524	-3.582827592	0.000339895	0.008863408	1634
Epb41	707.1477318	-0.493656993	0.165579625	-2.981387319	0.002869456	0.032215435	2577
Nucks1	314.8382184	-0.493242907	0.162984481	-3.026318241	0.002475516	0.029515642	2375
Scara3	918.1476131	-0.492505005	0.150679592	-3.268558119	0.00108097	0.017779587	3597
Phlda1	657.7342307	-0.491840016	0.15281106	-3.218615306	0.001288112	0.019794418	1949
Hspa2	232.7359795	-0.491105666	0.132724301	-3.700194037	0.000215435	0.00679363	2524
Mapk8ip1	7583.795322	-0.490959888	0.102553741	-4.787342542	1.69E-06	0.000297211	3274
Pafah1b2	160.940533	-0.490597714	0.149769429	-3.27568662	0.001054054	0.017520418	794
Rbms3	2070.716759	-0.488956344	0.140235014	-3.486692312	0.000489034	0.010997629	3320
Pnrc1	6402.836625	-0.488897089	0.157934126	-3.095575984	0.00196431	0.025641599	1648
Mrpl54	544.208104	-0.488738678	0.104188575	-4.690904699	2.72E-06	0.000408944	604
Cdk5r2	9308.877272	-0.487312672	0.104518258	-4.662464543	3.12E-06	0.000445995	2799
Tspan5	360.6242204	-0.487072028	0.148743307	-3.274581137	0.001058187	0.017542689	6653
Zkscan14	178.4261294	-0.48674075	0.141804311	-3.432482043	0.000598083	0.01238063	1967
Osbpl1a	873.1393221	-0.486415474	0.143788786	-3.382847063	0.000717386	0.013894212	2674
Gm13889	3057.045983	-0.486410816	0.113256515	-4.294771186	1.75E-05	0.001395339	1240
Aip	1566.90514	-0.486266225	0.081018172	-6.0019402	1.95E-09	1.59E-06	1261
Wipf3	1681.468065	-0.486182187	0.139637175	-3.481753233	0.000498143	0.011145464	4102
Dazap1	451.4176224	-0.486114227	0.151504732	-3.208574538	0.001333947	0.020237057	1994
Aatk	3649.355083	-0.486071277	0.165215291	-2.942047773	0.003260496	0.03480821	5687
Ndufa7	884.6744522	-0.484722662	0.116059147	-4.176514084	2.96E-05	0.001916657	528
Trnp1	24214.1774	-0.484640144	0.10618318	-4.564189412	5.01E-06	0.000612811	1654
Znhit2	798.8916028	-0.483499197	0.099446582	-4.86189861	1.16E-06	0.000230578	1281
Ube2q1	517.2618667	-0.483488293	0.125658886	-3.847625162	0.000119268	0.004632185	1650
C1qtnf1	52.15911374	-0.483420072	0.170553404	-2.83442055	0.004590887	0.042962613	684
Tfcp2	170.0588171	-0.482571259	0.152299178	-3.168574279	0.001531886	0.022008842	1903
Klf5	1486.548831	-0.482086909	0.088422801	-5.452065571	4.98E-08	2.52E-05	3352
Kcnc3	1823.867075	-0.481937565	0.166137877	-2.900828967	0.00372177	0.037832971	2731
Kcnb1	1915.443972	-0.48147254	0.08410483	-5.724671694	1.04E-08	6.60E-06	4015
Nbl1	4455.33365	-0.4812641	0.089966847	-5.349349413	8.83E-08	3.96E-05	1793
Fam189b	697.0721923	-0.480292388	0.108673283	-4.419599493	9.89E-06	0.000985813	469
Mocs3	494.3514192	-0.480187251	0.170257769	-2.820354416	0.004797064	0.044010511	1973
Ubald2	816.6322271	-0.480050751	0.098680789	-4.864682923	1.15E-06	0.000229376	1482
Lpgat1	92.75990859	-0.479984769	0.165702221	-2.89667071	0.003771454	0.038190638	586
Tmem160	451.3183952	-0.479962706	0.134110729	-3.57885392	0.000345104	0.008956618	815
Smoc1	663.0472511	-0.478783261	0.142791123	-3.353032387	0.000799314	0.01485912	3496
Naa12	1498.990855	-0.478179189	0.130441531	-3.665850777	0.000246518	0.007367322	1683
Zswim9	321.1173205	-0.477593163	0.137386739	-3.47626827	0.000508443	0.011276581	3346
Map3k12	500.4466811	-0.477068598	0.167578645	-2.846834079	0.004415637	0.041976981	5185
Socs7	1848.275379	-0.477025597	0.113404353	-4.206413465	2.59E-05	0.001782337	7131
E2f1	967.7465517	-0.476764577	0.096854054	-4.922505134	8.54E-07	0.000182355	2732
Mrps12	597.7737785	-0.476364616	0.148849796	-3.20030412	0.001372826	0.020613496	722
Smad2	452.0789562	-0.475267648	0.127068314	-3.740253054	0.000183835	0.006114877	2410
Oplah	881.9939971	-0.474844663	0.168000165	-2.826453553	0.004706657	0.043591357	3970
Tlcd3b	1314.1203	-0.473222166	0.13105532	-3.610858105	0.000305186	0.008388017	1894
Zfyve9	576.7813282	-0.472949774	0.104511319	-4.525344977	6.03E-06	0.000705043	2170
Selenoh	181.3392141	-0.472645596	0.167183972	-2.827098743	0.004697184	0.043539403	747
Slc45a4	1517.889738	-0.472144973	0.097983594	-4.818612534	1.45E-06	0.000268168	4240
Comt	457.2536651	-0.471426548	0.144475222	-3.263026986	0.00110229	0.017995615	1978
Tbp	116.441713	-0.47118942	0.164299383	-2.867870899	0.004132441	0.040362112	1652
Scoc	178.2214001	-0.470376282	0.113986452	-4.126598135	3.68E-05	0.00220826	409
P2rx4	337.6576914	-0.469311735	0.136227025	-3.445070716	0.00057091	0.012032097	2702
Hlcs	364.1921711	-0.468250685	0.126348553	-3.706023321	0.000210539	0.006702638	4021
Cpsf6	293.5060249	-0.467704388	0.12339037	-3.790444818	0.000150378	0.005363044	2227
Chchd3	267.6293513	-0.465973807	0.124388187	-3.74612588	0.000179587	0.006004198	734
Mrpl55	186.9296249	-0.465890371	0.096890565	-4.808418353	1.52E-06	0.000274776	940

Naa80	275.0044347	-0.465810701	0.158938427	-2.930761999	0.003381317	0.035505043	787
Syt5	922.7050089	-0.464931665	0.14677603	-3.167626648	0.001536888	0.02205983	1750
Shox2	539.6186453	-0.463891828	0.140790699	-3.294903937	0.000984553	0.016855875	2645
Tapbp	2969.893071	-0.463782207	0.164679237	-2.816276146	0.004858389	0.044324354	2589
Fam241b	78.26010546	-0.463407103	0.129363435	-3.582210882	0.000340699	0.008878965	1769
Homer2	547.3767038	-0.463388742	0.143432808	-3.230702586	0.001234864	0.019303128	1736
Tlx3	2929.741317	-0.462211856	0.106172366	-4.353410153	1.34E-05	0.001190484	1533
Cadm1	591.3335213	-0.462070725	0.132197891	-3.495295736	0.000473537	0.010759196	1254
Bag5	349.5300924	-0.462054686	0.164262925	-2.812896988	0.004909738	0.044567706	1852
Garnl3	183.421479	-0.461787357	0.146009493	-3.162721467	0.001563018	0.022282129	543
Impdh1	890.121978	-0.461360406	0.129219338	-3.570366582	0.000356482	0.00912266	2580
Aen	488.9752469	-0.460451426	0.114799565	-4.010916137	6.05E-05	0.002967814	2007
Gsg1l	809.7611473	-0.460052595	0.105079213	-4.378150359	1.20E-05	0.001117241	3923
Smim14	115.2107625	-0.459969554	0.15996447	-2.875448234	0.004034543	0.039788847	520
Trip4	197.8095672	-0.459636016	0.106045119	-4.334343894	1.46E-05	0.001245637	1999
Arfgap3	215.8373794	-0.45945388	0.165516301	-2.775882965	0.005505202	0.047866392	2461
Dpf2	223.5832152	-0.459214217	0.10506486	-4.370768833	1.24E-05	0.001134021	3314
Amigo1	1154.413891	-0.459160016	0.090587093	-5.068713472	4.01E-07	0.000110746	5482
Mxi1	508.9899762	-0.45904625	0.103236266	-4.446559999	8.73E-06	0.000906909	2340
Asic3	1619.082054	-0.458984573	0.107961478	-4.25137354	2.12E-05	0.001569909	1593
Klc1	3009.075929	-0.45813959	0.064777651	-7.072494635	1.52E-12	1.59E-09	2268
Trappc14	508.8864391	-0.457854428	0.130357601	-3.512295596	0.000444254	0.010368566	2093
Phactr1	539.8368118	-0.457786456	0.088758211	-5.157680079	2.50E-07	8.14E-05	2056
Zfp575	135.8119878	-0.457277734	0.134500149	-3.399830679	0.000674276	0.01341382	2741
Cndp2	1102.207271	-0.457126299	0.09831181	-4.649759761	3.32E-06	0.000462359	2124
Ptms	12114.658	-0.457013336	0.136137521	-3.356997638	0.000787938	0.014713792	1149
Arf5	2963.70249	-0.45694738	0.100607346	-4.541888831	5.58E-06	0.00066789	1116
Rgmb	449.4661633	-0.456667034	0.111280776	-4.103736963	4.07E-05	0.00236104	742
Abi2	154.4690185	-0.456434595	0.157622404	-2.895746937	0.003782573	0.03826526	763
Rab24	1013.977274	-0.456361318	0.093870236	-4.861618967	1.16E-06	0.000230578	1168
Haus4	246.7161032	-0.456310779	0.081335919	-5.610200046	2.02E-08	1.17E-05	1587
Zfp503	1102.944699	-0.456308117	0.096672976	-4.720120729	2.36E-06	0.000370098	4216
Rap1gap2	284.5816421	-0.455421174	0.164423501	-2.769805846	0.005608972	0.048371604	764
Bag1	1753.941566	-0.454546462	0.158728748	-2.86366816	0.004187665	0.040645959	1336
Btbd10	146.893187	-0.454514509	0.158738347	-2.863293705	0.004192617	0.040649384	2394
Car10	759.9630903	-0.454123679	0.137707254	-3.297746977	0.000974639	0.016732194	3293
Card14	285.7964763	-0.45382157	0.125359972	-3.620147345	0.000294435	0.008203326	4206
Stx7	1786.730959	-0.45374447	0.137617601	-3.297139796	0.000976749	0.01676142	3435
Grin1	4141.906369	-0.453648638	0.125596846	-3.611942914	0.000303911	0.008371847	3838
Pgap4	589.8072433	-0.453225555	0.095018149	-4.769884063	1.84E-06	0.000308143	748
Zfp511	195.0085978	-0.452685156	0.105352283	-4.296870872	1.73E-05	0.0013923	1076
Tlx2	3029.913618	-0.452609593	0.116193068	-3.895323541	9.81E-05	0.004086775	1236
Cpne8	268.6746701	-0.452465607	0.148683569	-3.043144657	0.002341198	0.028551708	697
Add1	537.2323575	-0.45232926	0.118171569	-3.827733382	0.000129329	0.004889027	745
Pla2g6	869.5781419	-0.452199151	0.158707167	-2.849267358	0.004382004	0.041759503	3365
Tacc2	1355.639657	-0.451736284	0.136443319	-3.310798126	0.000930303	0.016360308	5804
Frat2	315.1319833	-0.451718789	0.114701749	-3.93820314	8.21E-05	0.0036358	2161
Ppp1r18	379.864757	-0.451521544	0.161270561	-2.799776601	0.005113798	0.045696778	3185
Rps6ka3	334.1424531	-0.451216255	0.158719571	-2.842852042	0.004471182	0.042256132	3671
Cabp1	7536.366358	-0.451175071	0.108926875	-4.141999596	3.44E-05	0.002114071	1621
Prdm15	277.8283975	-0.451018481	0.112004979	-4.026771692	5.65E-05	0.002860907	6194
Trmt112	606.6762002	-0.449814644	0.118548204	-3.794360694	0.000148024	0.005328106	985
Synj1	657.7969993	-0.448434804	0.13281245	-3.376451551	0.000734273	0.014074952	727
Hspb1	7234.082045	-0.448240134	0.115468929	-3.881911244	0.000103639	0.004250444	903
Grip1	218.7310447	-0.447650455	0.13760344	-3.25319232	0.001141162	0.018384464	4857
Tns2	400.1807034	-0.446906845	0.131860969	-3.389227675	0.000700898	0.013695545	4268
Ankrd17	515.216426	-0.446326193	0.140398928	-3.178985767	0.001477913	0.021543921	9311
Csnk1g2	1622.498112	-0.445805334	0.092683648	-4.80996747	1.51E-06	0.000274776	1825
H1f10	1760.10413	-0.444855615	0.14645365	-3.037518114	0.002385351	0.028906269	1217
Gpx1	735.1992463	-0.444659807	0.120610912	-3.686729505	0.000227155	0.007011665	653
Sirt2	701.6168691	-0.444475342	0.137615572	-3.2298332	0.001238625	0.019331249	1843
Chpf	3960.330706	-0.444283885	0.11210237	-3.963197981	7.40E-05	0.003418841	2892
Mettl27	264.4506397	-0.443882736	0.092100787	-4.819532513	1.44E-06	0.000268065	2808
Hdac9	209.6449227	-0.443766024	0.151892167	-2.921585967	0.003482542	0.03617055	1683
Cstf2	88.42382418	-0.443605195	0.141176298	-3.142207302	0.001676793	0.023200707	574
Gabra1	299.9950427	-0.443292906	0.143765837	-3.083437037	0.002046244	0.0263127	1116
Serpinb6a	105.6611193	-0.442541766	0.149514279	-2.959862892	0.00307776	0.033696466	437
Marcks	6142.811495	-0.442210244	0.15077864	-2.932844103	0.003358725	0.035343787	4048
Cuedc2	845.4565771	-0.441927666	0.123712503	-3.572215062	0.000353975	0.009081164	1050
Keap1	1206.372825	-0.441723149	0.133146535	-3.317571493	0.000908037	0.016097514	3151
Lsm4	520.5814406	-0.441164002	0.109396265	-4.032715403	5.51E-05	0.00281213	925

Vstm2l	7749.530733	-0.440970099	0.108262422	-4.07315939	4.64E-05	0.002568113	1363
Cct7	1386.193268	-0.439612498	0.120949427	-3.634680299	0.000278326	0.007905353	1544
Basp1	7885.543538	-0.439484985	0.128505989	-3.419957223	0.00062631	0.012795405	1855
Ndfip2	1816.951898	-0.438702401	0.123439716	-3.55398096	0.000379447	0.009466978	1695
Hmbs	294.0004744	-0.437884859	0.155248313	-2.820545032	0.004794214	0.044010511	1605
Uqcr11	1230.357068	-0.437543697	0.123314169	-3.548202953	0.000387869	0.009575225	445
Ccm2	417.1420457	-0.437223002	0.118880413	-3.677838857	0.000235218	0.007156665	1551
Fkbpl	189.7062842	-0.437221491	0.111608762	-3.917447721	8.95E-05	0.003853567	1270
Papln	688.3259327	-0.437138139	0.131686635	-3.319533071	0.000901681	0.016010669	4260
Med16	1081.96452	-0.437062034	0.153699094	-2.843621407	0.004460401	0.042172375	2950
Eef1b2	1020.194056	-0.436524332	0.131058077	-3.330770162	0.000866061	0.015672695	1939
Cdk9	770.266952	-0.436314619	0.138985284	-3.139286453	0.001693598	0.023319461	1733
Arf6	2583.288257	-0.436217047	0.112144986	-3.889759714	0.000100344	0.004157967	1534
Ldb1	1027.225671	-0.43620353	0.131729273	-3.311363667	0.000928425	0.01633865	2108
Tspan8	360.6618548	-0.436193379	0.15370924	-2.837782422	0.004542814	0.042694486	1244
Pdf	206.6581438	-0.436165506	0.143470583	-3.040104078	0.002364964	0.028746377	1306
Mfsd11	180.9404235	-0.436090763	0.156259057	-2.790819121	0.005257484	0.046470167	1194
Cbr3	116.3282851	-0.436031312	0.132077531	-3.301328465	0.000962282	0.016695623	1170
Tkt	5440.172254	-0.436015216	0.074666157	-5.839529389	5.23E-09	3.84E-06	3223
Gm42517	884.6881569	-0.435840088	0.096417618	-4.520336615	6.17E-06	0.000712457	1850
Lsm14b	1537.196884	-0.43575223	0.106039094	-4.109354529	3.97E-05	0.00231966	2534
Nr2f6	1544.791586	-0.434749408	0.151962125	-2.860906356	0.004224318	0.040853969	2218
Zfp865	791.8296458	-0.434571732	0.154498634	-2.812786887	0.004911419	0.044567706	2751
Etv1	508.4111457	-0.434522073	0.127970702	-3.395480889	0.000685082	0.013530823	3981
Stambpl1	195.8582861	-0.434514257	0.132401437	-3.281794106	0.001031489	0.017340167	1988
Pbx3	270.6427688	-0.433860128	0.129201017	-3.358024084	0.000785018	0.014693104	2734
Tenm2	800.3070732	-0.432400436	0.107511575	-4.021896561	5.77E-05	0.002884274	8298
Iscu	109.6141952	-0.432264839	0.143692062	-3.008272217	0.002627377	0.030623706	1116
Arl2	798.9572801	-0.430485226	0.108897086	-3.953138149	7.71E-05	0.003514142	1286
Runx3	476.1649194	-0.43020928	0.104138112	-4.131141525	3.61E-05	0.002185934	3884
Ap1s2	327.8775446	-0.430136459	0.115163306	-3.735013137	0.000187705	0.006195546	1785
Cox6a1	2614.786238	-0.430116255	0.071702352	-5.998635189	1.99E-09	1.59E-06	571
Arid1b	1870.232128	-0.429859384	0.138554177	-3.102464261	0.001919167	0.025314497	9888
Dgkh	2339.22369	-0.42982302	0.125932253	-3.413128977	0.000642215	0.012975923	3319
Zfand2b	330.9860379	-0.42961816	0.094176852	-4.561823346	5.07E-06	0.000617596	1206
Kcnip2	311.5207409	-0.429612543	0.127884107	-3.359389632	0.000781148	0.014657786	1005
Syne1	802.3305711	-0.429502757	0.117068591	-3.668812892	0.000243679	0.007307887	3894
S100a6	1118.567514	-0.429330647	0.124788663	-3.440461959	0.000580722	0.012140488	464
Barx2	89.65769273	-0.428755325	0.127513542	-3.36242973	0.000772598	0.014528343	1968
Ntn4	472.3640846	-0.427885819	0.084832887	-5.043867229	4.56E-07	0.00012049	3646
Insyn1	5290.295789	-0.42769046	0.105117236	-4.068699642	4.73E-05	0.00259186	2139
Timm10b	129.3973225	-0.42757496	0.150487759	-2.841260732	0.004493556	0.042394673	694
Frmpd1	457.2833106	-0.427308279	0.130793306	-3.267050077	0.001086745	0.017832269	671
Thyn1	234.36243	-0.427294667	0.147976455	-2.887585507	0.003882111	0.038825523	918
Fryl	100.5129447	-0.427092195	0.14254875	-2.996113219	0.002734449	0.031291393	723
Gadd45b	180.1629727	-0.426747193	0.141679911	-3.012051525	0.002594885	0.030365755	1284
Irf2bpl	6392.42853	-0.426469918	0.124128019	-3.435726449	0.000590967	0.012290388	4098
Get4	436.5442556	-0.426438576	0.114342267	-3.729492052	0.000191866	0.006284249	1180
Mrps24	574.2862236	-0.426366959	0.154267736	-2.7638116	0.005713052	0.048919924	995
Jund	12592.44069	-0.426153242	0.144744889	-2.944167812	0.003238244	0.03471833	1667
Ppp1r14b	808.9307267	-0.426126172	0.10631035	-4.00832256	6.12E-05	0.002993907	999
Dnajc9	380.1202722	-0.425834258	0.134376189	-3.16897109	0.001529796	0.021993946	1631
Ltbp3	4039.776891	-0.425631521	0.129773419	-3.279805105	0.001038788	0.017391593	5190
C2cd2l	959.0480066	-0.425236989	0.105230206	-4.041016395	5.32E-05	0.00277096	2808
Tbcc	618.0125096	-0.425139947	0.101959668	-4.169687445	3.05E-05	0.001941505	1164
Diras1	10708.17921	-0.424843642	0.104183762	-4.077829715	4.55E-05	0.002529826	2953
Prrxl1	1266.134683	-0.424573156	0.111252629	-3.816297742	0.000135469	0.005026072	2371
Atp5j2	424.9823268	-0.424160162	0.114469472	-3.705443503	0.000211021	0.006706153	540
Zfp771	1604.556824	-0.424033406	0.141405619	-2.998702661	0.002711318	0.031154718	1484
Tox3	129.3607246	-0.423862444	0.114555994	-3.700045982	0.00021556	0.00679363	1109
BC031181	356.8566297	-0.423640763	0.128907258	-3.286399619	0.001014769	0.017159359	979
Arl6ip4	617.9804897	-0.423526844	0.095500642	-4.434806243	9.22E-06	0.000940047	1192
Gnai2	878.1331201	-0.423391289	0.134454043	-3.148966584	0.001638489	0.022883158	1777
Hs3st2	2344.36308	-0.422742275	0.130779276	-3.232486745	0.001227178	0.0192122	2278
Nek7	5315.157651	-0.422309383	0.097622628	-4.325937459	1.52E-05	0.001271924	5258
Rem2	1320.19683	-0.422027387	0.115393328	-3.657294511	0.000254891	0.007505896	1884
Dnaja1	70.79087073	-0.421730246	0.138511259	-3.044736216	0.002328845	0.028472652	916
Gtf3a	376.1133619	-0.421573791	0.140614694	-2.998077793	0.002716883	0.031154718	1298
Atp13a2	4974.845487	-0.421294615	0.073203364	-5.755126458	8.66E-09	5.60E-06	3882
Bbc3	282.8555328	-0.420738621	0.122559232	-3.432941074	0.000597072	0.01237053	1814
B9d2	186.6311003	-0.420253097	0.151760801	-2.7691808	0.005619744	0.048388572	995

Ptov1	3820.610613	-0.42001175	0.112955684	-3.718376405	0.000200507	0.006454906	1883
Lamtor4	413.099469	-0.419931644	0.108590834	-3.867100277	0.000110137	0.004397252	877
Uros	289.4396042	-0.419931302	0.129468662	-3.243497671	0.001180718	0.018753705	1644
Npm1	525.2956292	-0.419685868	0.148326008	-2.829482662	0.004662333	0.04334413	966
Tmed7	1010.382733	-0.419121025	0.1307857	-3.204639539	0.001352317	0.020410102	1348
Ptp4a3	1137.277761	-0.419078065	0.129972998	-3.224347143	0.001262602	0.019614952	1680
Pou4f1	6360.272455	-0.419050577	0.099136152	-4.227020801	2.37E-05	0.001668456	4445
Fez2	525.3002698	-0.418760786	0.139560217	-3.000574191	0.002694711	0.031078953	2001
Cacnb3	6481.83962	-0.418701381	0.100806964	-4.153496589	3.27E-05	0.002030408	2477
Pfdn4	85.53748682	-0.418586695	0.152073227	-2.752533787	0.005913604	0.049988775	736
Phf23	675.5901817	-0.41783456	0.120842942	-3.457666235	0.000544876	0.01173815	1797
Chchd2	6895.057283	-0.417428374	0.119080669	-3.505425154	0.000455879	0.0105377	915
Park7	805.7649365	-0.417323506	0.096303881	-4.333402799	1.47E-05	0.001248556	908
Ncoa2	1417.989815	-0.416960639	0.149005529	-2.79828972	0.005137401	0.045786708	4897
Ank2	1455.228747	-0.416930274	0.137986199	-3.021536035	0.002514957	0.029817416	3536
Pim1	801.0014912	-0.416720128	0.111261687	-3.745405435	0.000180103	0.00601688	2698
Satb1	813.0608369	-0.416389167	0.080401346	-5.178883056	2.23E-07	7.62E-05	5993
Ttc33	136.1830077	-0.415982086	0.147911682	-2.812368024	0.00491782	0.044616582	1583
Cacul1	381.7812817	-0.415373249	0.102793485	-4.04085189	5.33E-05	0.00277096	1305
Cltb	4149.781368	-0.415139942	0.076837724	-5.402814137	6.56E-08	3.17E-05	1004
Sucla2	781.3545848	-0.415004034	0.14019938	-2.960098927	0.003075403	0.033693018	1545
Psd3	246.1526494	-0.414794169	0.129918597	-3.192723583	0.001409378	0.020919412	1739
Gramd1b	735.264602	-0.414716273	0.130419531	-3.179863254	0.001473446	0.021507317	2781
Pik3r1	1346.562928	-0.413991004	0.131725946	-3.1428205	0.001673284	0.02318407	2923
Zfr2	2225.435924	-0.413942855	0.0996271	-4.154922256	3.25E-05	0.002023498	3349
Snrnp25	195.1249753	-0.413910229	0.077260233	-5.35735158	8.45E-08	3.83E-05	821
Sap30l	3359.980173	-0.413683839	0.101550091	-4.073692441	4.63E-05	0.00256547	1151
Tmem51	372.1457012	-0.413547232	0.141061155	-2.931687556	0.003371257	0.035428477	1875
Yif1b	241.34538	-0.412927758	0.114497214	-3.606443718	0.000310422	0.008471577	1111
Fam131a	211.6716568	-0.412686183	0.103238526	-3.997404821	6.40E-05	0.003097415	962
Ddit3	243.1100639	-0.411832537	0.128843504	-3.196377969	0.001391647	0.020782524	890
Hipk1	1378.940478	-0.411497079	0.111455263	-3.692038103	0.000222464	0.006908853	4185
Adrb1	201.6771294	-0.411467774	0.128965343	-3.190529827	0.001420122	0.021007959	2952
Anxa6	3997.102919	-0.410010327	0.072614567	-5.646392215	1.64E-08	9.70E-06	2471
Ppcs	266.9661105	-0.409967341	0.139266255	-2.943766529	0.003242445	0.034735403	1443
Ppp1r35	368.1189541	-0.409894869	0.124585149	-3.290078092	0.001001596	0.017041471	956
Atox1	688.034358	-0.409859666	0.09793962	-4.184819858	2.85E-05	0.001869936	541
Ccdc149	710.2755916	-0.40907261	0.106492715	-3.841320144	0.000122374	0.004730539	3295
Dync2i2	365.1593602	-0.408253123	0.145659549	-2.802789965	0.005066265	0.045410468	1814
Bex2	1571.812873	-0.408199146	0.138947664	-2.937790631	0.003305602	0.035027906	903
Sac3d1	683.5269594	-0.408051596	0.132776194	-3.073228606	0.002117562	0.026914893	1442
Farsa	495.9160584	-0.40729066	0.088266086	-4.614350543	3.94E-06	0.00052535	1826
Rbm5	1465.644593	-0.407239813	0.125344887	-3.248954327	0.001158301	0.018573122	3205
Snx21	644.6092401	-0.406865271	0.112680292	-3.61079353	0.000305262	0.008388017	2590
Sulf2	1521.319496	-0.406352952	0.121774975	-3.336916743	0.000847133	0.015473291	3808
Ube2j2	282.2342398	-0.406238462	0.090329832	-4.497279056	6.88E-06	0.00077591	3483
Nrxn2	10326.43903	-0.406132684	0.093632594	-4.337513968	1.44E-05	0.001244005	3503
Acsf3	341.820669	-0.405956714	0.132929805	-3.053917919	0.002258739	0.02797752	2171
Zfp414	374.6796262	-0.405931261	0.099975938	-4.060289588	4.90E-05	0.002653704	1197
Sbno2	313.2839086	-0.405917329	0.141940412	-2.859772802	0.004239446	0.040968024	3903
Hras	1616.029468	-0.404648922	0.078879961	-5.129933073	2.90E-07	9.17E-05	1199
Fndc4	543.4659533	-0.404126591	0.091738238	-4.405214241	1.06E-05	0.001030198	1375
Epdr1	587.4470657	-0.403718219	0.102643347	-3.93321369	8.38E-05	0.003685043	563
Skor2	1403.950986	-0.403267272	0.125034285	-3.225253557	0.001258611	0.019573701	3141
Slc5a5	580.5394165	-0.403229637	0.098209533	-4.105809518	4.03E-05	0.002346167	2928
Lmo4	1147.742384	-0.403156948	0.102912438	-3.917475453	8.95E-05	0.003853567	1669
Cnih2	518.3225871	-0.402484919	0.138378976	-2.908569865	0.00363086	0.037183968	1343
Exosc5	443.3452998	-0.402162101	0.091698454	-4.385702095	1.16E-05	0.001095451	1020
D030056L2	400.8883936	-0.401678336	0.136466018	-2.94343121	0.00324596	0.034747653	1677
Rab43	634.8506453	-0.401671005	0.144585059	-2.778094824	0.005467866	0.047650471	4605
Arfgap1	244.0432898	-0.401530895	0.141163322	-2.844442087	0.004448927	0.042154545	1209
Camk2n2	3609.396106	-0.401414931	0.126859242	-3.16425453	0.001554808	0.022214882	1291
Cyb561d2	120.9621175	-0.400966131	0.121730656	-3.293879646	0.000988148	0.016907178	1786
Psen2	1183.652628	-0.40084128	0.118540216	-3.381479233	0.000720967	0.013932882	1997
Brd9	423.0109843	-0.400744055	0.122889564	-3.261009648	0.001110163	0.018087928	2407
Irf2bp1	1984.330004	-0.400576803	0.140643385	-2.848173793	0.004397091	0.041861863	2734
Ddx54	2195.962027	-0.40019974	0.098615176	-4.058196279	4.95E-05	0.002662199	4322
Ssbp4	698.8489359	-0.399976155	0.10562245	-3.78684792	0.00015257	0.005409482	1475
Egfl7	127.4721738	-0.39981193	0.13210477	-3.026476101	0.002474224	0.029515642	1163
Acbd6	964.7735918	-0.399539675	0.141650811	-2.820595754	0.004793457	0.044010511	993
Faap20	359.3356148	-0.399531841	0.128043986	-3.120270254	0.001806852	0.024360803	1076

Dlg4	2160.855687	-0.399115462	0.129703438	-3.077138658	0.002089981	0.026695525	3488
Mrfap1	6212.025139	-0.398857725	0.124451569	-3.204923243	0.001350985	0.020396997	1648
Mff	169.3128848	-0.398481284	0.125657785	-3.171162726	0.001518301	0.021895287	440
Igfbp4	4828.231713	-0.398338704	0.126140926	-3.157886309	0.001589175	0.02249043	3831
Npdc1	7711.262328	-0.398230037	0.095341811	-4.176866701	2.96E-05	0.001916512	1485
Ndufa10	3560.136815	-0.397834375	0.058988314	-6.744291373	1.54E-11	1.50E-08	2020
Sh3glb2	2014.516338	-0.397617308	0.090761988	-4.380879221	1.18E-05	0.001108043	1733
Swi5	1610.692476	-0.397567627	0.118148044	-3.364995429	0.000765449	0.014455069	768
Stoml2	418.3740755	-0.397221326	0.112661573	-3.525792478	0.000422218	0.010094501	1874
Cox17	154.7308942	-0.397122271	0.126335286	-3.143399476	0.001669978	0.023169371	444
Gm49027	326.6260051	-0.396693962	0.098150214	-4.041702453	5.31E-05	0.002770763	346
Pacsin2	339.1691605	-0.396657028	0.117812631	-3.366846347	0.00076033	0.014402381	823
Fxyd7	3937.569852	-0.396613352	0.120919565	-3.27997668	0.001038157	0.017387641	693
Pfdn6	308.1945662	-0.396278447	0.141959566	-2.791488162	0.005246628	0.04641207	807
Flad1	402.7324525	-0.396259577	0.141438856	-2.801631657	0.005084489	0.045527719	2238
Itm2c	11995.53736	-0.396069508	0.121745483	-3.253258351	0.001140897	0.018384464	2230
Azin2	1772.121731	-0.396035433	0.094837196	-4.175950478	2.97E-05	0.001918583	2063
Ppp1r13l	345.3347699	-0.395873938	0.082823113	-4.779751964	1.76E-06	0.000306205	3130
Spg7	891.7976759	-0.395732561	0.090232111	-4.385717641	1.16E-05	0.001095451	2481
Cbr1	1115.6055	-0.395703669	0.084743917	-4.669405008	3.02E-06	0.000436864	1237
Rhoc	671.7806382	-0.395417411	0.131175391	-3.014417622	0.002574731	0.030206993	959
Abhd17a	3828.694617	-0.39508076	0.104223342	-3.790712833	0.000150216	0.005363044	1468
Dut	106.235013	-0.3948939	0.131453402	-3.004059949	0.002664028	0.030870849	1020
1110065P2	261.0402617	-0.3946879	0.134867906	-2.926477553	0.003428242	0.035818127	762
Ablim1	293.9892808	-0.394288724	0.094889018	-4.155261929	3.25E-05	0.002023356	1251
Pkdcc	936.1460708	-0.393575576	0.142563229	-2.760708906	0.005767606	0.049189678	2446
Nr1h2	786.0110786	-0.393550144	0.105969563	-3.713803577	0.000204167	0.006537665	1948
Chmp4b	4556.231204	-0.393071886	0.08070534	-4.870456949	1.11E-06	0.00022558	1616
Atp6v1h	713.9040258	-0.392532259	0.138083902	-2.842708345	0.004473198	0.042260643	2049
Rps24	797.9386963	-0.392283654	0.125495036	-3.125889801	0.00177268	0.024032026	525
Ccdc120	296.1355574	-0.391989297	0.100196568	-3.912202811	9.15E-05	0.003907632	3658
Dnajb2	218.6100048	-0.391978275	0.129473078	-3.027488649	0.00246595	0.029452725	499
Ccdc85b	2038.278972	-0.391807978	0.123175777	-3.180884963	0.001468259	0.021464956	4715
Micu3	975.9168892	-0.391736487	0.078801047	-4.971209141	6.65E-07	0.000156432	2219
Sarm1	2460.244184	-0.391698728	0.094795119	-4.132055884	3.60E-05	0.002180258	4083
Etv3	424.3272323	-0.391627934	0.129911886	-3.014565834	0.002573474	0.030203621	5309
Dmtn	1992.076816	-0.391350715	0.099681135	-3.926025885	8.64E-05	0.00374813	2643
Vasp	4244.8647	-0.391298226	0.079884329	-4.898310235	9.67E-07	0.000204782	2215
Hnrnpf	395.8806645	-0.391097293	0.1408095	-2.777492236	0.005478015	0.04771962	2401
C1qtnf12	398.763182	-0.391006975	0.127800865	-3.059501785	0.002217055	0.027691139	1315
Cdk12	770.8540329	-0.390894604	0.138635411	-2.81958701	0.004808549	0.044058267	5143
Dusp22	124.1530875	-0.390334602	0.116684898	-3.345202408	0.000822225	0.015169593	1161
Glo1	321.9937472	-0.38992889	0.136438582	-2.857907826	0.004264442	0.041124416	898
Pfn1	4486.058878	-0.38973244	0.095752459	-4.070208155	4.70E-05	0.002591075	861
Paics	1014.833561	-0.389608595	0.098115825	-3.970904746	7.16E-05	0.003334643	1862
Hnrnpa0	2085.150242	-0.389504821	0.138269038	-2.817006795	0.00484735	0.044251193	2678
Phf1	1381.419806	-0.389380732	0.107867032	-3.609821518	0.000306408	0.008403754	2522
Kcng2	385.8095758	-0.38897137	0.10980338	-3.542435299	0.000396451	0.009726569	2813
Rc3h1	808.5647552	-0.388881331	0.122343911	-3.178591615	0.001479924	0.02154617	3476
Ntmt1	250.7094836	-0.388536326	0.136022457	-2.856413082	0.004284573	0.041194034	1387
Myl12b	2218.439824	-0.388469738	0.075905939	-5.117777914	3.09E-07	9.71E-05	1001
0610012G(	855.178108	-0.388292828	0.120586223	-3.220043026	0.001281714	0.019780656	1445
Ndufab1	890.0913114	-0.388251478	0.093196917	-4.165926206	3.10E-05	0.001964431	1416
Msl1	2889.67415	-0.388059305	0.109776756	-3.534986083	0.000407797	0.009895745	4593
Gipc1	2740.779795	-0.387720776	0.078747798	-4.92357607	8.50E-07	0.000182244	1522
Rapgef1	638.5166899	-0.387549771	0.099429478	-3.89773513	9.71E-05	0.004057841	3908
D6Wsu163	521.8791123	-0.387304103	0.094684875	-4.090453738	4.31E-05	0.002448675	2501
Scyl1	2627.825596	-0.387160471	0.098003102	-3.95049199	7.80E-05	0.003536843	2731
Myl6	2508.547317	-0.387044675	0.116173497	-3.331609054	0.000863455	0.01564789	691
Klc2	5269.8861	-0.387028707	0.088577223	-4.369393093	1.25E-05	0.001136453	2940
Ric3	191.1184332	-0.386935084	0.125741465	-3.077227429	0.002089358	0.026695331	1633
Pdzd4	1675.865307	-0.386727924	0.073208833	-5.282530894	1.27E-07	5.19E-05	3970
Unc50	331.6293925	-0.386456074	0.133603171	-2.892566626	0.003821081	0.038516104	1414
Insm2	676.8871115	-0.386394113	0.10548771	-3.662930161	0.000249347	0.007412117	2660
Tedc2	262.3100007	-0.386274796	0.096453202	-4.004789742	6.21E-05	0.003028884	2166
Tle5	16826.20725	-0.386235556	0.092261915	-4.186294611	2.84E-05	0.001860607	1411
Tsc22d2	1892.896361	-0.386059884	0.134242747	-2.875834206	0.004029613	0.039762016	4659
Nedd8	888.7227866	-0.385621657	0.111876698	-3.44684519	0.000567173	0.012002273	824
Ccnl1	252.3103744	-0.385591471	0.132874058	-2.901931921	0.003708691	0.037726194	2169
Cpne6	1928.432798	-0.385553739	0.118523739	-3.252966378	0.00114207	0.018392349	2178
Sf3b4	1191.769704	-0.385547116	0.118659294	-3.249194419	0.001157323	0.018573122	1915

Bckdk	519.0238796	-0.385486643	0.136745144	-2.81901522	0.004817123	0.044090688	3405
Jmjd1c	411.0189438	-0.385401402	0.123702073	-3.115561381	0.001835951	0.024609025	3076
Etfb	1118.070042	-0.385265062	0.114003319	-3.379419699	0.00072639	0.013995644	893
Eid2	871.6101519	-0.385254602	0.095112398	-4.050519289	5.11E-05	0.002716798	1392
Rprm	856.8180999	-0.385220367	0.096487611	-3.992433478	6.54E-05	0.003142353	1460
Synm	10008.14895	-0.384805164	0.075617871	-5.088812477	3.60E-07	0.000103257	6953
Nudt18	218.8189789	-0.384737638	0.119465145	-3.220501166	0.001279667	0.019768288	3926
Selenom	3612.925183	-0.384633591	0.093990601	-4.092255878	4.27E-05	0.002444076	707
Asl	369.3952314	-0.384103669	0.099843905	-3.847041721	0.000119553	0.004635036	1824
Selenow	5919.874301	-0.384013601	0.095955643	-4.001990807	6.28E-05	0.00305816	736
Tlnrd1	2063.729882	-0.383613138	0.103894977	-3.692316498	0.000222221	0.006908853	4433
Adrm1	3654.491088	-0.383597963	0.090570482	-4.235353006	2.28E-05	0.001633958	1535
Atp5g3	1784.834409	-0.38324116	0.096827682	-3.957971005	7.56E-05	0.003472598	714
Thop1	2020.729595	-0.38321822	0.088311076	-4.339412854	1.43E-05	0.001241307	2832
2310022A1	489.5336562	-0.383145453	0.117024243	-3.274069056	0.001060107	0.017558455	2701
Adra2c	1764.54452	-0.3831214	0.11875369	-3.226185222	0.001254521	0.019516997	3445
Prdx5	1831.073196	-0.382943951	0.076291028	-5.01951488	5.18E-07	0.000131646	1262
Foxd3	990.1958882	-0.382909386	0.098413916	-3.89080531	9.99E-05	0.004143995	2324
Fam168b	387.868583	-0.382810454	0.106442452	-3.596407709	0.000322642	0.008607375	844
St6galnac6	419.5792483	-0.38255051	0.088310286	-4.331890758	1.48E-05	0.001252319	2402
Cant1	899.5443034	-0.382480661	0.117480897	-3.255683857	0.001131196	0.018270774	2873
Ndufv1	1867.248237	-0.382216617	0.110258028	-3.466564951	0.000527154	0.011533023	1661
Samd14	6592.812534	-0.382173531	0.075067826	-5.091042977	3.56E-07	0.000103257	3318
Map3k9	2993.977814	-0.382113646	0.116190382	-3.288685689	0.001006564	0.017072014	3303
Zfp637	386.7424477	-0.381642598	0.122729518	-3.109623536	0.001873259	0.024896568	1098
Tbc1d1	787.2361558	-0.380834539	0.107804131	-3.532652555	0.000411413	0.009949265	4760
Lactb	232.8693799	-0.380722605	0.122751109	-3.1015818	0.001924897	0.025330167	2031
Nefm	100568.2914	-0.38061985	0.118614421	-3.208883439	0.001332515	0.020236277	3334
Fis1	1615.072282	-0.380422	0.095692422	-3.975466314	7.02E-05	0.003292289	786
Jph4	8415.472025	-0.380210379	0.088898422	-4.276908087	1.90E-05	0.00146246	4489
Sbk1	583.1639506	-0.379578056	0.117768423	-3.223088561	0.001268163	0.019666597	1512
Fam89b	2904.814774	-0.379408856	0.093481331	-4.058659113	4.94E-05	0.002662199	1197
C77080	8576.525985	-0.379378369	0.096104676	-3.947553713	7.90E-05	0.003567527	5187
Mob2	652.6664026	-0.379180142	0.103722818	-3.655706124	0.000256475	0.007533413	1490
Abhd8	5619.126257	-0.378829935	0.118528237	-3.196115489	0.001392913	0.020787318	1978
Fkbp8	9004.258882	-0.378663935	0.088140687	-4.296130955	1.74E-05	0.001393845	1698
Ndufa5	141.1372597	-0.378585792	0.119553938	-3.16665264	0.001542044	0.022099394	875
Suv39h1	325.8263017	-0.378526266	0.12210928	-3.099897623	0.001935875	0.02543657	2778
Taf11	253.918756	-0.378460502	0.124631283	-3.036641304	0.0023923	0.028958548	1220
Cisd3	813.2225385	-0.378386049	0.083637148	-4.524138573	6.06E-06	0.000706459	737
H2az2	389.6275005	-0.378135114	0.095721659	-3.950361039	7.80E-05	0.003536843	1633
Arhgap27	2297.149183	-0.378120981	0.099751635	-3.790624401	0.000150269	0.005363044	4261
Pex6	1777.326683	-0.378065673	0.130736576	-2.89181256	0.003830264	0.038570213	3200
Dctn1	12975.51014	-0.378031095	0.08342068	-4.531623262	5.85E-06	0.00069363	4205
Ndufs8	365.9073243	-0.377944698	0.122295845	-3.090413227	0.001998782	0.025947377	983
Sod1	2927.372397	-0.377788843	0.087074696	-4.338675415	1.43E-05	0.001241818	641
Spock1	1178.532219	-0.377730056	0.128357712	-2.942792052	0.003252668	0.034771074	2005
Inafm1	960.6320651	-0.377694756	0.078174129	-4.831454626	1.36E-06	0.00025575	1605
Micu1	2115.460554	-0.377534689	0.131716765	-2.866261478	0.00415351	0.040444978	2336
Mrps34	724.7148052	-0.37750209	0.104444477	-3.614380594	0.000301067	0.008332066	918
Polr2l	521.8839453	-0.377476484	0.090094645	-4.189777119	2.79E-05	0.001843286	1650
Vps72	840.51093	-0.376181716	0.097055635	-3.875938934	0.000106214	0.004311831	1456
Pcmt1	914.2497322	-0.375561177	0.084596185	-4.439457603	9.02E-06	0.000928577	1132
Ogfod2	343.462347	-0.375427445	0.121385914	-3.09284192	0.001982497	0.025771876	1596
C1qbp	1204.049245	-0.375291526	0.094065335	-3.989689979	6.62E-05	0.00315749	1175
Prrt3	2033.728474	-0.374937942	0.117952944	-3.17870778	0.001479331	0.02154617	3424
Tsc22d1	2687.64127	-0.374935094	0.074742141	-5.016381534	5.27E-07	0.000132281	4999
Slc17a7	10022.48659	-0.374912608	0.120401476	-3.113853914	0.001846609	0.024699167	2915
Nyap1	1556.258382	-0.374697333	0.088880682	-4.215734216	2.49E-05	0.001729199	3912
Coro1b	2296.798898	-0.374661463	0.082066362	-4.56534755	4.99E-06	0.000612811	1888
Emc9	729.9702508	-0.374600033	0.094714435	-3.955046888	7.65E-05	0.003490762	855
Bri3	1491.483554	-0.374426115	0.126572649	-2.958191357	0.0030945	0.033772524	955
Mrpl12	1654.141843	-0.37430593	0.1190989	-3.14281601	0.00167331	0.02318407	1316
Ethe1	256.1511894	-0.374095225	0.085379002	-4.38158347	1.18E-05	0.001106827	1484
Spr	1119.025295	-0.373844264	0.082884665	-4.510415337	6.47E-06	0.00073885	1230
Gfra1	1894.215515	-0.373513691	0.100493514	-3.716794024	0.000201767	0.006484413	3224
Chchd6	540.2851033	-0.373489595	0.102069662	-3.659163644	0.00025304	0.007485218	1121
Nucb1	2544.657579	-0.371436568	0.128491443	-2.890749439	0.003843244	0.038621308	4853
Apoe	63483.02497	-0.371383041	0.102175899	-3.634742102	0.000278259	0.007905353	1104
Tfg	207.2503011	-0.370960203	0.101785821	-3.644517471	0.000267894	0.00772325	945
Cope	2292.416755	-0.370767822	0.102887601	-3.603620057	0.000313816	0.008516609	1300

Grk6	1160.241363	-0.370556638	0.080555647	-4.600008209	4.22E-06	0.00055778	2994
Ciao2b	565.1013126	-0.370291764	0.080712505	-4.587786806	4.48E-06	0.000572382	918
Fdx2	141.4602056	-0.370285956	0.123227223	-3.004903847	0.002656648	0.030831695	812
Arhgap35	5004.31127	-0.370073461	0.084428132	-4.383295609	1.17E-05	0.001100512	6251
Lingo1	3947.509272	-0.369824327	0.114232004	-3.237484371	0.001205885	0.018995612	2782
Nob1	377.9690227	-0.369798097	0.083501942	-4.428616734	9.48E-06	0.000956333	1658
Sncg	24553.9228	-0.36966021	0.099938942	-3.69886054	0.00021657	0.006801057	852
Larp4	988.742804	-0.369575845	0.10707941	-3.451418392	0.000557648	0.011866898	3935
Rabac1	4494.648358	-0.369437294	0.088830104	-4.15891999	3.20E-05	0.002002574	899
Eef1a2	50581.16005	-0.369315003	0.079507818	-4.645014918	3.40E-06	0.000471112	2084
Camsap2	2212.354232	-0.369286063	0.061769793	-5.978424775	2.25E-09	1.77E-06	4916
Fam78b	950.267464	-0.369168917	0.107704545	-3.427607612	0.000608925	0.012545166	4666
Rps19bp1	234.2578233	-0.369117072	0.130727927	-2.823551779	0.004749475	0.043821711	838
Cops7a	897.5607483	-0.369077417	0.081631598	-4.521256779	6.15E-06	0.000711233	1328
Dbn1	2743.244372	-0.369068659	0.073180641	-5.043255351	4.58E-07	0.00012049	2378
Bahd1	3091.723503	-0.369042975	0.071456301	-5.164596656	2.41E-07	7.98E-05	4594
Mrpl34	505.1343318	-0.368899812	0.11794468	-3.127735925	0.001761584	0.023925867	607
Ppp1r12c	2626.730775	-0.368776554	0.084931541	-4.342044766	1.41E-05	0.001233241	2983
Hmox1	559.0710557	-0.368734139	0.08799916	-4.190200657	2.79E-05	0.001842615	1569
Mfsd6	2946.719704	-0.368620842	0.074357313	-4.957425554	7.14E-07	0.000164428	3716
Cmip	9007.322887	-0.368207203	0.075308014	-4.889349502	1.01E-06	0.000209808	2408
Vstm2b	787.436061	-0.368099903	0.132123469	-2.786029661	0.005335799	0.046936454	2627
Fbxo2	4953.093732	-0.368002786	0.090017314	-4.088133382	4.35E-05	0.002466908	1288
Actr3b	474.4441714	-0.367960401	0.129981925	-2.830858219	0.004642329	0.043240743	1712
Pdlim4	353.8840992	-0.367902203	0.104155588	-3.53223682	0.00041206	0.00995417	1182
Tpbgl	6749.280542	-0.367686884	0.106065839	-3.466591027	0.000527103	0.011533023	3022
Armc7	179.4420994	-0.36752079	0.11152995	-3.295265438	0.000983288	0.016840762	2260
Rps25	826.7052056	-0.367393636	0.124426218	-2.952702748	0.003150051	0.034108036	577
Tm2d1	445.8215402	-0.367283174	0.086296417	-4.256065175	2.08E-05	0.001546407	1027
Parva	2007.740839	-0.367158894	0.083532157	-4.395419774	1.11E-05	0.001061293	2222
Thap1	248.6931454	-0.36629277	0.098946108	-3.701942183	0.000213955	0.006772173	2295
Fahd2a	99.82029809	-0.366064277	0.119872163	-3.053788867	0.002259711	0.02797752	1211
Ccdc92	14338.86535	-0.365950718	0.096523601	-3.791308174	0.000149856	0.005363044	2473
Pcbd1	298.9159955	-0.365762654	0.114684109	-3.189305458	0.001426151	0.021053292	789
Pias4	1073.348901	-0.36575788	0.090912091	-4.02320389	5.74E-05	0.002874838	2571
Ilf3	914.0323933	-0.365357316	0.107829585	-3.38828455	0.000703313	0.013724429	3549
Isl2	5388.554114	-0.365212199	0.096589764	-3.781065229	0.000156159	0.005501217	1854
Acly	4743.303908	-0.365176842	0.086166836	-4.238020804	2.25E-05	0.001624464	4393
Camk2g	2910.573107	-0.365126533	0.09592948	-3.806197331	0.00014112	0.005164687	3564
Adam11	2430.304781	-0.364865137	0.11635972	-3.135665301	0.001714648	0.023475144	4620
Kirrel3	449.6392601	-0.364797647	0.115573713	-3.156406732	0.00159726	0.022533695	4108
Limd2	440.124892	-0.364499972	0.130248995	-2.798485863	0.005134282	0.045786708	2950
Vat1	8679.883149	-0.364264479	0.084419197	-4.314948419	1.60E-05	0.001316799	2766
Borcs6	1673.789596	-0.364082016	0.097634031	-3.729048295	0.000192204	0.006287398	1862
Crtc2	920.9624013	-0.36394558	0.101391336	-3.589513598	0.000331296	0.00873226	2747
Ptprf	7777.440457	-0.363858331	0.082191148	-4.426977131	9.56E-06	0.000959229	4690
Furin	4380.786014	-0.363817288	0.100982162	-3.60278767	0.000314823	0.008533403	4286
Zfp428	276.7563132	-0.363662974	0.121947919	-2.982117094	0.002862625	0.032163606	1182
Cyfip2	2935.905511	-0.363477344	0.104821394	-3.467587397	0.000525153	0.011510076	6659
Taf6l	260.7599204	-0.363444845	0.111516876	-3.259101742	0.001117656	0.018158809	2065
Kcns1	2157.52103	-0.36332912	0.107881317	-3.367859509	0.000757542	0.0143808	2712
Nr2f2	4231.666587	-0.363234527	0.089261201	-4.069343924	4.71E-05	0.00259186	4222
Csnk1a1	2165.350541	-0.36316085	0.117823434	-3.082246355	0.002054447	0.026395025	2323
Septin3	516.7750915	-0.362736723	0.131057861	-2.767760134	0.005644298	0.04851448	2397
Rxrb	934.546743	-0.362270577	0.067553531	-5.362718566	8.20E-08	3.79E-05	2627
Mrpl14	448.6118242	-0.362167752	0.098466998	-3.678062283	0.000235013	0.007156665	645
Snapin	898.4668249	-0.362165889	0.080706575	-4.48743969	7.21E-06	0.000798283	1928
1110032A(	218.5824372	-0.361852601	0.093212174	-3.882031547	0.000103587	0.004250444	1015
Tmub2	948.6281231	-0.361765213	0.07576944	-4.774553074	1.80E-06	0.000307825	1876
Pcdhgc3	3709.003624	-0.36176193	0.10113406	-3.577053371	0.000347489	0.008988321	4687
Zfp358	871.2287797	-0.36167823	0.126129597	-2.867512757	0.004137121	0.040367532	1999
Them6	6731.90868	-0.36167073	0.090539476	-3.994619229	6.48E-05	0.003119514	1784
Coprs	348.7370445	-0.361640879	0.09497513	-3.807742899	0.000140241	0.005146656	793
Ift20	326.3909857	-0.361587197	0.125092041	-2.890569161	0.003845449	0.038623714	1141
Abhd17b	611.6487687	-0.361489348	0.10000026	-3.614884076	0.000300482	0.008332066	2249
Dpp7	568.2721505	-0.36140137	0.123923918	-2.916316515	0.00354191	0.036531646	1682
Snrpf	117.0979471	-0.361199503	0.127393926	-2.835296113	0.004578323	0.04287241	861
B4galnt4	836.2414578	-0.361133381	0.090971754	-3.969730863	7.20E-05	0.003344029	3556
Ndufa13	1460.543463	-0.361099842	0.094109844	-3.837003941	0.000124544	0.004790549	1251
Ntng2	1368.214835	-0.361097514	0.114124246	-3.164073617	0.001555774	0.022214882	1593
Mzt2	296.4420905	-0.361088523	0.101264472	-3.565796729	0.000362752	0.009234749	1035

Lrrc27	138.5630703	-0.360946751	0.120629611	-2.992190287	0.002769836	0.031480447	1692
Tmem161a	804.9781599	-0.360925718	0.100937842	-3.575722543	0.000349262	0.009011327	2080
Gadd45gip	790.0833121	-0.360915255	0.123644146	-2.918983768	0.003511745	0.03632797	2494
Kcns3	1456.075024	-0.360548953	0.113687052	-3.171416145	0.001516977	0.021895287	2933
Ubxn1	1677.840851	-0.360488755	0.071540262	-5.038963297	4.68E-07	0.000121765	1033
Eml2	5940.922748	-0.360477029	0.103752529	-3.474392709	0.000512011	0.011300486	2765
Dlgap3	5316.256541	-0.360162818	0.116382851	-3.0946382	0.001970531	0.025692289	3884
Ppp6c	312.1836578	-0.359986178	0.126495327	-2.845845654	0.004429366	0.042047309	1495
Neurl4	1243.012547	-0.359689191	0.102473442	-3.510072312	0.000447985	0.010420981	4886
Calr3	117.2728418	-0.35959699	0.114614098	-3.137458621	0.001704193	0.023378683	1371
Nosip	1854.895032	-0.359417046	0.063673218	-5.644713085	1.65E-08	9.70E-06	1814
Eif3f	3871.145181	-0.359028449	0.088966252	-4.035557761	5.45E-05	0.002804366	2514
Slc41a3	605.8449048	-0.358983928	0.096486031	-3.720579264	0.000198766	0.006406717	2412
Cntnap2	1217.062751	-0.358831294	0.124249024	-2.888000913	0.003876987	0.038801423	6279
Psmb4	2271.252483	-0.358622679	0.093753842	-3.825151814	0.000130692	0.004910985	1183
Evl	3205.711202	-0.358587352	0.088871863	-4.034880554	5.46E-05	0.002804366	2121
Pgbd5	1239.468718	-0.358410377	0.098122935	-3.652666694	0.000259531	0.007581214	2824
Ube2r2	2946.735252	-0.358057086	0.096072179	-3.726959134	0.000193804	0.00632091	3600
Cystm1	2014.152225	-0.357984918	0.109130828	-3.280328057	0.001036864	0.01737924	831
Bod1	1196.621681	-0.357882786	0.079042765	-4.527710839	5.96E-06	0.000700925	1394
Irf2bp2	5307.709785	-0.357526008	0.126697602	-2.821884564	0.004774236	0.043949029	5080
Dgki	1498.366223	-0.357245755	0.116668892	-3.062048069	0.002198281	0.027558439	3028
Gramd1a	1308.547016	-0.357110723	0.097686809	-3.655669855	0.000256511	0.007533413	2713
Pnkp	257.0724703	-0.357079387	0.119516607	-2.98769683	0.002810882	0.031768751	2184
Bicdl1	1430.101987	-0.356866723	0.121471946	-2.93785302	0.003304937	0.035027906	3026
Raph1	557.3479346	-0.35675252	0.125367501	-2.845653925	0.004432033	0.042057739	2241
Ddrgk1	720.7929737	-0.35674833	0.118950503	-2.999132592	0.002707495	0.031128399	1238
Surf1	226.4069395	-0.356676426	0.127947896	-2.787669341	0.00530887	0.046782631	1173
Rcsd1	742.2079967	-0.35644287	0.075046874	-4.749603166	2.04E-06	0.000332014	2622
Rex1bd	908.6754511	-0.356425051	0.103305346	-3.450209173	0.000560152	0.011891404	671
Six1	2018.501959	-0.356411301	0.116759005	-3.052538022	0.00226915	0.02801701	3316
Mpnd	2373.848495	-0.355665218	0.069862586	-5.09092548	3.56E-07	0.000103257	1701
Mypop	2190.465711	-0.355410759	0.11969056	-2.96941346	0.002983688	0.032950982	2173
Cd151	3305.705633	-0.355297418	0.094643582	-3.754057165	0.000173995	0.005884382	1708
Fam8a1	2867.063019	-0.355033088	0.124071365	-2.861523194	0.004216107	0.040810463	3664
Nabp2	999.7975662	-0.355013534	0.103376173	-3.434191098	0.000594325	0.012327478	1130
Nup62	559.6083472	-0.354966512	0.117732705	-3.015020446	0.002569619	0.030182558	2722
Slc2a8	822.420402	-0.354900204	0.077317693	-4.590155132	4.43E-06	0.000572382	2108
Tamalin	712.6181795	-0.354874463	0.083384494	-4.255880743	2.08E-05	0.001546407	2033
Pdlim7	1240.957944	-0.35449209	0.110128699	-3.218889301	0.001286882	0.019794418	899
Dclk1	1555.752971	-0.354024468	0.10952144	-3.23246724	0.001227262	0.0192122	7847
Atxn7l3	1176.24486	-0.353958274	0.099796898	-3.546786321	0.000389961	0.009604831	3408
Tfe3	1021.886622	-0.353841003	0.094975789	-3.725591612	0.000194858	0.006332821	3277
Ttc9b	2332.194792	-0.353764052	0.122184332	-2.895330745	0.003787592	0.038298411	2052
Cfap20	501.7626113	-0.353650765	0.10187074	-3.471563701	0.000517437	0.011380239	1295
Tmem79	1035.639862	-0.353481931	0.120152116	-2.941953429	0.00326149	0.03480821	2406
Wnt2b	635.2821167	-0.353286065	0.106737698	-3.309852772	0.000933451	0.016382896	3318
Clec2l	6024.11147	-0.3531751	0.101588529	-3.476525377	0.000507956	0.011276581	1431
Efcab14	1326.00009	-0.352969912	0.104243145	-3.386025178	0.000709129	0.013807284	2792
Capn1	5720.371088	-0.352502991	0.104143311	-3.384787619	0.000712333	0.013840707	3062
Armcx2	2177.647507	-0.35245117	0.111017904	-3.174723695	0.001499792	0.021747484	3666
Rnf40	1196.582076	-0.352338447	0.091230137	-3.862083951	0.000112424	0.004456914	5279
Dohh	1508.798847	-0.351925722	0.088781492	-3.963953663	7.37E-05	0.003412814	1444
Hgs	4438.710567	-0.351862049	0.089409318	-3.935406939	8.31E-05	0.003662538	2900
Kcnc4	1424.166451	-0.351756303	0.121210868	-2.902019497	0.003707655	0.037724378	2664
Scn1b	12861.526	-0.351477816	0.115187004	-3.051366952	0.00227802	0.028061962	1573
Pgls	972.0629156	-0.351187527	0.118478279	-2.964151148	0.003035192	0.033385589	893
Cep120	572.7592298	-0.351169581	0.114854322	-3.057521678	0.002231755	0.027814975	3261
Dcaf8	2199.878838	-0.351089018	0.061998395	-5.662872733	1.49E-08	8.97E-06	2859
Ssh2	687.3147731	-0.350861629	0.115238796	-3.04464853	0.002329524	0.028473036	4475
Arfip2	1369.830355	-0.350769761	0.104607662	-3.353193769	0.000798848	0.01485674	3275
Arl8a	8887.63606	-0.350756792	0.077619446	-4.518929336	6.22E-06	0.00071533	1713
Fbxl17	2241.526285	-0.350708751	0.083423573	-4.20395266	2.62E-05	0.001787868	4925
Ubb	38731.70041	-0.350537215	0.074581941	-4.700028036	2.60E-06	0.000399875	1499
Rpl38	268.0398658	-0.350389032	0.126856807	-2.76208301	0.005743387	0.049030685	364
Exosc4	669.1694241	-0.350346708	0.118580704	-2.954500147	0.00313176	0.034030861	1796
Psmb6	2021.5754	-0.350077809	0.079173876	-4.421632851	9.80E-06	0.000980294	798
Hebp2	996.1389412	-0.350043303	0.115378092	-3.033880141	0.002414303	0.029090346	2535
Lrrc47	2347.782186	-0.350009044	0.122953555	-2.846676899	0.004417818	0.041976981	3395
Man1c1	2911.368626	-0.349960323	0.088357341	-3.960738491	7.47E-05	0.003443387	4715
Rfng	1359.551573	-0.349798087	0.070853753	-4.936902748	7.94E-07	0.000173614	2118

Kcnh2	7727.792552	-0.349746585	0.106465279	-3.285076499	0.001019547	0.017200452	4221
Rab4b	2731.552873	-0.349629316	0.074924588	-4.66641626	3.06E-06	0.00044181	1192
Zfyve19	683.2200678	-0.349543843	0.10516069	-3.323902135	0.000887673	0.015877364	2008
Gpsm3	207.4582712	-0.349458867	0.120214125	-2.9069701	0.003649481	0.037305365	1343
Otud3	515.4410728	-0.349454101	0.081755619	-4.274374093	1.92E-05	0.001465564	1668
Eif6	1169.693851	-0.349299042	0.09008311	-3.877519772	0.000105527	0.0042998	1500
Prss23	426.1060101	-0.349206465	0.114658078	-3.045633348	0.002321908	0.028419455	3228
Supt5	3678.381332	-0.34915352	0.071776663	-4.864443489	1.15E-06	0.000229376	3513
Cotl1	2021.352966	-0.349121075	0.112187869	-3.111932486	0.00185867	0.024792605	1596
Slc25a39	4635.972157	-0.349058344	0.108272699	-3.223881443	0.001264657	0.019619324	1685
Ube2v1	4719.079608	-0.348970687	0.068564153	-5.089695856	3.59E-07	0.000103257	2041
Vti1b	818.214851	-0.348931792	0.076065837	-4.587233987	4.49E-06	0.000572382	1490
Fbxl3	1104.317837	-0.348796822	0.095445331	-3.654414718	0.000257769	0.007558228	4309
Gnb1	3636.951686	-0.348668446	0.112088331	-3.110657828	0.001866711	0.024851453	2832
Prph	31246.02322	-0.34864557	0.094871997	-3.674904957	0.000237938	0.007194432	1757
Dnajc30	799.0300896	-0.348101206	0.100036994	-3.479724766	0.000501929	0.011201683	1661
Fzd1	1633.469902	-0.347961899	0.086084715	-4.042086919	5.30E-05	0.002769512	4197
Crebbp	4680.359463	-0.347960188	0.125365692	-2.775561507	0.005510647	0.047884757	7749
Card19	1069.367002	-0.347881219	0.093558801	-3.718316342	0.000200555	0.006454906	804
Tubg1	1789.866536	-0.347802856	0.08975981	-3.874817201	0.000106705	0.004327745	1802
Map3k10	6013.954386	-0.347727533	0.103367007	-3.364008921	0.000768191	0.014482633	3682
Ubtd1	1091.330016	-0.346954258	0.082325624	-4.214413937	2.50E-05	0.001736601	1529
Srxn1	6670.641446	-0.34685327	0.125174642	-2.77095476	0.005589219	0.048258056	2812
LTO1	433.6822442	-0.346798619	0.12370689	-2.803389674	0.005056853	0.045356889	2015
Zfand2a	750.2399668	-0.346635184	0.077929659	-4.448052136	8.67E-06	0.000902767	3169
Prdm12	6851.431507	-0.346574484	0.077642216	-4.463737685	8.05E-06	0.000859475	2471
Slc66a2	1595.867237	-0.346486178	0.105058935	-3.298017232	0.000973702	0.016728843	1989
Tradd	242.1907811	-0.346430624	0.090304986	-3.836229182	0.000124938	0.004799322	1689
Eml1	4505.849141	-0.346385068	0.103607052	-3.343257635	0.00082801	0.015218968	2627
Gpr137	670.6820831	-0.346132422	0.106770142	-3.241846611	0.001187579	0.018835469	1434
Rap1gds1	4182.767575	-0.34612611	0.125675207	-2.754132007	0.005884803	0.049812351	3669
Lemd2	1417.446045	-0.346091335	0.12163991	-2.845212026	0.004438187	0.042089062	2627
Pcsk2	8904.495052	-0.346030541	0.123655992	-2.798332163	0.005136726	0.045786708	4723
Pmm1	3055.140793	-0.34602363	0.079391996	-4.358419587	1.31E-05	0.00117784	1300
Unc45a	1934.46122	-0.345407827	0.101265017	-3.410929447	0.000647418	0.013056766	3260
Psmg3	225.7854222	-0.345017019	0.117986541	-2.924206588	0.003453355	0.035990155	883
Hnrnpl	2598.785122	-0.344923415	0.121448765	-2.840073454	0.004510315	0.042516292	1898
Mkrn1	340.9641944	-0.344838566	0.122523892	-2.814459779	0.004885929	0.044442658	1485
Numbl	4366.617504	-0.344803427	0.114941761	-2.999809853	0.002701482	0.031116231	2760
Sh3bgr	233.0046215	-0.344783164	0.115224358	-2.992276719	0.002769051	0.031480447	1341
Arhgdig	2051.499229	-0.344607772	0.085872873	-4.01299923	6.00E-05	0.002951618	1032
Sf3a2	826.8934419	-0.344512936	0.124191578	-2.774044269	0.005536414	0.048011331	2213
Med30	261.4242858	-0.344445591	0.095718542	-3.598525245	0.000320027	0.008595206	1007
Zfp703	2551.677569	-0.344394799	0.076084027	-4.526505927	6.00E-06	0.000703052	3152
Agpat1	1719.592887	-0.344252187	0.121683233	-2.829084807	0.004668133	0.04334413	1911
Zfp64	759.9262003	-0.344042753	0.112713114	-3.052375556	0.002270378	0.028022793	2438
Rtca	1176.928385	-0.343932424	0.076943971	-4.469907373	7.83E-06	0.000849485	1594
Nhp2	449.015216	-0.343902469	0.073795614	-4.660202001	3.16E-06	0.000448016	1042
Vars	3583.36511	-0.343873654	0.077391119	-4.443321871	8.86E-06	0.000916339	4137
Ppp1r37	4871.349487	-0.343832482	0.096253088	-3.572170905	0.000354034	0.009081164	3816
Kif21a	6610.335246	-0.343708869	0.058977116	-5.827834482	5.62E-09	4.04E-06	6184
Kcnj12	1359.374286	-0.343661563	0.077222846	-4.450257652	8.58E-06	0.000895668	2291
Mlycd	774.7981237	-0.343589944	0.103358966	-3.324239364	0.000886601	0.01587109	2128
Hsf4	1412.042413	-0.343525325	0.082718239	-4.152957435	3.28E-05	0.002032334	1739
Septin5	9573.08957	-0.343211035	0.072965277	-4.703758374	2.55E-06	0.000394012	2143
Hs6st3	1046.656186	-0.343181611	0.080074847	-4.285760432	1.82E-05	0.001422326	1708
Mark3	936.1918029	-0.342935587	0.114749103	-2.988568774	0.002802874	0.031702691	3365
Aplp2	7208.852319	-0.342892785	0.123548919	-2.775360457	0.005514056	0.04789124	3462
Klf2	1218.835741	-0.342839838	0.112036856	-3.060062994	0.002212904	0.027655015	1847
Brd2	1860.538381	-0.342720125	0.082909044	-4.133688079	3.57E-05	0.002167814	4657
Mycbp2	717.4011857	-0.342299655	0.086855649	-3.941017757	8.11E-05	0.003614155	5099
Gas6	7189.262872	-0.342120119	0.059228111	-5.776313233	7.64E-09	5.13E-06	2548
Dvl3	1880.291524	-0.34206111	0.104590446	-3.270481424	0.001073646	0.01769222	2519
Def6	766.1899686	-0.342037428	0.089323094	-3.829216077	0.000128552	0.004868039	2277
Pitpnm2	5097.531308	-0.342017034	0.095039034	-3.598700656	0.000319811	0.008595206	6792
Creld1	4356.82253	-0.341940056	0.090255751	-3.788568074	0.000151518	0.005389557	2306
Pex14	2211.924455	-0.34187504	0.07721221	-4.427732874	9.52E-06	0.000958062	2006
Palm	6817.490169	-0.341788683	0.071087578	-4.807994472	1.52E-06	0.000274776	2651
Ngdn	310.183158	-0.341784299	0.097422762	-3.508259166	0.000451049	0.010463376	1200
Ttbk1	10059.7704	-0.341227951	0.111813362	-3.051763628	0.002275012	0.028048483	6959
Timm50	590.5777927	-0.340980639	0.072179517	-4.724063755	2.31E-06	0.000365602	1515

Tnik	1910.703435	-0.340954531	0.101267568	-3.366867987	0.000760271	0.014402381	4404
Dpysl3	2964.377414	-0.340557386	0.082457668	-4.130087527	3.63E-05	0.002189947	2106
Nf2	216.1510097	-0.340519283	0.115563502	-2.946598857	0.003212897	0.03456203	2394
Polr2j	346.6597473	-0.340191496	0.12188713	-2.791037059	0.005253946	0.046458109	646
Adam22	3692.149376	-0.340166282	0.103474379	-3.287444544	0.001011011	0.017109482	9245
Pkmyt1	272.3486692	-0.340098079	0.096006881	-3.54243442	0.000396452	0.009726569	2040
Bnip1	278.8797168	-0.340056253	0.115114146	-2.954078761	0.00313604	0.034068688	1098
2310061I0	1011.361743	-0.340026987	0.114496747	-2.969752368	0.002980399	0.032931199	1293
Mrnip	242.6489779	-0.339958783	0.079111293	-4.297221914	1.73E-05	0.0013923	1142
Pcbp2	263.7633506	-0.339863101	0.109367713	-3.107526817	0.001886599	0.025023239	674
Commd10	276.5534795	-0.339805084	0.119258875	-2.849306468	0.004381465	0.041759503	1610
Dyrk1b	1666.308546	-0.33959641	0.074098884	-4.583016501	4.58E-06	0.00057736	2534
Bola2	358.1011348	-0.339436246	0.104476041	-3.248938637	0.001158365	0.018573122	1266
Nol3	3989.153102	-0.339217632	0.096658286	-3.50945219	0.000449031	0.010423983	3176
Hagh	1147.947166	-0.339109794	0.073768702	-4.596933211	4.29E-06	0.000559353	1191
Timm44	597.5422814	-0.339081522	0.110231014	-3.07609908	0.002097281	0.026765451	1790
Mrps9	344.9846881	-0.339067938	0.094750504	-3.578534398	0.000345526	0.008956998	1418
Fam171a2	3515.638718	-0.339051624	0.089580766	-3.784870774	0.000153788	0.005439509	3118
Ptpmt1	252.131213	-0.33901726	0.081307086	-4.169590575	3.05E-05	0.001941505	1316
Dgkz	17819.17708	-0.339009218	0.085289685	-3.974797396	7.04E-05	0.00329804	3584
Sun2	2735.820614	-0.33899861	0.072059176	-4.704447511	2.55E-06	0.000394012	3804
Mpc2	843.6473007	-0.33857666	0.088554022	-3.823391088	0.000131629	0.004933553	1025
Ndn	5584.472052	-0.338503346	0.102462524	-3.303679546	0.000954248	0.016601048	3694
Rab11fip5	3815.177965	-0.338144961	0.088912117	-3.80313698	0.000142875	0.005187046	6119
Id4	2182.587631	-0.337974898	0.094483055	-3.577095362	0.000347433	0.008988321	3849
Nubp1	487.7417955	-0.337951379	0.073155814	-4.619610652	3.84E-06	0.00051533	2121
Cdc37	3090.747357	-0.337951005	0.068197541	-4.955472036	7.22E-07	0.000164576	7851
Gaa	13490.43757	-0.337944205	0.090515588	-3.73354703	0.000188802	0.006213081	3633
Kctd2	2107.418112	-0.337940532	0.07872987	-4.29240557	1.77E-05	0.001400129	1764
Fam43b	2776.422599	-0.337623693	0.114302054	-2.953784998	0.003139026	0.034084289	2437
Klhl21	2272.645437	-0.337589916	0.106950418	-3.156508603	0.001596702	0.022533695	3986
Hint1	1708.058129	-0.337573356	0.085652662	-3.941189302	8.11E-05	0.003614155	636
Rnf187	13730.6036	-0.337354257	0.082231184	-4.102510032	4.09E-05	0.00236423	1949
Prdm8	322.6563149	-0.337342957	0.095853088	-3.51937494	0.000432565	0.01018662	3316
Elmo2	2433.308761	-0.33732821	0.069835368	-4.830334796	1.36E-06	0.000256094	3502
Dnajc2	343.9638618	-0.337297873	0.097030412	-3.476207784	0.000508558	0.011276581	2133
Tesk1	1936.042246	-0.337203141	0.120869432	-2.789813245	0.005273845	0.046559158	3947
Sync	782.774346	-0.337163113	0.095324441	-3.537005939	0.000404691	0.009860072	2042
Hspa8	22668.52734	-0.337103952	0.094858471	-3.553756981	0.00037977	0.009466978	2019
Plpp1	1746.505007	-0.336958236	0.064952203	-5.187787618	2.13E-07	7.55E-05	1598
C130074G	469.1682292	-0.336390197	0.101517598	-3.313614616	0.000920984	0.016248411	3035
Psmd4	958.0516047	-0.336389909	0.107819652	-3.119931317	0.001808932	0.024360803	1276
Chst12	2645.923117	-0.336255755	0.06787221	-4.9542479	7.26E-07	0.000164576	2331
Ddx49	961.0644989	-0.336230453	0.10408466	-3.230355483	0.001236364	0.019303128	2137
Dhps	696.503808	-0.336094196	0.079980575	-4.202197819	2.64E-05	0.001796218	1333
Arrb2	663.0760478	-0.336012888	0.103152719	-3.257431225	0.001124255	0.018205477	1848
Mast1	16846.32123	-0.335984416	0.094552697	-3.553409123	0.000380273	0.009466978	4872
Scaf1	8676.1717	-0.335935704	0.092062805	-3.648984003	0.000263279	0.007635279	4209
Fbf1	1561.152883	-0.335911136	0.110561464	-3.03822981	0.002379724	0.028877768	4651
Tnrc18	210.1480269	-0.33567987	0.120768813	-2.779524472	0.005443855	0.04755696	696
Rtl8b	5424.723796	-0.335364534	0.092828892	-3.612717189	0.000303005	0.008357353	1232
Spag7	559.551562	-0.335344115	0.090864985	-3.69057581	0.000223747	0.006942157	1159
Egln2	2866.821876	-0.335341376	0.080419972	-4.169876799	3.05E-05	0.001941505	2107
Tppp3	44940.16892	-0.335207837	0.09294189	-3.606638904	0.000310189	0.008471577	1127
Specc1l	1290.593991	-0.335191621	0.112602878	-2.97675892	0.00291313	0.032444264	4725
Zbtb17	645.4432841	-0.335100516	0.092166066	-3.635833978	0.000277083	0.007894973	2722
Srp9	666.9119409	-0.33487293	0.120913973	-2.769513906	0.005614001	0.048378835	1367
Mapre3	3481.650336	-0.334841203	0.08912181	-3.757118546	0.000171881	0.005827613	1816
Sirt6	654.0907839	-0.334712403	0.071861652	-4.6577332	3.20E-06	0.000451963	1891
Cdr2l	10622.63248	-0.334684153	0.095029663	-3.521891404	0.00042848	0.010149842	3700
Naxe	980.9898984	-0.334494038	0.096238638	-3.475673006	0.000509573	0.011276581	908
Map2k2	962.1709078	-0.334445102	0.09482931	-3.526811516	0.000420596	0.01008261	2405
Lifr	6707.527007	-0.334379422	0.083710835	-3.994458072	6.48E-05	0.003119514	4143
Zfp574	1079.293666	-0.334322377	0.098862109	-3.381703868	0.000720378	0.013928611	3145
Rtn4rl1	3559.399762	-0.334314968	0.082377912	-4.058308346	4.94E-05	0.002662199	3525
Aarsd1	329.2115632	-0.334297718	0.098922224	-3.379399523	0.000726444	0.013995644	1516
Trim8	4717.383847	-0.334058533	0.094783965	-3.524420331	0.00042441	0.010115454	3580
Bcam	21922.97718	-0.334056832	0.097732892	-3.418059418	0.000630693	0.012838078	2429
Mrps15	465.3570959	-0.333967085	0.088513222	-3.77307569	0.000161247	0.005590883	937
Trir	1841.635737	-0.333934385	0.101825197	-3.279486746	0.001039961	0.017397977	902
Plxnc1	8085.926361	-0.333929733	0.109041422	-3.062411766	0.002195612	0.027532824	7302

Mid1ip1	4984.517185	-0.333677253	0.08104263	-4.117305345	3.83E-05	0.002268239	2019
Stat5b	1647.159531	-0.333338214	0.111606129	-2.986737535	0.002819717	0.031833297	5062
Brms1	668.7477157	-0.332980246	0.084392479	-3.945615167	7.96E-05	0.003581791	1388
Fzd7	1361.979704	-0.332890594	0.099766275	-3.336704655	0.00084778	0.015478672	4532
Rab3ip	666.5572811	-0.3328443	0.078031342	-4.265520621	1.99E-05	0.001498843	1844
Poll	689.2463202	-0.332843763	0.088422213	-3.764255061	0.000167046	0.00573317	2324
Gatad2a	464.3976492	-0.332753502	0.10381282	-3.205321876	0.001349115	0.020378942	3812
1110032F0	1756.714567	-0.332429038	0.1131335	-2.938378452	0.00329934	0.034998323	2578
Ano8	3410.895382	-0.332114193	0.09566036	-3.47180581	0.00051697	0.011379014	3653
Galnt11	711.6076355	-0.33208391	0.119783215	-2.772374321	0.0055649	0.048152105	2552
Cyb5r1	1642.107489	-0.332079098	0.10120719	-3.2811809	0.001033734	0.017353236	1837
Wrap73	443.3755044	-0.332007015	0.094389782	-3.517404185	0.00043579	0.010234771	1598
Coro7	3169.705108	-0.331778831	0.077800521	-4.264480842	2.00E-05	0.001503269	4296
Csk	3509.390704	-0.331724639	0.093969798	-3.530119752	0.000415372	0.010008683	2749
Peds1	6341.631351	-0.331666687	0.101828971	-3.257095533	0.001125585	0.018206904	2329
Pick1	746.2145522	-0.331636275	0.11721648	-2.829263213	0.004665531	0.04334413	1977
Dhx30	974.7729596	-0.331521931	0.091561849	-3.62074308	0.000293758	0.008189648	3968
Gatd3a	1558.00764	-0.331338348	0.072175989	-4.590700512	4.42E-06	0.000572382	1378
Noa1	894.5484293	-0.331087124	0.080315702	-4.122321224	3.75E-05	0.002236253	2288
Cpe	18533.94412	-0.331011984	0.095719775	-3.458135817	0.000543927	0.011733185	2117
Dnm1	13037.03758	-0.330798578	0.095528635	-3.462821146	0.000534544	0.011643088	3764
Ostf1	1471.804193	-0.330781389	0.094218513	-3.510789745	0.000446778	0.010409421	974
Phf24	339.1382979	-0.330740702	0.101151261	-3.269763511	0.001076374	0.017723894	492
C030006K1	464.8068321	-0.330446132	0.112003829	-2.950311031	0.003174542	0.034283647	1754
Mtmr4	488.8095106	-0.330232367	0.100091298	-3.299311451	0.000969223	0.016710547	792
Ube2d3	1085.24979	-0.330205079	0.105056649	-3.143114536	0.001671604	0.02317631	1512
Pgrmc1	3322.096642	-0.330046388	0.10443798	-3.160214188	0.001576532	0.022409385	1857
Fkbp4	12797.10512	-0.32999171	0.086943972	-3.795452418	0.000147374	0.005317833	2217
Pde4dip	5392.000457	-0.329949223	0.086021678	-3.83565201	0.000125232	0.00480437	8264
Tomm34	3055.085994	-0.329893288	0.069780325	-4.727597449	2.27E-06	0.00036409	1923
Rtn4rl2	2013.862693	-0.329790823	0.11908454	-2.769384032	0.005616239	0.048378835	1303
Tuba4a	2111.256803	-0.329628494	0.101262757	-3.255179916	0.001133206	0.018289787	1205
Twf2	534.7661381	-0.329581773	0.093596238	-3.521314317	0.000429413	0.010166481	1596
Aimp2	412.4117253	-0.329475088	0.09686316	-3.401448899	0.000670297	0.013358835	1073
Clta	1798.814699	-0.329375241	0.085446143	-3.854770122	0.000115838	0.004543321	1135
Adprh	2876.589699	-0.32936276	0.070609361	-4.66457642	3.09E-06	0.000442877	2966
Agtpbp1	1509.52067	-0.32911508	0.086478252	-3.805755478	0.000141372	0.005164687	3037
Tatdn2	738.6913323	-0.328955209	0.102828042	-3.199080749	0.001378665	0.020637987	3096
Fbxo31	9551.259171	-0.328886009	0.093004401	-3.53624134	0.000405864	0.009869358	4358
Srgap3	8177.334163	-0.328535981	0.055437499	-5.926241003	3.10E-09	2.39E-06	8887
Rusc2	4348.708584	-0.328290154	0.085732891	-3.82922061	0.00012855	0.004868039	5325
Fam104a	1454.656192	-0.328283486	0.107717689	-3.047628373	0.00230655	0.028286601	2619
Maf	9194.980339	-0.328263677	0.08816017	-3.723491864	0.000196486	0.006356524	6360
Sfrp5	8807.125992	-0.328053555	0.099445954	-3.298812474	0.000970948	0.01672145	1900
Srf	1329.612117	-0.327998996	0.1054561	-3.110289447	0.001869041	0.024865863	2616
Kat2a	1797.748898	-0.327971188	0.10741161	-3.053405383	0.002262601	0.027989655	3043
Gopc	520.9018017	-0.327937111	0.103197182	-3.177771958	0.001484114	0.021591358	4216
Acot7	9281.645011	-0.327921488	0.077090688	-4.253710751	2.10E-05	0.001558844	1457
Syn1	13128.53784	-0.327914674	0.101331381	-3.236062434	0.001211909	0.01907092	3209
Ndufb2	394.4527364	-0.327909048	0.100979876	-3.247271268	0.001165173	0.018621132	455
Ebf1	264.313508	-0.327835112	0.111491852	-2.940440093	0.003277464	0.034872626	2864
Lamtor1	2562.076541	-0.327747646	0.06624764	-4.947310512	7.52E-07	0.000168785	1119
Casz1	3985.837398	-0.327656434	0.092225192	-3.552786674	0.000381173	0.009467961	7926
Ubqln1	4430.172387	-0.327270035	0.101592795	-3.221390213	0.001275703	0.019748695	3561
Sbf1	4919.42582	-0.327247551	0.077870519	-4.202457567	2.64E-05	0.001796218	6190
Nrarp	1337.330842	-0.326969666	0.105017683	-3.113472483	0.001848998	0.024716076	2582
Lrp10	6418.307848	-0.326712867	0.090529848	-3.608896667	0.000307502	0.008418012	4228
Fam189a2	756.0377822	-0.326693598	0.069630971	-4.691785731	2.71E-06	0.000408944	2548
Nrg1	2931.603447	-0.326637418	0.083121437	-3.929641143	8.51E-05	0.003725321	3049
Nelfcd	1331.002251	-0.326637357	0.105986068	-3.081889576	0.002056911	0.026411245	2279
Ccdc124	2841.237312	-0.326402084	0.107981302	-3.022764854	0.002504768	0.029728367	1356
Ier3	325.3784055	-0.32624296	0.104986505	-3.107475196	0.001886928	0.025023239	1131
Prkcd	4003.68882	-0.32617259	0.109517558	-2.978267564	0.002898828	0.03238805	2777
Mvb12a	694.8694837	-0.326115174	0.103018881	-3.16558646	0.001547707	0.022157252	1062
Alyref	571.2882317	-0.326048253	0.099402816	-3.280070571	0.001037811	0.017387641	3527
Nt5c	547.6151754	-0.32595876	0.088434137	-3.68589293	0.000227902	0.007018954	858
Sympk	6083.580808	-0.325942336	0.078431753	-4.155744642	3.24E-05	0.002023356	4116
Rtn4r	672.6466132	-0.325893517	0.093758143	-3.475895582	0.00050915	0.011276581	1938
Tomm40	2045.411385	-0.325753144	0.079510332	-4.096991392	4.19E-05	0.002407993	1577
Aard	176.1168701	-0.325714517	0.10338174	-3.150600071	0.001629354	0.022859774	1348
Zfp821	197.1867413	-0.325634677	0.113925292	-2.858317684	0.004258938	0.041089271	2033

Psmd3	4941.031278	-0.325512266	0.060998234	-5.336421147	9.48E-08	4.17E-05	2148
Ephx4	150.7582861	-0.325464337	0.111583523	-2.916777736	0.003536677	0.036500001	1279
Tmub1	772.8004674	-0.325462702	0.115057798	-2.828688775	0.004673913	0.043378537	1333
Dbnl	1936.56435	-0.325294845	0.091539823	-3.553588295	0.000380014	0.009466978	2001
Clint1	1265.478906	-0.325280929	0.086008262	-3.781973063	0.00015559	0.005489987	3398
Akap12	24541.31395	-0.325228752	0.074052859	-4.391846002	1.12E-05	0.001074202	6195
Sumo3	1429.091443	-0.325091797	0.074867307	-4.34223975	1.41E-05	0.001233241	2630
Kcnh1	1663.226893	-0.324937807	0.093927272	-3.459461777	0.000541256	0.011711961	7161
5730409EC	3063.181526	-0.324871013	0.063497714	-5.116263162	3.12E-07	9.72E-05	2855
Ubxn6	2040.354069	-0.324838456	0.079963502	-4.062334043	4.86E-05	0.002640318	2949
Bag3	11823.35737	-0.324827838	0.10059162	-3.229173926	0.001241484	0.019368995	2562
Nefl	166541.2513	-0.324744013	0.094467103	-3.437641284	0.000586805	0.012240767	3380
Scaf4	1072.987177	-0.324677287	0.101482713	-3.199335885	0.001377446	0.020633529	4266
Ino80e	936.2751255	-0.324599652	0.104960004	-3.092603288	0.001984092	0.025784982	1973
Rab18	471.4743433	-0.324488975	0.083323388	-3.89433245	9.85E-05	0.004098445	1184
Map3k5	1607.924594	-0.324423879	0.095502482	-3.397020403	0.000681239	0.013485217	5450
Fignl2	778.6311703	-0.32427181	0.088512512	-3.66357031	0.000248724	0.00740362	4462
Bcas3	843.4255776	-0.324050588	0.106323207	-3.047787948	0.002305325	0.028286601	2789
Fbxo21	3556.029031	-0.323884502	0.074583067	-4.342601026	1.41E-05	0.001233241	3883
Rtn1	18494.25929	-0.323757737	0.077092535	-4.199599074	2.67E-05	0.001808571	3629
Sphk2	930.1443466	-0.323459246	0.091777989	-3.524366249	0.000424497	0.010115454	2968
Ndufb7	1506.873488	-0.323305607	0.108819142	-2.971036156	0.002967968	0.032851644	623
Gins4	373.6232775	-0.323279611	0.117163301	-2.759222447	0.005793908	0.049293406	1349
Ncaph2	413.0845338	-0.323114028	0.115361287	-2.800887852	0.005096223	0.045588764	3302
Aprt	559.8302337	-0.323111102	0.117098131	-2.759319043	0.005792196	0.049293406	849
Ssbp2	517.4090326	-0.322979073	0.089173349	-3.621923773	0.00029242	0.008162704	1633
Urm1	1490.341775	-0.322951666	0.069146952	-4.670511976	3.00E-06	0.000436864	2196
Cmas	2343.49271	-0.322759841	0.07832355	-4.120853074	3.77E-05	0.002239617	1760
Nbr1	3035.857008	-0.3226434	0.097973675	-3.2931642	0.000990666	0.016926868	4339
Rabgef1	166.9015985	-0.322609929	0.089866696	-3.589871912	0.00033084	0.008725496	828
Rhov	504.812499	-0.322505282	0.082367553	-3.915440843	9.02E-05	0.00387438	1703
Lrrc14	236.0606788	-0.322476664	0.100734912	-3.201240335	0.001368373	0.020565833	4849
Rac3	443.1423718	-0.322382678	0.089929127	-3.584852765	0.000337268	0.008836908	1076
Lrch4	1138.284453	-0.322353692	0.102644886	-3.140474926	0.001686741	0.023276079	3078
Cherp	2244.77509	-0.322253057	0.112048745	-2.876007717	0.004027399	0.039762016	3646
Spata13	2156.863426	-0.322223346	0.093436718	-3.44857302	0.000563557	0.011946377	5469
Auh	1128.141974	-0.322093333	0.062178867	-5.180109405	2.22E-07	7.62E-05	1320
Rbfa	503.8009594	-0.321984887	0.072018322	-4.470874606	7.79E-06	0.000847745	1377
Rnf220	1822.817858	-0.321953563	0.090117639	-3.572592074	0.000353465	0.009077158	1949
Suds3	1782.285591	-0.321737955	0.079720594	-4.035819829	5.44E-05	0.002804366	2425
Rtn2	3483.415774	-0.321641832	0.089399755	-3.597793213	0.000320929	0.008595206	1990
Cdk2ap1	1522.82592	-0.321223434	0.093290903	-3.443244992	0.000574778	0.012078934	1315
Serp2	320.8230334	-0.321009081	0.082991908	-3.867956406	0.000109751	0.004397252	748
P3h2	232.272662	-0.32098832	0.081756179	-3.926165859	8.63E-05	0.00374813	3283
Ube2e2	100.2662276	-0.320966019	0.116158502	-2.763172856	0.005724244	0.048971862	666
Btbd2	4946.531805	-0.320949511	0.076309971	-4.205865952	2.60E-05	0.00178387	2449
Tshz2	3232.464889	-0.320695596	0.075600312	-4.241987735	2.22E-05	0.001612643	4314
Rtn4	26759.00138	-0.320520114	0.08402189	-3.814721546	0.000136337	0.005049737	4618
Apeh	1246.109441	-0.320395527	0.105350929	-3.041221658	0.002356203	0.028655938	2518
Dph1	362.8835156	-0.32035632	0.108850167	-2.943094437	0.003249493	0.034771074	2139
Rbfox3	2295.288459	-0.320263729	0.083376482	-3.841175865	0.000122446	0.004730539	2809
Crebzf	2024.175512	-0.320196105	0.112483059	-2.846616274	0.004418659	0.041976981	3376
Nfkbib	922.4878604	-0.320162478	0.083400986	-3.838833257	0.00012362	0.004767516	1243
Fbxw8	3716.601874	-0.320114355	0.094812874	-3.37627518	0.000734744	0.014074952	5000
Cul9	4565.39668	-0.320089584	0.072730431	-4.40104063	1.08E-05	0.00104319	7848
Ubald1	1849.814517	-0.320079751	0.083676083	-3.825223862	0.000130653	0.004910985	1851
Map3k14	381.0095325	-0.319993427	0.087083472	-3.674559832	0.00023826	0.007194432	4246
Aldh2	2956.623607	-0.319819497	0.099579969	-3.211685025	0.00131959	0.020130381	1799
Psmc3	3596.38077	-0.319767703	0.065341682	-4.893778286	9.89E-07	0.000208083	1636
Wipi1	391.753311	-0.319759484	0.113756758	-2.810905376	0.004940231	0.044727609	2050
Spice1	193.7151276	-0.319596898	0.107757377	-2.965893457	0.00301805	0.03326362	4627
Stk11	1790.16309	-0.319456138	0.096104121	-3.324062838	0.000887162	0.015874676	2566
Copa	5674.975762	-0.319364309	0.112922412	-2.828174697	0.004681425	0.043430863	4351
Rnf103	3317.792575	-0.318619417	0.09978608	-3.193024682	0.001407909	0.020919412	3264
Rp9	444.9572686	-0.318580854	0.111243188	-2.863823469	0.004185612	0.040640555	1134
Golga3	1671.147203	-0.318566973	0.106337596	-2.995807567	0.002737191	0.031295403	4805
Lin37	348.9505835	-0.318476337	0.096427015	-3.30277087	0.000957346	0.016631694	998
Dusp1	648.5800957	-0.318430311	0.107619187	-2.958861882	0.003087775	0.033752862	1990
Anapc11	2162.773737	-0.318370788	0.090935629	-3.501056658	0.000463417	0.010644797	962
Riox1	325.8885693	-0.318286095	0.110976328	-2.868053955	0.004130051	0.040359567	2344
Mea1	744.3221428	-0.31824808	0.106139279	-2.998400634	0.002714006	0.031154718	945

Tsen34	1491.03602	-0.318128817	0.114483402	-2.778820435	0.005455667	0.047609849	1319
Chkb	571.9428094	-0.318100757	0.086499653	-3.677480164	0.000235549	0.007157955	1707
Clu	15653.7334	-0.318081286	0.088443861	-3.596420179	0.000322627	0.008607375	1810
Atp5d	8195.695751	-0.317986681	0.078817994	-4.034442697	5.47E-05	0.002804366	931
Ppp1r15a	927.3445657	-0.317956263	0.090202916	-3.524900046	0.000423643	0.010111535	2333
Babam1	1946.098043	-0.317926901	0.100662416	-3.158347622	0.001586662	0.022473457	1433
Gtf2f1	993.7855129	-0.317903264	0.074327697	-4.277049844	1.89E-05	0.00146246	1717
Cir1	505.7190231	-0.317851691	0.078132714	-4.06809996	4.74E-05	0.00259186	2821
Pomgnt2	1803.475613	-0.317843192	0.103664359	-3.066079742	0.002168855	0.027321975	2365
Lgals1	1572.390286	-0.317818913	0.11141095	-2.852672136	0.004335333	0.041501181	800
Pdcd5	335.260392	-0.317715152	0.111303399	-2.854496409	0.004310512	0.041332967	684
Ccdc61	280.4617196	-0.317630195	0.094258214	-3.369787961	0.00075226	0.014311177	2022
Uqcr10	853.1435027	-0.317519148	0.072737438	-4.365278118	1.27E-05	0.001150896	434
Prokr1	1039.535398	-0.31746591	0.113609647	-2.794356974	0.005200304	0.046134193	4075
Rpl24	1445.504634	-0.317417187	0.105212957	-3.016902067	0.002553723	0.030082961	677
Atp5j	628.8150229	-0.317389229	0.094893675	-3.344682657	0.000823768	0.015172579	809
Pfkl	2709.032839	-0.316997484	0.078095862	-4.05908171	4.93E-05	0.002662199	3730
Sox12	1484.675858	-0.316770874	0.097416101	-3.251730164	0.001147049	0.018459002	4453
Dgcr2	2176.259424	-0.316668876	0.110200362	-2.873573826	0.004058562	0.039945082	4045
Dus3l	759.082254	-0.316580803	0.083222971	-3.804007471	0.000142374	0.005183663	2025
Mrpl2	414.0420127	-0.316570094	0.113159579	-2.797554529	0.005149107	0.045845334	1031
Rad23a	876.9643169	-0.316435878	0.102797159	-3.078255086	0.002082166	0.026635988	2074
Plekhh3	743.669631	-0.316431586	0.114391216	-2.76622277	0.005670978	0.048658187	3010
Fam149a	705.707635	-0.316367687	0.077452575	-4.084663276	4.41E-05	0.002478479	2661
Slc49a4	1992.207417	-0.316363245	0.077823394	-4.065143264	4.80E-05	0.00261518	5395
Atxn2l	551.1039817	-0.316242037	0.109496856	-2.888138054	0.003875297	0.038801423	3853
Arhgef10l	2602.363927	-0.31603237	0.097170701	-3.252342174	0.001144581	0.018426042	4382
Tubg2	1198.221776	-0.315759678	0.082748986	-3.81587367	0.000135702	0.005030468	1754
Gale	436.3949671	-0.315738362	0.104056984	-3.034283226	0.00241108	0.029090346	1430
Ak2	221.5460952	-0.315699224	0.107591712	-2.934233662	0.003343724	0.035279397	986
Trp53rka	296.8578707	-0.31566845	0.085972147	-3.671752558	0.000240893	0.007249484	1880
Myh9	20341.96972	-0.315650597	0.099724535	-3.165225052	0.00154963	0.022161818	7433
Smdt1	1043.097575	-0.315619559	0.067247774	-4.693382991	2.69E-06	0.000408804	705
Fbxl16	7336.111498	-0.315560472	0.090346299	-3.492788053	0.000478006	0.010827162	3489
P4htm	1655.258792	-0.31537316	0.084636048	-3.72622739	0.000194367	0.006332821	1841
Marchf5	1445.126587	-0.31532231	0.071072332	-4.436639409	9.14E-06	0.000934248	1809
Mrpl20	328.1462453	-0.315236872	0.095357194	-3.305853071	0.000946877	0.016539316	764
Ccdc32	527.1306446	-0.315203386	0.065095602	-4.842161039	1.28E-06	0.000248069	1870
Lrfn1	1939.556012	-0.31493175	0.082235978	-3.829610322	0.000128346	0.004868039	3060
Gramd4	1089.876112	-0.314849265	0.073143921	-4.304517168	1.67E-05	0.001357483	4292
Recql5	490.3757848	-0.314727808	0.054025042	-5.825591218	5.69E-09	4.04E-06	3971
Spindoc	621.5478145	-0.314575439	0.061791218	-5.090940877	3.56E-07	0.000103257	3152
Eif3g	1499.181382	-0.314539784	0.096477674	-3.260233896	0.001113204	0.018119956	1101
U2af2	2435.807954	-0.314531351	0.106970446	-2.940357488	0.003278338	0.034873486	2183
Myadm	640.788704	-0.31430759	0.096308748	-3.263541446	0.001100291	0.017972429	1898
Zmynd19	584.3150306	-0.314171888	0.098121016	-3.201881722	0.00136533	0.02055007	3903
Med29	364.926065	-0.314168036	0.107585283	-2.920176708	0.00349833	0.03624868	1281
Med9	1924.651341	-0.313935174	0.07766611	-4.042112766	5.30E-05	0.002769512	1920
Cisd1	1264.405212	-0.313788999	0.069843443	-4.492748155	7.03E-06	0.000784562	1084
Zfta	1892.212035	-0.313739444	0.111744662	-2.807645912	0.004990507	0.044960578	4989
Midn	3229.716738	-0.313727243	0.108770441	-2.884306081	0.003922773	0.039045665	3597
Khnyn	441.2387666	-0.313668698	0.1005605	-3.119203845	0.001813405	0.024401153	3898
Gkap1	339.3314114	-0.313662368	0.086535584	-3.624663428	0.000289338	0.008106748	1523
Kcnip1	393.5867267	-0.31363739	0.100703259	-3.114471091	0.00184275	0.024679431	1591
Mtarc2	868.4000045	-0.313553915	0.095330482	-3.289125462	0.001004992	0.01705306	1917
Coasy	583.3054911	-0.313494608	0.106971378	-2.930640081	0.003382644	0.035510497	2138
Aamp	2174.141873	-0.313380803	0.102729025	-3.050557537	0.002284169	0.028111131	1817
Ube2o	14049.08581	-0.313314805	0.071641604	-4.373363877	1.22E-05	0.001132119	5205
Luc7l2	866.9754398	-0.31305857	0.099416215	-3.148968898	0.001638476	0.022883158	4253
Ndufs7	1793.226432	-0.313034616	0.065919037	-4.748774101	2.05E-06	0.000332014	758
Epn1	2882.587872	-0.312809053	0.073571515	-4.251768561	2.12E-05	0.00156978	2127
Gabarapl2	3043.119782	-0.312759445	0.080483183	-3.886022331	0.0001019	0.004194794	1677
Avpi1	280.6158483	-0.312753567	0.102108223	-3.062961609	0.002191582	0.027513675	1044
Spire2	4685.081031	-0.312352454	0.08406627	-3.71555027	0.000202762	0.006511635	2330
Psmb5	1985.33423	-0.312285313	0.08181962	-3.816753393	0.000135219	0.005021041	899
Pfdn1	628.0939475	-0.312002278	0.089767231	-3.475681205	0.000509558	0.011276581	1109
Rbbp7	1418.145923	-0.311997899	0.073032911	-4.272017854	1.94E-05	0.001470907	2244
Acad8	457.0219964	-0.311850836	0.09456503	-3.297739518	0.000974665	0.016732194	2859
D8Ertd738	862.3385103	-0.311703806	0.111267323	-2.80139575	0.005088208	0.045551427	616
Cacna2d2	550.169938	-0.311646061	0.102076818	-3.053054227	0.00226525	0.027991388	5332
Ttc39a	598.9140038	-0.311465135	0.111954708	-2.78206376	0.005401443	0.047343387	2356

Syk	137.7696156	-0.311243438	0.107454865	-2.896503931	0.003773459	0.038190638	5363
Kcnip3	1372.428879	-0.311204574	0.080785385	-3.852238549	0.000117043	0.004570342	1296
Rusc1	4773.133431	-0.311143598	0.098466686	-3.159886983	0.001578303	0.022420069	3316
Ngfr	24158.27903	-0.310671955	0.090556829	-3.430684996	0.000602059	0.012438692	3446
Pdpn	644.7173875	-0.310491496	0.101003666	-3.07406165	0.002111658	0.026860217	1817
Pik3r2	3609.658798	-0.310292749	0.085638532	-3.623284313	0.000290886	0.008135365	3165
Tbc1d9	2843.771427	-0.310219001	0.074459178	-4.166296338	3.10E-05	0.001964076	4631
Ywhah	34032.73198	-0.310097023	0.084336787	-3.676889231	0.000236095	0.007165366	1764
Qrich1	3146.981586	-0.310055411	0.094396775	-3.284597498	0.001021282	0.017209906	3040
Map3k21	190.3689948	-0.310021561	0.102608108	-3.021413877	0.002515972	0.029817416	5693
Abtb1	1027.269551	-0.309929351	0.069633265	-4.450880618	8.55E-06	0.000895668	1845
Arhgef25	3220.351493	-0.309925756	0.062700747	-4.942935631	7.70E-07	0.000171742	2475
Upf1	4062.588767	-0.309913216	0.087622129	-3.536928615	0.000404809	0.009860072	4518
Macf1	810.2212802	-0.309832834	0.084111614	-3.683591615	0.000229971	0.007067098	2808
Csnk2b	1322.38157	-0.309814674	0.092270115	-3.357692496	0.00078596	0.014693104	961
Bsn	30069.35045	-0.309647978	0.095578177	-3.239735118	0.001196408	0.018914085	15938
Sf3a1	3265.666764	-0.309624543	0.090586615	-3.417994418	0.000630844	0.012838078	4920
Mier2	889.0074209	-0.309540197	0.105930827	-2.922097426	0.003476828	0.03614536	2676
Efnb2	2675.255595	-0.309496879	0.104977383	-2.948224363	0.00319605	0.034448233	4793
Apbb1	8822.848406	-0.309305617	0.070241564	-4.403455756	1.07E-05	0.001034003	2690
Rogdi	1228.752445	-0.309257993	0.08982654	-3.442835404	0.00057565	0.012086166	1355
Mrpl13	244.7277511	-0.309206206	0.081614919	-3.788599056	0.000151499	0.005389557	1223
Zfp131	234.0189917	-0.30919001	0.087012215	-3.553409266	0.000380272	0.009466978	1783
Cpeb3	2044.400892	-0.308960619	0.109754645	-2.81501178	0.004877545	0.044422605	5899
Tmem120a	801.1825981	-0.308926591	0.074856157	-4.126936273	3.68E-05	0.002208029	1559
6430571L1	636.6760021	-0.308918181	0.095939729	-3.219919267	0.001282267	0.019780656	2599
Tubb3	59636.91194	-0.308888015	0.098307806	-3.142049727	0.001677695	0.023202227	1868
Slc35c2	761.031123	-0.308801911	0.063048254	-4.897866199	9.69E-07	0.000204782	2008
Slc45a1	3126.774609	-0.308590477	0.096582052	-3.195112038	0.001397766	0.020824388	3387
Nudt9	1274.767591	-0.30827463	0.090745121	-3.39714827	0.000680921	0.013484989	1488
Brf2	355.7075591	-0.308239121	0.086181544	-3.57662567	0.000348058	0.008996103	1899
Slc29a4	995.482043	-0.30817871	0.109239226	-2.821135985	0.004785391	0.043986981	2789
Jun	8109.660305	-0.307908297	0.097768041	-3.149375759	0.001636197	0.022883158	3189
Fsd1	1925.705944	-0.307862648	0.083526505	-3.685807867	0.000227978	0.007018954	1748
Gmpr	1197.835558	-0.307808078	0.060345369	-5.100773842	3.38E-07	0.000101863	1580
2210016L2	2699.842327	-0.30779182	0.087208229	-3.529389646	0.000416519	0.010017656	2607
Nub1	2387.353924	-0.307774086	0.074935914	-4.107163954	4.01E-05	0.00233555	3269
Cox5b	1962.567757	-0.307753945	0.066610237	-4.620219903	3.83E-06	0.00051533	865
Ap2m1	5353.85981	-0.30765059	0.087069039	-3.533409759	0.000410236	0.009934919	2937
Pusl1	153.2437465	-0.307524964	0.102715268	-2.993955706	0.002753859	0.031365611	1243
Gnaz	4248.661715	-0.307460316	0.075846803	-4.053701723	5.04E-05	0.002702962	3519
Ret	20311.14231	-0.307362789	0.1100407	-2.793173707	0.005219366	0.046236028	4497
Nsd3	508.7787387	-0.307276482	0.101096653	-3.039432791	0.002370241	0.028794595	3652
Pebp1	6924.468999	-0.307264147	0.084197384	-3.649331289	0.000262924	0.007634302	1240
Shc2	4405.29054	-0.307231936	0.08928226	-3.441130832	0.000579288	0.012122043	3243
Scrt2	6666.373433	-0.307068324	0.108878112	-2.820294355	0.004797962	0.044010511	3395
Smarca4	7298.4131	-0.306895609	0.106512031	-2.881323402	0.003960091	0.039265988	5405
Erich3	663.2582869	-0.306832307	0.089127725	-3.442613485	0.000576122	0.012090314	8280
Stxbp1	24875.04005	-0.306724452	0.080644392	-3.803419463	0.000142712	0.005185414	3892
Dffa	2032.804268	-0.306576708	0.100142782	-3.061395949	0.002203075	0.027597355	1835
Nacad	17879.99027	-0.306563346	0.087688367	-3.496054925	0.000472191	0.0107434	4892
Efna1	588.3668663	-0.306521121	0.07027694	-4.361617374	1.29E-05	0.001163131	1480
Ccdc92b	2262.660814	-0.306280627	0.096019159	-3.189786604	0.001423779	0.021040819	4002
Reep2	6557.489963	-0.30623478	0.088183802	-3.472687426	0.000515275	0.011349734	1926
Eif3k	1863.613909	-0.306087999	0.074042783	-4.133934264	3.57E-05	0.002167814	774
Zfp777	592.7968527	-0.30576717	0.096035148	-3.183908989	0.001453007	0.021313534	3184
Snx18	2626.384778	-0.30573835	0.070418793	-4.341715308	1.41E-05	0.001233241	4447
Mif	2379.542234	-0.305729266	0.094118885	-3.248330742	0.001160843	0.018573157	544
Dusp26	2189.150889	-0.305684075	0.107054943	-2.855394293	0.004298343	0.041261276	1938
Tnfrsf11a	902.8990862	-0.305497622	0.076735234	-3.981190976	6.86E-05	0.003231207	5029
Dok4	6188.121598	-0.305433073	0.086155462	-3.545138837	0.000392406	0.00964885	2590
Prr12	5255.140219	-0.305351642	0.108143842	-2.823569393	0.004749214	0.043821711	7023
Rpl28	1686.774392	-0.305339924	0.097101744	-3.144535951	0.001663505	0.023107736	1106
Ino80b	593.4371372	-0.305336265	0.095435987	-3.199382921	0.001377221	0.020633529	1503
Mtcl1	9963.450394	-0.305170451	0.082617078	-3.693793823	0.000220933	0.006886468	7221
Sptan1	56456.322	-0.304875628	0.072098288	-4.228611207	2.35E-05	0.001659367	7892
Dynll1	3499.872758	-0.304706851	0.103196133	-2.952696404	0.003150116	0.034108036	2030
Fam89a	713.4788083	-0.304664378	0.101310512	-3.007233622	0.002636371	0.030695984	1338
Cln6	827.9967659	-0.304594267	0.088880957	-3.426991313	0.000610309	0.0125619	2142
1700037H(	2586.993755	-0.304589075	0.102363146	-2.975573595	0.002924412	0.032516155	1241
Gne	1224.098827	-0.304550103	0.085405277	-3.565940129	0.000362554	0.009234749	2920

Prxl2b	500.9382337	-0.304549502	0.105530833	-2.885881719	0.003903188	0.038945728	883
Cd44	1743.702025	-0.304467242	0.106127285	-2.868887492	0.004119183	0.04031609	2848
Ndufb11	1018.61757	-0.304238757	0.107996915	-2.817105988	0.004845853	0.044246715	908
Snrpn	26764.16347	-0.304158904	0.084034459	-3.619454551	0.000295225	0.008220109	1932
Pja1	1301.537983	-0.30381688	0.093914455	-3.235038533	0.001216263	0.019104326	2108
Plbd2	8042.587692	-0.303702693	0.08245331	-3.683329303	0.000230207	0.007067787	4208
Uchl1	39932.5438	-0.303541488	0.100504994	-3.020163238	0.002526385	0.029842862	1177
Myo5a	6333.611424	-0.303529484	0.079578223	-3.814227955	0.000136609	0.00505558	6480
Lmo2	194.4695976	-0.303483451	0.097345842	-3.117580019	0.001823424	0.024478429	1529
Csnk2a2	581.2492161	-0.303410472	0.106042505	-2.861215622	0.004220199	0.040832099	3773
Sptbn2	16002.49724	-0.303146369	0.08303187	-3.650964027	0.000261258	0.007606755	8299
Bid	371.590669	-0.303141375	0.089514142	-3.386519345	0.000707853	0.013788547	2189
Sdr39u1	1120.338278	-0.303005419	0.074867302	-4.047233051	5.18E-05	0.002740802	1255
Ttll12	1020.236329	-0.302906579	0.096174161	-3.149562985	0.001635149	0.022883158	3774
Tbc1d10b	4872.705158	-0.302769433	0.07767533	-3.89788409	9.70E-05	0.004057841	3614
Acox1	1753.570235	-0.302757936	0.099120011	-3.054458265	0.002254674	0.027938764	3538
Frs3	1228.763902	-0.30270935	0.09800328	-3.088767532	0.002009886	0.026050065	2144
Ntpcr	289.6119491	-0.302414546	0.086443366	-3.498412425	0.000468037	0.010689474	1155
Snx32	1514.739242	-0.302406409	0.0859165	-3.519771023	0.00043192	0.01018662	1703
Tomm22	785.0766405	-0.302328523	0.086792341	-3.483354878	0.000495172	0.01109593	1569
Rarres1	1049.619195	-0.302267322	0.10048168	-3.008183393	0.002628145	0.03062454	1325
Ank1	15442.56151	-0.302215896	0.104812585	-2.883393211	0.00393416	0.039099309	7206
Wnt5a	1035.761054	-0.302128223	0.073063333	-4.135155204	3.55E-05	0.002159977	7010
Fkbp2	550.2664599	-0.302083368	0.102749449	-2.939999886	0.003282124	0.034905314	642
Tmem115	1667.04837	-0.301852404	0.097852816	-3.084759492	0.002037168	0.026242044	2194
Sgsm1	3264.1622	-0.301802316	0.106270056	-2.839956319	0.004511971	0.042519054	5161
Ahsa1	2694.836734	-0.301791645	0.079060786	-3.817210298	0.000134969	0.005015991	1377
Tmem59l	5246.558009	-0.301710406	0.098718373	-3.056274109	0.002241063	0.02789873	1491
Alkbh4	284.6791159	-0.301685936	0.094776813	-3.18311966	0.001456974	0.021338603	1773
Adam15	2164.51602	-0.30163664	0.066900716	-4.508720653	6.52E-06	0.000742846	2799
Mettl22	506.9922233	-0.30149235	0.069537724	-4.335666039	1.45E-05	0.001244005	1825
Ndufa8	1452.675027	-0.301361677	0.077937731	-3.866698096	0.000110319	0.004397252	861
Dlgap1	1118.260939	-0.301231252	0.088947771	-3.386608212	0.000707624	0.013788547	5276
Gpc1	8858.626039	-0.301179632	0.072103346	-4.177054876	2.95E-05	0.001916512	4176
Sart1	1004.895082	-0.30115336	0.105194997	-2.862810671	0.004199014	0.040680839	5091
Cbx7	814.6005237	-0.301109434	0.075614874	-3.982145533	6.83E-05	0.003225436	2899
Pcp4	1062.28423	-0.301067518	0.097901722	-3.07520147	0.002103604	0.026799464	641
Mrpl43	1260.242423	-0.300976455	0.080789821	-3.725425463	0.000194986	0.006332821	1398
Kcnj4	669.0197611	-0.300974088	0.09122142	-3.29937955	0.000968988	0.016710547	2152
Cacna1a	9258.350361	-0.300867317	0.104777149	-2.871497447	0.004085321	0.040090483	7926
Actl6b	646.2341473	-0.300673322	0.095397108	-3.151807523	0.001622632	0.022784738	1592
Lrrc28	249.488878	-0.300540481	0.105608321	-2.84580303	0.004429959	0.042047309	2923
Lpin1	3092.097245	-0.300508223	0.092913592	-3.234276244	0.001219514	0.019127249	4114
Fancf	249.6069822	-0.300423587	0.10238948	-2.934125533	0.003344889	0.035279397	1691
Zbtb7a	8195.010388	-0.300395649	0.069286412	-4.33556363	1.45E-05	0.001244005	5938
Zfhx3	16600.83844	-0.300343754	0.077704362	-3.865210968	0.000110993	0.004416124	16433
Rexo1	2280.408949	-0.300287117	0.076988101	-3.900435425	9.60E-05	0.004027264	5250
Orai2	1866.243319	-0.300250534	0.08014187	-3.746487765	0.000179328	0.00600011	3908
Fth1	19686.96198	-0.30013177	0.098460099	-3.048257846	0.002301723	0.028266835	929
Riok3	854.0677271	-0.299961563	0.08862588	-3.384582075	0.000712867	0.013843286	3790
Alg1	839.6251662	-0.29987175	0.087472973	-3.428164616	0.000607677	0.012525323	1755
Mrps18b	426.2606859	-0.299813627	0.092070512	-3.256348009	0.001128553	0.018241488	1079
Gnao1	6938.144487	-0.299196657	0.079089665	-3.783005762	0.000154946	0.005475915	2948
Eef2	54384.20675	-0.299137105	0.098557847	-3.035142431	0.002404221	0.029059838	3089
Cox7a2l	2980.953536	-0.299082056	0.079206128	-3.775996411	0.000159369	0.005566452	1076
Mrpl27	481.185086	-0.299071707	0.087530048	-3.416789008	0.000633644	0.012873449	756
Asic1	1781.372421	-0.29898936	0.089727441	-3.332195324	0.000861638	0.015634295	1818
Pitpnm1	3734.803037	-0.298929099	0.094104016	-3.176581731	0.001490218	0.021651497	4206
Gabbr1	7913.531933	-0.298918049	0.104039253	-2.873127589	0.004064299	0.039965762	5248
Etl4	683.9797066	-0.298914544	0.065686945	-4.550592879	5.35E-06	0.000646128	6635
Lypla2	4354.275209	-0.298782557	0.063616721	-4.696604163	2.65E-06	0.00040381	1626
Tprgl	6856.523862	-0.298738065	0.062586821	-4.773178471	1.81E-06	0.000307825	1724
Scg3	6265.518303	-0.298678405	0.087760792	-3.403323956	0.000665713	0.013311044	2152
Washc1	840.9935281	-0.298613081	0.077090821	-3.873523173	0.000107273	0.004339638	1831
Lrfn3	1114.519164	-0.298607757	0.088517827	-3.373419436	0.000742408	0.01415436	2886
Crmp1	2116.08027	-0.298576664	0.086986094	-3.432464328	0.000598123	0.01238063	2906
Dnlz	1418.164411	-0.298572238	0.084767353	-3.522255073	0.000427892	0.010141392	2916
Fbxl19	2142.63459	-0.29804815	0.077090692	-3.866201494	0.000110544	0.004402219	3410
Abcg4	1221.761024	-0.298007665	0.103588613	-2.876838066	0.004016817	0.039712018	3999
Sqstm1	12792.27376	-0.297649997	0.06898811	-4.314511539	1.60E-05	0.001316799	2037
Chst8	1584.422269	-0.297642977	0.105079117	-2.8325607	0.00461768	0.0431124	2372

Bcar3	1038.31162	-0.29757626	0.101508283	-2.9315466	0.003372788	0.035432403	3311
Sh3yl1	239.1003234	-0.297519459	0.080983429	-3.673831332	0.00023894	0.007210033	1750
Gatd1	964.9944415	-0.297393877	0.074865278	-3.972387268	7.12E-05	0.003320987	1898
Dgkq	942.6959168	-0.297211026	0.099983477	-2.972601439	0.002952876	0.032741723	4938
Numa1	2652.719206	-0.297112845	0.107317327	-2.768544958	0.005630722	0.048445142	2934
Rtl8a	5837.205278	-0.297000505	0.06744498	-4.403596925	1.06E-05	0.001034003	1195
Dtd1	374.3741527	-0.296955647	0.092990258	-3.193406002	0.001406051	0.020905321	1350
Rnf10	7209.203005	-0.296797869	0.093156381	-3.186017578	0.001442458	0.021209927	3112
Ipo4	3704.322648	-0.296781983	0.093488446	-3.174531129	0.001500788	0.021754745	3846
Bysl	856.3958502	-0.296735641	0.072057871	-4.118018445	3.82E-05	0.002264281	3854
Tle1	926.1672519	-0.296676674	0.056501551	-5.250770435	1.51E-07	5.89E-05	2067
Dctn2	11739.42668	-0.296553281	0.076402048	-3.881483393	0.000103821	0.004253959	1784
Trpv1	1995.526766	-0.296495727	0.099774441	-2.971660113	0.002961944	0.032801477	3361
Ftl1	10321.72555	-0.296376283	0.099138124	-2.989528857	0.002794081	0.031651613	968
Mgat2	1377.156257	-0.296334079	0.088869963	-3.33446836	0.000854626	0.015552004	2614
Mapk11	2204.634078	-0.29632704	0.088445772	-3.350381062	0.000807005	0.014950277	2461
Plcb3	11075.01018	-0.296146134	0.081184641	-3.647809885	0.000264485	0.007659844	4257
Iqsec2	2227.225829	-0.29611236	0.085647602	-3.457333947	0.000545548	0.011741383	5967
Zpr1	850.4501256	-0.296053101	0.092827404	-3.189285598	0.001426249	0.021053292	2984
Pygb	25360.34525	-0.295983746	0.084487208	-3.503296571	0.000459538	0.010588871	3909
Chd3	3830.943748	-0.295407822	0.083057482	-3.556667196	0.000375589	0.009461163	7261
Rnf126	1762.431311	-0.295397044	0.089517576	-3.299877603	0.00096727	0.016710547	1639
Klhdc2	3674.832293	-0.295309862	0.059607741	-4.954219959	7.26E-07	0.000164576	1905
Ddx27	787.860853	-0.295233886	0.063436345	-4.654017885	3.26E-06	0.000455792	2714
Ldhd	357.6889187	-0.29515569	0.099587473	-2.963783308	0.003038822	0.03341716	2273
Ddx28	347.1581009	-0.295088087	0.102221002	-2.886765751	0.003892239	0.038901349	2262
Lonp1	1590.136434	-0.294971853	0.078344706	-3.765051514	0.000166515	0.00573317	2951
Dnajc7	908.6284425	-0.294934689	0.08171101	-3.609485303	0.000306805	0.008409407	1828
Atp6v1f	1991.664657	-0.294896955	0.081767127	-3.606546612	0.000310299	0.008471577	703
Mex3d	1440.81979	-0.294804613	0.097649961	-3.018993663	0.002536158	0.029901368	3266
Atpif1	2332.515571	-0.294698189	0.086619744	-3.402205731	0.000668443	0.013333982	539
Pmvk	787.6160062	-0.294637024	0.091962251	-3.20389097	0.001355838	0.020438065	1194
Nt5m	438.7663356	-0.294237333	0.091960599	-3.199602167	0.001376174	0.020633529	1306
Abr	835.0753396	-0.294206632	0.09906288	-2.969897824	0.002978988	0.032929245	6271
Pip5k1c	10692.32256	-0.29401642	0.071720702	-4.099463767	4.14E-05	0.002389276	4208
Stk25	1033.773869	-0.293970479	0.09857785	-2.982114938	0.002862645	0.032163606	4249
Ppp3cc	737.9243056	-0.29383318	0.104954784	-2.799616841	0.00511633	0.045710106	1970
Igsf8	3091.177602	-0.293831267	0.091479642	-3.2119853	0.001318211	0.020116333	2278
Mark2	2818.834687	-0.293823831	0.068155341	-4.311090306	1.62E-05	0.001322626	4488
Trim3	1504.189536	-0.293822073	0.093939293	-3.127786732	0.001761279	0.023925867	2841
Dhx38	1317.830811	-0.293783598	0.087083371	-3.373590087	0.000741948	0.014151726	4463
Ap2s1	3322.65011	-0.29374405	0.087784652	-3.346189147	0.000819305	0.015122056	836
Kansl3	2470.487373	-0.293737354	0.090542139	-3.244206022	0.001177785	0.018731519	4663
Atp1a1	93271.86988	-0.293731141	0.083236317	-3.528882015	0.000417319	0.010026166	3679
Palld	1116.41549	-0.293680989	0.082017963	-3.58069107	0.000342687	0.008914922	4193
Naaa	870.5517421	-0.293668575	0.071777342	-4.091382677	4.29E-05	0.002444076	2414
Lysmd2	1321.244734	-0.293667717	0.09807995	-2.994166659	0.002751956	0.031365611	1189
Brd3	1359.894916	-0.293527237	0.100751924	-2.913366073	0.003575552	0.036754532	5272
Pkm	22077.94507	-0.293152312	0.078473289	-3.735695505	0.000187197	0.0061882	2041
Galnt18	1795.965671	-0.293092262	0.080785318	-3.628038722	0.000285582	0.008043326	2530
Trpc3	1919.44526	-0.293026924	0.105858925	-2.768088969	0.005638606	0.048503486	3694
Ptges2	680.1073125	-0.29293708	0.056008673	-5.230209245	1.69E-07	6.47E-05	4978
Ttc39c	611.7328973	-0.292927637	0.085221836	-3.437236866	0.000587681	0.012244191	1950
Zbtb45	824.2272415	-0.292920373	0.097666003	-2.999205096	0.00270685	0.031128399	2171
Bckdha	1691.987182	-0.292916574	0.090968865	-3.219965131	0.001282062	0.019780656	1899
Mettl21a	217.6122831	-0.292808717	0.091483562	-3.200670248	0.001371083	0.020594357	2030
Srp68	2066.940255	-0.29265955	0.103278453	-2.833694174	0.004601334	0.043015059	2526
Fxr2	7108.388203	-0.292633258	0.057624579	-5.078271474	3.81E-07	0.000107343	2951
Fgf13	819.2995163	-0.292625491	0.085855483	-3.408349491	0.000653571	0.013138484	2499
Mrpl28	854.7336486	-0.29249261	0.061295573	-4.771839071	1.83E-06	0.000307851	1068
Bsdc1	2152.86435	-0.292398959	0.086132343	-3.394763767	0.000686878	0.013554133	2823
Tysnd1	1133.108293	-0.292386726	0.104184814	-2.806423652	0.005009478	0.045085304	2286
Thsd7b	544.6366667	-0.292382118	0.080887891	-3.614658703	0.000300744	0.008332066	6213
Smn1	257.9312173	-0.292288767	0.097865771	-2.986629186	0.002820716	0.031833297	1222
Pdgfa	1625.982588	-0.292249032	0.076250623	-3.832742872	0.000126722	0.004840443	2060
Ppm1j	3709.326333	-0.292167309	0.103006501	-2.83639678	0.004562572	0.042779589	1721
Gcsh	830.3387427	-0.292146398	0.058509395	-4.99315365	5.94E-07	0.000141934	1443
Cog8	1186.612474	-0.292107475	0.084138954	-3.471726957	0.000517122	0.011379014	2954
Isoc2a	470.2010584	-0.292005795	0.077378109	-3.773752022	0.000160811	0.005588963	2244
Eri3	3581.640409	-0.291838492	0.065571522	-4.450689605	8.56E-06	0.000895668	1719
Hsdl1	3928.053322	-0.291802574	0.077279384	-3.775943313	0.000159403	0.005566452	3141

Apc	1629.575609	-0.291764381	0.08386037	-3.479168772	0.000502972	0.011219255	3588
Utp3	486.6350657	-0.291728198	0.079235645	-3.681779826	0.000231611	0.007105928	1629
Serf2	422.2831283	-0.291587229	0.089287557	-3.265709546	0.001091902	0.017871884	525
Mrtfa	1058.301362	-0.291572418	0.07196285	-4.051707519	5.08E-05	0.002709587	4366
Prr5	998.3474647	-0.291528172	0.072333505	-4.030333844	5.57E-05	0.002830924	1719
Vps28	2765.876618	-0.291458873	0.062980058	-4.627796219	3.70E-06	0.000503544	911
2310011J0	817.1660742	-0.291432239	0.099676737	-2.923773863	0.003458159	0.03602795	1649
Nkain4	157.1062854	-0.291401766	0.096080305	-3.032898023	0.002422174	0.029151628	950
Zswim4	1949.14	-0.291349266	0.077472341	-3.760687527	0.000169447	0.005779923	4625
Smurf1	1939.682273	-0.291297128	0.072506299	-4.017542393	5.88E-05	0.002916448	5343
Igsf21	2326.538469	-0.291291295	0.098644213	-2.952948631	0.003147543	0.034108036	1979
Flywch1	1190.81408	-0.291234473	0.100509016	-2.897595494	0.003760352	0.038145799	665
Cfl1	11047.18332	-0.291224879	0.079192286	-3.677439964	0.000235586	0.007157955	2354
Ndufa2	440.4389307	-0.291098284	0.100090929	-2.90833832	0.00363355	0.037202848	572
Ciao3	560.6114343	-0.291027571	0.071115302	-4.092334011	4.27E-05	0.002444076	2555
Alkbh6	562.2144725	-0.290954161	0.105058544	-2.76944786	0.005615139	0.048378835	982
Msantd3	239.5426516	-0.290915036	0.078639151	-3.699366464	0.000216138	0.006797049	1597
Itfg2	746.0682649	-0.290821136	0.068160668	-4.266700182	1.98E-05	0.001498093	2320
Gcc1	1003.374381	-0.290760493	0.102994551	-2.823066762	0.004756667	0.043860496	4650
Pim2	995.0162701	-0.290691606	0.098918464	-2.938699167	0.003295928	0.034980616	2045
Plekha2	596.0619499	-0.290586966	0.104365895	-2.784309621	0.00536418	0.047120116	3463
Mrpl4	1434.558196	-0.290545731	0.098836326	-2.939665446	0.003285668	0.034917669	1288
Elp3	1999.12766	-0.290379633	0.069488428	-4.178819979	2.93E-05	0.001905749	2851
Asic2	2926.249594	-0.290227695	0.089640145	-3.237697725	0.001204984	0.018995025	3436
Arhgap24	1038.539245	-0.290139133	0.082345091	-3.523453899	0.000425961	0.01012291	3346
Eif4h	5980.683333	-0.290098885	0.083089551	-3.491400335	0.000480496	0.010861179	2367
Tpst2	1183.483967	-0.289954569	0.097632804	-2.969847809	0.002979473	0.032929245	1813
Cnbp	562.8758578	-0.289921903	0.09106338	-3.183737565	0.001453868	0.021313534	2178
Tmem109	1781.860971	-0.289654107	0.090257315	-3.209203677	0.001331032	0.020230206	2188
Thrap3	3337.138442	-0.289524836	0.097498432	-2.969533251	0.002982525	0.032946414	4378
Lgmn	4468.918685	-0.289198022	0.081815788	-3.534745913	0.000408167	0.009898004	1835
Tmem234	870.6784034	-0.289185166	0.081095479	-3.565983817	0.000362494	0.009234749	1437
Mthfsd	550.3205253	-0.289100888	0.094094871	-3.072440444	0.002123162	0.026931568	1366
Nprl2	522.7243977	-0.289022808	0.07831949	-3.69030504	0.000223985	0.00694465	1441
Tmem151a	10122.09185	-0.288859998	0.095563384	-3.022705835	0.002505256	0.029728367	4006
Fgfr1	7226.515194	-0.288694935	0.056034826	-5.15206266	2.58E-07	8.27E-05	4046
Pygo2	1901.811208	-0.288447273	0.071665906	-4.024888422	5.70E-05	0.002870131	3198
Bcr	7471.90461	-0.287530296	0.062210236	-4.621912948	3.80E-06	0.000512769	6839
Rbm42	1268.21102	-0.287430614	0.087655626	-3.279089185	0.001041427	0.017415879	1784
Trim36	1763.391066	-0.287382053	0.086746865	-3.312881131	0.000923402	0.016278018	2553
Zbtb18	800.3997858	-0.287329072	0.09963387	-2.883849369	0.003928466	0.039075795	3734
Wbp4	1208.089141	-0.287278427	0.060171989	-4.774288332	1.80E-06	0.000307825	3704
Tlk1	3092.137848	-0.28714366	0.062064518	-4.626534907	3.72E-06	0.000503544	4280
Clstn1	38234.64647	-0.286986225	0.055075349	-5.210792687	1.88E-07	7.00E-05	4459
Ap1ar	2036.223407	-0.286690801	0.086998767	-3.295343265	0.000983015	0.016840762	2580
Myh14	4045.201189	-0.286645298	0.091812777	-3.122063262	0.001795883	0.02425684	6499
Mad2l1bp	284.0053075	-0.286569837	0.092137056	-3.110256052	0.001869252	0.024865863	1272
Tmem64	1562.124328	-0.286493116	0.09970823	-2.873314636	0.004061894	0.039961573	4670
Pdcd6	591.7578109	-0.286490534	0.092090914	-3.110953315	0.001864844	0.024851453	1131
Ruvbl1	654.0966532	-0.286452604	0.060959977	-4.699027465	2.61E-06	0.000400439	1675
Lhfpl4	15029.70927	-0.286353006	0.091990337	-3.112859638	0.001852841	0.024754649	4762
Fam32a	1834.174442	-0.286323875	0.085313391	-3.356142238	0.000790379	0.014736648	2564
Map7d1	8008.013221	-0.286236777	0.099878404	-2.865852516	0.00415888	0.040461416	3195
Jph3	8917.122515	-0.286233644	0.08730557	-3.278526709	0.001043505	0.01744399	3637
Apba1	28042.21852	-0.286192975	0.082833395	-3.455043418	0.000550204	0.011774414	6620
Map1s	4075.843838	-0.286175971	0.077315687	-3.701395943	0.000214417	0.006777013	3314
Cwc25	616.308181	-0.286024649	0.10218425	-2.799106981	0.005124416	0.045749717	3105
Paqr4	7727.642567	-0.28598078	0.099164084	-2.883914923	0.003927648	0.039075795	2575
Tmem104	2215.793016	-0.285973438	0.079312031	-3.60567537	0.000311342	0.008480748	4564
Syf2	705.2405524	-0.285960767	0.085190062	-3.356738568	0.000788676	0.014717386	1369
Grm4	3091.072288	-0.28588329	0.073210536	-3.904947369	9.42E-05	0.003988454	4607
Slc9a7	468.8879452	-0.285746778	0.081698474	-3.4975779	0.000469504	0.010700739	1151
Nelfb	1792.546301	-0.285731929	0.081005043	-3.52733507	0.000419765	0.010068181	2637
Smg9	1350.812217	-0.285571249	0.081148171	-3.519133503	0.000432959	0.01019006	2242
Vps33b	519.1827782	-0.285536444	0.081585168	-3.499857286	0.000465507	0.010670505	2574
Tmem150c	1677.606614	-0.285451815	0.072331465	-3.946440387	7.93E-05	0.003576797	3084
Prkaca	12385.94572	-0.285435355	0.065970506	-4.326711585	1.51E-05	0.001269883	2276
Trappc9	1842.969744	-0.285280597	0.06392934	-4.462436136	8.10E-06	0.00086262	3301
Fzr1	1269.031345	-0.284857521	0.080174584	-3.552965384	0.000380915	0.009467961	3057
Pitrm1	705.0034547	-0.284723279	0.064513998	-4.413356601	1.02E-05	0.00100331	3318
Kbtbd11	2757.924302	-0.284708157	0.084947033	-3.351596264	0.000803471	0.014904896	6973

Crtc1	3911.786242	-0.284570412	0.096121873	-2.960516723	0.003071234	0.033659894	5683
Med27	919.636791	-0.284532082	0.060659937	-4.6906096	2.72E-06	0.000408944	1254
Ephx1	2677.433214	-0.284468739	0.071546842	-3.975978968	7.01E-05	0.003290325	1741
Camk1g	886.9841568	-0.284399152	0.080515434	-3.532231492	0.000412068	0.00995417	2469
Rassf5	3906.782985	-0.284381039	0.081473132	-3.490488626	0.000482138	0.01088109	3485
Thra	7443.45954	-0.284296711	0.07210225	-3.942965848	8.05E-05	0.0035995	2452
Mark1	3519.443106	-0.284164082	0.082275095	-3.453828674	0.000552688	0.011793424	4221
Syp	17990.76801	-0.284149148	0.084949875	-3.344903649	0.000823112	0.015172579	2591
Tbcb	1143.664944	-0.284146068	0.077240003	-3.678742322	0.000234387	0.007146201	1416
Dnpep	935.5275665	-0.284036465	0.084961004	-3.343139229	0.000828363	0.015218968	1571
Mcrs1	1468.228117	-0.283966112	0.075288335	-3.771714607	0.00016213	0.005615494	1916
Fmnl1	2746.659781	-0.283940425	0.078053737	-3.637755689	0.000275024	0.007856683	3824
Flii	7340.611309	-0.283575197	0.063648065	-4.455362434	8.38E-06	0.000880894	4060
Anapc5	1529.300971	-0.283473485	0.068300527	-4.150385038	3.32E-05	0.002049537	2760
Aig1	1054.481215	-0.28330953	0.078730994	-3.598449827	0.00032012	0.008595206	1554
Mxra7	1119.193949	-0.283292322	0.063167208	-4.484800417	7.30E-06	0.000806197	2063
Chmp6	826.1236677	-0.282925233	0.101204183	-2.795588347	0.005180534	0.046040465	1557
Trim67	7191.604514	-0.282614453	0.097703745	-2.8925652	0.003821099	0.038516104	8735
Fcsk	401.1889772	-0.282589489	0.096499445	-2.928405343	0.003407056	0.035664571	3838
Unc13a	2230.965998	-0.282530958	0.071436718	-3.954982331	7.65E-05	0.003490762	5654
Pelo	576.7551585	-0.282509772	0.09624468	-2.93532872	0.003331945	0.035231189	1600
Gpn3	375.0835992	-0.28249225	0.098484287	-2.868399203	0.004125546	0.040333476	1592
Rbck1	2194.62024	-0.282449169	0.073345236	-3.850954541	0.000117658	0.004589928	2384
Mgat4b	2873.871089	-0.282444616	0.078166389	-3.613376784	0.000302235	0.008346584	2429
Gng3	5718.599575	-0.282393179	0.082055892	-3.441473507	0.000578555	0.012113579	1643
Wbp2	12420.64739	-0.282386214	0.069676372	-4.052826006	5.06E-05	0.002709587	1826
Akt1s1	1941.195909	-0.282289431	0.093459029	-3.020461833	0.002523895	0.029840362	1592
Cib2	432.409175	-0.282262483	0.100923276	-2.796802606	0.005161105	0.045914205	1433
Mrps7	676.4788498	-0.282249575	0.067331991	-4.191908922	2.77E-05	0.001834046	1962
Ndufa6	568.3549729	-0.282186159	0.091310901	-3.090388506	0.001998948	0.025947377	590
Wfs1	3887.149193	-0.28211547	0.079065481	-3.568124393	0.000359546	0.009179693	3787
Ggact	612.5105698	-0.282008475	0.069310251	-4.068784535	4.73E-05	0.00259186	1076
Hspa4l	1089.025279	-0.281989086	0.084635528	-3.331805117	0.000862847	0.015643319	2744
Tle4	2729.393679	-0.281982358	0.096071024	-2.935144697	0.003333922	0.035234585	4545
Ankrd52	15938.06711	-0.281922488	0.081125181	-3.475153896	0.00051056	0.01128417	6620
Borcs5	884.1400204	-0.281865357	0.052941931	-5.324047553	1.01E-07	4.37E-05	1764
Brf1	1342.071405	-0.281863915	0.096978857	-2.906447065	0.003655587	0.037352379	2615
Pam16l	276.1138257	-0.281807067	0.085432242	-3.298603209	0.000971672	0.016723026	563
Hs6st1	5346.464304	-0.281794669	0.078469081	-3.591155478	0.000329215	0.008698284	3719
Pa2g4	1022.438209	-0.281461273	0.101703487	-2.76746924	0.005649338	0.048519853	2483
Tnfrsf21	9296.273912	-0.281444825	0.086269873	-3.262376712	0.001104822	0.018030255	3627
Guk1	1153.852109	-0.281424055	0.071820192	-3.918453109	8.91E-05	0.003845063	1078
Fam207a	1070.000909	-0.281410317	0.088472503	-3.180765868	0.001468863	0.021464956	2394
Jup	10537.00976	-0.281225524	0.08809587	-3.192266817	0.001411609	0.020938386	3205
Dclk3	1423.163137	-0.281022282	0.053891547	-5.214589266	1.84E-07	6.92E-05	3005
Mapk4	1733.526031	-0.280930245	0.09041109	-3.10725427	0.001888339	0.025028889	4594
Ctif	10853.37632	-0.280764794	0.07209869	-3.894173282	9.85E-05	0.004098445	6031
Dapk1	2266.948088	-0.280711052	0.083187132	-3.374452822	0.000739626	0.014113565	5491
Armc5	1498.251686	-0.2805342	0.068553806	-4.092175421	4.27E-05	0.002444076	3834
Ttc9	3992.845385	-0.280469481	0.086307338	-3.249659748	0.001155432	0.018559938	2042
Pard3	863.3457033	-0.280461392	0.100903396	-2.779504001	0.005444198	0.04755696	5659
Tmem250-	7962.878715	-0.280418499	0.091264836	-3.07257987	0.00212217	0.026931568	2483
Eme2	401.2640071	-0.280416607	0.087784182	-3.194386499	0.001401284	0.020862662	1521
Mtfmt	244.7884142	-0.280379358	0.073877286	-3.795203805	0.000147522	0.005317833	2042
Pitpna	3689.733105	-0.280265937	0.064935062	-4.316095623	1.59E-05	0.001314919	1665
Cct3	2999.269934	-0.280232751	0.071066713	-3.943235009	8.04E-05	0.0035995	1977
Cep250	1143.402626	-0.280216143	0.082170494	-3.410179583	0.000649201	0.013074727	7977
Smap1	1848.733203	-0.280203604	0.059294099	-4.725657524	2.29E-06	0.00036409	2289
Mrpl46	330.6692579	-0.280143036	0.074101002	-3.780556673	0.000156478	0.005501347	1134
Eif2b2	929.561277	-0.28013763	0.057375719	-4.882511868	1.05E-06	0.000215188	1605
Mpped2	593.2062733	-0.280040506	0.078852964	-3.551426494	0.000383149	0.00950629	2848
Msrb2	366.1674108	-0.279984486	0.083348217	-3.359213866	0.000781646	0.014660855	1196
Caprin1	3662.427059	-0.279957788	0.081245764	-3.445813966	0.000569342	0.012011098	6169
Rnf167	1532.486835	-0.279813099	0.082218249	-3.403296746	0.000665779	0.013311044	1833
Niban2	5477.474196	-0.279808533	0.097979786	-2.855778177	0.00429315	0.041247448	3714
Msrb1	1206.648253	-0.279781159	0.069343247	-4.034728254	5.47E-05	0.002804366	886
Lrpap1	4560.488066	-0.279766707	0.067383206	-4.15187587	3.30E-05	0.002039092	2981
Txndc15	1005.03201	-0.279516535	0.081238679	-3.44068293	0.000580248	0.012136349	1527
Lcp1	1072.901134	-0.27940482	0.098776622	-2.828653313	0.004674431	0.043378537	2523
Thy1	30389.17696	-0.279213734	0.08774884	-3.181964963	0.001462795	0.021401593	1735
Ogfrl1	4826.021975	-0.279203255	0.094682366	-2.948841121	0.003189679	0.034396429	4844

Ttl	7690.12177	-0.279108953	0.087649645	-3.184370615	0.001450691	0.021290919	4567
Mapk8ip3	16412.42068	-0.279031275	0.092567027	-3.014370055	0.002575135	0.030206993	5418
Tmem242	697.0097017	-0.27896353	0.090677813	-3.07642544	0.002094987	0.026743931	948
Rad23b	5608.145594	-0.278490092	0.070480706	-3.951295452	7.77E-05	0.003530339	3810
Cpne2	1194.12845	-0.278418536	0.093014603	-2.993277696	0.002759985	0.0314034	2342
Anxa11	2333.57835	-0.278414109	0.069505347	-4.005650244	6.18E-05	0.003021231	2394
Adcy9	1570.128349	-0.278398872	0.100936819	-2.758149856	0.005812954	0.049413483	4974
Zbtb22	1220.936743	-0.278374418	0.06674004	-4.171025644	3.03E-05	0.001939388	2612
Stim2	1612.683856	-0.278310101	0.053541327	-5.19804266	2.01E-07	7.20E-05	4934
Slc9a3r2	1843.62451	-0.278250298	0.086128752	-3.230631965	0.001235169	0.019303128	2136
Snf8	488.8146099	-0.27824165	0.066355434	-4.193200696	2.75E-05	0.001834046	1004
Dner	7694.527034	-0.278034324	0.086380226	-3.218726535	0.001287612	0.019794418	3632
Bbln	3439.306955	-0.278030224	0.096168593	-2.891070934	0.003839315	0.038599466	680
Brdt	177.3215843	-0.277661136	0.086168483	-3.222305028	0.001271637	0.01970656	4745
Afg3l2	2627.512408	-0.277575582	0.067925499	-4.086471017	4.38E-05	0.002468715	3094
Arf1	6058.675752	-0.277477721	0.072552798	-3.824493733	0.000131041	0.004919915	1800
Dync1li1	5652.611183	-0.277257684	0.06366135	-4.355196419	1.33E-05	0.001185607	2601
Tmem147	828.8786388	-0.277088921	0.078802437	-3.516248096	0.000437692	0.010262997	870
Glrx5	617.4819245	-0.276958582	0.084569221	-3.274933596	0.001056868	0.01754066	2925
Ptpra	2445.75823	-0.27684059	0.064749832	-4.275541413	1.91E-05	0.001464608	3066
Pstpip2	746.6244373	-0.276758725	0.067810143	-4.081376545	4.48E-05	0.002501019	2267
Slc25a38	1023.098114	-0.276747924	0.069883192	-3.960150018	7.49E-05	0.003448269	1791
Wiz	763.9856992	-0.276729204	0.091760714	-3.015769958	0.002563276	0.030124151	4175
Arf3	14106.35602	-0.276715032	0.063811302	-4.336457991	1.45E-05	0.001244005	4566
Sart3	1069.383626	-0.2766848	0.084529413	-3.273236971	0.001063233	0.017584757	4447
Psma7	940.2171641	-0.276667756	0.09141076	-3.026643224	0.002472856	0.029515642	914
Gripap1	1627.049162	-0.276631461	0.051839831	-5.336272483	9.49E-08	4.17E-05	3015
Mien1	1034.182513	-0.276511213	0.079513101	-3.477555391	0.000506008	0.011258192	747
Kmt2c	805.3968473	-0.276311713	0.07517366	-3.675645346	0.000237249	0.007178698	6486
Mthfd2	566.4835482	-0.276264035	0.076642737	-3.60456901	0.000312671	0.008496042	2085
Mdh2	9165.418447	-0.276100579	0.060186086	-4.587448672	4.49E-06	0.000572382	1456
Pacsin1	6766.953914	-0.275851742	0.100075306	-2.75644165	0.005843404	0.049576466	4566
Spock2	127874.1501	-0.275673238	0.089053234	-3.095600514	0.001964148	0.025641599	5238
Bcl9	1883.589055	-0.275602987	0.085030275	-3.241233632	0.001190136	0.018848822	6102
Ik	1261.079539	-0.275584905	0.076881609	-3.584536141	0.000337678	0.008836908	1996
Qsox1	1776.151577	-0.275507746	0.063707992	-4.324539759	1.53E-05	0.00127529	3348
Strap	2696.460125	-0.275393253	0.063018229	-4.370057011	1.24E-05	0.001135358	2650
Nubp2	644.9752543	-0.275313722	0.085617675	-3.215617816	0.001301641	0.019946541	1427
Lancl1	2657.449532	-0.275303154	0.090659871	-3.036659458	0.002392155	0.028958548	4461
Eepd1	2946.005756	-0.27528762	0.091134287	-3.020681116	0.002522068	0.02983927	2723
Sf3b2	6138.024347	-0.274823887	0.0804103	-3.417769717	0.000631365	0.012839021	3222
Hopx	1937.721107	-0.274752756	0.06222106	-4.415751744	1.01E-05	0.000995381	1113
Khdrbs1	3615.371036	-0.274722796	0.079486555	-3.45621715	0.000547814	0.011763883	3738
Gle1	1889.516318	-0.274530156	0.092095361	-2.980933591	0.002873711	0.032238506	4030
Usf2	3076.328597	-0.274363575	0.081243549	-3.377050588	0.000732676	0.014060274	2419
Jpt1	1510.587291	-0.274359636	0.095468155	-2.873834075	0.004055219	0.03992112	1375
Atp2a2	10897.77343	-0.274284086	0.099555322	-2.755092149	0.005867561	0.049737225	4486
Znfx1	2334.310405	-0.274216767	0.092735139	-2.956988801	0.003106594	0.033849171	4147
Sec22c	414.8618461	-0.274211027	0.067900548	-4.038421432	5.38E-05	0.002789917	2669
Rrp1	5595.229059	-0.274190636	0.074003248	-3.70511625	0.000211294	0.006706153	1984
Tbca	675.04211	-0.27412049	0.092477915	-2.964172467	0.003034981	0.033385589	559
Drg2	1682.3627	-0.273982548	0.071864947	-3.812464355	0.000137588	0.005070461	1788
Otud7a	471.5901569	-0.27389775	0.086658253	-3.160665492	0.001574092	0.022389174	2057
Gap43	2938.146937	-0.273889155	0.085217315	-3.214008259	0.001308959	0.020010066	1420
Hspbp1	1882.211706	-0.273866208	0.076645806	-3.573140192	0.000352726	0.009072816	1593
Nudc	1374.288574	-0.273820878	0.091121597	-3.005005263	0.002655763	0.030831695	1316
Snrpc	653.9778714	-0.273792467	0.091101557	-3.005354412	0.002652716	0.030831695	758
Ppp4c	804.9723982	-0.273754165	0.097157399	-2.817635795	0.004837865	0.044192135	1359
Mfsd12	1909.269524	-0.273728052	0.075727726	-3.614634505	0.000300772	0.008332066	3971
Sestd1	867.9610195	-0.273637285	0.093135323	-2.938061258	0.003302718	0.035014608	2720
Gdpd5	2289.526587	-0.273356017	0.078227536	-3.494370768	0.000475181	0.010790969	4611
Tomm5	598.3074004	-0.273312705	0.09748971	-2.803503112	0.005055074	0.045356889	639
Ttc38	539.3101672	-0.273239455	0.078613033	-3.475752597	0.000509422	0.011276581	3749
Cd2bp2	392.7376322	-0.273132291	0.087575477	-3.118821602	0.001815759	0.024404961	3284
Gna14	1204.711254	-0.273057885	0.092050906	-2.966379121	0.003013288	0.033227791	3290
Golga7b	1833.393472	-0.272819444	0.093170133	-2.92818562	0.003409464	0.03568129	2823
Preb	1502.673213	-0.272687714	0.079617169	-3.424986302	0.00061483	0.012619524	1869
Actg1	52591.01731	-0.272629412	0.070242555	-3.88125706	0.000103918	0.004253959	1952
Nptx1	10700.21522	-0.272560511	0.094555111	-2.882557135	0.003944616	0.039147864	5217
Ercc3	1238.651121	-0.272539047	0.065867254	-4.137701675	3.51E-05	0.002139114	2708
Slit1	3801.241096	-0.272304205	0.089690414	-3.036045799	0.00239703	0.028991858	5673

Mrpl10	1302.612579	-0.272297716	0.073493451	-3.705060974	0.00021134	0.006706153	1708
Lrrc59	4708.385543	-0.272165496	0.052806807	-5.153985045	2.55E-07	8.24E-05	2837
Otub1	5613.747776	-0.272115134	0.053338506	-5.101663984	3.37E-07	0.000101863	1713
Ap3d1	8668.30583	-0.271840227	0.058118393	-4.677352774	2.91E-06	0.000431632	4805
Vash1	4952.313597	-0.271734415	0.082359432	-3.299372139	0.000969014	0.016710547	2599
Aggf1	1030.509498	-0.271695948	0.083508841	-3.2534992	0.00113993	0.018384464	3249
Pde6d	1035.608573	-0.271448473	0.098088299	-2.767388921	0.00565073	0.048522331	1159
Bcl6	1760.507378	-0.271446906	0.070063676	-3.874288647	0.000106937	0.00433315	3525
Khsrp	4833.049978	-0.271390968	0.097917087	-2.771640545	0.005577459	0.048202026	3978
Nectin1	3860.073203	-0.271181332	0.095023927	-2.853821556	0.004319679	0.041393783	5653
Aars	6548.44403	-0.271177678	0.075350216	-3.598897166	0.00031957	0.008595206	5750
Ndufs5	764.7947179	-0.271065939	0.060287586	-4.496214813	6.92E-06	0.000777757	525
Ankrd13b	2414.389319	-0.270995683	0.077913083	-3.478179457	0.000504832	0.01123794	3105
Chmp1a	2821.373904	-0.270888585	0.065925361	-4.109019346	3.97E-05	0.002319944	2140
Xab2	1099.463634	-0.270879367	0.091744123	-2.952552786	0.003151582	0.034111102	2677
Ppp2r5b	13219.87036	-0.270824385	0.073579947	-3.680681956	0.000232611	0.007113166	2742
Plekhg5	1088.429747	-0.270711752	0.086628829	-3.124961463	0.001778284	0.024093139	3818
Csnk1d	5600.159114	-0.270613503	0.068003053	-3.979431655	6.91E-05	0.003251726	3500
Tac1	771.4031839	-0.270553873	0.084366976	-3.206869373	0.001341879	0.020312743	691
Dclk2	1962.082023	-0.270459304	0.090155331	-2.999925796	0.002700454	0.03111254	4012
Ppp2r2c	13856.13105	-0.270409055	0.070053296	-3.860047575	0.000113365	0.004478068	4088
Crip2	9049.663613	-0.270365038	0.077931849	-3.469249621	0.000521914	0.011450081	1439
Pef1	2566.554842	-0.270302711	0.05878558	-4.598112522	4.26E-06	0.000559353	1551
Mthfd1	1136.529837	-0.270290058	0.093081815	-2.903790151	0.003686752	0.037572564	3241
Rbm17	775.7132897	-0.270153311	0.081213741	-3.326448316	0.000879603	0.015822443	1599
Zc3h13	864.1036727	-0.270138534	0.093212922	-2.898080304	0.003754544	0.038110626	6144
Cnn3	6962.736911	-0.270107941	0.081505384	-3.313988954	0.000919751	0.01623547	2049
Golga2	4061.388026	-0.269933276	0.068455352	-3.943201912	8.04E-05	0.0035995	4373
Wdr13	2444.833774	-0.269787073	0.074982406	-3.598005033	0.000320667	0.008595206	4183
Rab15	5639.759011	-0.269736231	0.087937997	-3.067345653	0.00215969	0.027245554	3158
Lonrf1	496.6285177	-0.269581405	0.08299112	-3.248316272	0.001160902	0.018573157	3930
Lrsam1	7484.520091	-0.269531552	0.093103547	-2.894965466	0.003792002	0.038316568	4030
Ppp6r1	8141.673978	-0.269505096	0.063706119	-4.230442835	2.33E-05	0.001653876	3862
Uqcrq	1082.580607	-0.269471584	0.077876699	-3.460233788	0.000539707	0.011694529	1521
Svop	2484.872158	-0.269463001	0.079412835	-3.393192083	0.000690832	0.013591189	3379
Slu7	877.7793131	-0.269328041	0.051777666	-5.201625782	1.98E-07	7.17E-05	3625
Ubap1	1615.648254	-0.269303301	0.065196491	-4.130641045	3.62E-05	0.002187685	3456
Emc1	2578.343491	-0.269049772	0.055598723	-4.839135785	1.30E-06	0.000249272	6232
Nrbf2	723.448352	-0.268954904	0.095403942	-2.819117313	0.004815591	0.044090688	1813
Crbn	1741.392139	-0.268917925	0.073391859	-3.664138321	0.000248173	0.007394167	2084
Armc6	940.047156	-0.268786458	0.097540925	-2.755627519	0.005857967	0.04969043	2263
Fam219a	9679.792199	-0.268778117	0.077773634	-3.455902753	0.000548453	0.011763883	3357
Sgsm3	1405.818421	-0.268754654	0.068867005	-3.902516966	9.52E-05	0.004008966	2997
Mrgpre	795.5463375	-0.268728922	0.080804566	-3.325665073	0.000882079	0.015829516	3761
Mrps2	724.3733734	-0.26872056	0.061279752	-4.385144379	1.16E-05	0.0010959	2080
Stub1	401.2302957	-0.268635699	0.077531034	-3.464879587	0.000530469	0.011585726	1803
Edem2	967.4510765	-0.268608648	0.094924338	-2.82971314	0.004658976	0.043332317	2290
Def8	1307.449992	-0.268313432	0.075848793	-3.537477945	0.000403968	0.009860072	3127
Trim33	1154.483967	-0.268236248	0.090724616	-2.956598331	0.00311053	0.033883663	5025
Smim12	828.2649926	-0.267716044	0.074068959	-3.61441617	0.000301025	0.008332066	1022
C2cd5	388.9233165	-0.267711733	0.095053681	-2.816426775	0.004856111	0.04431277	3332
Tufm	1822.563003	-0.267673532	0.074404218	-3.597558555	0.000321218	0.00859547	1679
Sh3bp5l	2476.014332	-0.267652653	0.092834226	-2.883124723	0.003937515	0.039099309	2977
Cby1	585.166163	-0.267341055	0.078860059	-3.390069173	0.00069875	0.013659644	1147
Pgp	2373.420001	-0.267130483	0.079829296	-3.346271292	0.000819062	0.015122056	2627
Cnpy3	1554.133812	-0.267121642	0.093456402	-2.858248723	0.004259863	0.041089271	1908
Isl1	2275.043487	-0.267094395	0.072176457	-3.700575023	0.000215111	0.00679363	2516
Brd4	4524.763232	-0.266966241	0.077898607	-3.427099045	0.000610067	0.0125619	5922
Cacng7	4996.749412	-0.266741247	0.096434919	-2.766023445	0.005674446	0.048668942	2212
Snrpb2	345.5662614	-0.266732806	0.061011306	-4.371858673	1.23E-05	0.001132119	2061
Gdi2	1136.120102	-0.266661367	0.077994729	-3.418966501	0.000628595	0.012823952	1107
Tmem62	539.8672975	-0.266610172	0.080726721	-3.30262604	0.00095784	0.016631694	2921
Rab11b	5397.316087	-0.26630193	0.069218121	-3.847286312	0.000119433	0.0046345	1544
Dda1	1808.322561	-0.266096514	0.049136386	-5.41546775	6.11E-08	2.99E-05	1933
Rpusd4	214.4510151	-0.266041543	0.080849629	-3.290572203	0.000999838	0.017018155	1821
Rmnd5b	1032.849528	-0.265963946	0.081861766	-3.248939763	0.00115836	0.018573122	1946
Tmem192	412.5739682	-0.265944245	0.063433085	-4.192516331	2.76E-05	0.001834046	1122
Cdr2	1970.774162	-0.265921113	0.095019621	-2.798591605	0.005132601	0.045786708	2525
Cyb5r3	4202.424666	-0.265748328	0.082511022	-3.220761555	0.001278505	0.019766061	1982
Kcmf1	2684.199298	-0.26567125	0.066845022	-3.974435843	7.05E-05	0.003299539	3251
Ampd2	1725.770412	-0.265663405	0.090236505	-2.944079046	0.003239173	0.03471833	3368

Tfip11	2424.560077	-0.265641515	0.068324696	-3.887928216	0.000101104	0.004169999	3560
Stoml1	1904.865541	-0.265629938	0.085881731	-3.092973725	0.001981617	0.025771876	1984
Mrpl49	735.5964595	-0.265584866	0.088711534	-2.993803102	0.002755237	0.031365611	1764
Tmem184b	4217.251422	-0.26557227	0.061572873	-4.313137541	1.61E-05	0.001316799	3315
Rnf41	2190.114337	-0.265435867	0.077758248	-3.413604007	0.000641097	0.012970926	3144
Mecr	388.5262115	-0.265271768	0.076556675	-3.465037733	0.000530157	0.01158467	1337
Megf8	20179.05319	-0.265240655	0.072889798	-3.638927028	0.000273776	0.007836313	10040
Ypel3	5884.052846	-0.264992319	0.075207323	-3.523490917	0.000425902	0.01012291	973
Tmem259	2759.948599	-0.264897505	0.062927842	-4.209543791	2.56E-05	0.001766101	2225
Sema4f	3407.9076	-0.264787329	0.081066886	-3.266282222	0.001089696	0.017849657	4086
Larp4b	963.6952174	-0.26475006	0.087460202	-3.027091788	0.00246919	0.029483412	5720
Arhgef11	9572.49937	-0.264739765	0.073538205	-3.600030268	0.00031818	0.00858208	6776
Magee1	9938.036238	-0.264658523	0.075551427	-3.503024797	0.000460007	0.010594129	3550
Rpl13a	1352.709826	-0.26459126	0.080523571	-3.285885831	0.001016622	0.017170876	552
Fam234a	688.4464348	-0.26455647	0.083640048	-3.163035843	0.001561331	0.0222653	2696
Fkbp1b	2474.00691	-0.264498142	0.072057037	-3.670677484	0.000241908	0.007269654	976
Zswim8	5541.650537	-0.264492646	0.084772285	-3.120036783	0.001808285	0.024360803	6073
Eif3i	1159.659406	-0.264423649	0.091338631	-2.894981523	0.003791809	0.038316568	1123
Ctsf	1500.044571	-0.264322183	0.085649198	-3.086102275	0.002027991	0.026197759	1980
Shmt2	630.591904	-0.264296915	0.09284616	-2.846611144	0.00441873	0.041976981	2289
Tsc2	2394.843253	-0.264271493	0.07150632	-3.695778105	0.000219214	0.006864501	5432
Stau1	1200.094011	-0.264222207	0.088198928	-2.995753052	0.00273768	0.031295403	2976
Adipor1	4407.841713	-0.263670351	0.068189264	-3.866742847	0.000110299	0.004397252	3123
Ehd1	2023.56431	-0.26363557	0.094348997	-2.794259384	0.005201874	0.046134193	3353
Slc35f6	1418.753225	-0.263609839	0.087508509	-3.012390931	0.002591986	0.030339895	3088
Mipep	669.2980953	-0.263554845	0.061272002	-4.301391136	1.70E-05	0.001374244	3015
Klf16	1185.846119	-0.26348371	0.074866829	-3.519365148	0.000432581	0.01018662	2674
Cmc2	295.021382	-0.263406491	0.090866492	-2.898829752	0.003745582	0.038048641	1459
Sult4a1	19666.01225	-0.26326174	0.07828433	-3.362891901	0.000771306	0.014515707	2383
Stip1	6490.881615	-0.26322431	0.083603632	-3.148479367	0.001641223	0.022906785	2108
Cend1	4823.383678	-0.263087017	0.093332988	-2.8187999	0.004820356	0.044096326	1733
Ddx23	1576.713447	-0.263025898	0.07909542	-3.325425151	0.000882838	0.015829516	3187
Map1a	132907.4635	-0.263019688	0.081701107	-3.219291625	0.001285077	0.019794418	10145
Apc2	20202.14881	-0.262988653	0.085540878	-3.074420795	0.002109117	0.026846363	9173
Tnpo3	1392.85576	-0.262831351	0.072126438	-3.64403623	0.000268396	0.007731042	4251
Tcerg1l	842.8474141	-0.262739716	0.089808986	-2.925539284	0.003438598	0.03590925	2565
Caskin1	10483.92177	-0.26223571	0.083566105	-3.138063081	0.001700683	0.023358487	5938
Rab1b	5594.812578	-0.26222385	0.073664506	-3.559704179	0.000371273	0.009393812	1913
Hey1	1181.569854	-0.262164292	0.091792217	-2.856062312	0.004289309	0.041219561	2423
Ubc	23197.82888	-0.262129384	0.075596698	-3.467471319	0.00052538	0.011510076	2618
Mfap1b	985.8653795	-0.26211435	0.081636092	-3.21076551	0.001323819	0.020173902	3362
Parp1	4045.187437	-0.262100714	0.062481866	-4.194828498	2.73E-05	0.001827379	3873
Agl	646.477751	-0.261848695	0.088868896	-2.946460524	0.003214334	0.034569035	9625
2900026A(	3738.721123	-0.261730501	0.082127825	-3.186867579	0.001438226	0.02116185	10238
Aldh7a1	1703.025324	-0.261719476	0.073734649	-3.549477495	0.000385996	0.009546826	4131
Soga3	9221.830669	-0.261719334	0.070859007	-3.693522462	0.000221169	0.006886468	4105
Mnt	2716.676265	-0.261581644	0.080563506	-3.246899954	0.001166694	0.018621132	4590
Socs5	401.089329	-0.261574752	0.078099021	-3.349270545	0.000810246	0.01499894	978
Braf	573.3059593	-0.261330287	0.065508962	-3.989229552	6.63E-05	0.00315749	2253
Mcu	1391.288963	-0.261221255	0.051887414	-5.034385682	4.79E-07	0.000123977	2872
Fry	2177.728722	-0.261056425	0.0749824	-3.481569359	0.000498485	0.011147446	4379
Urod	927.4403201	-0.260968342	0.063853641	-4.08697668	4.37E-05	0.002468082	1489
Tmem101	398.9950955	-0.260561416	0.093820227	-2.77724136	0.005482245	0.047737554	1574
Wdtc1	6053.005032	-0.260168066	0.060066285	-4.331349353	1.48E-05	0.001252988	4191
Chmp7	1379.480785	-0.260104554	0.074105769	-3.509909648	0.000448259	0.010421844	2649
Mgat5	5204.412522	-0.2598229	0.084591214	-3.071511663	0.002129778	0.026992133	8436
Tmem163	1676.899627	-0.259737596	0.080358763	-3.232224925	0.001228303	0.01921792	2657
Smad1	2252.488994	-0.259680193	0.07940779	-3.270210576	0.001074675	0.017702535	3099
Tsr3	468.7903037	-0.259664067	0.08470648	-3.065456953	0.002173377	0.027371098	1192
Pop5	748.8875753	-0.259485982	0.078219618	-3.317402835	0.000908585	0.016100744	1025
Smarcc1	3257.774668	-0.259411841	0.086010642	-3.016043555	0.002560965	0.030106104	5717
Psmc5	1994.173335	-0.259409967	0.049883307	-5.200336193	1.99E-07	7.17E-05	1316
Zdhhc7	1825.268639	-0.259397036	0.067891305	-3.820769651	0.000133036	0.004960917	3069
Irgq	9093.867416	-0.25938834	0.065039801	-3.988147821	6.66E-05	0.00316506	6083
Coro1a	2000.225091	-0.259283554	0.082078947	-3.158953209	0.001583369	0.022448508	1660
Supt4a	432.3166212	-0.259183585	0.074231551	-3.49155556	0.000480217	0.010860454	706
Acad9	687.9488496	-0.259092869	0.090488601	-2.863265287	0.004192993	0.040649384	3930
Psmc4	2491.307428	-0.258980383	0.054205874	-4.777718085	1.77E-06	0.000307081	1440
Srp19	254.0592999	-0.258901368	0.079955357	-3.23807405	0.001203396	0.0189836	876
Armcx1	1304.688234	-0.258881463	0.090680408	-2.854877585	0.004305342	0.04130141	2413
Dpp3	2273.483426	-0.258805606	0.07684813	-3.367754126	0.000757831	0.0143808	2683

Syt1	5810.15108	-0.258795189	0.059483248	-4.350723914	1.36E-05	0.001200314	4745
Ccdc97	2263.38736	-0.25868014	0.057442506	-4.503287894	6.69E-06	0.000758171	3032
Mafk	463.4523994	-0.25866195	0.068483005	-3.777023957	0.000158713	0.005551184	2849
Med31	178.3032522	-0.258585344	0.093255534	-2.772868616	0.005556455	0.048107432	1161
Rps11	2465.946201	-0.258506472	0.090007335	-2.872060072	0.004078054	0.040047277	661
Snx8	652.975237	-0.258425927	0.085100073	-3.036729772	0.002391598	0.028958548	2627
Rad51d	738.4300268	-0.258398244	0.082003839	-3.151050561	0.001626843	0.022836578	3426
Pex11b	163.9335416	-0.258180136	0.086914394	-2.970510667	0.002973051	0.032883064	1122
Snrpd1	201.3017553	-0.258135884	0.084093953	-3.06961292	0.002143364	0.027101807	858
Vegfb	875.4292454	-0.258126152	0.093132916	-2.771588864	0.005578344	0.048202026	1158
Cacnb1	3001.438882	-0.258095924	0.077873179	-3.314310867	0.000918693	0.01623406	3396
Magi1	1148.955294	-0.257878592	0.08884302	-2.902632	0.003700412	0.037668119	4598
Rnf123	2833.184222	-0.257816058	0.064914406	-3.971630843	7.14E-05	0.003328019	4309
Amigo3	2230.865348	-0.257673543	0.076321397	-3.376163857	0.000735041	0.014074952	2543
Zmat5	513.46545	-0.257268349	0.068611141	-3.749658499	0.000177076	0.005956485	792
Tspyl1	5351.878117	-0.257264898	0.062990811	-4.084165544	4.42E-05	0.002480629	3086
Letm1	3105.208728	-0.257125334	0.064265381	-4.000992895	6.31E-05	0.0030643	5272
Apbb2	1845.252751	-0.257051749	0.08998957	-2.856461578	0.004283919	0.041194034	3362
Usp19	4114.275168	-0.257017101	0.047676182	-5.390891033	7.01E-08	3.28E-05	4512
Arhgdia	15332.4195	-0.25699096	0.076772245	-3.34744619	0.000815598	0.015072625	1867
Usp25	3504.030063	-0.256905409	0.050503716	-5.086861564	3.64E-07	0.000103257	4776
Vars2	894.9469363	-0.256905056	0.064889393	-3.959122477	7.52E-05	0.003459515	4425
Pcyox1l	2883.883072	-0.256843884	0.081604756	-3.147413184	0.00164722	0.022961329	2017
Diablo	2008.263591	-0.256787963	0.083370722	-3.080073656	0.002069494	0.02651087	1576
Cnnm4	2206.701646	-0.256677203	0.089620922	-2.864032155	0.004182855	0.040631859	4547
Scamp3	1837.999426	-0.256406424	0.084395638	-3.038147838	0.002380371	0.028877768	1489
Ccdc91	1452.289653	-0.256378845	0.074497362	-3.441448622	0.000578608	0.012113579	2459
Srp14	1264.88815	-0.256201675	0.083383389	-3.072574499	0.002122209	0.026931568	798
Rangap1	6709.595593	-0.256164498	0.072637245	-3.526627387	0.000420889	0.010084125	3033
Neu1	1278.683355	-0.256073678	0.085411978	-2.9981003	0.002716682	0.031154718	2474
Dync1h1	116579.3304	-0.255900655	0.079799208	-3.206806944	0.00134217	0.020312743	14342
Ece2	1967.074362	-0.25583367	0.08186469	-3.125079581	0.00177757	0.02409089	3152
Spred2	1263.504134	-0.255812474	0.070569872	-3.624953086	0.000289014	0.008106748	2852
Tbx2	3036.781018	-0.255572579	0.089133622	-2.86729714	0.004139941	0.040367532	3626
Cox4i1	4667.009622	-0.255563127	0.063613803	-4.01741625	5.88E-05	0.002916448	745
Ap3s2	6798.299532	-0.255491891	0.08705108	-2.934965194	0.003335851	0.035246501	5813
Tln1	19070.24817	-0.255274369	0.081818266	-3.120016861	0.001808407	0.024360803	8393
Ptprs	18792.24848	-0.255256333	0.058593842	-4.356367936	1.32E-05	0.001184413	5608
Dynll2	15803.35988	-0.25508419	0.062582882	-4.075941893	4.58E-05	0.002544	2476
Paqr7	1326.505924	-0.254971901	0.061118806	-4.171742198	3.02E-05	0.001939388	3550
Pcbp3	6044.272743	-0.254762739	0.072052937	-3.535771744	0.000406586	0.009875992	2013
Amer3	1074.279968	-0.254750674	0.074638583	-3.413123105	0.000642229	0.012975923	4392
Trim62	1622.394745	-0.254718838	0.076951017	-3.310142603	0.000932485	0.016372477	3752
Ndufaf5	496.1671017	-0.254718523	0.089409492	-2.848898013	0.004387094	0.04177574	1166
Slmap	5137.935949	-0.254660724	0.07442085	-3.421900226	0.000621851	0.012727976	5476
Prkar1b	18981.37123	-0.254512401	0.086350246	-2.947442699	0.003204141	0.034502587	2637
Kifbp	3581.161474	-0.254243593	0.074058181	-3.433025067	0.000596887	0.01237053	2472
Frmpd3	2783.673544	-0.254171488	0.08580602	-2.96216382	0.003054852	0.033531953	7314
Phc2	4324.499017	-0.254151285	0.074681273	-3.40314613	0.000666146	0.013312331	2535
Atn1	6222.272894	-0.25395203	0.076863963	-3.303915409	0.000953446	0.016601048	4433
Tbc1d9b	8670.8666	-0.253795358	0.087799728	-2.890616695	0.003844868	0.038623714	5213
Fam117b	3229.415461	-0.253632826	0.079129768	-3.205276991	0.001349326	0.020378942	5532
Tagln3	3325.476412	-0.253589588	0.07803193	-3.249818238	0.001154788	0.018556378	1240
Abhd16a	2753.179971	-0.253575895	0.074624827	-3.398009808	0.00067878	0.013461867	1945
G6pc3	1081.595951	-0.253170062	0.058700832	-4.312887104	1.61E-05	0.001316799	1573
Psap	23915.94472	-0.253012194	0.052593636	-4.810699805	1.50E-06	0.000274776	2657
Pink1	13238.4965	-0.252987066	0.080734916	-3.133552107	0.001727043	0.023587892	2375
Dhdds	2685.8301	-0.252918777	0.064139927	-3.943234589	8.04E-05	0.0035995	3219
Txnl1	936.2148023	-0.252902759	0.070819386	-3.571095065	0.000355492	0.009105381	1998
Cenpb	7665.784584	-0.252768186	0.088270254	-2.863571536	0.004188942	0.040645959	4886
Usp35	3470.048943	-0.252711768	0.080546755	-3.137454351	0.001704218	0.023378683	4262
Flot1	6012.666648	-0.2526997	0.067672706	-3.734145056	0.000188354	0.006202981	1825
Zswim1	848.2209839	-0.252690447	0.079631403	-3.173251225	0.00150742	0.021814922	2705
Mroh1	2983.58379	-0.252667784	0.075943444	-3.327051975	0.0008777	0.015795367	5268
Ndufa11	1292.259183	-0.252644711	0.082098816	-3.077324659	0.002088677	0.026694382	545
Kxd1	574.6753457	-0.252565782	0.089842306	-2.811212156	0.004935523	0.04469509	1216
Ankrd11	7879.661543	-0.252508289	0.069211007	-3.648383396	0.000263896	0.007648102	8455
Pcnx3	3870.751919	-0.252269842	0.081807536	-3.08369932	0.002044441	0.026297207	7264
Rrp7a	2316.690746	-0.252084153	0.080142147	-3.145462954	0.001658242	0.023066188	4363
Bcl7b	1272.329849	-0.252055855	0.088208788	-2.857491424	0.004270042	0.041151334	1758
Mbnl1	1000.171423	-0.252003783	0.086338823	-2.918777137	0.003514074	0.036334959	4319

Gga3	2598.778802	-0.251861188	0.060635156	-4.153715502	3.27E-05	0.002030408	3816
Ccndbp1	883.9655482	-0.251778512	0.04489604	-5.6080339	2.05E-08	1.17E-05	1569
Bag6	2126.553126	-0.251651548	0.081988843	-3.069338942	0.00214533	0.027107452	1722
Puf60	2210.312766	-0.251590722	0.073697545	-3.413827729	0.000640571	0.012966247	1881
Aldoa	49136.81735	-0.251588528	0.064298906	-3.912796383	9.12E-05	0.003901828	1511
Fbxw5	2087.150851	-0.251313535	0.082732189	-3.037675382	0.002384106	0.028899154	2388
Mrpl16	258.6875794	-0.251269787	0.076510483	-3.284122338	0.001023005	0.017232344	1162
Schip1	908.4659421	-0.25121981	0.082243654	-3.054579874	0.00225376	0.027937492	1730
B3gat3	4072.198434	-0.251129951	0.068639317	-3.658689529	0.000253508	0.007485218	1603
Ppil2	1155.095116	-0.251079271	0.075186132	-3.339435926	0.000839487	0.015366665	2333
Coq7	380.6409479	-0.250961633	0.069693265	-3.600945253	0.000317062	0.00856058	914
Tada3	1092.8702	-0.250957097	0.085027	-2.951498906	0.003162357	0.034194051	2848
Eif3c	5158.387473	-0.250918915	0.054237338	-4.62631319	3.72E-06	0.000503544	3383
Wasf1	1346.583045	-0.250898514	0.070859107	-3.540808313	0.000398903	0.009775798	2719
Rgl1	1717.812571	-0.250837433	0.087472685	-2.867608702	0.004135867	0.040367532	4572
Skiv2l	3345.256957	-0.250833559	0.063508668	-3.949595637	7.83E-05	0.003544518	3951
Atg101	550.5819658	-0.250826754	0.08443183	-2.97076062	0.002970632	0.032864582	1270
Naa35	906.2334012	-0.250812313	0.084338524	-2.973876019	0.002940639	0.032647772	3793
Usp20	5181.145187	-0.250790523	0.089083111	-2.815242072	0.00487405	0.044411944	4970
Dipk1b	1597.783714	-0.250654989	0.089232045	-2.80902437	0.004969188	0.044860446	1622
Trmt10a	251.3977607	-0.250604346	0.086633873	-2.892683174	0.003819664	0.038516104	4025
Clstn2	1263.618127	-0.250584926	0.079400184	-3.155974135	0.00159963	0.022553573	4174
Pigs	3129.907376	-0.25026533	0.074467784	-3.360719434	0.000777398	0.014606103	2501
Mtmr3	2864.44816	-0.250197773	0.076774593	-3.258861622	0.001118602	0.018167471	5674
Sugp1	1352.207374	-0.250117526	0.071248505	-3.51049505	0.000447273	0.010411214	2681
Gabbr2	15132.1311	-0.250111519	0.085507274	-2.925032084	0.003444207	0.035942218	5750
Bub3	1752.979697	-0.250075954	0.051706985	-4.836405682	1.32E-06	0.00025054	2218
Higd2a	1133.800415	-0.249978134	0.07539053	-3.315776301	0.00091389	0.016162169	648
Lrrfip2	605.2256329	-0.249840566	0.078827118	-3.16947481	0.001527147	0.021970228	1839
Psmb1	2343.780728	-0.249756566	0.064008037	-3.901956329	9.54E-05	0.00401442	1758
Atg3	1042.265428	-0.249749803	0.058167132	-4.293658567	1.76E-05	0.001396003	2057
Stmn3	16571.3878	-0.249741893	0.073665317	-3.390223539	0.000698357	0.013659644	1136
Med8	417.9300877	-0.249720444	0.079930831	-3.124206786	0.001782851	0.024117862	1145
Nfic	17537.23615	-0.249661516	0.056597917	-4.411143168	1.03E-05	0.001009098	6237
Klhl17	1214.488737	-0.249649126	0.065247922	-3.826162107	0.000130157	0.004906744	2723
Get3	1655.893446	-0.249642612	0.061945142	-4.030059586	5.58E-05	0.002830956	1256
Coq2	1016.156124	-0.249553484	0.071446583	-3.492868011	0.000477863	0.010827162	1743
Btf3	2010.457448	-0.249367146	0.065632296	-3.799457888	0.000145013	0.005243005	888
Ssbp3	4041.569313	-0.249353183	0.045240347	-5.511743374	3.55E-08	1.93E-05	3195
Slc6a17	16562.74764	-0.249235927	0.084124475	-2.962704098	0.003049496	0.033493259	6322
H13	2991.830183	-0.249123486	0.083206203	-2.994049442	0.002753013	0.031365611	1743
Dnajc11	1419.929682	-0.248986824	0.068596388	-3.629736678	0.00028371	0.008000854	3151
Rbm43	324.004429	-0.248694819	0.079261116	-3.137664849	0.001702995	0.023378683	2057
Gak	3549.681329	-0.248621782	0.052320675	-4.751884066	2.02E-06	0.000331845	4453
Myo1e	9382.514343	-0.248323041	0.081504408	-3.046743688	0.002313349	0.02834626	4625
Hdhd2	678.9121868	-0.248119051	0.0721892	-3.437066069	0.000588052	0.012244191	1538
Ift172	2272.154283	-0.24810769	0.063258922	-3.922097947	8.78E-05	0.003791065	5403
Lztr1	3838.437023	-0.247972218	0.08583063	-2.88908771	0.003863613	0.038741876	3394
Grpel1	854.3827095	-0.247897733	0.072784091	-3.405932944	0.000659384	0.013219246	3528
Arl6ip5	3032.975822	-0.24782991	0.059499826	-4.165220778	3.11E-05	0.00196768	1442
Ap2a2	12615.68732	-0.247793012	0.062916345	-3.938452138	8.20E-05	0.0036358	4646
Nek9	4609.808463	-0.247772276	0.075612128	-3.276885361	0.00104959	0.017479247	5386
Coro2b	8917.277856	-0.247716724	0.077917393	-3.179222442	0.001476707	0.021533477	3610
Cog1	3103.06835	-0.247602383	0.054250728	-4.564037949	5.02E-06	0.000612811	3935
Eif4g3	3761.437769	-0.247541036	0.071558081	-3.459302337	0.000541577	0.011711961	5289
Pcdh1	4303.758121	-0.247518552	0.07978329	-3.102385882	0.001919675	0.025314497	3889
Rab11fip3	6855.164458	-0.247517368	0.070838781	-3.494094096	0.000475673	0.010796581	5383
Hsdl2	1885.391088	-0.247416162	0.054031387	-4.579119242	4.67E-06	0.000585149	2611
Rcc2	4009.826248	-0.247361553	0.072920183	-3.392223425	0.000693279	0.013596341	3767
Dbndd1	693.6539219	-0.247345184	0.07246201	-3.41344638	0.000641468	0.012972462	1677
Cfdp1	760.1036885	-0.247205916	0.061082955	-4.047052316	5.19E-05	0.002740802	1178
Ppfia3	4698.467743	-0.247161183	0.074339437	-3.324765328	0.00088493	0.01585246	4647
Vps51	1870.895914	-0.246765714	0.072355073	-3.410482568	0.00064848	0.013072185	2675
Ubl5	611.4025193	-0.246379135	0.078685023	-3.131207493	0.001740891	0.023726258	489
Eral1	610.402849	-0.246309101	0.065276563	-3.77331603	0.000161092	0.005589906	2014
Taf6	974.1377229	-0.24629359	0.086931846	-2.833180264	0.004608739	0.043065505	2304
Elp2	910.238391	-0.246129939	0.064978986	-3.787839003	0.000151963	0.00539666	2497
Tom1	2351.855727	-0.24597411	0.055973667	-4.39446125	1.11E-05	0.001063664	2267
Kif1a	8805.47656	-0.245844244	0.066741441	-3.683532147	0.000230024	0.007067098	5931
Cluh	6007.51886	-0.245739234	0.059871121	-4.104470267	4.05E-05	0.002356677	5379
Nalf2	1245.970905	-0.245380525	0.084470237	-2.904934727	0.003673297	0.037470186	3909

Zdhhc18	3342.574048	-0.245099574	0.06334155	-3.869491267	0.000109063	0.004386954	4580
Kcnq4	6983.435062	-0.245030634	0.083931368	-2.919416644	0.003506872	0.036286094	3919
Dnaja2	2317.868164	-0.245025371	0.074997963	-3.267093659	0.001086577	0.017832269	2919
Dpp9	3742.838533	-0.244892465	0.078268949	-3.128858466	0.001754868	0.023856765	3374
Adgrl1	11596.02251	-0.244757708	0.069892909	-3.501896135	0.00046196	0.010626266	8181
Rhot2	2387.309616	-0.244698067	0.06016303	-4.067249707	4.76E-05	0.0025981	3189
Prcc	2426.338733	-0.24460162	0.078413228	-3.119392325	0.001812245	0.02439539	2099
Med4	268.7738474	-0.24456644	0.070257577	-3.480997363	0.00049955	0.011165599	1328
Npr2	4445.208297	-0.244452423	0.069624788	-3.510997009	0.000446429	0.010406824	3660
Grina	15328.43757	-0.244424657	0.080914952	-3.020760071	0.002521411	0.02983927	1725
Pcbp4	10546.57434	-0.244404037	0.073552677	-3.322843535	0.000891049	0.015915763	2035
Mprip	9533.783237	-0.244261942	0.075293193	-3.244143756	0.001178043	0.018731519	7765
Vapa	3481.761048	-0.24417932	0.065085447	-3.751673058	0.000175658	0.005918204	3369
Me2	950.0455875	-0.243947195	0.055802847	-4.371590457	1.23E-05	0.001132119	2638
Lrrc8a	8222.701372	-0.243942276	0.060280767	-4.04676796	5.19E-05	0.002740802	4301
Miga2	2290.338043	-0.243916995	0.072196612	-3.378510289	0.000728797	0.014022566	3381
Vps25	1402.40856	-0.243890286	0.070568419	-3.456082602	0.000548087	0.011763883	1106
Grb14	1473.631597	-0.243605422	0.059880842	-4.068169607	4.74E-05	0.00259186	1986
Vapb	7506.917387	-0.243357818	0.050419759	-4.826635872	1.39E-06	0.000259784	7032
Mief2	498.0327953	-0.243279238	0.074833865	-3.250924389	0.001150304	0.018504623	2522
Mfhas1	6007.738551	-0.243218074	0.082172789	-2.959837177	0.003078017	0.033696466	6408
Dele1	1435.049738	-0.243149686	0.074333682	-3.271056689	0.001071464	0.017669515	2394
Tubb5	33389.04369	-0.24310507	0.073872261	-3.290884407	0.000998729	0.017005863	2649
Faah	1419.495003	-0.243070179	0.079524973	-3.056526405	0.002239178	0.027888227	3827
Dynlrb1	2444.693228	-0.24297618	0.072638378	-3.345011089	0.000822793	0.015172579	684
Acat2	1881.101476	-0.242826286	0.086322013	-2.813028524	0.00490773	0.044567706	2250
Uba1	16686.50886	-0.242694934	0.062678531	-3.872058434	0.00010792	0.004348951	4071
Polr2a	5265.185113	-0.242658661	0.081966022	-2.960478696	0.003071614	0.033659894	6740
Cdc42bpb	7266.087794	-0.242611049	0.074721787	-3.246858231	0.001166865	0.018621132	6714
Abcb9	2667.250045	-0.242565745	0.084202775	-2.880733388	0.003967511	0.039312963	3661
Calm3	55612.58381	-0.242442565	0.074735391	-3.244012809	0.001178584	0.018733357	2290
Ctbp1	3496.075661	-0.242218391	0.064983574	-3.727378721	0.000193482	0.006315085	2279
Atl3	794.3178194	-0.242198529	0.082467994	-2.936879129	0.003315333	0.035105639	4368
Rnf216	562.0232574	-0.242164506	0.076701931	-3.157215262	0.001592837	0.022503224	3704
Hsp90ab1	51993.78897	-0.242033743	0.076025301	-3.183594683	0.001454585	0.021316948	2520
Rida	266.8680128	-0.241707965	0.085066908	-2.84138653	0.004491783	0.042387048	1004
Syt12	875.3528531	-0.241693514	0.087553977	-2.760508694	0.005771142	0.049210292	3581
Gnl1	2824.237264	-0.241684719	0.080713362	-2.994358241	0.002750228	0.031365611	2848
Dcaf6	1805.330501	-0.241631902	0.079587225	-3.036063899	0.002396886	0.028991858	4110
Vamp2	12654.36134	-0.241630069	0.055210884	-4.37649341	1.21E-05	0.001121004	2205
Ndufb8	3066.413146	-0.24160375	0.069740562	-3.464321819	0.00053157	0.011592501	680
Actr1b	8190.649577	-0.241461206	0.055885335	-4.320654189	1.56E-05	0.001292914	3248
Cyc1	2408.12255	-0.2414553	0.070571405	-3.421432502	0.000622922	0.012743955	1520
Ift140	1309.77946	-0.241424775	0.05857243	-4.121815943	3.76E-05	0.00223631	5489
Vps18	3046.663503	-0.241357496	0.059075491	-4.085577466	4.40E-05	0.002475062	4054
Lrrc41	2532.327997	-0.241215741	0.082022224	-2.940858343	0.003273042	0.034872626	3065
Katnb1	1618.694161	-0.241152837	0.066295977	-3.637518391	0.000275278	0.007858816	3777
Akirin1	1812.2715	-0.241121828	0.061192853	-3.940359297	8.14E-05	0.003620423	3052
Plcd4	2423.189432	-0.241057598	0.085450178	-2.821030975	0.004786958	0.043992184	2720
Brap	5431.187419	-0.241016641	0.074178113	-3.249161133	0.001157459	0.018573122	3744
Stk24	6192.522474	-0.240997764	0.073668956	-3.271361184	0.001070311	0.017657123	2676
Tpd52l2	570.5780209	-0.240983382	0.082073867	-2.936176769	0.003322849	0.035159822	985
B4galt5	3710.905462	-0.240944915	0.070091186	-3.437592197	0.000586911	0.012240767	4210
Clybl	330.1270501	-0.240929023	0.085222445	-2.827060673	0.004697743	0.043539403	1231
Tubb2a	15275.65009	-0.240826251	0.083193049	-2.894788143	0.003794145	0.038329409	1610
Rnf181	1570.04145	-0.240569677	0.077014614	-3.123688682	0.001785993	0.024145507	1434
Kdm4b	2052.649794	-0.240492878	0.06971041	-3.449884721	0.000560826	0.011899959	4577
Dkk3	3873.902684	-0.240371829	0.0662683	-3.627252054	0.000286454	0.008062698	3359
Pacs1	1870.391926	-0.240344603	0.084717152	-2.837024099	0.004553617	0.04274256	4869
Zswim5	1775.474979	-0.240330915	0.07330985	-3.278289563	0.001044382	0.017452017	5582
Sord	1359.639615	-0.240205894	0.061938082	-3.878161678	0.000105249	0.004292451	2352
Ttc1	816.2019744	-0.240137523	0.082925316	-2.895828839	0.003781586	0.03826408	1438
Ube2e3	1430.340931	-0.240025096	0.079907563	-3.003784458	0.002666442	0.030890673	2249
Emc10	4495.035609	-0.239918512	0.065616207	-3.656391017	0.000255791	0.007522311	1855
Cog4	1984.766676	-0.239869025	0.064919838	-3.694849401	0.000220017	0.006875681	2737
Mef2d	1594.607718	-0.239798572	0.06562606	-3.654014449	0.000258172	0.007561972	3488
Vps11	2327.548274	-0.239635651	0.069203849	-3.46275033	0.000534684	0.011643088	3536
Oaz1	2615.476129	-0.23955692	0.083157656	-2.880756041	0.003967226	0.039312963	1044
Pi4k2a	5263.881235	-0.239462732	0.077859624	-3.075570091	0.002101005	0.026781879	3604
Eif4g1	10843.67864	-0.23943201	0.079536675	-3.01033468	0.0026096	0.03048115	5444
Nsun2	1908.082321	-0.239420643	0.063883812	-3.747751341	0.000178427	0.005983633	2834

Sugp2	1968.140562	-0.239358455	0.082308093	-2.908079234	0.003636562	0.037216351	3874
Ttll1	1651.561335	-0.239279595	0.079612771	-3.005542857	0.002651072	0.030831695	2060
Nckap1	4809.841485	-0.239261205	0.072910832	-3.281559096	0.001032349	0.017342822	4597
Uvrag	1866.631837	-0.239190254	0.072733252	-3.288595628	0.001006886	0.017072014	5157
Ndufs6	407.3506725	-0.239087456	0.070034057	-3.413874151	0.000640462	0.012966247	568
Gapdh	48220.34873	-0.239001815	0.071204769	-3.356542233	0.000789237	0.014721591	1420
Vac14	1481.859636	-0.239001583	0.064674691	-3.695442223	0.000219504	0.006868691	3069
Reep1	9966.838801	-0.238909685	0.075704692	-3.155810794	0.001600526	0.022553573	3924
Camk2a	3116.951138	-0.238819712	0.068712542	-3.475634938	0.000509646	0.011276581	4970
Ube3b	9048.547311	-0.238778882	0.081560461	-2.927630365	0.003415558	0.035728057	5194
Syn2	3809.942283	-0.238564399	0.075344024	-3.16633471	0.001543731	0.022114732	3806
Tbc1d13	3293.675815	-0.238508935	0.06500943	-3.668835978	0.000243657	0.007307887	3595
Abhd12	11156.34402	-0.238500098	0.072412607	-3.293626753	0.000989037	0.016912885	1999
Optn	972.8982443	-0.238462966	0.071155018	-3.351316217	0.000804284	0.014913685	2614
Tmsb4x	8566.018506	-0.238278675	0.070242345	-3.392236903	0.000693245	0.013596341	768
Jak1	11956.5606	-0.238241168	0.064953382	-3.667879354	0.000244571	0.007329614	4906
Bap1	4227.120705	-0.238176417	0.067610325	-3.522781747	0.000427043	0.010137992	3460
Parl	414.7323338	-0.238089578	0.060427163	-3.940108456	8.14E-05	0.003620546	1348
Noc4l	502.1777918	-0.238052065	0.065886509	-3.61306236	0.000302602	0.00835147	2075
Mrps18a	939.7329681	-0.237740477	0.075723332	-3.139593438	0.001691825	0.023309641	884
Grsf1	3153.403575	-0.237380266	0.085298637	-2.782931514	0.005387018	0.047261451	2538
Spryd3	3685.598619	-0.237309003	0.067312217	-3.525496763	0.000422689	0.010094501	2456
Foxk1	3635.562883	-0.237308954	0.062353107	-3.805888224	0.000141296	0.005164687	7439
Copg1	10582.0709	-0.237152791	0.065309637	-3.631206663	0.000282099	0.007975878	4140
Pank1	859.0892344	-0.237077067	0.084101841	-2.818928375	0.004818427	0.044090688	7414
Mark4	3781.407086	-0.237072983	0.074192936	-3.195357906	0.001396575	0.020824388	3966
Larp1	13275.39456	-0.236983056	0.055627077	-4.260210486	2.04E-05	0.001529671	6617
Ppm1h	5494.343458	-0.236873584	0.078819855	-3.005252712	0.002653603	0.030831695	6369
Tmem179	2235.920859	-0.236838993	0.078999003	-2.997999789	0.002717579	0.031154718	2380
Dst	104100.3972	-0.236717815	0.076896782	-3.078383881	0.002081266	0.026635988	17212
Mapk8ip2	23441.29973	-0.236667598	0.071167352	-3.32550798	0.000882576	0.015829516	5558
Tmem65	4243.753713	-0.236654308	0.059974526	-3.945913794	7.95E-05	0.003580995	3747
Napa	8584.698746	-0.236533587	0.066178124	-3.574195995	0.000351306	0.009048129	2517
Psmd2	7677.79385	-0.236448261	0.066540828	-3.553431319	0.00038024	0.009466978	2961
Zmat2	1260.837948	-0.236344608	0.043396721	-5.446139752	5.15E-08	2.57E-05	2310
Nomo1	5479.670315	-0.236322847	0.065841505	-3.589268603	0.000331607	0.008735233	4257
St3gal2	5111.982023	-0.236266651	0.066564924	-3.549416653	0.000386086	0.009546826	4396
Chmp5	1137.834828	-0.236192616	0.084285794	-2.802282635	0.00507424	0.045472676	1519
Osbpl10	1280.247547	-0.236115741	0.081742515	-2.888530403	0.003870466	0.0387708	2477
Nmt1	5171.464062	-0.23608918	0.050340944	-4.689804423	2.73E-06	0.000408944	4861
Amdhd2	435.6317775	-0.23598553	0.079754484	-2.958899839	0.003087394	0.033752862	1496
Msto1	662.1120385	-0.235950884	0.062208695	-3.792892374	0.000148903	0.005344088	1875
Ndel1	3264.448354	-0.235879273	0.068930021	-3.422010773	0.000621599	0.012727976	2382
Nme1	1282.76229	-0.23580958	0.065647454	-3.59205979	0.000328075	0.008678579	3181
Mrpl11	329.8589554	-0.235802501	0.083673991	-2.818109887	0.004830727	0.044153946	3045
Gabarapl1	13166.02428	-0.235799784	0.066881836	-3.525617668	0.000422496	0.010094501	1820
Cog7	1801.485885	-0.23559526	0.068009503	-3.464152044	0.000531906	0.011594069	2902
Tusc2	3335.542599	-0.235550969	0.079987843	-2.944834617	0.003231273	0.034700276	1665
Psmd11	2883.685027	-0.235526766	0.056832063	-4.14425857	3.41E-05	0.002096269	2848
Aff3	3754.425434	-0.235483802	0.076643095	-3.072472514	0.002122934	0.026931568	5857
Ralbp1	3789.030248	-0.23547856	0.052445078	-4.490003028	7.12E-06	0.00079273	3811
Ubac1	1365.744337	-0.235470698	0.054819121	-4.295411825	1.74E-05	0.001393845	1852
Hectd3	2881.224595	-0.235340513	0.043262176	-5.439867739	5.33E-08	2.63E-05	4583
Sap30bp	984.7297385	-0.235301114	0.073858507	-3.185836323	0.001443362	0.021216123	3654
Prrc2b	23529.97414	-0.235272298	0.06903067	-3.408228523	0.000653861	0.013138484	10671
Sbds	900.3100151	-0.235223721	0.076707487	-3.066502773	0.002165788	0.027291973	1548
Fam102a	5603.445368	-0.234912013	0.079611445	-2.950731672	0.003170222	0.034253824	4223
Rab10	4492.045022	-0.234768118	0.049671817	-4.726384745	2.29E-06	0.00036409	3513
Sdf2	760.7005293	-0.234747867	0.077188729	-3.041219482	0.00235622	0.028655938	1268
Dpysl2	37109.8034	-0.234708147	0.06787355	-3.458020818	0.000544159	0.011733185	4520
Eif3d	1909.245969	-0.234594402	0.07068614	-3.31881756	0.000903995	0.016032322	1899
Cap1	3912.982827	-0.234555558	0.077212599	-3.037788664	0.00238321	0.028899154	2620
Mrpl18	465.1481507	-0.234447331	0.074371299	-3.152389909	0.001619399	0.022761146	1219
Nacc1	8053.921177	-0.234358572	0.06646729	-3.525923358	0.000422009	0.010094501	4344
Ift88	475.5775946	-0.234271913	0.064764083	-3.617312284	0.000297678	0.008277936	3088
Sharpin	1042.285896	-0.234100502	0.071425172	-3.277563034	0.001047073	0.017473364	1734
Abcf2	3625.893502	-0.234085712	0.070465093	-3.322009525	0.000893716	0.015933594	2569
Chn2	412.1450429	-0.233978533	0.075816812	-3.086103536	0.002027982	0.026197759	2418
Sars	2402.190788	-0.233938344	0.066150719	-3.536444447	0.000405552	0.00986723	1866
Prkar2a	6731.93373	-0.233868067	0.055918053	-4.182335644	2.89E-05	0.001887679	4965
Cnst	9207.914306	-0.233678089	0.078221929	-2.987373124	0.002813861	0.031794239	4709

Smc1a	2056.849563	-0.23353933	0.051229461	-4.558691933	5.15E-06	0.000623421	4691
Slc39a6	2223.69427	-0.233503731	0.072720752	-3.210964209	0.001322904	0.020166948	3882
AU040320	3343.987472	-0.233412918	0.0749569	-3.113961724	0.001845934	0.024697658	4384
Tmem9	1450.269168	-0.233408053	0.050912489	-4.58449502	4.55E-06	0.000576594	1806
Deaf1	1216.86616	-0.233364603	0.064113603	-3.639860978	0.000272785	0.007824201	2135
Pknox2	1326.617403	-0.233280992	0.062527179	-3.730873416	0.000190817	0.006265327	3632
Rps6ka4	1625.466854	-0.232995891	0.054449368	-4.279129362	1.88E-05	0.001454844	3140
Exoc8	1913.242478	-0.232709625	0.076751614	-3.03198348	0.002429525	0.029203319	4598
Prpf19	5202.734188	-0.232553306	0.073558572	-3.1614712	0.001569743	0.022349018	2134
Snx3	1938.640777	-0.232468666	0.072931334	-3.187500548	0.001435082	0.021136809	1401
Ppp2r1a	14144.07661	-0.232358165	0.066415271	-3.498565352	0.000467768	0.010688898	2410
Mcoln1	1995.358514	-0.232208043	0.062361859	-3.723558735	0.000196434	0.006356524	2065
Slc27a4	6619.302233	-0.232083089	0.072844143	-3.186022667	0.001442433	0.021209927	4054
Nop16	307.5659257	-0.231860843	0.083485431	-2.777261137	0.005481912	0.047737554	1756
Psmd9	470.4775319	-0.231709265	0.078067587	-2.968059761	0.002996861	0.033079833	2597
Mlf2	7853.528796	-0.23167228	0.077270723	-2.998189642	0.002715886	0.031154718	1475
Iars2	2241.26491	-0.231589515	0.044170889	-5.243034982	1.58E-07	6.09E-05	5471
Elac2	997.6560491	-0.231587951	0.061624091	-3.758074924	0.000171226	0.005822068	2884
Ripor1	2253.509815	-0.231513663	0.047645414	-4.859096457	1.18E-06	0.000232487	4108
Dhx37	1056.026447	-0.231388862	0.0665587	-3.476463044	0.000508074	0.011276581	4760
Ptcd1	881.4503423	-0.231259628	0.058780166	-3.934313973	8.34E-05	0.003675556	3087
Drosha	3467.580119	-0.230994335	0.058564404	-3.944278731	8.00E-05	0.00359814	4519
Dctn3	1912.752104	-0.230967799	0.071862915	-3.214005441	0.001308972	0.020010066	973
Grm7	1526.764302	-0.230966047	0.080009702	-2.886725488	0.003892737	0.038901349	4197
Ndufv3	677.6587507	-0.230960478	0.078520685	-2.941396645	0.003267359	0.034839677	1582
Rtraf	980.5018523	-0.230863484	0.081637599	-2.827906327	0.004685351	0.043458117	1562
Jagn1	658.247303	-0.230778006	0.058926497	-3.916370716	8.99E-05	0.003863247	1201
Gfod2	644.233012	-0.230761727	0.0623766	-3.699491895	0.000216032	0.006797049	4352
Chpf2	3103.824846	-0.230732761	0.069093751	-3.339415782	0.000839548	0.015366665	3715
Pgm1	2696.716856	-0.230339891	0.060079066	-3.833945917	0.000126104	0.004825202	2371
Cops6	651.6226995	-0.230167245	0.073448903	-3.133705674	0.001726139	0.023587892	1787
Cpt1a	6368.868193	-0.229995079	0.06501013	-3.537834471	0.000403423	0.009853604	4317
Fkrp	1524.801406	-0.229717518	0.075171698	-3.055904344	0.002243828	0.02789873	2827
Nckipsd	2582.593398	-0.229628944	0.080128838	-2.865746569	0.004160271	0.040466004	3360
Atmin	4464.322321	-0.229575046	0.075635156	-3.035295453	0.002403002	0.029056096	4877
Ubl3	3820.23699	-0.229522328	0.064596135	-3.553189804	0.00038059	0.009467961	2537
Ywhae	16864.52282	-0.229375469	0.057881659	-3.962835096	7.41E-05	0.003420447	2100
Ddx56	917.3927263	-0.229369707	0.058478591	-3.922285112	8.77E-05	0.003791065	1956
Gpi1	16135.27922	-0.229353911	0.063514452	-3.611050782	0.000304959	0.008388017	2884
Lrrc75b	4841.127981	-0.22933406	0.073484212	-3.120861683	0.001803227	0.024333603	4601
Prkag2	1278.917641	-0.229143798	0.076984274	-2.976501381	0.002915578	0.032444264	3331
Cc2d1b	2683.291182	-0.229046854	0.066202124	-3.459811242	0.000540554	0.011707125	3303
Tecr	4918.222759	-0.228940785	0.067280889	-3.402761007	0.000667086	0.013319	1158
Tomm6	891.1605669	-0.228931838	0.0724737	-3.158826404	0.001584058	0.022451037	749
Ncdn	29807.02489	-0.228911819	0.082465321	-2.775855542	0.005505666	0.047866392	3681
Gatc	531.5525281	-0.228824467	0.072882661	-3.139628345	0.001691623	0.023309641	1797
Cadm3	33739.34528	-0.228817518	0.079640297	-2.873137441	0.004064173	0.039965762	5155
Eif3l	2967.560889	-0.228792441	0.060837214	-3.760731744	0.000169417	0.005779923	1942
Ap1m1	3937.07263	-0.228782365	0.062849282	-3.64017468	0.000272453	0.007823908	2084
Ctnnbl1	1136.74106	-0.228739149	0.076263643	-2.999321025	0.00270582	0.031128399	2442
Ddx24	5188.939336	-0.228735175	0.055621153	-4.112377438	3.92E-05	0.002298656	2850
Ctsa	3197.052261	-0.228555974	0.049671192	-4.601378846	4.20E-06	0.000555791	2534
Dlc1	2370.698053	-0.228367663	0.072772045	-3.138123466	0.001700332	0.023358487	6159
Dcaf5	3444.605863	-0.228337318	0.068379404	-3.339270393	0.000839988	0.015368312	5706
Triap1	373.1460363	-0.228287786	0.065178166	-3.502519333	0.00046088	0.010608693	1088
Polrmt	1347.787964	-0.228122035	0.078410675	-2.909323704	0.003622116	0.037129012	3755
Clip2	10678.74271	-0.228073728	0.07383189	-3.089095065	0.002007672	0.026029871	4994
Ski	7011.235144	-0.227922177	0.07116145	-3.202888314	0.001360567	0.02049241	5522
Raf1	2556.026109	-0.227780682	0.076994991	-2.958383132	0.003092575	0.033772144	3070
Kif2a	1299.075344	-0.227775959	0.080775957	-2.819848479	0.004804633	0.044053325	2570
Dmap1	458.1888165	-0.227774769	0.077179827	-2.95122154	0.003165198	0.034216359	1566
Gspt1	1844.254055	-0.227770726	0.078869364	-2.88794933	0.003877623	0.038801423	3716
Tbc1d8	1588.33928	-0.22775592	0.074220223	-3.068650436	0.00215028	0.027150223	4451
Taco1	359.8314154	-0.227710008	0.081962081	-2.778236037	0.00546549	0.047648278	1383
Vps4a	2324.68801	-0.22770444	0.054288606	-4.194332035	2.74E-05	0.001828602	2178
Apmap	884.0139634	-0.227628587	0.06699226	-3.397834099	0.000679216	0.013463359	2235
Gtf2ird2	603.2038113	-0.227494896	0.056277726	-4.042361174	5.29E-05	0.002769512	3482
Ulk1	5205.437298	-0.227190593	0.064494749	-3.522621545	0.000427301	0.010137992	5213
Tmem30a	12554.38704	-0.227168861	0.066365049	-3.423019579	0.000619296	0.012693406	3658
Poldip2	1432.110095	-0.227161617	0.068748576	-3.304237422	0.000952351	0.016601048	2054
Chst11	3173.643257	-0.227075024	0.069104296	-3.285975515	0.001016298	0.017170876	5527

Plekha3	1132.957553	-0.227019133	0.067798423	-3.348442657	0.000812671	0.015037491	2534
Lnpk	1136.993362	-0.226905007	0.047553109	-4.771612498	1.83E-06	0.000307851	3195
Hdac2	1595.1775	-0.226885507	0.060458921	-3.752721714	0.000174925	0.005902209	2005
Rtf2	2534.159758	-0.22681696	0.060392416	-3.75571927	0.000172844	0.005849963	2173
Hdgf	4656.976393	-0.226348993	0.06732699	-3.361935405	0.000773982	0.014548153	2245
Gtf3c1	8663.707657	-0.226325152	0.067666827	-3.344698738	0.00082372	0.015172579	6961
Smg8	702.2087593	-0.226135177	0.074510832	-3.034930237	0.002405913	0.029059838	3218
Ttc28	3566.087804	-0.226068097	0.080255758	-2.816845849	0.00484978	0.044264182	10688
Spats2	992.8176542	-0.226046277	0.07840416	-2.883090353	0.003937945	0.039099309	3096
Myt1l	1145.400975	-0.226013057	0.077795576	-2.905217346	0.003669982	0.037469578	5027
Actb	56272.46098	-0.225992697	0.057561826	-3.926086315	8.63E-05	0.00374813	1920
Timm22	419.0304528	-0.225984976	0.078704869	-2.871296009	0.004087925	0.040090483	2817
Adgra3	1488.570675	-0.225960957	0.0686944	-3.28936503	0.001004137	0.017051713	4480
Polr3h	595.1569371	-0.225949376	0.071427638	-3.163332591	0.00155974	0.02226428	2542
Rundc3a	5329.472779	-0.225853422	0.074105961	-3.047709241	0.002305929	0.028286601	2046
Vps26b	3454.925682	-0.225814447	0.058962684	-3.829785745	0.000128255	0.004868039	3575
Fam20a	435.2666308	-0.225757931	0.076696117	-2.943537929	0.003244841	0.034744135	2541
Xylt2	1798.429256	-0.225717452	0.063810854	-3.537289283	0.000404257	0.009860072	3442
Mrpl36	482.1577546	-0.225541877	0.063347393	-3.560397154	0.000370294	0.009393812	923
Ddx47	936.962337	-0.225471617	0.059132224	-3.813007572	0.000137286	0.005064499	1764
Atp13a1	1976.789924	-0.225462361	0.0798045	-2.825183569	0.004725354	0.043736878	3915
Akirin2	865.3365135	-0.225423862	0.071441539	-3.155361222	0.001602995	0.022581121	1397
Dnttip2	512.1626993	-0.225315805	0.08128048	-2.772077699	0.005569974	0.048173052	2429
Itprid2	4114.315774	-0.225261556	0.055534254	-4.056263263	4.99E-05	0.002676768	5168
Timmdc1	796.5386367	-0.225253136	0.073849327	-3.050171813	0.002287105	0.028134462	1573
Wwp2	2573.000824	-0.225148164	0.045130682	-4.98880477	6.08E-07	0.000144381	4314
Cbx6	13327.10617	-0.225043619	0.070684394	-3.183780826	0.00145365	0.021313534	5806
Foxj2	2423.679499	-0.224811808	0.07809699	-2.878623224	0.003994152	0.039514603	5283
Pik3ip1	1720.801422	-0.224736579	0.06991751	-3.214310405	0.001307582	0.020007928	2573
Akt1	8174.240497	-0.224652726	0.047059443	-4.773807551	1.81E-06	0.000307825	2690
Rheb	1703.541947	-0.224575863	0.072955994	-3.07823731	0.00208229	0.026635988	1784
Atg7	729.2756246	-0.224340241	0.072933978	-3.075935911	0.002098429	0.02677233	3761
Uqcrc1	7692.9612	-0.224015707	0.062515283	-3.58337505	0.000339183	0.008860477	1653
Wdr1	7411.7103	-0.223800581	0.057651578	-3.881950682	0.000103622	0.004250444	3089
Plekha6	1086.147374	-0.223568833	0.076907904	-2.906968243	0.003649502	0.037305365	5040
Phospho1	1002.30499	-0.223500283	0.076295206	-2.92941449	0.003396012	0.035601293	590
Gpatch1	624.0493367	-0.223417891	0.067078845	-3.330675896	0.000866354	0.015672695	3065
Lysmd1	668.3773538	-0.223259516	0.075561276	-2.954681648	0.003129918	0.034027414	2394
Ptpa	5694.855288	-0.223108455	0.063300703	-3.524581017	0.000424153	0.010115454	2586
Ankrd27	2569.907114	-0.222915959	0.063623963	-3.503647796	0.000458932	0.010580462	4284
Taf5l	652.4421996	-0.222647463	0.078610374	-2.83229111	0.004621575	0.043121297	2973
Eaf1	2304.150277	-0.222578325	0.073700308	-3.020046047	0.002527363	0.029842862	4918
Tsnax	1979.575932	-0.222544072	0.058668501	-3.793246241	0.000148691	0.005340834	2391
B4galt6	6452.478726	-0.222477434	0.069131598	-3.218172901	0.0012901	0.019816562	5808
Ap1s1	1752.788736	-0.222409742	0.045971247	-4.838018413	1.31E-06	0.000249592	1353
Nus1	1885.361866	-0.22229316	0.061722172	-3.60151226	0.000316372	0.008557067	4605
Mrpl37	852.7023303	-0.222238191	0.062635612	-3.548112389	0.000388003	0.009575225	1498
Rfk	2452.533536	-0.222083738	0.075158533	-2.95487059	0.003128002	0.034014994	2482
Kpnb1	8065.764705	-0.222040636	0.075807849	-2.92899268	0.003400624	0.035624079	5894
Pcid2	1020.232312	-0.221941443	0.074382939	-2.98376812	0.002847225	0.032051372	2860
Rac1	7307.563627	-0.221932937	0.044924926	-4.940084695	7.81E-07	0.000173392	2325
Nipsnap1	1418.635731	-0.221896815	0.061666872	-3.598314772	0.000320286	0.008595206	1926
Cds2	29676.80268	-0.221883546	0.071032508	-3.123690147	0.001785984	0.024145507	8396
Cibar1	351.0780326	-0.221879132	0.075647723	-2.933057662	0.003356415	0.035329053	1639
L3mbtl2	806.7906791	-0.221826009	0.077304889	-2.869495194	0.004111276	0.040260912	3487
Ube2j1	4415.338246	-0.221805507	0.070647577	-3.139605281	0.001691756	0.023309641	3530
Rnf26	2136.299465	-0.221778519	0.060301068	-3.677853935	0.000235205	0.007156665	2724
Nuak1	3461.734779	-0.221752473	0.064115304	-3.458651189	0.000542887	0.011733185	5558
Mrps31	335.8139433	-0.221635583	0.079994676	-2.770629192	0.00559481	0.048287362	1515
Zfyve28	4523.862658	-0.221627192	0.074984237	-2.955650414	0.003120105	0.033954313	3983
Smg5	3584.010208	-0.221615535	0.061589875	-3.598246228	0.00032037	0.008595206	4448
Actr1a	9093.677828	-0.221546877	0.062191344	-3.562342659	0.00036756	0.009335519	2757
Cox10	765.0367511	-0.221313341	0.057891793	-3.822879333	0.000131902	0.004935423	2915
Mex3c	1976.455944	-0.221270697	0.062022763	-3.5675724	0.000360304	0.009193708	3738
Supt6	6075.554558	-0.221207396	0.079782378	-2.772634792	0.005560449	0.048123055	6488
Bace1	5378.310611	-0.221114218	0.06717549	-3.291590704	0.000996225	0.016989537	6058
Snx12	1402.856272	-0.221110179	0.060377254	-3.662143668	0.000250114	0.007424876	2415
Mt3	1366.767227	-0.220884792	0.078980713	-2.796692817	0.005162859	0.045920517	536
Bop1	1477.246975	-0.220873757	0.072332735	-3.053579483	0.002261288	0.02798917	2491
Ppp6r2	2639.763291	-0.220754268	0.062983116	-3.504975311	0.00045665	0.010549974	2887
Nlgn2	10836.88586	-0.220726949	0.076916284	-2.86970377	0.004108565	0.04025658	5568

Mad1l1	1012.669649	-0.220724854	0.062459981	-3.533860413	0.000409537	0.009925748	2640
Mib2	1473.265296	-0.220723632	0.067752558	-3.257790416	0.001122833	0.018191252	3642
Mon1b	1705.349802	-0.22067028	0.066743142	-3.306261467	0.000945498	0.01652836	4998
Cyb5r4	819.0232443	-0.220668107	0.059044552	-3.737315303	0.000185996	0.006162244	2644
Carmil3	2078.766834	-0.220647254	0.065612356	-3.36289181	0.000771306	0.014515707	4602
Capn15	1715.188865	-0.220207336	0.0780309	-2.822053006	0.004771729	0.043949029	5216
Epas1	10234.27195	-0.2201909	0.055381926	-3.975862108	7.01E-05	0.003290325	5516
Tsg101	1320.134918	-0.219904531	0.063515747	-3.462204883	0.000535769	0.011660936	1949
Txn2	1885.246981	-0.219521247	0.077162308	-2.844928459	0.00444214	0.042117464	1298
Peli3	1797.015454	-0.219312948	0.073224268	-2.995085578	0.002743679	0.031331383	4110
Phaf1	692.508409	-0.21927615	0.058909121	-3.722278425	0.000197433	0.006368414	2916
Paqr9	5541.301716	-0.218912123	0.076629071	-2.856776428	0.004279672	0.041189966	8367
Eif3m	910.7620332	-0.218901415	0.0664674	-3.293365083	0.000989958	0.016922053	1283
Calm2	9254.524201	-0.218868244	0.046236551	-4.733662814	2.21E-06	0.000355108	1205
Cic	8894.434586	-0.218862822	0.066248286	-3.303675249	0.000954263	0.016601048	8221
Sorcs2	1587.756789	-0.218853014	0.07759498	-2.820453266	0.004795586	0.044010511	5707
Sash1	5638.272196	-0.21880655	0.0781506	-2.799806388	0.005113327	0.045696778	7183
BC005624	1008.120316	-0.218743368	0.066073923	-3.310585458	0.00093101	0.016366199	1984
Icmt	3331.69486	-0.218699084	0.066292775	-3.298988221	0.00097034	0.01672145	4919
Nudt4	4836.005705	-0.218561755	0.067345224	-3.245393553	0.001172884	0.018662992	3210
Akr1a1	9713.340627	-0.218535602	0.067128413	-3.25548591	0.001131985	0.0182768	1420
Ift22	854.0214349	-0.218490379	0.071826279	-3.041928144	0.00235068	0.028628157	3071
Zfp628	951.3175605	-0.218379357	0.067331514	-3.243345429	0.001181349	0.018756954	3523
Sin3b	2505.405639	-0.21833747	0.064036416	-3.409582916	0.000650623	0.013091368	4115
Ppa2	573.4244767	-0.218301451	0.063316277	-3.44779353	0.000565186	0.011975132	1226
Ctsd	19331.86613	-0.218292686	0.064673487	-3.375304121	0.000737342	0.014100958	2127
Timp2	44029.33261	-0.218224494	0.059289947	-3.680632328	0.000232656	0.007113166	3709
Exoc4	3498.270922	-0.218217284	0.051988647	-4.197402652	2.70E-05	0.001820594	4824
Cep104	2841.508224	-0.218165865	0.060723863	-3.592753414	0.000327202	0.00866593	5151
Wbp11	2036.885361	-0.21814299	0.076687953	-2.84455358	0.004447371	0.042148878	2739
Anapc7	707.1935316	-0.217914134	0.075937889	-2.869636431	0.00410944	0.04025658	2751
Hcfc1	7406.918936	-0.217413233	0.076969803	-2.824656244	0.004733137	0.043758985	8138
Pgam1	10119.33096	-0.217351033	0.058867318	-3.692219034	0.000222306	0.006908853	1775
Tecpr1	4259.75788	-0.217121502	0.06358076	-3.414893139	0.00063807	0.012945438	5106
Ptpn9	2467.137804	-0.217016809	0.065106824	-3.33324214	0.000858402	0.015600915	4101
Slc25a16	891.8219956	-0.217005843	0.058690972	-3.697431391	0.000217792	0.006834567	3145
Atp11b	557.7801532	-0.216838039	0.065910437	-3.289889245	0.001002268	0.017043909	4102
Zdhhc8	9281.357332	-0.216618646	0.072121269	-3.003533483	0.002668642	0.030899878	4868
Maf1	1114.01275	-0.216349573	0.077843414	-2.779291924	0.005447754	0.047562094	1686
Rsl24d1	819.6073129	-0.216297591	0.051191106	-4.225296264	2.39E-05	0.001673241	1543
Rab6a	9212.279543	-0.216279796	0.076271327	-2.83566322	0.004573064	0.042841415	3214
Dnajc8	1093.627427	-0.216242855	0.070313829	-3.075395807	0.002102234	0.026789769	1401
Fdft1	1201.977768	-0.216120147	0.07231537	-2.988578318	0.002802787	0.031702691	3698
Bloc1s2	249.3338141	-0.216016859	0.073955272	-2.920912237	0.003490081	0.036209134	885
Ube2d2a	4468.269285	-0.215950155	0.063250012	-3.414231049	0.000639623	0.012964974	2480
Chrnb2	3882.900937	-0.215869651	0.071805895	-3.006294282	0.002644529	0.030774677	6399
Cntfr	2380.778303	-0.215856613	0.073374206	-2.94185961	0.003262478	0.03480821	1995
Setd3	3598.309223	-0.215839031	0.053675759	-4.021164003	5.79E-05	0.002889976	2755
Ugcg	3058.527599	-0.21557113	0.071030134	-3.034925019	0.002405955	0.029059838	4012
Ctnnbip1	1546.391033	-0.215355856	0.063624986	-3.384768612	0.000712383	0.013840707	2674
Evi5l	3609.458233	-0.215329002	0.072019075	-2.989888481	0.002790793	0.03163222	3938
Ap3b2	9055.352129	-0.215304429	0.074316385	-2.897132716	0.003765904	0.038175691	3806
Rbm19	675.919882	-0.215172599	0.074096693	-2.90394335	0.003684948	0.037571603	4010
Tubb4b	18080.10317	-0.215142183	0.071309859	-3.017004751	0.002552858	0.030082125	1598
Ndufs2	3544.917902	-0.21506019	0.055703406	-3.860808618	0.000113012	0.004472178	1623
Gde1	3180.28526	-0.215026891	0.058583944	-3.670406509	0.000242165	0.007272395	1582
Lats2	3877.584449	-0.214972801	0.055627799	-3.864485106	0.000111324	0.004422744	5191
Ppme1	4212.453986	-0.214960578	0.066654591	-3.224992803	0.001259758	0.01958461	2772
Trpc4ap	6561.246575	-0.214466518	0.051006095	-4.204723314	2.61E-05	0.001785822	3204
Uap1l1	520.8160578	-0.21440305	0.076705065	-2.795161564	0.005187378	0.046064046	3349
Ubr4	13582.43843	-0.214280853	0.06157714	-3.479876682	0.000501645	0.011201683	15798
Rwdd1	615.6918142	-0.214223108	0.073794107	-2.902983947	0.003696256	0.037634527	1090
Zfyve1	2359.042996	-0.21418828	0.057503765	-3.724769698	0.000195494	0.006338405	4036
Hpcal1	3039.256769	-0.214164698	0.061450646	-3.48514963	0.000491862	0.011049928	1526
Mrpl51	621.4286661	-0.214060197	0.064132018	-3.337805424	0.000844429	0.015436717	3427
Rab35	2570.045689	-0.213828944	0.050523528	-4.23226465	2.31E-05	0.001648509	2820
Rassf3	1348.709232	-0.213776399	0.076489042	-2.794863059	0.00519217	0.046097288	3522
Cox19	327.6745762	-0.213554262	0.059900209	-3.565167221	0.000363624	0.009251586	2461
Tpcn1	3817.548655	-0.21350721	0.076712493	-2.783213021	0.005382345	0.047241928	4712
Gpatch11	645.6033141	-0.213203683	0.075656543	-2.818046843	0.004831676	0.044153946	4134
Mon1a	980.5421968	-0.213178834	0.070690677	-3.01565697	0.002564232	0.030127325	2042

Hhatl	1066.942829	-0.213083508	0.068587188	-3.106753794	0.001891539	0.025056191	1870
Med15	1074.86353	-0.213022029	0.064636163	-3.295709688	0.000981734	0.016827267	3410
Clpb	1299.436243	-0.2129954	0.064845972	-3.284635792	0.001021143	0.017209906	4627
Lamp1	12843.46001	-0.212987649	0.068376085	-3.114943603	0.001839801	0.024653107	2229
Impdh2	688.0083022	-0.212959619	0.071694458	-2.970377708	0.002974338	0.032889027	1858
Yy1	2596.258418	-0.21290433	0.070289489	-3.028963973	0.002453939	0.029355959	6178
Clk3	873.4181597	-0.212828777	0.049593884	-4.291431902	1.78E-05	0.001403754	2496
Clip4	1051.476666	-0.212789248	0.06380154	-3.335174156	0.00085246	0.015525435	2920
Zdhhc22	1403.018982	-0.212775084	0.06492562	-3.277212995	0.001048373	0.017473364	3462
Ap1b1	6517.476578	-0.212563627	0.05861593	-3.626379834	0.000287422	0.008084792	4085
Actr8	919.8982366	-0.212514237	0.054816407	-3.876836322	0.000105823	0.00430283	2119
Rab5c	3533.143933	-0.212339352	0.075533198	-2.811205641	0.004935623	0.04469509	1915
Agpat5	916.9802879	-0.212296384	0.071168454	-2.983012435	0.002854264	0.032118688	2841
Eif1b	2976.224465	-0.212108865	0.067008332	-3.165410301	0.001548644	0.022161818	1035
Decr2	639.8588633	-0.212101923	0.061598856	-3.443276981	0.00057471	0.012078934	2224
Cct5	3655.037855	-0.212088799	0.060948539	-3.479801179	0.000501786	0.011201683	1853
Maea	2293.623353	-0.212079624	0.069607563	-3.046789963	0.002312993	0.02834626	2193
Ube4b	8516.469084	-0.21207251	0.040359727	-5.254557567	1.48E-07	5.82E-05	5626
Eif2b5	1785.066694	-0.212059325	0.05806125	-3.652338243	0.000259863	0.007581214	2554
Trap1	2351.850439	-0.21205357	0.052391025	-4.047517072	5.18E-05	0.002740802	2320
Faf1	1825.596908	-0.211998889	0.052754599	-4.018585917	5.85E-05	0.002911859	4468
Fam171a1	2551.401248	-0.211764812	0.06071253	-3.487991874	0.000486663	0.010961134	4149
Smyd5	918.4450204	-0.211676701	0.059144976	-3.578946452	0.000344982	0.008956618	2486
Mex3b	619.4687709	-0.211526275	0.067768851	-3.121290571	0.001800603	0.024305649	3355
Arid1a	5749.608452	-0.211510837	0.064914772	-3.258285155	0.001120877	0.018184267	8187
Tbc1d22b	1639.339162	-0.211500747	0.071924146	-2.94060839	0.003275684	0.034872626	3595
Ehd3	10776.45371	-0.211259479	0.075801407	-2.787012636	0.00531964	0.046822421	3659
Pigk	2675.89701	-0.211158811	0.049745696	-4.244765437	2.19E-05	0.001604716	4737
Ppid	835.2718266	-0.211086143	0.065891523	-3.203540205	0.001357491	0.020453079	1752
Lars2	790.5485975	-0.21104573	0.057163318	-3.69197832	0.000222516	0.006908853	3894
Tgoln1	7707.862528	-0.210979054	0.075075381	-2.81022953	0.004950618	0.044757129	5013
Mrpl41	1043.498207	-0.210933508	0.063909105	-3.300523557	0.000965046	0.016704036	776
St13	4339.498361	-0.21083388	0.075859298	-2.779275394	0.005448032	0.047562094	3346
Cttn	2512.906046	-0.210817487	0.072753606	-2.897691227	0.003759205	0.038142959	2913
Psmb7	1832.039956	-0.210725006	0.07409313	-2.84405595	0.004454323	0.042165696	1160
Eftud2	1807.670255	-0.210503381	0.074598861	-2.82180421	0.004775432	0.043949029	3339
Wars	3671.353115	-0.210243482	0.062385438	-3.370072998	0.000751483	0.014302571	2831
Hspa4	6620.065915	-0.210186719	0.059309609	-3.543889813	0.00039427	0.009689257	4618
Tcaf1	4438.905583	-0.210129905	0.067824948	-3.098121124	0.001947518	0.025528514	5257
Poldip3	2995.404813	-0.209547778	0.071086896	-2.947769401	0.003200757	0.034490513	3299
Clip3	15235.67063	-0.209423234	0.063921786	-3.276241907	0.001051984	0.017499237	3300
Sars2	518.5047655	-0.209404455	0.065473099	-3.198328159	0.001382269	0.020674205	1868
Rps6ka2	6161.760221	-0.209191875	0.066693027	-3.136637876	0.001708971	0.023421549	5406
Eif2ak3	1848.509079	-0.209069824	0.075207166	-2.779918921	0.005437247	0.047542772	4513
Ankrd33b	966.9984557	-0.209058506	0.075080174	-2.784470199	0.005361525	0.047106202	7466
Ptpn23	3818.821992	-0.209048262	0.059320201	-3.524065301	0.000424979	0.010121465	5346
Hars	1811.869699	-0.208935383	0.047886197	-4.363165109	1.28E-05	0.001159252	1989
Cln5	591.1019618	-0.208793253	0.071503859	-2.92002776	0.003500002	0.03624868	2445
Pgd	1640.002939	-0.208327371	0.067803284	-3.072526244	0.002122552	0.026931568	2218
Cep19	771.3711525	-0.20825491	0.062465274	-3.333930966	0.000856279	0.015575635	1718
Tnpo2	4039.624347	-0.208124048	0.061528801	-3.382546799	0.00071817	0.013903286	4863
Fastk	1997.464971	-0.207923317	0.068710957	-3.026057625	0.002477651	0.029520303	1893
Car11	1374.887601	-0.207879422	0.065458899	-3.175724385	0.001494629	0.021695322	1585
Cyhr1	2765.451876	-0.207753818	0.051934742	-4.000285908	6.33E-05	0.003066692	3959
Cab39	6196.062603	-0.207641427	0.052887413	-3.926102954	8.63E-05	0.00374813	3805
Nlk	1925.843026	-0.207548026	0.050760004	-4.088810293	4.34E-05	0.002462899	4444
Man2a2	7262.164736	-0.207524264	0.047464698	-4.372181278	1.23E-05	0.001132119	6554
Spata2	2297.100864	-0.207311644	0.066610532	-3.112295287	0.001856387	0.024769669	4012
Prnp	10870.58304	-0.207101354	0.06692117	-3.094706124	0.00197008	0.025692289	2184
Nap1l4	2340.36548	-0.206994861	0.057935174	-3.572870293	0.00035309	0.009072816	2259
Atp1b3	6148.495746	-0.206829417	0.066890545	-3.09205756	0.001987743	0.0258248	1994
Phb	2442.935699	-0.206745518	0.057249266	-3.611321732	0.00030464	0.008386674	1799
Mrpl39	418.3160385	-0.206724946	0.057667385	-3.584780972	0.000337361	0.008836908	2294
Hivep1	6574.266373	-0.206695436	0.06465815	-3.196742216	0.001389891	0.020763353	8753
Bfar	835.7511798	-0.206565428	0.061645357	-3.350867557	0.000805588	0.014931571	3003
Bcap31	2471.477609	-0.206066275	0.057503428	-3.583547648	0.000338959	0.008859886	1219
Atp6v0d1	5332.479434	-0.205606391	0.065389874	-3.144315462	0.001664759	0.023117851	1629
Kctd17	1733.035246	-0.20545553	0.068167212	-3.013993432	0.002578334	0.030236449	1632
Pcx	3732.300458	-0.205400536	0.071425149	-2.875745268	0.004030749	0.039762016	4061
Gsk3b	18968.04492	-0.204883961	0.062947214	-3.254853534	0.001134509	0.018304099	8303
Pcyox1	3755.526966	-0.204781224	0.051028047	-4.013111173	5.99E-05	0.002951618	4295

Pelp1	1895.13165	-0.204549378	0.06211736	-3.292950267	0.00099142	0.016926868	3435
Utp14a	328.3944103	-0.204206532	0.072688084	-2.809353613	0.004964109	0.044833001	2507
Txnrd3	398.8078602	-0.203924855	0.071572935	-2.849189517	0.004383076	0.041759503	2836
Taf1a	314.6009567	-0.203874821	0.067724558	-3.010352903	0.002609443	0.03048115	2384
Stat6	3917.764241	-0.203771731	0.070842877	-2.876389814	0.004022526	0.039732725	3775
Casc3	2588.183402	-0.203705435	0.04342949	-4.69048644	2.73E-06	0.000408944	3741
Nedd4	19038.90069	-0.203592255	0.070123331	-2.903345469	0.003691991	0.037608525	5499
Ubqln4	3250.795524	-0.203308122	0.071281456	-2.852188121	0.00434194	0.041507583	3330
Sema6d	3048.245565	-0.20329928	0.058948452	-3.448763703	0.000563159	0.011943704	6225
Nav1	10013.50378	-0.203240975	0.058892166	-3.451069797	0.000558369	0.011876377	12767
Tceanc2	877.5380495	-0.203096937	0.062719663	-3.238170089	0.001202991	0.0189836	6431
Ncald	2527.347845	-0.203047504	0.071570851	-2.837013956	0.004553762	0.04274256	3295
Atp5h	1878.493981	-0.203037319	0.060229579	-3.371056569	0.000748805	0.014263953	625
Wsb2	5194.673694	-0.202521322	0.062036372	-3.264557785	0.001096351	0.017918614	2421
Tmem8b	2668.513285	-0.202362743	0.048480122	-4.174138462	2.99E-05	0.001928244	5102
Pfdn2	551.1029063	-0.202350872	0.068203927	-2.966850751	0.00300867	0.033195773	1735
Usp10	1678.524464	-0.202260433	0.072680859	-2.78285694	0.005388256	0.047261451	3274
Zrsr1	2358.417927	-0.202195296	0.063162974	-3.201168085	0.001368717	0.020565833	4492
Slc7a1	5772.731535	-0.202032968	0.07174602	-2.815946676	0.004863374	0.044346657	7179
Znrf1	1470.553402	-0.201978571	0.069790567	-2.894066923	0.003802871	0.038391096	4600
Ubr3	6319.6298	-0.201876644	0.064247708	-3.142161022	0.001677058	0.023200707	8072
Ube2z	4841.58417	-0.201700505	0.048736668	-4.138578092	3.49E-05	0.00213392	3997
Aktip	2320.273088	-0.20168305	0.067162161	-3.002926749	0.002673968	0.030953395	2079
Sdhb	2249.256157	-0.201547201	0.060675785	-3.321707367	0.000894685	0.015942686	1142
Map2k1	2838.721523	-0.201488147	0.062920325	-3.202274402	0.001363471	0.020529103	2436
Fgfr1op2	1404.78419	-0.201396006	0.070977624	-2.837457709	0.004547437	0.042710547	2766
Zfp775	371.7442835	-0.201278874	0.072743047	-2.76698438	0.005657747	0.048563614	4143
Ythdf1	2223.777176	-0.201216935	0.064102495	-3.138987567	0.001695326	0.02333595	3184
Nf1	1217.16448	-0.201196502	0.069440316	-2.897401898	0.003762674	0.038160544	4133
Smyd2	902.1046512	-0.201016748	0.058444975	-3.439418848	0.000582965	0.012181576	1680
Bag4	1292.98679	-0.201009579	0.066054939	-3.043066589	0.002341805	0.028551708	4876
AI837181	1916.051032	-0.200892638	0.062350833	-3.221971976	0.001273116	0.01972253	1528
Ocel1	421.6769222	-0.200869344	0.068926219	-2.914266097	0.003565259	0.036691619	5285
Ecd	1331.22847	-0.200843968	0.062350832	-3.221191448	0.001276588	0.019755441	3132
Fndc10	1737.196578	-0.20053988	0.070626145	-2.839456699	0.004519043	0.04254491	2140
Garem1	1732.925205	-0.200349066	0.068289193	-2.933832674	0.003348046	0.035279397	4923
Kctd10	4457.486892	-0.20017217	0.064159793	-3.119900505	0.001809121	0.024360803	3103
Il6st	7364.510995	-0.200072222	0.05682229	-3.521016504	0.000429896	0.010170907	5207
Mindy1	1901.119061	-0.199945817	0.0644375	-3.102941876	0.001916073	0.025297338	2827
Psmc1	2139.470157	-0.199888102	0.055174908	-3.622808041	0.000291422	0.008145183	1591
Ext2	2080.129599	-0.1996684	0.069645222	-2.866936102	0.004144667	0.040376751	2871
Rtcb	1890.383689	-0.199461597	0.061402352	-3.248435774	0.001160414	0.018573157	2009
Picalm	2695.451619	-0.19942469	0.070278367	-2.83763978	0.004544844	0.04270444	3528
Got2	10044.80146	-0.199294617	0.062493777	-3.189031411	0.001427504	0.021053404	2530
Pde12	924.3150481	-0.199282464	0.070006708	-2.84661955	0.004418613	0.041976981	7725
Atp5b	22897.93561	-0.199261519	0.049937209	-3.990241404	6.60E-05	0.003154301	1916
Dhx8	1618.467553	-0.199205497	0.067711867	-2.941958418	0.003261437	0.03480821	4325
Daglb	962.9861422	-0.19907863	0.062866125	-3.166707503	0.001541753	0.022099394	4651
Smap2	4196.171184	-0.198984867	0.068173267	-2.918810766	0.003513695	0.036334959	2905
1600014C1	852.0718982	-0.198814903	0.055990531	-3.550866524	0.000383965	0.009515798	3282
Gpd1l	2894.785794	-0.198667439	0.05756919	-3.45093339	0.000558651	0.011876377	4391
Ociad1	3435.916829	-0.198644832	0.050263373	-3.952079235	7.75E-05	0.003526078	1523
Trim35	5349.236221	-0.198587913	0.068868841	-2.88356693	0.003931991	0.039093165	3678
Ctr9	1814.615101	-0.19857725	0.070585349	-2.813292742	0.004903699	0.044562032	4300
Pepd	981.2018162	-0.198152787	0.054960638	-3.605358174	0.000311723	0.008480748	1881
Prmt3	872.3675959	-0.197764219	0.058972994	-3.353470916	0.000798048	0.014854437	2507
Large1	3634.270726	-0.197743822	0.069646652	-2.839243753	0.00452206	0.04254491	4647
Rraga	2501.712338	-0.19757929	0.066062323	-2.99080142	0.002782464	0.03156115	1618
Ppp2r5c	659.2071137	-0.197560134	0.06953432	-2.841188832	0.004494569	0.042395136	956
Nfu1	533.5471169	-0.197513496	0.057601592	-3.428958971	0.000605901	0.012502969	950
Tmf1	3077.989183	-0.197374271	0.062939935	-3.135914765	0.00171319	0.023464297	5732
Dhx57	1871.668771	-0.197349208	0.06246934	-3.159137055	0.001582371	0.022441588	4737
Hs1bp3	761.1228308	-0.197252578	0.064187074	-3.073088789	0.002118554	0.026919724	2959
Gdi1	14159.77601	-0.196861109	0.062236896	-3.163093323	0.001561023	0.0222653	2666
Usp7	2206.270279	-0.196845594	0.04949674	-3.976940601	6.98E-05	0.00328245	4003
Eps15l1	2004.393819	-0.196503605	0.05170718	-3.80031561	0.000144512	0.005233498	3129
Mmab	894.0676919	-0.196240731	0.059893984	-3.276468134	0.001051142	0.017491842	3020
Iffo2	937.4282006	-0.196125152	0.067050212	-2.925048964	0.00344402	0.035942218	5716
Praf2	2269.796385	-0.19602955	0.064957693	-3.017803439	0.00254614	0.030011003	1339
Snx9	1422.989509	-0.195941724	0.043884372	-4.464954516	8.01E-06	0.000857154	3331
Tmem131	4578.319409	-0.195882453	0.065278143	-3.000735667	0.002693283	0.031077518	6546

Gclm	2230.594663	-0.195875565	0.069223763	-2.829600085	0.004660622	0.043338462	5589
Slc12a6	3153.245628	-0.195764967	0.053326782	-3.671044091	0.000241562	0.007264199	4556
Hectd4	38561.66767	-0.195666003	0.068612342	-2.851761033	0.004347777	0.041536294	15482
Mbnl2	7229.335854	-0.195527695	0.06546821	-2.986605192	0.002820938	0.031833297	2390
Dcun1d2	1178.915845	-0.195149697	0.049121734	-3.972777018	7.10E-05	0.003319079	2748
Atp5a1	17118.3765	-0.19429721	0.046167636	-4.208515411	2.57E-05	0.001768604	2471
Stx1a	661.7428215	-0.194078394	0.065923499	-2.943994114	0.003240062	0.03471833	2162
Txnl4b	566.4362709	-0.194078327	0.059541313	-3.259557408	0.001115862	0.018143073	1890
Kctd7	924.3032973	-0.193972688	0.063423219	-3.058386061	0.002225327	0.02776291	4291
Mgst3	1685.411576	-0.193796101	0.064007462	-3.02771107	0.002464136	0.029439055	1097
Ilrun	5784.14954	-0.193790714	0.054390719	-3.562937181	0.000366728	0.009319778	3892
Hivep3	2842.664041	-0.193712952	0.042823674	-4.523501458	6.08E-06	0.000706459	11838
Ogdh	10368.46877	-0.193647305	0.067441928	-2.871319245	0.004087625	0.040090483	4127
Mrps5	1114.669344	-0.193502278	0.059111985	-3.273486363	0.001062295	0.017584265	4481
Msh2	845.8147004	-0.193236981	0.067380652	-2.867840751	0.004132835	0.040362112	3283
Prpsap1	1648.703557	-0.193069612	0.063272731	-3.051387341	0.002277865	0.028061962	1841
Mtss1	2433.57708	-0.19305882	0.069708995	-2.769496521	0.0056143	0.048378835	4938
Rab3gap2	3488.202779	-0.192795121	0.059844095	-3.221623154	0.001274667	0.019739599	7052
Zfyve26	1679.436979	-0.192620811	0.049030236	-3.928612786	8.54E-05	0.003733847	9391
Ralyl	651.3324032	-0.19255216	0.0642004	-2.999236127	0.002706575	0.031128399	2933
Sgpp1	2343.790469	-0.192266525	0.063403555	-3.032424997	0.002425974	0.029181378	3323
Ppp3ca	9782.222029	-0.192192475	0.047712161	-4.028165407	5.62E-05	0.00284728	4758
Ak1	3720.148089	-0.191846451	0.046326414	-4.141189328	3.46E-05	0.002118596	2063
Stt3b	5091.235075	-0.191714871	0.0689797	-2.779294068	0.005447718	0.047562094	4221
Pithd1	1338.978734	-0.191501565	0.058016634	-3.300804468	0.00096408	0.016700472	1700
Slc9a8	2042.803089	-0.191416582	0.059162745	-3.235424278	0.001214621	0.019092174	4521
Arfgef2	8380.94912	-0.191222811	0.056371383	-3.392196535	0.000693347	0.013596341	8770
Slc48a1	5224.339127	-0.191209604	0.063214593	-3.024769986	0.002488223	0.029606148	2536
Slc25a4	9463.287037	-0.19119511	0.055960218	-3.416625522	0.000634024	0.012875232	1925
Ostm1	1256.82293	-0.191126444	0.062441818	-3.060872491	0.002206931	0.027616011	3006
Cops4	1229.680009	-0.191015941	0.067274238	-2.839362395	0.004520379	0.04254491	1755
Map4	15769.05119	-0.190958714	0.053057289	-3.599104246	0.000319315	0.008595206	5573
Atg2a	2671.424171	-0.190941332	0.062258658	-3.066904075	0.002162883	0.027270184	6371
Tex2	8921.781169	-0.190928669	0.058988321	-3.236719848	0.001209121	0.01903975	4934
Zfyve27	1386.26629	-0.19081204	0.053469853	-3.568591085	0.000358906	0.009172419	2573
Slc4a2	3091.197929	-0.190759056	0.064867026	-2.940770773	0.003273967	0.034872626	3924
Cdh2	2945.840886	-0.190620098	0.061417373	-3.10368369	0.001911275	0.025256756	4843
Ube4a	3324.734922	-0.190604939	0.056443415	-3.376920737	0.000733022	0.014060776	6039
Tshz3	1690.320172	-0.190197349	0.040315141	-4.717764752	2.38E-06	0.000373077	5098
Entpd7	1452.29649	-0.190163703	0.066780369	-2.847598877	0.004405041	0.041918739	5911
Tdrp	706.2142462	-0.189917791	0.05283187	-3.594758053	0.000324693	0.008634394	2463
Snrnp200	6065.881774	-0.189909049	0.060886319	-3.119075858	0.001814192	0.024401153	6745
Acvr1b	2300.885567	-0.189478117	0.055119229	-3.437604607	0.000586884	0.012240767	4418
Scamp1	4220.055359	-0.189468307	0.063043561	-3.005355423	0.002652707	0.030831695	4259
Ap5z1	830.991006	-0.189200365	0.064296435	-2.942626069	0.003254412	0.034774414	3689
Ubtd2	543.3905414	-0.189147665	0.06100402	-3.100577084	0.001931439	0.025385869	1012
Vipas39	1256.541647	-0.189024223	0.039315049	-4.807935534	1.52E-06	0.000274776	2524
Dlat	3683.520171	-0.188952062	0.055467769	-3.406519973	0.000657967	0.01320892	4035
Ppp2cb	1622.054903	-0.188762034	0.064107587	-2.944457022	0.003235219	0.034717206	1469
Wdr77	1413.453719	-0.188674036	0.063296085	-2.980816857	0.002874807	0.03224257	3682
Ist1	1916.982883	-0.188607749	0.048651969	-3.876672491	0.000105895	0.00430283	2304
Taok1	6473.075671	-0.18856478	0.052764689	-3.573692597	0.000351982	0.009060243	12409
1810013L2	2430.837721	-0.188492229	0.057996091	-3.250085082	0.001153705	0.018545753	4072
Ppp5c	2807.035181	-0.188229646	0.066773965	-2.818907741	0.004818737	0.044090688	2072
Dop1b	6648.862554	-0.188208959	0.056154471	-3.351629096	0.000803376	0.014904896	7319
Cand1	7764.557626	-0.187799288	0.060330669	-3.112832866	0.001853009	0.024754649	7766
Rpa1	1829.823182	-0.187639973	0.059173034	-3.171038564	0.00151895	0.021895287	2942
Bod1l	3336.323847	-0.187492083	0.058661317	-3.196179199	0.001392606	0.020787318	10399
Atp8b2	3971.339043	-0.187291638	0.067343047	-2.78115775	0.005416541	0.047400124	5559
Map4k4	3048.595996	-0.18724177	0.061281657	-3.055429311	0.002247386	0.027919275	5782
Prkar1a	15313.6957	-0.187223396	0.057201152	-3.273070395	0.00106386	0.017584757	3359
Trappc3	836.0703754	-0.187209583	0.063819012	-2.933445337	0.003352227	0.035309212	1336
Ccni	8382.449645	-0.186819367	0.055728728	-3.35229914	0.000801434	0.014892237	2804
Glg1	15007.39461	-0.186482794	0.056517975	-3.299530718	0.000968466	0.016710547	7021
Tmem222	891.4388074	-0.186199963	0.066291652	-2.808799572	0.004972659	0.044873352	1484
Gpt2	3374.179375	-0.185792669	0.05397717	-3.442060199	0.000577302	0.012103515	3639
Psmb3	1762.324685	-0.185705351	0.067279597	-2.760203074	0.005776544	0.049220247	766
Slc25a46	2968.762808	-0.18545754	0.06354677	-2.918441633	0.003517857	0.036365529	4371
Minpp1	1112.690086	-0.185360466	0.060870328	-3.045169483	0.002325492	0.028447434	2613
Rabl6	3635.751854	-0.184957126	0.055888103	-3.30941857	0.0009349	0.016401779	3230
Tex264	768.783603	-0.184904203	0.065243229	-2.834074997	0.004595854	0.042990796	1896

Pip4k2b	5660.680845	-0.18432649	0.0535319	-3.443301818	0.000574658	0.012078934	5053
Tex261	2422.860301	-0.184288223	0.059977706	-3.072612083	0.002121941	0.026931568	2984
Yars	2596.847434	-0.184144839	0.058935144	-3.124533612	0.001780872	0.024105919	2909
Rptor	3130.728434	-0.184115086	0.066859894	-2.753744826	0.005891769	0.049833869	6594
Slc30a9	4011.638319	-0.183673857	0.05385944	-3.410244483	0.000649047	0.013074727	5711
Arpin	637.8028663	-0.183226106	0.058525905	-3.130683851	0.001743998	0.023753058	2277
Strip1	1706.632674	-0.182987294	0.044328972	-4.127939068	3.66E-05	0.002201432	3215
Prune1	1627.130761	-0.182625707	0.060758901	-3.005744091	0.002649319	0.030822253	3140
Mtmr9	5034.595493	-0.182293658	0.061803245	-2.949580673	0.003182055	0.034347911	2570
Tmem126b	661.2590666	-0.182233081	0.053668211	-3.395549768	0.000684909	0.013530823	2259
Dcaf12	2087.973996	-0.182080895	0.065079918	-2.797804598	0.005145123	0.045827659	3442
Mepce	1885.272065	-0.181940992	0.055191037	-3.296567751	0.00097874	0.016789039	3128
Psmd8	2188.686279	-0.181697337	0.048712754	-3.729974623	0.000191499	0.006278342	1391
AU022252	2588.234199	-0.181641209	0.052593094	-3.453708384	0.000552935	0.011793424	7151
Glrx3	2098.833685	-0.181580009	0.061510675	-2.952008086	0.003157147	0.034154516	1685
Tbcel	2222.261637	-0.181416714	0.040016121	-4.533590744	5.80E-06	0.00069115	5000
Anapc2	4111.476078	-0.181366984	0.064773012	-2.800039375	0.005109637	0.045687453	3004
Slc44a1	5335.706593	-0.181054174	0.062625104	-2.891079827	0.003839206	0.038599466	3102
Extl3	7543.919128	-0.180064245	0.042924507	-4.194905414	2.73E-05	0.001827379	5977
Topbp1	744.0726002	-0.179710015	0.058863045	-3.053019373	0.002265513	0.027991388	5224
Prkce	9665.534914	-0.17904156	0.042576173	-4.20520554	2.61E-05	0.001785822	6254
Zbtb4	9369.889033	-0.179030302	0.049309381	-3.630755392	0.000282593	0.007984703	7811
Tomm70a	4545.186323	-0.178993645	0.060991385	-2.93473651	0.00333831	0.035261392	5964
Gnl2	1050.543505	-0.178742348	0.05571669	-3.20805756	0.001336347	0.020259489	2366
Ythdf2	1503.59727	-0.178553042	0.054256218	-3.290923132	0.000998592	0.017005863	4129
Dolk	948.0892414	-0.178551568	0.059017708	-3.025389728	0.002483129	0.029561544	2104
Oaz2	2624.043912	-0.178526753	0.046337978	-3.852709134	0.000116818	0.004569314	1843
Supt16	2197.197657	-0.178504022	0.057330812	-3.113579183	0.001848329	0.024714655	4673
Dyrk1a	2675.630534	-0.178074536	0.040160333	-4.434090122	9.25E-06	0.000940993	5759
Zmiz1	5658.185669	-0.177965467	0.059354862	-2.998330076	0.002714635	0.031154718	7482
Heca	1304.826322	-0.177771091	0.049244959	-3.609934784	0.000306274	0.008403754	5098
Actr3	3876.438685	-0.177696545	0.046663944	-3.808005277	0.000140092	0.005145614	2565
Tollip	4771.304043	-0.177651577	0.041921081	-4.237762317	2.26E-05	0.001624464	3788
Degs1	4343.8998	-0.177402094	0.051772371	-3.426578509	0.000611237	0.012575122	2048
Hdlbp	15861.21452	-0.177244614	0.061466027	-2.883619177	0.003931339	0.039093165	6231
Emc8	2071.293237	-0.177239049	0.047138004	-3.760003311	0.000169911	0.005785884	4942
Ulk2	5006.558784	-0.176976668	0.050134162	-3.530061324	0.000415463	0.010008683	5743
Hif1an	2960.420496	-0.176931798	0.057096746	-3.098807026	0.001943015	0.025499901	6194
Rit1	944.0601018	-0.176826665	0.045379043	-3.896659198	9.75E-05	0.004068169	1204
Csnk1e	1439.935169	-0.17651743	0.054691218	-3.227527868	0.001248649	0.019460065	2695
Trappc10	7562.526718	-0.176388626	0.055493053	-3.178571313	0.001480028	0.02154617	5047
Nploc4	4660.48208	-0.176377209	0.048213025	-3.658289657	0.000253904	0.007486847	4121
Vps52	2605.506175	-0.176309348	0.061130618	-2.88414143	0.003924824	0.039057244	3383
Samm50	2113.386615	-0.175911984	0.058541645	-3.004903342	0.002656653	0.030831695	4629
Pdcd6ip	5376.543449	-0.175836437	0.049134361	-3.578685747	0.000345326	0.008956998	5951
Drg1	1193.649111	-0.175690737	0.051155291	-3.434458789	0.000593738	0.012327478	1546
Ubr5	3318.83027	-0.175678639	0.059769536	-2.939267254	0.003289893	0.03494567	9242
Usp4	1931.711654	-0.175612728	0.058580128	-2.997820836	0.002719175	0.03116489	3662
Wdfy3	17301.85919	-0.175424558	0.055296483	-3.172436091	0.001511658	0.021847487	14274
Spring1	1382.770894	-0.175361588	0.053306772	-3.28966811	0.001003056	0.017046527	5649
Tctn1	622.4713097	-0.175272284	0.062146411	-2.820312239	0.004797694	0.044010511	5717
Pip4k2c	2522.263309	-0.175113225	0.049800786	-3.516274307	0.000437649	0.010262997	3307
Cct8	3381.623617	-0.174666846	0.054266053	-3.218712907	0.001287673	0.019794418	2394
Selenoo	1587.983699	-0.173672093	0.059451619	-2.921234045	0.003486478	0.03618814	2383
Ykt6	2111.47463	-0.173335831	0.05542607	-3.127333955	0.001763994	0.023944466	2541
Ank3	4363.353682	-0.173272871	0.04744107	-3.652381157	0.00025982	0.007581214	7404
Nudt21	1638.55809	-0.173153172	0.04730273	-3.660532329	0.000251692	0.007456624	5010
Sfrp1	2015.939411	-0.172895027	0.06185206	-2.795299423	0.005185167	0.046053707	4372
Vps33a	4073.690035	-0.172832043	0.061624444	-2.80460207	0.005037873	0.045276466	4235
Rmc1	827.8370204	-0.172121954	0.052523183	-3.277066324	0.001048917	0.017474668	2170
Gars	6272.031295	-0.171956002	0.060942718	-2.821600468	0.004778467	0.043960094	2380
Mllt1	1943.47488	-0.171803141	0.060167864	-2.855397057	0.004298305	0.041261276	3636
Vps16	1069.686658	-0.171691921	0.055852791	-3.07400789	0.002112038	0.026860217	3161
Hprt	1525.915349	-0.171579948	0.049318314	-3.479031128	0.00050323	0.011219327	1289
Atp9b	1942.949901	-0.171471639	0.057673024	-2.973168863	0.002947422	0.032698317	5150
Dock5	4494.703436	-0.171436282	0.04844052	-3.539109007	0.00040148	0.00982799	9988
Lbh	2125.61786	-0.171127422	0.061610272	-2.777579378	0.005476546	0.047716281	3069
Cdk5	2603.56043	-0.170819184	0.061166268	-2.792702408	0.005226975	0.046294113	2059
BC005537	8872.044581	-0.169953237	0.042623966	-3.987269445	6.68E-05	0.003170171	5849
Acat1	1807.670271	-0.169928883	0.034412353	-4.938019895	7.89E-07	0.000173614	3405
Eif2ak1	1690.93558	-0.169879853	0.054211183	-3.133668052	0.00172636	0.023587892	4325

Ndufb9	1294.702111	-0.169815296	0.052771773	-3.217919115	0.001291242	0.019821741	704
Snx19	4477.12252	-0.169668885	0.055804077	-3.040438872	0.002362336	0.028722378	5762
Amfr	5288.116526	-0.168798293	0.0482033	-3.501799557	0.000462127	0.010626266	3880
Nlgn3	3177.106842	-0.168765576	0.051570207	-3.272540207	0.001065857	0.017596849	8465
Gid4	2953.18371	-0.168144772	0.060729789	-2.768736298	0.005627416	0.0484389	4283
Ranbp10	902.0073211	-0.16794777	0.053670706	-3.129226058	0.001752674	0.023834308	5308
Ksr1	1815.990895	-0.167527759	0.04285234	-3.909419148	9.25E-05	0.003945264	5495
Gsk3a	5100.079645	-0.167374158	0.057906963	-2.890397766	0.003847547	0.038629227	2260
Nup133	914.3024262	-0.167302778	0.056205286	-2.976637777	0.002914281	0.032444264	5808
Mkrn2	1187.806551	-0.167269491	0.034332551	-4.872037995	1.10E-06	0.000224818	6230
Mib1	7153.033996	-0.166479625	0.051360727	-3.241379861	0.001189526	0.018848822	10231
Crkl	2721.500859	-0.166468283	0.051365249	-3.240873657	0.00119164	0.018865843	5042
Tnks	7959.968742	-0.166435695	0.045608987	-3.649186405	0.000263072	0.007634302	9163
Emc3	1920.727563	-0.166179739	0.051264231	-3.241631372	0.001188476	0.018842902	1985
Micos10	715.8832445	-0.165991328	0.046018706	-3.607040276	0.00030971	0.0084679	2549
Prkx	2037.104607	-0.165690382	0.059899561	-2.766136838	0.005672473	0.048661514	4146
Klhl29	3149.719348	-0.165533452	0.055910093	-2.960707855	0.003069329	0.033659894	7041
Tmem63b	6999.474363	-0.165084657	0.053083608	-3.109898974	0.001871513	0.024880885	3284
Ip6k1	7543.165836	-0.164330055	0.042568005	-3.860412408	0.000113196	0.004475408	4559
Ppm1f	2512.276865	-0.163398382	0.054325833	-3.007747397	0.002631918	0.030652259	4922
Rabep1	2458.735491	-0.163355909	0.055952821	-2.919529464	0.003505603	0.036281501	5408
Acox3	1743.529385	-0.162488919	0.048746523	-3.333343786	0.000858088	0.015600915	3976
Ahcyl2	4224.294445	-0.162126188	0.057408087	-2.824100155	0.004741357	0.043797969	5155
Tmem127	5420.879233	-0.16136585	0.047753782	-3.379121899	0.000727178	0.013997532	4811
Map3k1	2333.119116	-0.161334182	0.047738759	-3.379521888	0.00072612	0.013995644	6978
Rnf185	1024.396142	-0.161039889	0.053029944	-3.036772766	0.002391256	0.028958548	2787
Rrn3	2527.27649	-0.160316481	0.050833019	-3.153786351	0.00161167	0.022674267	3582
Nfs1	811.7149115	-0.160192355	0.050562369	-3.168213025	0.001533791	0.022022572	2051
Senp3	1762.945023	-0.159930225	0.050148245	-3.189148974	0.001426923	0.021053292	2313
Pex19	999.5551081	-0.158805084	0.053057387	-2.993081544	0.002761759	0.031415461	2844
Rab5b	7932.257241	-0.158795408	0.053771075	-2.953175231	0.003145234	0.034108036	3180
Slc35e1	3640.690008	-0.158790307	0.05298406	-2.996944848	0.002727	0.031238295	4383
Znrf2	832.6400152	-0.158729509	0.056195052	-2.824617154	0.004733714	0.043758985	5886
Nol10	715.3648669	-0.158548334	0.047358502	-3.347832515	0.000814462	0.01505796	3021
Kif5b	13912.39073	-0.158212486	0.050262569	-3.147719817	0.001645493	0.022944534	6032
Metap1	905.6732154	-0.157720277	0.04569494	-3.451591753	0.00055729	0.01186502	2686
Tax1bp1	4854.516509	-0.15699922	0.055451508	-2.831288573	0.004636087	0.043210849	3285
Ensa	3296.060144	-0.156960381	0.04192784	-3.743583768	0.000181414	0.00605609	3845
Phf20	3215.145721	-0.15680618	0.048980269	-3.201415238	0.001367543	0.020565833	5737
Epm2a	780.5087477	-0.156251655	0.047451825	-3.292848188	0.00099178	0.016926868	3287
Gpr107	2722.643969	-0.156110437	0.055802571	-2.797549178	0.005149193	0.045845334	6134
Lamtor5	482.972564	-0.155646452	0.056245711	-2.767259043	0.005652982	0.048532189	743
Ipo5	3563.836829	-0.155499757	0.053069295	-2.930126652	0.003388239	0.035543764	4518
Pdha1	4245.483637	-0.155494753	0.040692244	-3.821238085	0.000132783	0.004955706	2848
Entpd4b	2573.902997	-0.155196784	0.051802181	-2.99595075	0.002735906	0.031291393	3589
Entpd4	2573.902997	-0.155196784	0.051802181	-2.99595075	0.002735906	0.031291393	3589
Gnaq	8884.475109	-0.155185856	0.054852533	-2.8291466	0.004667231	0.04334413	5644
Dip2c	5263.431833	-0.155183014	0.041982195	-3.696400714	0.000218678	0.00685633	8023
Ube3c	4194.396106	-0.154746929	0.048730861	-3.175542677	0.001495565	0.021700503	5048
Hectd1	6957.741374	-0.154547022	0.046399611	-3.330782729	0.000866022	0.015672695	8988
Prdm2	4335.637345	-0.154533309	0.054913186	-2.814138455	0.004890816	0.044472538	7304
Usp22	9564.682719	-0.152856221	0.044365018	-3.445422268	0.000570168	0.01202275	4434
Gtpbp2	1391.175532	-0.15271545	0.036350837	-4.201153577	2.66E-05	0.00180174	2966
Pdzd8	7502.824699	-0.152554764	0.055076378	-2.769876498	0.005607755	0.048370601	5982
Syde1	1168.154635	-0.152003899	0.055023284	-2.762537775	0.005735392	0.049021493	3288
Zcchc18	2413.864805	-0.151802786	0.047293896	-3.209775426	0.001328387	0.020215486	2259
Fem1b	4546.48342	-0.151771588	0.053781213	-2.822018702	0.004772239	0.043949029	6785
Rnf115	1295.263711	-0.151497807	0.048351139	-3.133283089	0.001728626	0.023602191	2209
Sorl1	17631.73306	-0.151467541	0.052624001	-2.878297719	0.003998276	0.039546503	10715
Pmpca	2198.654375	-0.150644223	0.045336281	-3.322818285	0.000891129	0.015915763	3135
Crk	2091.716113	-0.150264878	0.051204867	-2.934581934	0.003339974	0.035264635	3642
Wdr48	1816.976954	-0.149472234	0.047122152	-3.172016296	0.001513845	0.021864886	3845
Vps26a	1009.628218	-0.149225221	0.048842835	-3.055212111	0.002249014	0.027923723	2597
Lars	1251.088135	-0.149097981	0.047343737	-3.149265116	0.001636816	0.022883158	3980
Vkorc1l1	1842.247044	-0.148762175	0.048281988	-3.081111238	0.002062296	0.026441773	4809
Zfp664	5007.621079	-0.148655484	0.047201077	-3.149408698	0.001636012	0.022883158	4092
Marchf6	7636.519921	-0.148309492	0.032834803	-4.516838201	6.28E-06	0.000720542	6260
Ndufa9	1595.623445	-0.148066855	0.051836958	-2.856395528	0.00428481	0.041194034	1445
Setd1a	2421.161024	-0.147129354	0.043264521	-3.400693022	0.000672153	0.013377633	5927
Sdha	5893.936492	-0.147103795	0.04220605	-3.485372234	0.000491453	0.011046383	2900
Slc25a5	4235.479898	-0.146972469	0.05152798	-2.852284701	0.004340621	0.041507583	1240

Psma6	1408.938509	-0.14636672	0.052959019	-2.763773276	0.005713723	0.048919924	1042
Psme3	3076.723191	-0.146036035	0.051962049	-2.810436403	0.004947437	0.04474677	2664
Heatr5b	3072.653001	-0.145625148	0.052060428	-2.79723301	0.005154235	0.045871645	6388
Hspa9	4338.413627	-0.144804674	0.043013078	-3.366526708	0.000761212	0.014412873	3049
Klf9	7771.617229	-0.143923487	0.048077314	-2.993584184	0.002757214	0.031379997	4486
Asxl1	3063.739853	-0.143900921	0.035774422	-4.022452782	5.76E-05	0.002880743	6968
Castor2	4364.674642	-0.143825662	0.045046822	-3.192803714	0.001408987	0.020919412	7367
Kidins220	7241.473757	-0.141185561	0.048851974	-2.890068679	0.003851577	0.038653161	7409
Kif3b	2820.335635	-0.1409736	0.039700674	-3.550911995	0.000383899	0.009515798	5635
Sppl3	3094.391511	-0.140847119	0.050883844	-2.768012536	0.005639929	0.048505372	3204
Rab1a	3712.986832	-0.14029615	0.042305209	-3.316285501	0.000912226	0.016139239	2657
Adss	1527.961364	-0.140292461	0.049194001	-2.85182054	0.004346963	0.041536294	2771
Jmy	4094.432858	-0.140087536	0.036982287	-3.787962991	0.000151887	0.00539666	8776
Morf4l1	5441.210049	-0.139316149	0.042541726	-3.274811891	0.001057324	0.017541596	1824
Ufd1	1238.206402	-0.139241006	0.05053127	-2.755541403	0.005859509	0.049693925	1996
Aagab	1241.824239	-0.138998272	0.047754705	-2.910671738	0.003606527	0.037021004	5294
Armh3	1173.272541	-0.137784094	0.044746418	-3.079220638	0.002075429	0.026571416	3676
Helz	3921.158917	-0.137573553	0.04993806	-2.754883828	0.005871298	0.049745926	13147
Coro1c	3218.85979	-0.137104334	0.037591432	-3.647222988	0.00026509	0.00766755	3491
Pcgf3	2040.115664	-0.134442966	0.036680465	-3.665247091	0.0002471	0.007370249	7708
Prps2	722.5405092	-0.134110808	0.047049558	-2.850415879	0.00436621	0.041676165	3699
Exoc2	1422.481939	-0.131193213	0.042984387	-3.052113158	0.002272364	0.02802709	4256
Kpna6	3727.282731	-0.131067224	0.041365841	-3.168489273	0.001532334	0.022008842	5710
Hip1	5371.478408	-0.131000655	0.046377575	-2.824655159	0.004733153	0.043758985	7835
Fbxl20	3730.545251	-0.125297292	0.039599517	-3.164111588	0.001555571	0.022214882	8601
Ddx3x	5957.036974	-0.124601541	0.044762785	-2.783596689	0.005375984	0.047195511	4692
Nup214	2993.029423	-0.124071739	0.039125391	-3.171130945	0.001518467	0.021895287	6605
Rxra	2251.054449	-0.123734062	0.042243799	-2.929046743	0.003400033	0.035624079	4905
Mknk1	901.0816194	-0.122263395	0.044004599	-2.778423116	0.005462344	0.047648278	2543
Scarb2	6802.915264	-0.117868013	0.041471632	-2.842135872	0.004481239	0.042323881	4698
Cop1	1352.468561	-0.116279619	0.037491721	-3.101474569	0.001925594	0.025331761	5116
Rad54l2	1959.607347	-0.112087162	0.031284559	-3.582826935	0.000339896	0.008863408	9305
Dusp3	5285.887407	-0.109847375	0.039883326	-2.754217994	0.005883257	0.049808854	4212
Nudcd3	2272.08124	-0.108854545	0.037460653	-2.905836804	0.003662724	0.037405731	3927
Tnrc6b	9319.502234	-0.10143971	0.036136056	-2.807160525	0.004998033	0.045009937	17332
Rb1	1272.240969	0.170609127	0.056162997	3.03774969	0.002383518	0.028899154	4656
Exoc5	867.9312416	0.178097309	0.054373581	3.27543827	0.001054982	0.017529202	12699
Brwd1	1590.463212	0.190559229	0.060638005	3.142570901	0.001674711	0.023190139	9843
Son	2639.298636	0.199809707	0.065653689	3.043388902	0.002339298	0.028544889	7529
Scai	778.1986785	0.200100472	0.056240474	3.55794428	0.000373769	0.009431526	10709
Aebp2	671.2251584	0.202587952	0.060296731	3.359849689	0.000779849	0.014645902	5932
Pnpla8	759.692711	0.204738494	0.065555138	3.123149466	0.001789269	0.024174921	13507
Tardbp	1302.973999	0.20692473	0.06844496	3.023228159	0.002500936	0.029709175	7477
Ogt	3246.659516	0.221330795	0.080093071	2.763420018	0.005719911	0.048944313	5384
Rev3l	1207.664099	0.222782622	0.080052344	2.782961871	0.005386514	0.047261451	10399
Zbtb37	1417.498439	0.22997114	0.066273944	3.470008373	0.000520442	0.01142919	18814
Mdm4	1700.856121	0.258132235	0.071961604	3.587082834	0.000334398	0.008787695	7105
Ivns1abp	673.3179509	0.269456228	0.083752268	3.217300666	0.001294029	0.01985066	2783
Dusp11	1883.521272	0.271825877	0.070289516	3.867232156	0.000110078	0.004397252	6435
Hcfc2	184.4875451	0.274101228	0.095065137	2.88329916	0.003935335	0.039099309	2887
Anapc1	519.9101218	0.276324596	0.09391707	2.942219106	0.003258693	0.034807683	4214
Nxf1	1105.57544	0.284079324	0.095206276	2.983829799	0.002846651	0.032051372	4042
Smc5	518.7600544	0.285031579	0.092450077	3.083086434	0.002048656	0.026336018	5683
Pwwp2a	214.8753016	0.286332491	0.071362475	4.012367705	6.01E-05	0.002956217	2739
Vezt	338.3464215	0.296129752	0.099487909	2.976540117	0.002915209	0.032444264	3705
Fam53b	1473.193271	0.303439929	0.100989835	3.00465813	0.002658795	0.030834589	5465
Cep95	109.8383668	0.309177327	0.111141008	2.78184741	0.005405045	0.047358051	2696
Phf14	427.0795817	0.314252442	0.106780161	2.942985283	0.003250639	0.034771074	3985
Nopchap1	5072.032875	0.327187171	0.105483905	3.101773396	0.001923651	0.025330167	123179
Zcchc7	986.693736	0.32878727	0.103445545	3.178360855	0.001481103	0.02155468	4251
Gm14399	128.5494356	0.328932373	0.112571081	2.921997106	0.003477948	0.036147955	1911
Fus	412.0725917	0.330488166	0.096455648	3.426322576	0.000611813	0.012581091	5536
Prpf4b	821.9423288	0.348105462	0.098111972	3.548042669	0.000388105	0.009575225	7844
Ggnbp2	522.9338011	0.351247743	0.126160602	2.78413179	0.005367122	0.047136541	2950
Srek1	426.6365494	0.351646419	0.126649192	2.77653898	0.005494105	0.047795496	6330
Qk	3772.999726	0.351848944	0.113437043	3.101711164	0.001924056	0.025330167	8547
Brd8	352.1678501	0.355792512	0.079642833	4.467351278	7.92E-06	0.000855471	3869
Pnisr	600.5421271	0.362658575	0.122572102	2.958736677	0.003089029	0.033758184	2922
Marf1	278.1425061	0.362920511	0.109636068	3.310229184	0.000932196	0.016372477	2782
Txndc9	116.8913855	0.368699711	0.127541844	2.890813714	0.003842458	0.038621308	3472
Gm4787	110.0167631	0.370323083	0.132441456	2.796126637	0.005171913	0.045973142	2474

Ecpas	239.4578938	0.370386715	0.132313481	2.799311988	0.005121163	0.045734701	4946
Cspp1	92.73585941	0.377913746	0.127424153	2.965793671	0.00301903	0.033266074	2535
Fdx1	95.4989917	0.378693086	0.123737929	3.060444666	0.002210086	0.027638699	2759
Apex2	284.2221816	0.379477011	0.099071375	3.830339586	0.000127967	0.004867313	4742
Ddx17	1951.34035	0.382211324	0.08784412	4.35101774	1.36E-05	0.001200314	2821
Rc3h2	976.1671381	0.384210473	0.108743547	3.5331795	0.000410594	0.009934919	8799
Safb2	221.1687579	0.387983508	0.123626697	3.138347283	0.001699034	0.023357739	2636
Zkscan3	1450.128349	0.38925401	0.094648111	4.112644244	3.91E-05	0.002298656	5788
Zfp207	241.1846447	0.39188714	0.124039928	3.15936283	0.001581145	0.022437277	2898
Hnrnpr	968.5511224	0.39217966	0.10379788	3.778301235	0.000157902	0.005531596	21452
Zfp950	339.3691972	0.405272929	0.105776751	3.831398919	0.000127417	0.004854312	4825
Krba1	337.3110387	0.406588724	0.136550195	2.977577033	0.002905366	0.032419905	8380
Slc25a36	172.6210768	0.407320822	0.136675971	2.980193377	0.002880665	0.032283564	3229
Usp48	419.584565	0.407712794	0.10916707	3.734759884	0.000187894	0.006196346	3036
Ppcdc	200.8590147	0.416521974	0.123319718	3.377578059	0.000731272	0.014046266	1814
Unc13b	418.3098741	0.422253715	0.128002283	3.298798305	0.000970997	0.01672145	14754
Atp2b1	120.6572741	0.42667857	0.129902053	3.284617608	0.001021209	0.017209906	2393
Rbmx	291.320143	0.429385378	0.099999637	4.293869354	1.76E-05	0.001396003	3107
Olfr46	183.4745903	0.42949649	0.154475622	2.780351253	0.005430013	0.047497057	7750
Fam193a	142.7457435	0.429751309	0.099666998	4.311871705	1.62E-05	0.001320405	2283
Zfp101	203.2871583	0.432430122	0.154257402	2.803302241	0.005058224	0.045356889	6095
Strada	103.6157	0.436357665	0.155353158	2.808811037	0.004972482	0.044873352	3386
Luc7l3	341.198721	0.436373329	0.138736462	3.145339888	0.00165894	0.023066188	5244
Skp2	84.01105074	0.440791234	0.158220073	2.785937491	0.005337316	0.04694041	6184
Gm42669	80.18228184	0.44534251	0.143885249	3.095122758	0.001967315	0.025673192	3445
Cdc37l1	523.5774541	0.445500242	0.125909524	3.538256912	0.000402778	0.009848795	4163
Bcl7a	248.9792266	0.446122875	0.157563714	2.831380798	0.004634751	0.043210849	4642
Eif4e2	95.37050541	0.450448882	0.1623391	2.774740541	0.005524576	0.047944729	1807
Kif16b	75.55165779	0.45364695	0.155340362	2.92034178	0.003496477	0.03624868	2158
Nptn	127.6012713	0.455686925	0.132217367	3.446498262	0.000567902	0.01200376	4531
Zc3h11a	74.57772926	0.456061382	0.142134944	3.208650653	0.001333594	0.020237057	786
Slc38a6	1800.284048	0.456170494	0.107091359	4.259638668	2.05E-05	0.001530981	3927
Kif1b	501.1111794	0.458235155	0.109217265	4.195629278	2.72E-05	0.001827379	10434
Cep170	119.0182538	0.461626564	0.16227324	2.844748546	0.004444649	0.042132172	2708
Nrd1	178.3460208	0.463463967	0.155567318	2.979185947	0.002890153	0.032343245	2190
Araf	360.8617209	0.464333081	0.087505419	5.306335167	1.12E-07	4.75E-05	2854
Zfp386	145.9656493	0.464518847	0.129230862	3.594488508	0.000325029	0.008634394	4779
Acadsb	149.5804344	0.467475039	0.12884771	3.628120657	0.000285492	0.008043326	2466
Ubap2l	199.9521546	0.469127421	0.1669128	2.810613814	0.00494471	0.044737082	3503
Gfm2	96.1263768	0.469169068	0.165713746	2.831201878	0.004637344	0.043213403	4416
Cd46	107.4888793	0.475050993	0.166311818	2.856387469	0.004284919	0.041194034	6288
Tom1l2	336.7799092	0.476917193	0.104189124	4.577418204	4.71E-06	0.000586826	2151
Eif2b3	42.49182945	0.478150277	0.163171277	2.930358126	0.003385716	0.035525772	3754
Mbtd1	66.08331812	0.478398706	0.173028293	2.764858257	0.005694754	0.048805037	2138
Tut4	426.5652471	0.478879181	0.141481216	3.384754488	0.000712419	0.013840707	6054
Calcoco1	154.9994321	0.48278375	0.122001954	3.957180481	7.58E-05	0.003473218	2968
Mbp	4672.25815	0.48624017	0.175369998	2.772653103	0.005560136	0.048123055	4797
Rhno1	78.08004114	0.491155699	0.15705293	3.12732593	0.001764042	0.023944466	3697
Rapgef6	1165.368309	0.495395357	0.17464455	2.83659213	0.004559782	0.04277167	8189
Lcor	69.13786901	0.500637978	0.142654925	3.509433531	0.000449062	0.010423983	4663
Slc35b3	96.87757577	0.505463163	0.157028077	3.21893493	0.001286677	0.019794418	2486
4833420G1	154.595038	0.510166989	0.174297144	2.926995699	0.003422536	0.035775512	3406
Ankrd55	50.744329	0.510582321	0.183570664	2.781393877	0.005412602	0.047391453	3209
Gapvd1	179.8285222	0.518462054	0.124017909	4.180541799	2.91E-05	0.001894177	8339
Nisch	549.3777817	0.519105354	0.115211604	4.505669028	6.62E-06	0.000751655	2415
Cdk11b	133.5214855	0.52080572	0.157833381	3.299718458	0.000967819	0.016710547	3170
Hmg20a	86.76048372	0.522392326	0.168666067	3.097198723	0.001953589	0.025577583	2468
Zfp426	210.8396942	0.522575578	0.184991086	2.824868967	0.004729995	0.043758985	4262
Psmd13	64.2039786	0.523215408	0.159980533	3.270494208	0.001073597	0.01769222	1215
Sorbs2	146.6072786	0.524839979	0.141306432	3.714197387	0.00020385	0.006532252	3798
Peg3	1677.550937	0.526033902	0.153313077	3.431109146	0.000601119	0.012425098	8986
Plpbp	182.700232	0.528294465	0.121881807	4.334481716	1.46E-05	0.001245637	3954
Ptn	80.52759568	0.532137277	0.153466753	3.467443392	0.000525434	0.011510076	2558
Cabin1	127.5799626	0.538335637	0.178191321	3.0211103	0.002518496	0.029817416	2115
Fto	322.9670468	0.541020965	0.106000655	5.103939847	3.33E-07	0.000101563	4707
Dnase1l2	51.44284055	0.546092753	0.171732765	3.179898438	0.001473267	0.021507317	1629
Btf3l4b	53.09799288	0.546720505	0.179204243	3.050823439	0.002282147	0.028097062	2161
Plekha1	75.66664754	0.550596855	0.19933109	2.762222671	0.005740931	0.049030685	2397
BC024063	71.26908221	0.550876193	0.16945971	3.250779745	0.00115089	0.018507268	2726
Naa20	46.80320177	0.556299702	0.174145329	3.194456641	0.001400943	0.020862662	1883
1700029J0	75.67973161	0.567874336	0.172483565	3.292338818	0.000993578	0.016950978	2740

Sdhaf2	61.24207471	0.568879747	0.140704807	4.043072585	5.28E-05	0.002769512	3916
Slc7a2	72.48362476	0.570448989	0.163085106	3.497860737	0.000469006	0.010700739	3681
Sfxn3	144.5114195	0.570863556	0.164974789	3.460307844	0.000539558	0.011694529	2867
Zfp787	48.78241799	0.590922484	0.147102788	4.017071965	5.89E-05	0.002917421	880
Gm21897	194.2671886	0.597268967	0.154206856	3.873167397	0.00010743	0.004339638	1500
Mcm9	92.14279411	0.602567252	0.154323595	3.904569827	9.44E-05	0.003990379	4132
Sorbs1	142.1611068	0.602627788	0.163423142	3.687530298	0.000226441	0.007001044	1057
Limch1	672.2297204	0.608581765	0.188274832	3.232411679	0.001227501	0.0192122	7498
Klf15	83.73374085	0.626609751	0.209195151	2.99533592	0.002741427	0.031313811	1545
Tenm4	364.2023494	0.627977492	0.174656427	3.595501771	0.000323767	0.008617725	11021
Kremen1	100.691742	0.628354415	0.187918502	3.343760231	0.000826511	0.015210369	3183
Cyth2	106.1819247	0.631656062	0.210224842	3.004668977	0.0026587	0.030834589	2527
Mocs2	43.30516901	0.634474228	0.186279317	3.406036904	0.000659133	0.013219246	4559
Foxo3	92.56408131	0.634695607	0.220965852	2.872369648	0.004074061	0.040025944	1316
Dtymk	55.05315719	0.638633846	0.175457108	3.639828869	0.000272819	0.007824201	1040
2410002F2	129.2123613	0.639587076	0.231901488	2.758011948	0.005815407	0.049424778	4201
Ube2i	117.5325381	0.64012796	0.14788831	4.328455454	1.50E-05	0.001262281	1144
Rnf145	127.316923	0.645040574	0.160259944	4.024964422	5.70E-05	0.002870131	4214
Tfeb	73.35766531	0.648513488	0.195729293	3.313318497	0.000921959	0.016259101	1974
Stxbp4	38.20190937	0.651027631	0.193418868	3.365895157	0.000762957	0.014439692	2952
Ints6l	110.1501827	0.652095657	0.167202826	3.900027719	9.62E-05	0.004027264	2766
Frem1	35.6160153	0.653119723	0.225657119	2.894301431	0.003800032	0.038371244	3574
Kmt5b	120.3851233	0.65439152	0.159734408	4.096747404	4.19E-05	0.002407993	3216
Hook2	45.7963854	0.665232525	0.24160811	2.753353457	0.005898817	0.049882958	3080
Zeb2	259.1922168	0.669684817	0.196752022	3.403699793	0.000664798	0.01330352	2070
Smim1	60.63669967	0.6701669	0.20507076	3.267978822	0.001083185	0.017809355	5091
Utp14b	108.5181625	0.685708478	0.198545828	3.45365342	0.000553048	0.011793424	3685
Elf2	28.77431556	0.690679359	0.244848375	2.820845184	0.004789731	0.044008471	2161
BC004004	56.08739967	0.691019592	0.196269401	3.520770885	0.000430294	0.010170907	1694
Acot6	26.83308017	0.698561787	0.215667031	3.239075461	0.001199178	0.018951071	3622
Nnt	94.10881771	0.700409629	0.225036201	3.112430911	0.001855534	0.024765807	3198
Eif4a2	1828.315587	0.708005606	0.183732007	3.853469057	0.000116456	0.004559205	2208
Frmd4a	55.81774934	0.710773987	0.244141267	2.911322592	0.003599022	0.036952595	2094
Il18bp	74.31724169	0.712535551	0.182049849	3.913958479	9.08E-05	0.003886868	1594
Eml4	100.7199535	0.73020327	0.236791416	3.083740462	0.002044158	0.026297207	5286
Srrm2	5522.208256	0.733687397	0.23977589	3.05988812	0.002214197	0.027663304	8864
Txnrd1	174.6591446	0.7348174	0.21819567	3.367699282	0.000757982	0.0143808	3622
Zfp507	115.7883293	0.736782609	0.265514324	2.774926032	0.005521426	0.047936317	3598
Acrbp	63.14980867	0.73899897	0.26212401	2.819272337	0.004813266	0.04408651	1883
Uckl1	23.51997758	0.740265382	0.235856738	3.138622994	0.001697437	0.023350379	1043
Dcaf17	47.90852852	0.740938004	0.180024463	4.115762888	3.86E-05	0.002275592	2490
Fkbp5	90.13421735	0.749358154	0.194089007	3.86089953	0.00011297	0.004472178	2431
Arhgef9	111.3880184	0.760515571	0.190317459	3.996036796	6.44E-05	0.00310852	6222
Dusp16	65.30715226	0.763673681	0.252843259	3.020344236	0.002524875	0.029840362	3305
Rpl31	28.49431209	0.76780105	0.275433194	2.787612628	0.005309799	0.046782631	703
Atp1b1	50.8650144	0.76994523	0.263665594	2.920158134	0.003498538	0.03624868	628
Pcdh9	620.2965938	0.770805343	0.279358487	2.759197876	0.005794344	0.049293406	3014
Ap2b1	87.62282232	0.798997408	0.243946216	3.275301505	0.001055493	0.017531066	3596
Ccdc88a	64.39519064	0.840620579	0.217851287	3.858689981	0.000113996	0.004494936	1716
Bcl2l2	80.7147236	0.840900859	0.219917173	3.823716209	0.000131455	0.004931253	3813
Arpp21	76.29997692	0.8459942	0.295662601	2.861350057	0.00421841	0.040823771	3419
Aspm	26.54313268	0.846900482	0.282680906	2.995959272	0.00273583	0.031291393	9867
Abcg1	52.70340767	0.850590465	0.219380031	3.877246526	0.000105645	0.004300644	1372
Hyal2	27.00302632	0.854628002	0.297115251	2.876419161	0.004022152	0.039732725	2968
Usp53	40.49719071	0.862025672	0.246857827	3.491992465	0.000479432	0.010848285	3586
Tlk2	46.36507092	0.868051727	0.310174816	2.79858867	0.005132647	0.045786708	2776
Grik2	129.3007026	0.882938787	0.222402035	3.970012168	7.19E-05	0.003343618	2610
Dnm1l	75.95748477	0.884962918	0.308458564	2.868984759	0.004117916	0.040312669	2151
Apobec3	30.02033011	0.896850835	0.281567799	3.185203846	0.001446521	0.021248341	2765
Nsmf	335.8731874	0.899945001	0.314846534	2.858360837	0.004258358	0.041089271	2922
Atxn7l1	46.35745254	0.925619214	0.301510195	3.069943337	0.002140994	0.02707963	3548
Ttc14	71.69892143	0.94375185	0.252299692	3.740598509	0.000183583	0.006114877	2724
Trpm3	80.32724901	0.974926699	0.303006622	3.217509547	0.001293087	0.019843134	3486
Efr3a	39.12513043	0.989025353	0.267704881	3.694461414	0.000220353	0.006875681	2848
Atf7	67.371551	1.00913313	0.363451797	2.776525355	0.005494335	0.047795496	2887
Prx	908.3392966	1.019369947	0.26472615	3.850658292	0.000117801	0.00459141	4497
Cux2	519.8656929	1.02630744	0.344557241	2.978626822	0.002895432	0.032366554	5113
Odf2	46.36820187	1.065414276	0.297626516	3.579702134	0.000343986	0.008938149	2450
Ano10	23.90870573	1.10421472	0.39500504	2.795444633	0.005182838	0.04605163	1770
Rnf38	285.6590976	1.154793236	0.344003442	3.356923489	0.000788149	0.014713792	5061
Armcx3	114.6854221	1.166470125	0.27806086	4.195017333	2.73E-05	0.001827379	3488

Wrn	34.82694698	1.280080285	0.430023087	2.97677107	0.002913014	0.032444264	4856
Btbd6	394.0499172	1.301452226	0.26024945	5.000787603	5.71E-07	0.000137926	2085
Gucy1a1	32.59822446	1.848344509	0.592291948	3.12066459	0.001804434	0.024342422	4710
Dsp	27.07086557	2.535881958	0.888063211	2.855519659	0.004296646	0.041261276	9592
Slc5a6	32.47365229	2.548425107	0.883527493	2.884375561	0.003921907	0.039045665	3248
Spint2	23.0491309	3.923211312	1.317655373	2.977418369	0.00290687	0.032428459	1384
Slc6a5	98.77748379	4.415486439	1.429741048	3.088312003	0.00201297	0.026072875	6966
Efcab12	22.36827885	7.930656045	1.585586715	5.00171701	5.68E-07	0.000137926	2384
Med6	32.48410656	8.46897098	1.605627142	5.274556438	1.33E-07	5.37E-05	1003
Anks1b	32.5677976	8.472681366	1.598248789	5.301228085	1.15E-07	4.82E-05	2799
Capzb	34.30783101	8.547696689	2.616377046	3.266997279	0.001086947	0.017832269	730
Cyfip1	48.20280639	9.03824767	2.758429519	3.276591846	0.001050681	0.017490801	2908
Eya3	23.02101658	22.43842344	2.987683883	7.510307087	5.90E-14	6.48E-11	5020
Gm14325	23.97141907	22.49453374	2.987642035	7.529193081	5.11E-14	5.76E-11	1494
Srr	24.66520194	22.53708309	2.987613523	7.543506854	4.57E-14	5.29E-11	3239
Ppip5k1	25.29736534	22.57259134	2.987588899	7.555454281	4.17E-14	4.96E-11	5404
Zmynd8	25.71368359	22.59506033	2.987573346	7.563014431	3.94E-14	4.81E-11	5093
Zfp267	27.73084153	22.7004402	2.987504639	7.598461907	3.00E-14	3.88E-11	6503
Mef2a	34.91939715	22.95740772	2.987324234	7.684940075	1.53E-14	2.04E-11	2476
Synrg	42.64194672	23.15769101	2.987198287	7.752311292	9.02E-15	1.30E-11	3651
