# Supplementary material for: Gender-different effect of Src family kinases antagonism on photophobia and trigeminal ganglion activity
Source: J Headache Pain. 2024 Oct 11;25(1):175. doi: 10.1186/s10194-024-01875-3 (PMC11468534; doi:10.1186/s10194-024-01875-3)
Supplement: Supplementary file 3 — Supplementary Material 3: S3 Table. Gene expression of 33 candidate genes of TG (criteria: absolute value of log2FoldChange greater than 20) in male photophobia (UMB) vs. control (DMSO) groups. [file 10194_2024_1875_MOESM3_ESM.pdf]

Supporting Table 3: Gene expression of 33 candidate genes of TG (criteria: absolute value of log2FoldChange greater than 20) in male photophobia (UMB） vs. control (DMSO) groups.

GeneID	baseMean	log2FoldChange	lfcSE	stat	pvalue	padj	length
Scn8a	257.2014901	-26.25555456	2.986726589	-8.790745916	1.49E-18	6.53E-14	7120
Flot2	145.1322853	-25.622442	2.986801122	-8.578556442	9.61E-18	1.10E-13	2699
Add2	127.1955564	-25.55770401	2.986825246	-8.556812636	1.16E-17	1.10E-13	2289
Rreb1	123.9258763	-25.52342244	2.986830397	-8.545320305	1.28E-17	1.10E-13	8395
Mast2	119.1556595	-25.46828832	2.986838418	-8.526838335	1.50E-17	1.10E-13	5445
Baz2a	70.53504558	-24.64203859	2.986982041	-8.249811431	1.59E-16	9.96E-13	8385
Magi2	51.84183823	-24.32901978	2.987108951	-8.144671045	3.80E-16	2.09E-12	4478
Erc1	58.22990974	-24.24608183	2.987056446	-8.11704843	4.78E-16	2.33E-12	8356
Tdp1	42.9359032	-24.07854831	2.987208269	-8.060552244	7.60E-16	2.62E-12	2019
Tnk2	49.71361202	-24.07534091	2.987129472	-8.059691128	7.65E-16	2.62E-12	939
Clvs1	42.29274194	-24.05420447	2.987217067	-8.052379164	8.12E-16	2.62E-12	3526
Birc2	41.17666479	-24.02145917	2.987233019	-8.041374414	8.88E-16	2.62E-12	3130
Asap1	69.39943913	-24.01737149	2.986987848	-8.040665953	8.94E-16	2.62E-12	3228
Dync1i2	37.9572083	-23.90854738	2.987284166	-8.003439262	1.21E-15	3.13E-12	2466
Lasp1	39.89513944	-23.85516029	2.987252442	-7.985652621	1.40E-15	3.41E-12	686
Ambra1	34.43407912	-23.76881103	2.987351038	-7.956484096	1.77E-15	4.10E-12	4690
Gtf2i	30.59348367	-23.61742404	2.987441594	-7.905568461	2.67E-15	5.86E-12	4381
Stat3	28.92380479	-23.54004105	2.987488408	-7.879542222	3.29E-15	6.88E-12	2506
Gm28635	25.92558239	-23.38733426	2.987587652	-7.828166731	4.95E-15	9.72E-12	4734
Tyk2	25.77005346	-23.37728823	2.987593368	-7.824789171	5.09E-15	9.72E-12	4828
Ckmt1	24.80768624	-23.3315088	2.98763081	-7.80936812	5.75E-15	1.01E-11	851
Fyn	23.34015111	-23.24864831	2.987693757	-7.781469655	7.17E-15	1.17E-11	675
Pabpc4	21.70240843	-23.15054333	2.987774039	-7.748425092	9.30E-15	1.30E-11	3050
Snx25	21.65576292	-23.14514014	2.987776351	-7.746610664	9.44E-15	1.30E-11	3067
Fubp1	33.81722288	-23.14389691	2.98736401	-7.747263753	9.39E-15	1.30E-11	2552
Eya3	23.02101658	22.43842344	2.987683883	7.510307087	5.90E-14	6.48E-11	5020
Gm14325	23.97141907	22.49453374	2.987642035	7.529193081	5.11E-14	5.76E-11	1494
Srr	24.66520194	22.53708309	2.987613523	7.543506854	4.57E-14	5.29E-11	3239
Ppip5k1	25.29736534	22.57259134	2.987588899	7.555454281	4.17E-14	4.96E-11	5404
Zmynd8	25.71368359	22.59506033	2.987573346	7.563014431	3.94E-14	4.81E-11	5093
Zfp267	27.73084153	22.7004402	2.987504639	7.598461907	3.00E-14	3.88E-11	6503
Mef2a	34.91939715	22.95740772	2.987324234	7.684940075	1.53E-14	2.04E-11	2476
Synrg	42.64194672	23.15769101	2.987198287	7.752311292	9.02E-15	1.30E-11	3651
