# Supplementary material for: Gender-different effect of Src family kinases antagonism on photophobia and trigeminal ganglion activity
Source: J Headache Pain. 2024 Oct 11;25(1):175. doi: 10.1186/s10194-024-01875-3 (PMC11468534; doi:10.1186/s10194-024-01875-3)
Supplement: Supplementary file 4 — Supplementary Material 4: S4 table. Gene expression of 32 genes of TG (criteria: absolute value of log2FoldChange greater than 20) in male photophobia mice in the presence or absence of pretreatment of SRCT groups. [file 10194_2024_1875_MOESM4_ESM.pdf]

Supporting Table 4：Gene expression of 32 genes of TG (criteria: absolute value of log2FoldChange greater than 20) in male photophobia mice in the presence or absence of pretreatment of SRCT groups.

GeneID	baseMean	log2FoldChange	lfcSE	stat	pvalue	padj	length
Repin1	95.45922301	-25.13464925	2.986882666	-8.415010586	3.93E-17	3.67E-13	3145
Nfat5	54.04328904	-24.35256291	2.987076245	-8.152641884	3.56E-16	1.16E-12	5899
Snx14	48.46336325	-24.21678225	2.987127618	-8.107046417	5.19E-16	1.57E-12	3112
Kat7	37.47141806	-23.86662314	2.987273546	-7.989433433	1.36E-15	3.59E-12	3324
Zfp869	32.5601884	-23.67036576	2.987370616	-7.923478138	2.31E-15	4.70E-12	3199
Fam13c	32.52910613	-23.66699811	2.987371316	-7.922348983	2.33E-15	4.70E-12	3262
Pja2	27.151529	-23.30319568	2.987518101	-7.800185601	6.18E-15	1.09E-11	4531
Gm14325	23.824918	-23.25304983	2.987642108	-7.78307742	7.08E-15	1.20E-11	1494
Nes	24.65187567	-23.1329672	2.987608056	-7.742972562	9.71E-15	1.42E-11	5958
Zmynd8	25.57768887	-23.04088212	2.987572873	-7.712241038	1.24E-14	1.74E-11	5093
Srr	24.45045639	-21.78352955	2.987616493	-7.291273696	3.07E-13	3.33E-10	3239
Arrb2	25.50321129	22.53296517	2.987599279	7.542164481	4.62E-14	5.29E-11	1443
Stk3	24.25759133	22.54775073	2.987649151	7.546987477	4.45E-14	5.24E-11	2730
Slc37a1	26.30357241	22.66443381	2.987569816	7.586244075	3.29E-14	4.10E-11	2972
Kmt2d	31.69309066	22.92159489	2.98740968	7.672732347	1.68E-14	2.23E-11	19805
Myo7a	32.11561202	22.94005779	2.987399377	7.678939071	1.60E-14	2.19E-11	6849
Katna1	38.24868356	23.18627252	2.987275766	7.761677973	8.38E-15	1.27E-11	1774
Pgap6	39.09506463	23.21692507	2.987261744	7.771975494	7.73E-15	1.24E-11	2235
Gtf2ird1	45.44948756	23.42319216	2.987173187	7.841256831	4.46E-15	8.58E-12	3487
Erc1	56.4164708	23.72314507	2.98706727	7.941952064	1.99E-15	4.43E-12	8356
Stat3	139.7767755	23.73691542	2.986805524	7.947258443	1.91E-15	4.43E-12	2506
Rreb1	95.29432381	23.74977049	2.986888217	7.951342256	1.85E-15	4.43E-12	8395
Spats2l	69.252571	24.00999092	2.986985901	8.038200285	9.12E-16	2.57E-12	2315
Synm	88.83101444	24.3615825	2.986907081	8.156123319	3.46E-16	1.16E-12	2697
Kif1a	194.638917	24.57217222	2.986755569	8.227044914	1.92E-16	7.39E-13	5391
Ckmt1	105.6162621	24.60003704	2.986862771	8.236078764	1.78E-16	7.39E-13	851
Baz2a	130.0568146	24.89273898	2.986818705	8.334198169	7.80E-17	3.67E-13	8385
Flot1	138.2100407	24.9722443	2.986807471	8.360848346	6.23E-17	3.67E-13	1383
Flot2	139.9584275	24.99666758	2.986805232	8.369031672	5.81E-17	3.67E-13	2699
Rpl22	145.3683636	25.04916804	2.986798645	8.386627628	5.00E-17	3.67E-13	2051
Scn8a	233.6444727	25.65525574	2.986734267	8.589734958	8.72E-18	1.85E-13	7120
Add2	290.9506269	25.93317119	2.95494577	8.776191922	1.69E-18	7.16E-14	2289
