# Supplementary material for: Gender-different effect of Src family kinases antagonism on photophobia and trigeminal ganglion activity
Source: J Headache Pain. 2024 Oct 11;25(1):175. doi: 10.1186/s10194-024-01875-3 (PMC11468534; doi:10.1186/s10194-024-01875-3)
Supplement: Supplementary file 5 — Supplementary Material 5: S5 Table. Statistic data for Figs. 2, 3, 4 and 6–7 [file 10194_2024_1875_MOESM5_ESM.pdf]

S5 Table. Statistic data for Figures 2-4 and 6-7

Figures	Groups	Passed normality (Shapiro-Wilk test)?	Statistic and calculations	P value	Mean±SEM, sample number
Fig.2 A (Male & Female total time in light)	Self-control Vs DMSO (male)	Yes	Paired t test (two-tailed)	ns p=0.2849	Self-control: 416.3±74.08 (n=10) DMSO:536.8±102 (n=10)
	Self-control Vs UMB (male)	No	Paired t test (two-tailed)	*** p=0.0010	Self-control: 455.1±52.34 (n=11) UMB:248.2±53.85 (n=11)
	Self-control Vs SRCT+UMB (male)	Yes	Paired t test (two-tailed)	ns p=0.1083	Self-control: 455.1±52.40 (n=10) SRCT+UMB:546.7±72.11 (n=10)
	Self-control Vs DMSO (Female)	Yes	Paired t test (two-tailed)	** p=0.0070	Self-control: 554.5±93.12 (n=12) DMSO:326.4±68.25 (n=12)
	Self-control Vs UMB (Female)	No	Paired t test (two-tailed)	*** p=0.0002	Self-control: 512.9±49.80 (n=13) UMB:149.0±24.82 (n=13)
	Self-control Vs SRCT+UMB (Female)	Yes	Paired t test (two-tailed)	*** p=0.0001	Self-control: 569.3±84.00 (n=12) SRCT+UMB:183.1±47.33 (n=12)
Fig.2 B (Total time in light)	Self-control Vs DMSO (male)	Yes	Paired t test (two-tailed)	ns p=0.2849	Self-control: 416.3±74.08 (n=10) DMSO:536.8±102 (n=10)
	Self-control Vs UMB (male)	No	Paired t test (two-tailed)	*** p=0.0010	Self-control: 455.1±52.34 (n=11) UMB:248.2±53.85 (n=11)
	Self-control Vs SRCT+UMB (male)	Yes	Paired t test (two-tailed)	ns p=0.1083	Self-control: 455.1±52.40 (n=10) SRCT+UMB:546.7±72.11 (n=10)
	DMSO Vs UMB (male)	Yes	Kruskal–Walli one-way ANOVA followed by Mann-Whitney test	# p=0.0182	DMSO:536.8±102 (n=10) UMB:248.2±53.85 (n=11)
	UMB Vs SRCT+UMB (male)	No	Kruskal–Walli one-way ANOVA followed by Mann-Whitney test	## p=0.0075	UMB:248.2±53.85 (n=11) SRCT+UMB:546.7±72.11 (n=10)
	Self-control Vs DMSO (Female)	Yes	Paired t test (two-tailed)	** p=0.0070	Self-control: 554.5±93.12 (n=12) DMSO:326.4±68.25 (n=12)
	Self-control Vs UMB (Female)	No	Paired t test (two-tailed)	*** p=0.0002	Self-control: 512.9±49.80 (n=13) UMB:149.0±24.82 (n=13)

	Self-control Vs SRCT+UMB (Female)	Yes	Paired t test (two-tailed)	*** p=0.0001	Self-control: 569.3±84.00 (n=12) SRCT+UMB:183.1±47.33 (n=12)
	DMSO Vs UMB (Female)	No	Kruskal–Walli one-way ANOVA followed by Mann-Whitney test	ns p=0.0686	DMSO:326.4±68.25 (n=12) UMB:149.0±24.82 (n=13)
	UMB Vs SRCT+UMB (Female)	No	Kruskal–Walli one-way ANOVA followed by Mann-Whitney test	ns p>0.9999	UMB:149.0±24.82 (n=13) SRCT+UMB:183.1±47.33 (n=12)
Fig.2 C (Total time in light fold change)	DMSO Vs UMB (Male)	Yes	Kruskal–Walli one-way ANOVA, followed by Mann-Whitney test	* p=0.0185	DMSO:0.9805±0.09286 (n=8) UMB:0.4982±0.09587 (n =11)
	UMB Vs SRCT+UMB (Male)	No	Kruskal–Walli one-way ANOVA followed by Mann-Whitney test	*** p=0.0005	UMB:0.4982±0.09587 (n=11) SRCT+UMB:1.306±0.2006 (n=10)
	DMSO Vs UMB (Female)	Yes	Kruskal–Walli one-way ANOVA followed by Mann-Whitney test	* p=0.0155	DMSO:0.6291±0.08075 (n=12) UMB:0.3256±0.06432 (n=13)
	UMB Vs SRCT+UMB (Female)	Yes	Kruskal–Walli one-way ANOVA followed by Mann-Whitney test	ns p=0.9184	UMB:0.3256±0.06432 (n=13) SRCT+UMB:0.2934±0.05978 (n=12)
Fig.2 D (Total time in light male Vs female)	Male DMSO Vs Female DMSO	Yes	Paired t test (two-tailed)	ns p=0.1056	Male DMSO:536.8±102.0 (n=10) Female DMSO:326.4±68.25 (n=12)
	Male UMB Vs Female UMB	No	Paired t test (two-tailed)	ns p=0.3031	Male UMB:248.2±53.85 (n=11) Female UMB:149.0±24.82 (n=13)
	Male SRCT+UMB	Yes	Paired t test (two-tailed)	*** p=0.0007	Male SRCT+UMB:546.7±72.11 (n=10)

	Vs Female SRCT+UMB				Female SRCT+UMB:183.1±47.33 (n=12)
Fig.2 E (Total time in light fold change male Vs female)	Male DMSO Vs Female DMSO	Yes	Paired t test (two-tailed)	* p=0.0115	Male DMSO:0.9805±0.09286 (n=8) Female DMSO:0.6291±0.08075 (n=12)
	Male UMB Vs Female UMB	Yes	Paired t test (two-tailed)	ns p=0.1522	Male UMB:0.4982±0.09587 (n=11) Female UMB:0.3256±0.06432 (n=13)
	Male SRCT+UMB Vs Female SRCT+UMB	No	Paired t test (two-tailed)	**** p<0.0001	Male SRCT+UMB:1.306±0.2006 (n=10) Female SRCT+UMB:0.2934±0.05978 (n=12)
Fig.3 A (Male & female transitions)	Self-control Vs DMSO	Yes	Paired t test (two-tailed)	ns p=0.5177	Self-control:43.40±8.394 (n=10) DMSO: 37.50±6.330 (n=10)
	Self-control Vs UMB	Yes	Paired t test (two-tailed)	** p=0.0022	Self-control: 52.50±8.422 (n=11) UMB: 23.86±5.932 (n=11)
	Self-control Vs SRCT+UMB	Yes	Paired t test (two-tailed)	ns p=0.4062	Self-control: 46.45±5.650 (n=10) SRCT+UMB: 42.40±5.285 (n=10)
	Self-control Vs DMSO	Yes	Paired t test (two-tailed)	** *p*=0.0064	Self-control: 108.0±16.49 (n=12) DMSO: 42.55±7.430 (n=11)
	Self-control Vs UMB	Yes	Paired t test (two-tailed)	**** *p*<0.0001	Self-control: 110.7±8.666 (n=13) UMB: 26.62±4.720 (n=13)
	Self-control Vs SRCT+UMB	Yes	Paired t test (two-tailed)	*** *p*=0.0003	Self-control: 112.8±13.07 (n=9) SRCT+UMB: 17.78±3.063 (n=9)
Fig.3 B (Transitions)	Self-control Vs DMSO (Male)	Yes	Paired t test (two-tailed)	ns p=0.5177	Self-control:43.40±8.394 (n=10) DMSO: 37.50±6.330 (n=10)
	Self-control Vs UMB (Male)	Yes	Paired t test (two-tailed)	** p=0.0022	Self-control: 52.50±8.422 (n=11) UMB: 23.86±5.932 (n=11)
	Self-control Vs SRCT+UMB (Male)	Yes	Paired t test (two-tailed)	ns p=0.4062	Self-control: 46.45±5.650 (n=10) SRCT+UMB: 42.40±5.285 (n=10)
	DMSO Vs UMB (Male)	Yes	Kruskal–Walli one-way ANOVA followed by Mann-Whitney test	ns p=0.2676	DMSO: 37.50±6.330 (n=10) UMB: 23.86±5.932 (n=11)
	UMB Vs SRCT+UMB (Male)	Yes	Kruskal–Walli one-way ANOVA	ns p=0.0959	UMB: 23.86±5.932 (n=11) SRCT+UMB: 42.40±5.285 (n=10)

			followed by Mann-Whitney test		
	Self-control Vs DMSO (Female)	Yes	Paired t test (two-tailed)	** p=0.0064	Self-control: 108.0±16.49 (n=11) DMSO: 42.55±7.430 (n=11)
	Self-control Vs UMB (Female)	Yes	Paired t test (two-tailed)	**** *p*<0.0001	Self-control: 110.7±8.666 (n=13) UMB: 26.62±4.720 (n=13)
	Self-control Vs SRCT+UMB (Female)	Yes	Paired t test (two-tailed)	*** *p*=0.0003	Self-control: 112.8±13.07 (n=9) SRCT+UMB: 17.78±3.063 (n=9)
	DMSO Vs UMB (Female)	Yes	Kruskal–Walli one-way ANOVA followed by Mann-Whitney test	ns *p*=0.0803	DMSO: 42.55±7.430 (n=11) UMB: 26.62±4.720 (n=13)
	UMB Vs SRCT+UMB (Female)	Yes	Kruskal–Walli one-way ANOVA followed by Mann-Whitney test	ns *p*=0.4534	UMB: 26.62±4.720 (n=13) SRCT+UMB: 17.78±3.063 (n=9)
Fig.3 C (Transitions fold change)	DMSO Vs UMB (Male)	Yes	Kruskal–Walli one-way ANOVA followed by Mann-Whitney test	ns *p*=0.0708	DMSO:0.8931±0.1690 (n=9) UMB:0.4574±0.1173 (n=11)
	UMB Vs SRCT+UMB (Male)	No	Kruskal–Walli one-way ANOVA followed by Mann-Whitney test	* *p*=0.0113	UMB: 0.4574±0.1173 (n=11) SRCT+UMB:1.000±0.1523 (n=10)
	DMSO Vs UMB (Female)	Yes	Kruskal–Walli one-way ANOVA followed by Mann-Whitney test	* *p*=0.0180	DMSO: 0.5343±0.08546 (n=12) UMB: 0.2500±0.04524 (n=13)
	UMB Vs SRCT+UMB (Female)	No	Kruskal–Walli one-way ANOVA followed by	ns *p*=0.9249	UMB: 0.2500±0.04524 (n=13) SRCT+UMB: 0.2765±0.06048 (n=12)

			Mann-Whitney test		
Fig.3 D (Transitions male Vs female)	Male DMSO Vs Female DMSO	Yes	Paired t test (two-tailed)	ns *p*=0.6113	Male DMSO:37.50±6.330 (n=10) Female DMSO: 42.55±7.430 (n=11)
	Male UMB Vs Female UMB	Yes	Paired t test (two-tailed)	ns *p*=0.7204	Male UMB: 23.86±5.932 (n=11) Female UMB: 26.62±4.720 (n=13)
	Male SRCT+UMB Vs Female SRCT+UMB	Yes	Paired t test (two-tailed)	** *p*=0.0012	Male SRCT+UMB:42.40±5.285 (n=10) Female SRCT+UMB: 17.78±3.063 (n=10)
Fig.3 E (Transitions fold change male Vs female)	Male DMSO Vs Female DMSO	Yes	Paired t test (two-tailed)	ns *p*=0.0824	Male DMSO:0.8931±0.1690 (n=9) Female DMSO:0.5343±0.08546 (n=12)
	Male UMB Vs Female UMB	Yes	Paired t test (two-tailed)	ns *p*=0.1231	Male UMB: 0.4574±0.1173 (n=11) Female UMB: 0.25±0.04524 (n=13)
	Male SRCT+UMB Vs Female SRCT+UMB	No	Paired t test (two-tailed)	*** *p*=0.0001	Male SRCT+UMB: 0.8680±0.08493 (n=9) Female SRCT+UMB: 0.2765±0.06048 (n=12)
Fig.4 A Male CGRP hypothalamus	DMSO Vs UMB	Yes	Kruskal–Walli one-way ANOVA followed by Mann-Whitney test	ns *p*>0.9999	DMSO: 114.9±35.05 (n=10) UMB:126.4±12.28 (n=10)
	UMB Vs SRCT+UMB	Yes	Kruskal–Walli one-way ANOVA followed by Mann-Whitney test	ns *p*=0.8020	UMB: 126.4±12.28 (n=10) SRCT+UMB:160.7±20.64 (n=11)
Fig.4 B Female CGRP hypothalamus	DMSO Vs UMB	Yes	Kruskal–Walli one-way ANOVA followed by Mann-Whitney test	ns *p*=0.1821	DMSO: 140.5±24.96 (n=10) UMB: 214.90±32.02 (n=15)
	UMB Vs SRCT+UMB	No	Kruskal–Walli one-	ns *p*>0.9999	UMB: 214.90±32.02 (n=15) SRCT+UMB: 207.6±18.45 (n=13)

			way ANOVA followed by Mann-Whitney test		
Fig.4 C CGRP hypothalamus (Male Vs female)	Male DMSO Vs Female DMSO	No	Paired t test (two-tailed)	ns *p*=0.7394	Male DMSO: 114.9±35.05 (n=10) Female DMSO: 140.5±24.96 (n=10)
	Male UMB Vs Female UMB	Yes	Paired t test (two-tailed)	* *p*=0.0189	Male UMB: 126.4±12.28 (n=10) Female UMB: 214.9±32.02 (n=15)
	Male SRCT+UMB Vs Female SRCT+UMB	No	Paired t test (two-tailed)	ns *p*=0.1339	Male SRCT+UMB:160.7±20.64 (n=11) Female SRCT+UMB:207.6±18.45 (n=13)
Fig.4 D Male PACAP hypothalamus	DMSO Vs UMB	No	Kruskal–Walli one-way ANOVA followed by Mann-Whitney test	ns *p*>0.9999	DMSO: 13.29±1.514 (n=10) UMB: 12.52±0.9416 (n=10)
	UMB Vs SRCT+UMB	No	Kruskal–Walli one-way ANOVA followed by Mann-Whitney test	ns *p*>0.9999	UMB: 12.52±0.9416 (n=10) SRCT+UMB: 11.66±1.112 (n=10)
Fig.4 E Female PACAP hypothalamus	DMSO Vs UMB	Yes	Kruskal–Walli one-way ANOVA followed by Mann-Whitney test	ns *p*>0.9999	DMSO: 24.11±2.186 (n=10) UMB: 23.45±1.608 (n=13)
	UMB Vs SRCT+UMB	No	Kruskal–Walli one-way ANOVA followed by Mann-Whitney test	ns *p*=0.1290	UMB: 23.45±1.608 (n=13) SRCT+UMB: 28.09±1.963 (n=12)
Fig.4 F PACAP hypothalamus	Male DMSO Vs Female DMSO	Yes	Paired t test (two-tailed)	*** *p*=0.0009	Male DMSO: 13.29±1.514 (n=10) Female DMSO: 24.11±2.186 (n=10)

S5 Table. Statistic data for Figures 2-4 and 6-7

(Male Vs female)					
	Male UMB Vs Female UMB	No	Paired t test (two-tailed)	****<br>$p<0.0001$	Male UMB: 12.52±0.9416 (n=10)<br>Female UMB: 23.45±1.608 (n=13)
	Male SRCT+UMB Vs Female SRCT+UMB	No	Paired t test (two-tailed)	****<br>$p<0.0001$	Male SRCT+UMB: 11.66±1.112 (n=10)<br>Female SRCT+UMB: 28.09±1.963 (n=12)

Figures	Groups	Statistic and calculations	Adjust p-value	Mean ± SEM, sample nubmer
Fig.6 A (*Crebbp* fold change in male)	UMB *Vs* DMSO	Wald test	*<br>padj= 0.0479	DMSO: 5220 ± 373.7 (*n* =7)<br>UMB: 4218 ± 93.26 (*n* =7)<br>UMB_SRCT : 5180 ± 330.2 (*n* =7)
	UMB_SRCT *Vs* UMB	Wald test	*<br>padj = 0.0439	
Fig.6 B (*Trpm3* fold change in male)	UMB *Vs* DMSO	Wald test	**<br>padj = 0.0233	DMSO: 351.6 ± 30.30 (*n* =7)<br>UMB: 226.3 ± 24.05 (*n* =7)<br>UMB_SRCT : 377.7 ± 27.77 (*n* =7)
	UMB_SRCT *Vs* UMB	Wald test	*<br>padj = 0.0088	
Fig.6 C (*Zmynd8* fold change in male)	UMB *Vs* DMSO	Wald test	****<br>padj = 4.81E-11	DMSO: 0 ± 0 (*n* =7)<br>UMB: 52.90 ± 37.13 (*n* =7)<br>UMB_SRCT : 0 ± 0 (*n* =7)
	UMB_SRCT *Vs* UMB	Wald test	****<br>padj = 1.74E-11	
Fig.6 D (*Akt1* fold change in male)	UMB *Vs* DMSO	Wald test	**<br>padj = 0.0003	DMSO: 8834 ± 111.4 (*n* =7)<br>UMB: 7719 ± 179.6 (*n* =7)<br>UMB_SRCT : 9225 ± 464.7 (*n* =7)
	UMB_SRCT *Vs* UMB	Wald test	*<br>padj = 0.0349	
Fig.6 E (*Atp5a1* fold change in male)	UMB *Vs* DMSO	Wald test	*<br>padj = 0.0018	DMSO: 18321 ± 220.5 (*n* =7)<br>UMB: 16360 ± 453.2 (*n* =7)<br>UMB_SRCT : 19150 ± 650.4 (*n* =7)
	UMB_SRCT *Vs* UMB	Wald test	*<br>padj = 0.0052	
Fig.6 F (*Scn8a* fold change in male)	UMB *Vs* DMSO	Wald test	****<br>padj = 6.53E-14	DMSO: 490.1 ± 282.0 (*n* =7)<br>UMB: 0 ± 0 (*n* =7)<br>UMB_SRCT : 449.9 ± 354.5 (*n* =7)
	UMB_SRCT *Vs* UMB	Wald test	****<br>padj = 1.85E-13	

Figures	Groups	Passed normality (Shapiro-Wilk test)?	Statistic and calculations	P value	Mean ± SEM, sample number
Fig.7 A	UMB *Vs* DMSO	Yes	Unpaired t test	*	Male:

(*Crebbp* fold change in male *Vs* female)	(male)		(one-tailed)	*p* = 0.0295	DMSO: 1.000 ± 0.1250 (*n* =7)<br>UMB: 1.391 ± 0.1399 (*n* =7)<br>UMB_SRCT : 0.9586 ± 0.04221 (*n* =6)
	UMB_SRCT *Vs* UMB (male)	No	Mann-Whitney test (one-tailed)	*<br>*p* = 0.0175	
	UMB *Vs* DMSO (female)	Yes	Unpaired t test (one-tailed)	ns<br>*p* = 0.1320	Female:<br>DMSO: 1.000 ± 0.1593 (*n* =12)<br>UMB: 0.7813 ± 0.1051 (*n* =12)<br>UMB_SRCT : 0.8805 ± 0.1440 (*n* =11)
	UMB_SRCT *Vs* UMB (female)	No	Mann-Whitney test (one-tailed)	ns<br>*p* = 0.4640	
	male *Vs* female in DMSO	Yes	Unpaired t test (one-tailed)	ns<br>*p* = 0.5000	
	male *Vs* female in UMB	Yes	Unpaired t test (one-tailed)	##<br>*p* = 0.0014	
	male *Vs* female in UMB_SRCT	No	Mann-Whitney test (one-tailed)	ns<br>*p* = 0.2624	
Fig.7 B (*Trpm3* fold change in male *Vs* female)	UMB *Vs* DMSO (male)	Yes	Unpaired t test (one-tailed)	*<br>*p* = 0.0489	Male:<br>DMSO: 1.000 ± 0.09371 (*n* =7)<br>UMB: 1.253 ± 0.1050 (*n* =7)<br>UMB_SRCT : 0.7406 ± 0.04260 (*n* =6)
	UMB_SRCT *Vs* UMB (male)	No	Mann-Whitney test (one-tailed)	**<br>*p* = 0.0012	
	UMB *Vs* DMSO (female)	Yes	Unpaired t test (one-tailed)	ns<br>*p* = 0.0534	Female:<br>DMSO: 1.000 ± 0.1292 (*n* =12)<br>UMB: 0.7440 ± 0.08067 (*n* =12)<br>UMB_SRCT : 0.7020 ± 0.06399 (*n* =11)
	UMB_SRCT *Vs* UMB (female)	No	Mann-Whitney test (one-tailed)	ns<br>*p* = 0.2433	
	male *Vs* female in DMSO	Yes	Unpaired t test (one-tailed)	ns<br>*p* = 0.5000	
	male *Vs* female in UMB	Yes	Unpaired t test (one-tailed)	###<br>*p* = 0.0007	
	male *Vs* female in UMB_SRCT	No	Mann-Whitney test (two-tailed)	ns<br>*p* = 0.4038	

Fig.7 C (*Zmynd8* fold change in male *Vs* female)	UMB *Vs* DMSO (male)	No	Mann-Whitney test (one-tailed)	* p =0.0131	Male: DMSO: 1.000 ± 0.06578 (n =7) UMB: 1.310 ± 0.08986 (n =7) UMB_SRCT : 0.9728 ± 0.04428 (n =6)
	UMB_SRCT *Vs* UMB (male)	No	Mann-Whitney test (one-tailed)	* p = 0.0111	
	UMB *Vs* DMSO (female)	Yes	Unpaired t test (one-tailed)	ns p = 0.1758	Female: DMSO: 1.000 ± 0.1360 (n =12) UMB: 0.8358± 0.1061 (n =12) UMB_SRCT : 0.8469 ± 0.1304 (n =12)
	UMB_SRCT *Vs* UMB (female)	Yes	Unpaired t test (one-tailed)	ns p = 0.9481	
	male *Vs* female in DMSO	Yes	Welch's t test (one-tailed)	ns p = 0.5000	
	male *Vs* female in UMB	Yes	Unpaired t test (one-tailed)	## p = 0.0037	
	male *Vs* female in UMB_SRCT	No	Mann-Whitney test (two-tailed)	ns p = 0.3845	
Fig.7 D (*Akt1* fold change in male *Vs* female)	UMB *Vs* DMSO (male)	Yes	Unpaired t test (one-tailed)	* p=0.0467	Male: DMSO: 1.000 ± 0.08423 (n =7) UMB: 1.200 ± 0.06995 (n =7) UMB_SRCT : 1.283 ± 0.04914 (n =7)
	UMB_SRCT *Vs* UMB (male)	Yes	Unpaired t test (one-tailed)	ns p = 0.1739	
	UMB *Vs* DMSO (female)	Yes	Unpaired t test (one-tailed)	ns p = 0.2662	Female: DMSO: 1.000± 0.1131 (n =12) UMB: 0.8909 ± 0.1295 (n =12) UMB_SRCT : 0.1295 ± 0.09827 (n =12)
	UMB_SRCT *Vs* UMB (female)	Yes	Unpaired t test (one-tailed)	ns p = 0.2050	
	male *Vs* female in DMSO	Yes	Unpaired t test (one-tailed)	ns p = 0.5000	
	male *Vs* female in UMB	Yes	Welch's t test (one-tailed)	# p = 0.0262	
	male *Vs* female in UMB_SRCT	Yes	Welch's t test (one-tailed)C	### p = 0.0001	

Fig.7 E (*Atp5a1* fold change in male *Vs* female)	UMB *Vs* DMSO (male)	Yes	Unpaired t test (one-tailed)	* *p* =0.0226	Male: DMSO: 1.000 ± 0.09068 (*n* =7) UMB: 1.232 ± 0.05076 (*n* =7) UMB_SRCT : 1.158 ± 0.04061 (*n* =7)
	UMB_SRCT *Vs* UMB (male)	Yes	Unpaired t test (one-tailed)	ns *p* = 0.1367	
	UMB *Vs* DMSO (female)	Yes	Unpaired t test (one-tailed)	ns *p* = 0.3495	Female: DMSO: 1.000 ± 0.1511 (*n* =12) UMB: 1.100 ± 0.2062 (*n* =12) UMB_SRCT : 0.9131 ± 0.1470 (*n* =12)
	UMB_SRCT *Vs* UMB (female)	Yes	Unpaired t test (one-tailed)	ns *p* = 0.2339	
	male *Vs* female in DMSO	Yes	Unpaired t test (one-tailed)	ns *p* = 0.5000	
	male *Vs* female in UMB	Yes	Unpaired t test (one-tailed)	ns *p* = 0.2726	
	male *Vs* female in UMB_SRCT	Yes	Welch's t test (one-tailed)	ns *p* = 0.0667	
Fig.7 F (*Scn8a* fold change in male *Vs* female)	UMB *Vs* DMSO (male)	No	Mann-Whitney test (one-tailed)	* *p* = 0.0427	Male: DMSO: 1.021 ± 0.03332 (*n* =6) UMB: 0.9095 ± 0.04819 (*n* =6) UMB_SRCT : 1.096 ± 0.08208 (*n* =6)
	UMB_SRCT *Vs* UMB (male)	No	Mann-Whitney test (one-tailed)	* *p* = 0.0465	
	UMB *Vs* DMSO (female)	Yes	Unpaired t test (one-tailed)	ns *p* = 0.1983	Female: DMSO: 1.000 ± 0.1068 (*n* =12) UMB: 0.8661 ± 0.1123 (*n* =12) UMB_SRCT : 0.7651 ± 0.1184 (*n* =12)
	UMB_SRCT *Vs* UMB (female)	Yes	Unpaired t test (one-tailed)	ns *p* = 0.5423	
	male *Vs* female in DMSO	No	Mann-Whitney test (one-tailed)	ns *p* = 0.3082	
	male *Vs* female in UMB	No	Mann-Whitney test (one-tailed)	ns *p* = 0.2766	

	male *Vs* female in UMB_SRCT	No	Mann-Whitney test (one-tailed)	# $p = 0.0207$	
	Self-control Vs ISO+UMB	No	Wilcoxon matched-pairs signed rank test (one-tailed)	ns p=0.0881	Self-control: 347.2±63.50 (n=12) ISO+UMB: 278.9±91.64 (n=12)
	Self-control Vs UMB	Yes	Paired t test (one-tailed)	** p=0.0018	Self-control: 480.3±65.42 (n=12) UMB: 192.8±51.90 (n=12)
	Self-control Vs ISO+UMB	No	Wilcoxon matched-pairs signed rank test (one-tailed)	ns p=0.0881	Self-control: 347.2±63.50 (n=12) ISO+UMB: 278.9±91.64 (n=12)
	UMB Vs DMSO	No	Mann-Whitney test (one-tailed)	ns p= 0.1532	
	ISO+UMB Vs UMB	No	Mann-Whitney test (one-tailed)	ns p= 0.1560	
